# Targeted resequencing identifies genes with recurrent variation in cerebral palsy

**DOI:** 10.1038/s41525-019-0101-z

**Published:** 2019-11-04

**Authors:** C. L. van Eyk, M. A. Corbett, M. S. B. Frank, D. L. Webber, M. Newman, J. G. Berry, K. Harper, B. P. Haines, G. McMichael, J. A. Woenig, A. H. MacLennan, J. Gecz

**Affiliations:** 1 Robinson Research Institute, Faculty of Health and Medical Sciences, University of Adelaide, Adelaide, SA Australia; 2 Adelaide Medical School, Faculty of Health and Medical Sciences, University of Adelaide, Adelaide, SA Australia; 3 Alzheimer’s Disease Genetics Laboratory, Centre for Molecular Pathology, School of Biological Sciences, University of Adelaide, Adelaide, SA Australia; 4 South Australian Health and Medical Research Institute, Adelaide, SA Australia

**Keywords:** Molecular medicine, Genetic testing, Genetic testing, Neurodevelopmental disorders, Paediatric neurological disorders

## Abstract

A growing body of evidence points to a considerable and heterogeneous genetic aetiology of cerebral palsy (CP). To identify recurrently variant CP genes, we designed a custom gene panel of 112 candidate genes. We tested 366 clinically unselected singleton cases with CP, including 271 cases not previously examined using next-generation sequencing technologies. Overall, 5.2% of the naïve cases (14/271) harboured a genetic variant of clinical significance in a known disease gene, with a further 4.8% of individuals (13/271) having a variant in a candidate gene classified as intolerant to variation. In the aggregate cohort of individuals from this study and our previous genomic investigations, six recurrently hit genes contributed at least 4% of disease burden to CP: *COL4A1*, *TUBA1A, AGAP1*, *L1CAM*, *MAOB* and *KIF1A*. Significance of Rare VAriants (SORVA) burden analysis identified four genes with a genome-wide significant burden of variants, *AGAP1*, *ERLIN1*, *ZDHHC9* and *PROC*, of which we functionally assessed *AGAP1* using a zebrafish model. Our investigations reinforce that CP is a heterogeneous neurodevelopmental disorder with known as well as novel genetic determinants.

## Introduction

Cerebral palsy (CP) is the most common motor disability of childhood, with a frequency of around 2 per 1000 live births.^1,2^ CP encompasses a clinically heterogeneous spectrum of disorders of movement, posture or motor function, which are collectively defined by being permanent and non-progressive. These disorders are the result of a lesion or abnormality in the developing brain occurring in the antenatal, perinatal or early postnatal period. Variable patterns of neuropathology are observed on brain imaging, and for most cases the aetiology of the brain injury is not well understood. A number of neurodevelopmental problems frequently co-occur with CP, including intellectual disability (ID), autism spectrum disorder (ASD), epilepsy and visual and hearing impairment.

A number of observations have long suggested a likely genetic contribution to CP: the increased concordance rate for CP in monozygotic twins compared to dizygotic twins,^3^ the higher risk of CP in consanguineous families^4–6^ and the frequent co-occurrence of CP with comorbidities with known genetic contribution. Causative gene variants have been identified in large CP pedigrees.^7–11^

Recent studies utilising genome-wide arrays^12–14^ and next-generation sequencing technologies^15–18^ have begun to unravel the genetic contribution to CP. In sporadic cases, the majority of causative variants have been shown to be de novo, a trend that is also observed in other neurodevelopmental disorders such as ID and epilepsy.^19,20^ Whole-exome sequencing (WES) studies to date have reported varying diagnostic rates, seemingly at least partly dependent upon the clinical selection criteria used, ranging from 14% in our clinically unselected cohort,^15^ to up to 57% of cases in homogenous cohorts with extensive prior work-up.^16,17^ The modest reported diagnostic rate reported in McMichael et al.^15^ is likely also due in part to the stringent criteria used to select causative variants.

The interpretation of genetic variants in CP is complicated by the fact that the majority of the cases are singletons and that CP encompasses a range of movement problems of variable clinical severity, with or without comorbidities. Consequently, large cohorts of sporadic cases need to be sequenced before a gene achieves sufficient hits for significance to be supported through, for example, burden analysis.^21^ In light of this, we designed a custom CP gene panel also including selected candidate CP genes from the literature (Supplementary Table 1) with the aim of identifying recurrently variant CP genes. Here we report the results of this effort of resequencing of 366 CP cases, including 95 cases previously tested by WES.

## Results

In total, we analysed 403 CP cases using our custom-designed gene panel, including re-screening 97 cases previously examined by WES to compare the detection rate of known and novel variants for the 112 genes in the HaloPlex gene panel. Overall, a median of 96.7% (minimum 86.5%, maximum 99.2%) of targeted bases were covered by at least 20 reads across all samples, with a median of 98.9% (minimum 95.2%, maximum 99.7%) of targeted regions covered by at least 20 reads across all samples (Supplementary Table 2, Supplementary Fig. 1). For the 97 samples previously examined by WES, we also calculated coverage across the regions targeted by the HaloPlex gene panel (Supplementary Fig. 2). We achieved median coverage of 355.5× coverage of targeted regions with the HaloPlex gene panel across these samples, compared to median coverage of 75.6× for these same target regions by WES. A total of 37 samples of the 403 cases (including two samples previously examined by WES) were excluded from further analysis after failing quality control (>50% of targeted bases with <20× coverage), therefore the final number of samples analysed was 366, including 271 naïve cases and 95 re-sequenced cases. Principal Component Analysis did not demonstrate bias in coverage of targeted regions when either sample type (buccal or Lymphoblastoid cell line) or sequencing platform were considered (Supplementary Fig. 3).

Variants detected in samples that passed quality control were filtered for likely pathogenicity as described in Methods. Prioritised variants validated in this study are listed in Supplementary Tables 3–5. We validated a total of 135 variants in 103 new cases, therefore 103/271 new cases (38%) in this study had at least one variant of interest (Supplementary Table 5). Considering cases sequenced with both technologies, the HaloPlex gene panel and WES technologies together detected 76 high-quality variants in 95 cases. Of these 76 variants, 69 were identified by WES, while a total of 70 variants were detected using the HaloPlex gene panel, including seven variants not covered by the WES data. Two variants detected by WES were not called by SureCall, but manual inspection showed that they were covered, and an additional four variants were not covered well by this gene panel, including two pathogenic variants in *TUBA1A*. Therefore, if we consider the joint set of high-quality variants detected in these 95 samples from WES plus HaloPlex as the ‘gold-standard’, the gene panel approach successfully identified 70/76 variants, achieving comparable sensitivity to WES, which identified 69/76 variants.

We identified 23 individuals (6% of the 366 cases) with variants of potential clinical relevance in genes associated with other disorders (Table 1, Supplementary Table 6). Of these, seven individuals harboured variants that were classified as pathogenic, and a further two as likely pathogenic according to ACMG guidelines.^22^ We have previously reported two of these variants (Table 1 and ref. ^15^): a likely pathogenic de novo variant in *MAST1* in P009 (NM_014975.3: c.1499C>T: p.P500L) and a de novo pathogenic variant in *KDM5C* in P026 (NM_001146702.1: c.1238C>T: p.P413L). For an additional two individuals, the variants were detected by WES but were not classified as pathogenic at the time of the analysis. These variants are a pathogenic *COL4A1* variant in P033 (NM_001845.5: c.2413G>A: p.G805R) and compound heterozygous pathogenic variants in the protein C gene, *PROC* (NM_000312.3: c.169C>T: p.R57W / NM_000312.3: c.814C>T: p.R272C) in P052. For P033, we have also reported a de novo splice site variant in *AGAP1*, which was predicted to be likely pathogenic (Table 2 and ref. ^15^). One individual with the *COL4A1* p.G805R variant has previously been reported: a 25-year-old male who, following infantile hemiparesis, had repeated deep intracerebral haemorrhages from 17 years of age.^23^ The contribution of each of these variants requires further investigation, particularly given the reported clinical heterogeneity and reduced penetrance of *COL4A1* mutations.^24^ In the case of P052, the subsequent availability of further clinical information revealed a family history of progressive neurodevelopmental decline and death from an obscure degenerative leuko-encephalopathy in two female siblings of the proband. P052 had a complex clinical phenotype, with mixed spastic/dyskinetic quadriplegic CP, epilepsy, mild ID and a diagnosis of possible Gardner–Diamond syndrome at 18 years of age. Periventricular cystic porencephaly with bilateral frontal pachygyria was observed by magnetic resonance imaging (MRI) at 2 years of age. Compound heterozygous mutations in *PROC* have been previously associated with familial CP,^25^ with clinical features including global developmental delay, cortical visual blindness, spastic diplegia and recurrent purpura fulminans. Brain MRIs typically show bilateral periventricular cystic porencephaly, with loss of cerebral white matter.

**Table 1:** Cases with variants of possible clinical significance in known disease genes

Sample	Gene	Inheritance	gnomAD frequency	Variant	dbSNP ID, ACMG classification
P009^a^	MAST1	De novo	0	c.1499C>T:p.P500L^b^	Likely pathogenic
P015^a^	PAK3	X-linked	0	c.1477C>T:p.R493C^b^	Uncertain significance
P026^a^	KDM5C	De novo	0	c.1238C>T:p.P413L^b^	rs1057518697 Pathogenic
P033^a^	COL4A1	Paternal	0	c.2413G>A:p.G805R	Pathogenic
P035^a^	COL4A1	Not maternal	8.12E−06	c.136G>A:p.G46R	Uncertain significance
P052^a^	PROC	Paternal	2.53E−05	c.169C>T:p.R57W	rs757583846 Pathogenic
		Maternal	2.53E−05	c.814C>T:p.R272C	rs121918154 Pathogenic
P106^a^	COL4A1	Paternal	0	c.4516A>G:p.N1506D	Uncertain significance
P174	KIF1A	Not maternal, father unavailable	0	c.296C>T:p.T99M	rs387906799 Pathogenic
P204	COL4A1	Not maternal	0	c.2494G>A:p.G832R	rs797044867 Pathogenic
P718	NT5C2	Homozygous—identical by descent	0	c.115C>T:p.R39*	Pathogenic
P724	L1CAM	X-linked	0	c.2137C>T:p.P713S	Likely pathogenic
P773	MAST1	Not maternal	8.12E−06	c.1066G>A:p.D356N	Uncertain significance
P781	KIF1A	De novo	0	c.946C>T:p.R316W	rs672601370 Pathogenic
P904	SCN8A	De novo, also present in identical twin	4.06E−06	c.4724C>T:p.A1575V	Uncertain significance
P915	HUWE1	Not maternal, also present in twin	5.60E−06	c.6391T>A:p.L2131M	Likely benign
P968	BRWD3	Unknown	0	c.3088G>A:p.V1030I	Uncertain significance
P981	COL4A1	Not maternal	6.63E−05	c.2447C>T:p.P816L	Uncertain significance
P1102	HUWE1	Paternal	1.69E−05	c.8558A>T:p.E2853V	Uncertain significance
P1116	COL4A1	Maternal	7.21E−05	c.4856G>A:p.R1619H	Uncertain significance
P1147	IQSEC2	Paternal	0	c.1753C>T:p.R585W	Likely benign
10249	HUWE1	Unknown	5.60E−06	c.6500C>T:p.A2167V	Uncertain significance
10894	SYNGAP1	Not maternal	0	c.3436C>G:p.P1146A	Uncertain significance
14986	MAST1	Paternal	1.44E−05	c.421G>A:p.E141K	Uncertain significance

See Supplementary Table 6 for full version of this table including clinical information

*gnomAD* genome aggregation database frequency

*indicates translation termination codon

^a^Case reported in ref. ^15^

^b^Variant reported in ref. ^15^

**Table 2:** Variation intolerant CP candidate genes harbouring variants of potential clinical significance (this study and ref. ^15^)

Gene	Sample	Inheritance	gnomAD frequency	Variant	CADD Phred	GERP++	MTR centile	Poly-Phen2 HVAR	Mut. Taster
ADCY3	P443^a^	Paternal	0	c.1138C>T: p.R380W^b^	33	4.60	0.79	D	D
	P947	Unknown	4.07E−06	c.1789C>T: p.R597*	43	4.21	–	–	A
	16922	Not maternal	2.04E−05	c.2042C>A: p.A681D	26.7	5.40	49.08	D	D
AGAP1	P033^a^	De novo	0	Splicing c.957+1G>A^b^	27.3	5.08	–	–	D
	P738	De novo	1.94E−04	c.1400C>G: p.P467R	27.4	4.21	77.01	P	D
	P1126	Not maternal	0	c.1232C>T: p.P411L	33	4.66	11.42	D	D
COPS3	16458	Not maternal	0	c.7A>T: p.S3C	24.6	5.52	92.08	P	D
GAD1	P176	Not maternal	4.06E−06	c.773A>G: p.Y258C	26.3	5.67	33.11	D	D
	10635	Not maternal	1.65E−05	c.1208C>T: p.P403L	34	5.91	20.17	D	D
INHBB	P058^a^	De novo	8.21E−06	c.472C>T: p.R158C^b^	26.2	5.09	30.77	B	D
KDM7A	P105^a^	De novo	0	c.2180C>G: p.S727W^b^	34	5.85	36.31	D	D
	11348	Unknown	1.22E−05	c.484G>T: p.V162F	32	5.37	20.59	D	D
MAOB	P025^a^	X-linked	0	Splicing c.1138-1G>A^b^	23.8	6.17	–	–	D
	P216	X-linked	0	c.980C>T: p.T327M	32	5.47	25.58	D	D
	P915	Not maternal, also present in twin	0	c.980C>T: p.T327M	32	5.47	25.58	D	D
NAA35	P117^a^	De novo	0	c.1596G>T: p.W532C^b^	33	5.40	20.14	D	D
	P783	Unknown	0	c.134T>C: p.L45S	27	3.94	28.97	D	D
RNF214	P007^a^	De novo	0	c.1363C>T: p.P455S^b^	23.4	4.76	97.16	D	D
SLC6A3	P042^a^	Not maternal	0	c.1199C>T: p.T400M	18.55	4.69	11.78	D	D
	P082^a^	Paternal	0	c.253C>T: p.R85W	32	1.46	8.54	D	D
SREK1	P436^a^	De novo	4.09E−06	c.169C>T: p.P57S^b^	18.21	5.61	7.20	P	D
	10894	Not maternal	8.12E−06	c.937C>T: p.R313C	24.3	5.40	91.68	D	D
TENM1	P026^a^	Paternal	0	c.6118G>C: p.D2040H^b^	23.6	5.44	14.87	D	D
	10397	Not maternal	0	c.6584G>A: p.R2195Q	25.4	5.52	88.08	D	D
UBXN7	P067^a^	De novo	0	c.310G>A: p.A104T	20.1	4.47	62.04	P	D
ZMYM3	11451	Not maternal	0	c.857G>A: p.R286H	34	4.90	8.38	B	P

See Supplementary Table 7 for full version of this table including clinical information

*A* automatically annotated disease causing, *CADD Phred* combined annotation dependent depletion scaled score, *D* damaging(PolyPhen2)/disease causing (MutationTaster), *GERP++* genomic evolutionary rate profiling, *gnomAD* genome aggregation database frequency, *MTR centile* missense tolerance Ratio percentile, *Mut Taster* Mutation Taster prediction, *PolyPhen2 HVAR* polymorphism phenotyping v2 human

*indicates translation termination codon

^a^Case reported in ref. ^15^

^b^Variant reported in ref. ^15^

A further five individuals were naïve cases in whom we identified a pathogenic or likely pathogenic genetic variant in a known disease gene using the HaloPlex gene panel. Firstly, a pathogenic *COL4A1* variant (NM_001845.5: c.2494G>A: p.G832R) was identified in P204, a male with choreoathetoid quadriplegia, cystic porencephaly and epileptic encephalopathy. Two individuals, P174 and P781, had known pathogenic variants in *KIF1A* (NM_004321.7: c.296C>T: p.T99M and NM_004321.7: c.946C>T: p.R316W, respectively). P174 had spastic quadriplegic CP with partial dysgenesis of the corpus callosum, global developmental delay, epilepsy, scoliosis in the lumbar region and optic atrophy resulting in cortical blindness. The KIF1A p.T99M variant identified in P174 is a recurrent de novo variant previously reported in a number of individuals with thin corpus callosum, cerebellar atrophy and a progressive clinical course involving microcephaly, severe global developmental delay, ID, cortical visual impairment, hypotonia, hyperreflexia and variable features including optic atrophy, spastic paraparesis and seizures.^26–28^ P781 had mixed spastic/dystonic diplegic CP with intermittent hypertonicity, microcephaly, developmental delay and cortical visual impairment. Individuals with the KIF1A p.R316W variant identified in P781 have also previously been reported.^26,28^ A homozygous pathogenic stop-gain mutation in *NT5C2*, NM_001134373.2: c.115C>T: p.R39*, was identified in a female, P718, who had spastic diplegia, developmental delay, autism, attention deficit hyperactivity disorder (ADHD), visual problems and severe behavioural problems and was from a consanguineous family. Autosomal recessive mutations in *NT5C2* are the cause of spastic paraplegia type 45 and are associated with ID.^29^ Finally, a novel X-linked likely pathogenic variant was identified in *L1CAM* (NM_001143963.2: c.2137C>T: p.P713S), mutations in which cause CRASH syndrome (corpus callosum hypoplasia, retardation, aphasia, spastic paraplegia and hydrocephalus). The affected boy, P724, was born at term with antenatally diagnosed gross ventriculomegaly, absent corpus callosum and thinning of the cortical mantle. He presented with spastic diplegia, spastic paraparesis and macrocephaly. One male cousin was also reported with CP and three maternal aunts were confirmed carriers of the mutation. A further nine naïve cases harboured variants of uncertain significance in a known disease gene, and two cases harboured variants that were likely benign (Table 1, Supplementary Table 6). Therefore, overall 14/271 (5.2%) of naïve cases harboured a variant of potential clinical significance in a known disease gene, with five of these classified as either pathogenic or likely pathogenic.

Twenty-six individuals (Table 2, Supplementary Table 7) harboured variants in candidate genes, which are predicted to be intolerant to variation (Supplementary Table 1), thirteen of whom were naïve cases in whom no other candidate variant was identified. Therefore, excluding cases that we have previously investigated using WES, 27/271 (10%) of individuals in this cohort were found to harbour a variant of potential clinical relevance in either a known disease gene, or a variation intolerant candidate CP gene.

Two unrelated individuals were identified with a novel variant of uncertain clinical significance in *MAOB* (NM_000898.5: c.980C>T: p.T327M, Table 2). The first of these individuals, P216, had X-linked inheritance of the variant from his unaffected mother. He was born at term with seizures as a neonate and later developed childhood epilepsy, spastic quadriplegia, developmental delay, kyphoscoliosis, visual problems and progressive muscle weakening. The second, P915, was a female monozygotic twin born at 35 weeks reported with spastic diplegia, epilepsy and visual problems while her twin was developmentally normal despite also carrying the *MAOB* variant. The variant was confirmed not to be maternally inherited; however, the father was unavailable for testing. We examined the ratio of X-chromosome inactivation in blood-derived DNA for each twin and found no evidence for non-random inactivation as an explanation for clinical discordance; however, we cannot rule out tissue-specific variation in X-inactivation ratios in this twin pair.

In order to determine the contribution of rare genetic variation to CP aetiology, we assessed the frequency of rare genetic variation in candidate genes in our new CP cohort of 271 cases compared to 503 controls (all 503 individuals in the EUR cohort of the 1000 Genomes Project) using Significance of Rare VAriants (SORVA). SORVA analysis ranks genes based on their mutation burden in a cohort of interest compared to controls, with the assumption that fewer control individuals will carry rare, protein-altering or loss-of-function variants in genes associated with the disorder of interest. Therefore, while this analysis does not directly assess pathogenicity of a variant, it provides a tool for prioritising variants by quantifying the significance of seeing a variant within a particular gene. We identified four genes for which the burden of rare variants passed the threshold for genome-wide significance (Table 3, Bonferroni corrected *p*-value < 0.05, Supplementary Table 8). These genes are *AGAP1* (corrected *p* = 3.33E−04), *ERLIN1* (corrected *p* = 4.03E−03), *ZDHHC9* (corrected *p* = 4.03E−03) and *PROC* (corrected *p* = 4.59E−02). While supporting a role for rare genetic variation in these genes in CP causation, we note that the small available sample sizes for both cases and control in this analysis, as well as the differences in sequencing platform and coverage between the HaloPlex gene panel and 1000 genomes data, are potential confounders and therefore further validation is required.

**Table 3:** Genes with a statistically significant burden of rare genetic variants in 271 cerebral palsy cases

Gene	MAF < 0.001 in 271 cases	Number confirmed de novo	Frequency of variants with MAF < 0.001 in EUR	Genome-wide significance (Bonferroni corrected)	Number of likely pathogenic/VUS (this study, and ref. ^15^)	pLI (gnomAD v2.1)	% HI	%ExAC v2 RVIS	Constraint metric (*Z*-score)- missense (gnomAD v2.1)
AGAP1	8	1	0.006	3.33 × 10^−4^	4 (2 de novo)	1.00	17.79	−1.14 (10.60%)	1.41
ERLIN1	3	0	0.0002	4.03 × 10^−3^	2	0.18	19.99	−0.06 (46.69%)	1.89
ZDHHC9	3	0	0.0002	4.03 × 10^−3^	2 (none hemizygous)	0.74	13.32	−0.88 (14.42%)	2.64
PROC	5	0	0.002	4.59 × 10^−2^	4	0	66.64	−0.76 (19.23%)	0.96
KIF1A	9	1	0.03	0.075	3 (1 de novo)	1.00	50.61	−2.71 (1.35%)	5.41
SCN8A	4	1	0.02	0.13	1 (de novo)	1.0	5.52	−3.46 (0.66%)	7.94
TENM1	11	0	0.001	0.15	1	1.00	1.32	−1.52 (4.70%)	3.63
MYO1F	11	0	0.0001	0.15	4 (1 compound heterozygous individual)	0.16	45.46	0.49 (71.22%)	2.93
ENPP4	5	0	0.004	0.90	2	0.41	44.16	0.25 (61.40%)	0.44

Genome-wide significance was calculated using Significance of Rare VAriants (SORVA),^61^ using MAF < 0.001 in EUR population of the 1000 genomes project data

*pLI* probability of loss of function intolerance, *%HI* haploinsufficiency rank, *MAF* minor allele frequency, *RVIS* residual variance intolerance score, *VUS* variant of uncertain significance, *gnomAD* genome aggregation database

**Fig. 1:**
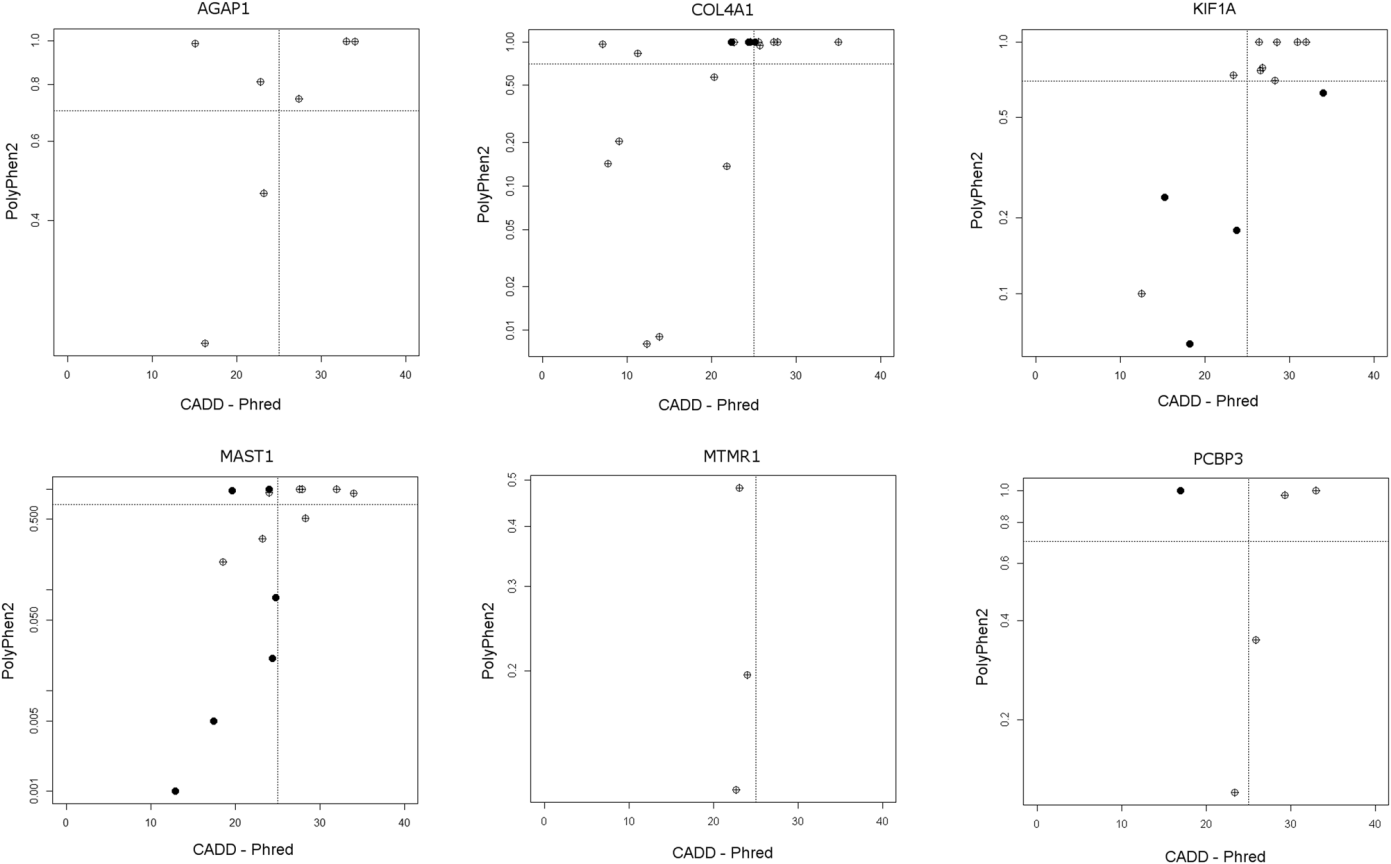
Scatterplots of CADD Phred vs PolyPhen2 scores for variants in genes with an overrepresentation of pathogenic variants in cerebral palsy cases compared to 1000 genomes controls. Controls—filled dots, CP cases—crossed dots

To assess the pathogenicity of rare variants identified in CP cases, we used the same criteria to examine regions covered in our HaloPlex gene panel and annotated them for the 1000 genomes control data. We found no significant difference in the distribution of CADD, SIFT or PolyPhen2 scores for rare variants in our CP cohort compared to controls (Supplementary Fig. 4, Supplementary Fig. 5), suggesting no overall increased burden of deleterious variants. We next looked at the number of rare, likely deleterious variants detected in each gene covered by the gene panel (using criteria ExAC < 0.0001, 1000 g < 0.001, CADD Phred > 20). Six genes were identified with a significant overabundance of rare, likely deleterious variants in CP cases compared to controls: *AGAP1* (*p* < 0.0001), *COL4A1* (*p* = 0.0011), *KIF1A* (*p* < 0.0001), *MAST1* (*p* = 0.0044), *MTMR1* (*p* < 0.0001) and *PCBP3* (*p* < 0.0001) (Fig. 1). Using the machine learning tool DOMINO,^30^ we assessed the likelihood of each of these genes being associated with a dominant genetic disorder. Of the 5/6 genes that are located on an autosome, three of these genes are predicted to be dominant (*KIF1A*, *COL4A1* and *MAST1*), while *AGAP1* is predicted either dominant or recessive.

**Fig. 2:**
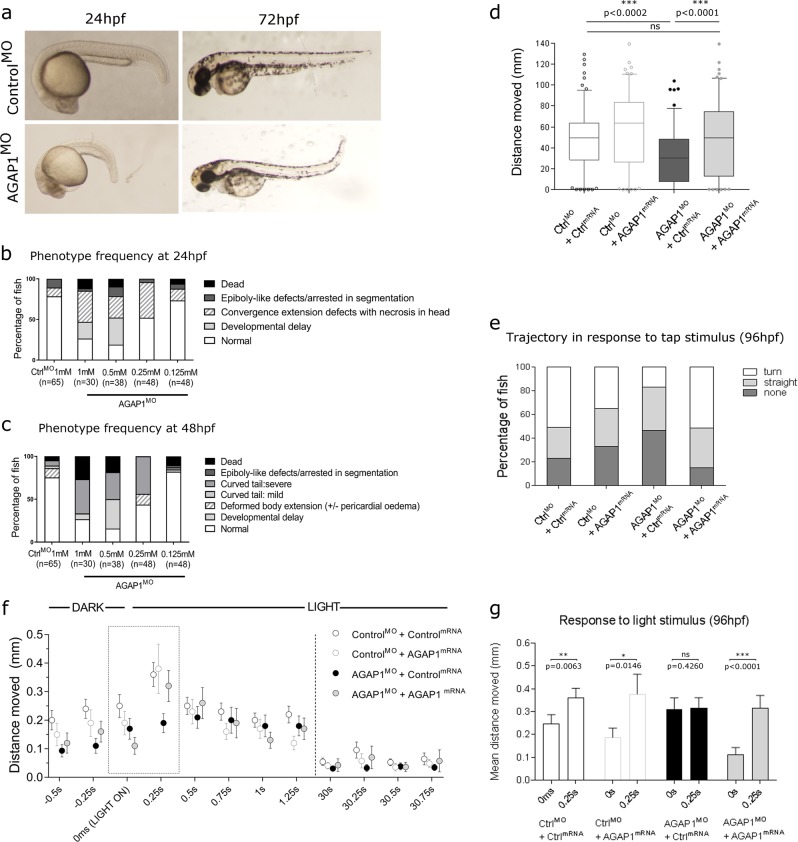
AGAP1 morphant zebrafish show gross developmental defects, neurological deficits and reduced motility. **a** Representative images of developmental phenotypes observed at 24 hpf and 72 hpf in AGAP1 morphant zebrafish larvae. At 24 hpf, we frequently observed gross developmental delay, including reduced pigment, with some larvae exhibiting necrosis in the head. At 72 hpf, AGAP1 morphant larvae frequently showed milder developmental delay and a curved tail. **b**, **c** Phenotype frequencies observed in morphant larvae at 24 and 48 hpf, respectively. AGAP1 morpholino was injected in a concentration range to assess dosage dependence of the phenotypes in morphant larvae. **d**–**g** AGAP1 morphant larvae show decreased activity and reduced escape response to stimuli at 96 hpf, which is partially rescued by co-injection of human *AGAP1* mRNA. CTRL^MO^ + CTRL^mRNA^; *n* = 48, CTRL^MO^ + AGAP1^MO^; *n* = 42, AGAP1^MO^ + CTRL^mRNA^; *n* = 40, AGAP1^MO^ + AGAP1^mRNA^; *n* = 24. **d** AGAP1 morphant larvae (dark grey) show reduced activity compared to controls during 1 min in the dark (*p* = 0.0002). Larvae co-injected with human *AGAP1* mRNA and AGAP1 morpholino (light grey, rescue) show significantly greater activity than AGAP1 morphants (*p* < 0.0001). There is no significant difference in activity between AGAP1 morphants co-injected with human *AGAP1* mRNA (light grey, rescue) and either control. Complete statistics can be found in Supplementary Table 9. **e** AGAP1 morphant larvae display both a reduced escape response and an altered trajectory in the 250 ms following tap stimulation compared to larvae co-injected with Control morpholino and either Control mRNA or human *AGAP1* mRNA. Larvae co-injected with AGAP1 morpholino and human *AGAP1* mRNA show an increased reaction compared to both controls. Complete statistics can be found in Supplementary Table 10. ***p < 0.001; ns, not significant. **f** Activity of larvae measured over a time course in darkness and then following the light in the apparatus being switched on. Error bars are s.e.m. **g** Response to light measured over 250 ms following the light stimulus. All groups except AGAP1 morphant larvae display a characteristic spike in activity immediately following the light stimulus. AGAP1 morphant larvae (black bars) show no significant increase in activity in response to light (*p* = 0.426). Error bars are s.e.m. *p < 0.05, **p < 0.01, ***p < 0.001; ns, not significant

Based on the significant burden of rare variants in *AGAP1* to CP in this cohort, the overabundance of predicted pathogenic variants in CP cases compared to controls and the identification of two de novo *AGAP1* variants in CP to date (this study and ref. ^15^), we investigated the functional consequences of knocking down *AGAP1* expression in a zebrafish model. AGAP1 is a regulator of endosomal trafficking and AGAP1 expression levels have been shown to inversely modulate dendritic spine density in hippocampal neuron cultures, suggesting that AGAP1 activity is highly regulated to ensure correct development and maturation of dendritic spines during development.^31^ We knocked down expression of *AGAP1* in zebrafish larvae and assessed gross morphology at 24 hour post fertilisation (hpf), 48 hpf and 72 hpf and motility and responses to light and tapping stimuli at 96 hpf. Morphant zebrafish larvae showed a range of dosage-dependent, early developmental phenotypes, including generalised developmental delay and convergence extension defects with necrotic tissue in the brain at 24 hpf (Fig. 2a, b). By 48 hpf, fish frequently exhibited a curved tail phenotype of varying severity (Fig. 2a, c), with some fish showing tail rigidity when stimulated by touch (Supplementary Movie 1). *AGAP1* morphant fish showed a decrease in total activity compared to control larvae at 96 hpf, a phenomenon which was rescued by co-injection of human AGAP1 mRNA (Fig. 2d), supporting a functional overlap between the human and zebrafish orthologues. In addition, there was both a qualitative and quantitative difference in the startle/escape response of morphant fish at 96 hpf (Fig. 2e). Fewer AGAP1 morphant fish reacted to a tapping stimulus, which should elicit a characteristic escape response. Of those that showed any response, fewer fish responded with the fast, large angle burst (C-turn), which is typical of zebrafish larvae when threatened.^32^ Additionally, in response to a light flash, morphant fish show a dampened and delayed escape response, where all other groups react with a statistically significant burst of activity (Fig. 2f, g). Together these data support a neurological deficit in *AGAP1* morphant fish, in addition to motility defects.

## Discussion

In this study, we sought to demonstrate the utility of a targeted gene panel for genetic diagnosis of individuals with CP. Gene panels have lower computational and economic costs, better sequence coverage of specified targets and the potential for higher confidence discovery of somatic mosaicism compared to WES.^33^ They allow the relatively rapid assessment of larger cohorts of individuals for recurrent variance, thereby providing support for further investment in functional validation of specific genes or variants. For genetically heterogeneous disorders, this is becoming increasingly important as the pace of variant discovery using next-generation sequencing technologies has far outstripped the pace of functional genomics. Follow-up validation studies, for example Sanger sequencing validation of variants and familial segregation analysis, to confirm that variants are real and inherited in a pattern consistent with the affected status in the family, as well as functional validation of variants using animal or cell models, remain major bottlenecks in the analysis of large cohorts of patients.

Since relatively few genes from a limited number of studies have so far been associated with CP, a definitive gene list is not yet possible for CP. The gene list used in this study was derived from published known and candidate CP genes from our studies and those of others;^8–11,15,25,34–40^ however, this panel has flexibility to be regularly updated with novel published CP genes. Since the design of this gene panel, pathogenic variants in a number of other genes have been identified in individuals with CP and future designs should include these candidates; for example, *CTNNB1*,^18,41^ *PDCD6IP*,^42^ *AMPD2*,^18^ *ITPR1, KCNC3* and *SPTBN2*.^17^

Excluding cases previously investigated using WES, we identified variants of potential clinical significance in known disease genes in 14/271 (5.2%) of new cases. These genes were *KIF1A* (two cases with pathogenic variants, one confirmed de novo), *L1CAM* (X-linked likely pathogenic variant), *MAST1* (two cases with variants of uncertain significance), *HUWE1* (two cases with variants of uncertain significance)*, BRWD3* (one case with variant of uncertain significance)*, COL4A1* (three cases, one with a pathogenic variant, two with variants of uncertain significance), *SYNGAP1* (one case with a variant of uncertain significance) and *NT5C2* (homozygous pathogenic variant, identical by descent). We also identified a de novo variant in *SCN8A*, which was shared by a pair of clinically discordant monozygotic twins. We have classified this as a variant of uncertain significance based on the known broad clinical spectrum and expressivity of *SCN8A* mutations.^43,44^

The identification of variants in genes known to cause other neurodevelopmental problems in individuals with CP is not surprising given the frequency of comorbidity of these developmental disabilities; however, this should not change the original CP diagnosis, which is based on defined clinical inclusion and exclusion criteria and not genetic criteria.^45^ There are three likely contributing and non-exclusive explanations for the high frequency of variation in known disease genes in CP cohorts: firstly, many of the genes associated with neurodevelopmental disorders are likely to have as yet incompletely reported phenotypic spectrums; secondly the observed clinical phenotypes may represent blended phenotypes resulting from multiple genetic hits; and thirdly, there is ascertainment bias inherent in genetic investigations of neurodevelopmental disorders dependent on the specific criteria on which recruitment was based due to the frequent co-occurrence of multiple neurodevelopmental phenotypes. Rather than suggesting incorrect inclusion of an individual in a CP cohort, we argue that these findings further highlight the utility of genomics for accurate diagnosis in individuals with CP.

In the total cohort of 366 cases, three individuals with variants of uncertain significance in *MAOB* were identified, including a female, P915, with a clinically discordant monozygotic twin. MAOB is a monoamine oxidase, which catalyses the oxidative deamination of neuroactive and vasoactive amines, as well as the oxidation of several xenobiotics. It is a highly environmentally responsive gene and has been shown to be differentially methylated in response to some environmental stressors, for example smoking;^46^ therefore, localised changes to methylation caused by environmental differences between the twins could explain the clinical discordance. Of note, there was reported twin-to-twin transfusion syndrome (TTTS) during pregnancy, with P915 being plethoric and polycythaemic after birth and requiring phototherapy, while her twin had no reported neurological sequelae. A second discordant monozygotic twin pair included in this study, P904 and his twin, were found to both carry a de novo rare variant of uncertain significance in *SCN8A* (*SCN8A* p.A1575V), with TTTS also detected in their mother’s pregnancy. Monozygotic twins, particularly clinically discordant twin pairs, offer a unique and powerful tool to examine the relative contributions of genetics and environment to CP aetiology. Intrauterine environmental differences, such as those resulting from TTTS and placenta sharing, or postzygotic genetic variation likely account for the extreme differences in clinical outcome and warrant closer examination.

One gene, *AGAP1*, showed both a statistically significant burden of rare variants and an overabundance of rare, likely deleterious variants, including two de novo variants. AGAP1 is a phosphoinositide-dependent Arf GAP that affects the actin cytoskeleton, as well as localising to endosomes where it alters stress fibres,^47^ and is therefore thought to link endocytic traffic to the actin cytoskeleton. Both overexpression and downregulation of *AGAP1* have been shown to affect neuronal endosomal trafficking and dendritic spine morphology in mouse primary neurons.^31^ Rare variants in *AGAP1* have also been previously implicated in autism^48^ and suggested as a risk factor for schizophrenia.^31^ We demonstrated that *AGAP1* morphant zebrafish have reduced startle and escape responses, reduced motility and developmental delay, further supporting a role for *AGAP1* in neurodevelopment.

We have previously reported recurrent genetic variance in *TUBA1A* and *L1CAM* in an unselected cohort of CP cases.^15^ We identified one additional likely pathogenic X-linked *L1CAM* variant in this cohort, confirming its status as a recurrent CP gene; however, the HaloPlex gene panel achieved poor coverage of *TUBA1A* (Supplementary Table 2), and therefore we were unable to properly assess recurrence. In addition, the relatively low number of complete parent–child trios (45/271 for naïve cases) available for confirmation of variant segregation in this study made interpretation of many genetic variants difficult; therefore, we have likely underestimated the true recurrence of causative genetic variation in genes tested in this gene panel.

We identified a large number of variants of uncertain significance (VUS), including single hit rare or novel likely deleterious variants in genes that have been associated with autosomal recessive disorders (Supplementary Table 1), and rare or novel variants inherited from an unaffected parent that are associated with a dominant genetic disorder. A further class was unable to be classified due to unavailability of parental samples for segregation analysis. The interpretation of VUS in CP is made significantly more complex by both the high level of clinical heterogeneity amongst affected individuals and the likely interplay of environmental and genetic factors. While selecting likely monogenic cases of CP with mutations in known disease genes and a clearly consistent clinical picture is relatively straightforward, the majority of individuals with CP will likely not fall into this category. It has not escaped our notice that a number of individuals in whom we have identified potentially causative genetic variation have additional risk factors or known precipitants for CP, for example premature birth, intrauterine growth restriction or intraventricular haemorrhage. In some cases, genetic variation may be the cause of or a contributor to these precipitants: for example, individuals with *COL4A1* mutations have high susceptibility to intracerebral haemorrhage. The additive effects of a less damaging mutation coupled with an environmental insult may be responsible in other cases. Larger cohort studies will also be required to address the contribution of common genetic variation, in combination with environmental factors, to CP burden. Additional factors such as variable penetrance and clinical variability of mutations in some genes, as well as the potential for polygenic aetiology in some cases, will also need to be addressed.

Excluding cases previously investigated using WES, 27/271 (10%) of individuals in this cohort were found to harbour a variant of possible clinical relevance in a known disease gene or variation intolerant candidate gene (Tables 1 and 2). Together with our previous study,^15^ these data highlight several genes as key CP genes, which should be considered as part of a genetic diagnosis for individuals with CP. In our aggregate cohort of 489 clinically unselected individuals with CP (this study and ref. ^15^), six individuals with variants of potential clinical significance have been identified in *COL4A1*, three individuals with variants in each of *L1CAM, KIF1A, MAOB* and *AGAP1*, and two individuals with *TUBA1A* variants, therefore these six genes alone may contribute at least 4% of disease burden to CP. Additional functional studies are required to confirm the disease association of the novel candidate genes *MAOB* and *AGAP1*. Genetic diagnoses have important implications both for clinical management of the individual patient and family planning. Our data demonstrate potential clinical utility of genetic testing for at least 5.2% of individuals with CP.

## Methods

### Study samples

All study samples were obtained from the DNA Biobank of the Australian Collaborative Cerebral Palsy Research Group and written informed consent was given, either by the participant or their guardian, for the use of their sample. This study was approved by the Women’s and Children’s Health Network (WCHN) Human Research Ethics Committee (reference number: HREC/15/WCH/148). Each case was confirmed to fit the published inclusion criteria for CP^49,50^ by a paediatric rehabilitation specialist at the time of recruitment to the Biobank or to the state-based CP registers. Cases were otherwise clinically unselected. Clinical information was obtained by completion of a questionnaire by the participant or their guardian and clinical review by a treating clinician. Where clinical review was not possible, or where discrepancies between the questionnaire and the clinical review were identified, patient case notes were reviewed.

### HaloPlex gene panel design

The CP gene panel was custom-designed using the HaloPlex Design Wizard. The total target region size is 388,046 bp, with 384,858 bp of target bases analysable (99.44% target coverage). It includes 19,989 amplicons covering a total of 112 candidate genes (see Supplementary Table 1).

### DNA extraction

Genomic DNA was either extracted from patient-derived lymphoblastoid cell lines (LCLs) at Genetic Repositories Australia (GRA, Sydney, Australia), extracted from blood samples at the Australian Genomics Research Facility (AGRF), or extracted from buccal samples as previously described.^51^ DNA integrity and quantity were verified by gel electrophoresis and Qubit dsDNA assay (Life Technologies).

### HaloPlex protocol

HaloPlex panel library preps were performed according to manufacturer’s instructions. Briefly, genomic DNA from patient derived LCLs or buccal samples (225 ng of gDNA) was digested with 16 different restriction enzymes at 37 °C for 30 min to create a library of gDNA restriction fragments. Following digestion, restriction fragment libraries were analysed using the 2100 Bioanalyzer (Agilent Technologies Inc., Santa Clara, CA, USA) to confirm appropriate digestion. Fragments were then selectively hybridised for 3 h to biotinylated HaloPlex probes (Agilent Technologies Inc., Santa Clara, CA, USA) from the custom-designed CP gene panel in the presence of HaloPlex indexes and sequencing motifs. Circularised target DNA-HaloPlex probe hybrids containing biotin were then captured by HaloPlex Magnetic Beads on the Agencourt SPRIPlate Super magnet magnetic plate. DNA ligase was added to close the nicks in the hybrids, and freshly prepared 50 mM NaOH was used to elute the captured target libraries. The target libraries were amplified (98 °C 2 min, 18 cycles of 98 °C 30 s, 60 °C 30 s, 72 °C 1 min, then 72 °C for 10 min) and purified using AxyPrep Magnetic beads (Axygen). Amplicons ranging from 175 to 625 bp were then quantified using an Agilent BioAnalyzer High Sensitivity DNA Assay kit on the 2100 Bioanalyzer to validate the enrichment of the libraries prior to pooling for multiplexed sequencing. Sequencing was performed at the ACRF Cancer Genomics Facility (Adelaide, Australia) either on the Illumina HiSeq 2500 platform with 100 bp paired end reads or the Illumina NextSeq 500 with 150 bp paired end reads.

### HaloPlex sequence analysis

Trimming (to remove low-quality bases from the ends of reads, remove adaptor sequences and mask enzyme footprints), alignment to the genome and variant calling were performed using the standard settings for HaloPlex in the SureCall software (V3.0.1.4, Agilent). Samples were considered failed and removed from analysis if <50% of targeted bases were covered by at least 20 reads. Variant call format files were then exported for annotation by ANNOVAR.^52^

### Variant filtering and prioritisation

Variants were initially hard filtered based on population frequency (using ExAC frequency < 0.0001 and 1000 genomes frequency < 0.001), evolutionary conservation of the variant residue (using Genomic Evolutionary Rate Profiling (GERP) score > 4)^53^ and predicted functional effect (using Combined Annotation Dependent Depletion (CADD) Phred score > 20 where available).^54^ Clinically reportable variants: Following hard filtering of variants, we applied ACMG guidelines to identify variants that would be considered pathogenic or likely pathogenic in a diagnostic laboratory. Variants that fit these criteria are denoted by ACMG classifications “likely pathogenic” or “pathogenic” in Table 1. Research variants: We also considered a further category of variants that did not meet criteria to be classified as “pathogenic” or “likely pathogenic” by ACMG guidelines, but are candidate novel CP variants of research interest. For this purpose, variants meeting the initial hard filtering thresholds were further prioritised for validation by predicted functional effect using the following criteria: Missense Tolerance Ratio < 25th percentile,^55^ PolyPhen2 prediction of probably damaging or possibly damaging^56^ and MutationTaster prediction of disease causing.^57^ A further group of hard filtered variants which met 2/3 of the prioritisation criteria were validated based on additional support, e.g. predicted genic intolerance to variation or available clinical data. Candidate genes were considered intolerant to variation if three or more of the following criteria were met: DOMINO probability of being autosomal dominant > 0.5,^30^ Haploinsufficiency index percentile < 25th,^58^ ExAC v2 RVIS < 25%,^59^ pLI > 0.9 and Constraint metric (*Z-*score Missense) > 2.^60^

### Statistical significance of rare missense variants

In order to test the significance of finding a particular number of rare missense variants in any gene in our targeted CP gene panel, we used SORVA.^61^ Of the 271 new cases included in this study, 209/271 (77%) reported both parents EUR, with a further 26/271 (9.6%) reporting one parent EUR (for 24/26 of these, the other parent was either not reported or unknown). A further 24/271 (8.9%) did not report ethnicity. A small number of individuals reported East Asian (4/271, 1.5%), South Asian (3/271, 1.1%), Australian Aboriginal/Torres Strait Islander (1/271, 0.4%) or Polynesian (2/271, 0.7%) ethnic background. We therefore selected the EUR population from the 1000 genomes project as the most appropriate for statistical analysis of the frequency of rare missense variants in our dataset. After calculating the observed number of rare mutations in the 503 individuals in the EUR population for each gene in our panel (parameters set to: protein consequence missense or LOF, population EUR, MAF < 0.001, Binarity Binary and Zygosity Both), we then calculated the significance of detecting the number of rare variants identified in the 271 individuals sequenced in this study for each gene (Bonferroni corrected for the number of genes sequenced). Bonferroni corrected *p-*values < 0.05 were considered to reach genome-wide significance.

To identify genes with a significant overabundance of rare, likely deleterious variants in CP cases, we extracted variant data for regions included in the HaloPlex gene panel design for 403 samples from the 1000 genomes EUR dataset. We then annotated variants in these regions with ANNOVAR. Variants for CP cases and 1000 genome controls were then filtered for variant frequency (using ExAC frequency < 0.0001 and 1000 genomes frequency < 0.001) and CADD score > 20. The number of variants meeting these cut-offs for 1000 genome controls was considered the expected count of pathogenic variants. A binomial test (*α* = 0.05, two-tailed test) was performed (GraphPad Prism) comparing the frequency of CP cases harbouring a pathogenic variant (observed) to the frequency of control individuals harbouring a pathogenic variant (expected).

### Variant validation

All reported variants were validated by Sanger sequencing using BigDye terminator chemistry 3.1 (ABI) and analysed using a 3730xl genetic analyzer (Applied Biosystems, Foster City, CA, USA). Sequencing data were analysed using DNASTAR Lasergene 10 Seqman Pro8 (DNASTAR, Inc., Madison, WI, USA). Where possible, DNA from blood or buccal samples was used for validation, and segregation in patient–parent trios was performed to confirm the inheritance pattern of the variants. For the 271 new cases described in this study, 55 had no parental DNA available, 171 had DNA available for one parent and 45 were complete parent–child trios.

### Zebrafish husbandry

All experiments using zebrafish were conducted under the auspices of the Animal Ethics Committee of the University of Adelaide (project numbers S-2017-073 and S-2017-089). *Danio rerio* were bred and maintained at 28.5 °C on a 14-h light/10-h dark cycle. Embryos were collected from natural mating of the Tübingen strain (Tu), grown in E3 medium,^62^ and staged.

### Zebrafish morpholino and mRNA injections

Morpholinos were synthesised by Gene Tools LLC (Corvallis, OR, USA). Stock solutions of morpholinos were made up in sterile water for further dilution to working concentration immediately prior to injection.

Sequences for morpholinos are:

5′ TCGCCAGGTGCTGCTGATAATTCAT 3′ AGAP1 translation blocker;

5′ CCTCTTACCTCAGTTACAATTTATA 3′ standard control.

The human AGAP1 ORF clone for mRNA production was engineered by PCR from a cDNA clone (Dharmacon, Clone ID 9021724, Accession BC140856) with primers designed to add *EcoR*I and *Not*I restriction enzyme sites to the 5′ and 3′ ends of the ORF, respectively (*EcoR*I*-AGAP1 ORF* and *Not*I*-AGAP1 ORF)*. Following PCR and restriction digest with *EcoR*I and *Not*I, AGAP1 ORF was subcloned into the pcGlobin zebrafish expression vector.^63^ The control mRNA construct was generated by PCR amplifying the ORF of TUBA1A from a cDNA clone (Dharmacon, Clone ID 6050536) with primers incorporating a 5′ *BamH*I and a TAA stop codon 4 amino acids into the encoded protein, and directly ligating the product into pGEM-T Easy vector (Promega) (*TUBA1A-*TAA Fw and Rv).

The resultant pGEM-T clone was then digested with *BamH*I and *Not*I restriction enzymes and the TUBA1A-TAA insert was ligated into the pcGlobin zebrafish expression vector.

The pcGlobin-AGAP1 and pcGlobin-TUBA1A-TAA clones were then used for generating capped mRNA with the mMessage mMachine T7 Ultra kit (Ambion Inc., Austin, TX, USA) as per manufacturer’s instructions. Capped mRNAs were precipitated with LiCl and then redissolved in water for injection.

Fertilised zebrafish eggs were rinsed in E3 embryo medium and injected before cleavage. The maximum concentration of experimental morpholino injected was 1 mM (24 ng of morpholino). For rescue experiments, Control and AGAP1 morpholinos were injected at 250 µM concentration (6 ng of total morpholino) and messenger RNAs were injected at 100 ng/µl (0.3 ng of total RNA). Morpholinos were co-injected with mRNA before first cleavage. Embryos which were not fertilised were discarded at 6 hpf.

### Zebrafish DanioVision assays

Larval activity was assayed at 4 days post fertilisation (dpf) using the DanioVision Observation Chamber (Noldus), which was fitted with a Basler GenICam camera, independent light source, temperature control unit (set to 28.5 °C) and tapping device. Individual embryos were placed in each well of a 96-well tray in pre-warmed embryo medium, then placed in a dark incubator set to 28.5 °C to acclimatise for at least 30 min before behavioural testing. The settings used for tracking experiments were defined using EthoVisionXT software (Version 11.5). Briefly, zebrafish were in darkness for 5 min, then subjected to two maximum intensity taps 10 s apart. After a delay of 1 min, the lights were switched on and zebrafish were assayed in light at 50% intensity for a further 3 min before the trial ended. Trials were run in triplicate and wells where tracking failed were excluded from analysis.

### Imaging

Embryos were examined at 24 hpf and any dead embryos were removed. Larvae were examined at 4 dpf and scored for morphological abnormalities. Larvae were imaged after activity assays using a Nikon SMZ1000 dissecting microscope with a Leica DFC450 C camera and Leica Application Suite software.

### Statistical analysis of zebrafish activity

Box-and-whisker plots of distance moved were generated using the standard method to calculate 5th and 95th percentiles, with data falling outside this range plotted as individual data points. Differences in distance moved during 1 min in darkness were tested using the Kruskal–Wallis test to account for unequal variance, with corrections for multiple comparisons made using Dunn’s method. Multiplicity adjusted *p*-values are reported. Differences in trajectory were tested using *χ*^2^ tests to compare the observed versus expected distribution of categorical data. Differences in activity before and after lights came on were tested for each treatment group using a paired *t*-test. GraphPad Prism was used to generate figures and perform statistical analyses.

### X-chromosome inactivation analysis

X-chromosome inactivation was tested by DNA digestion with the methylation sensitive restriction enzyme *Hpa*II, and PCR amplification of digested and undigested samples at the highly polymorphic FRAXA and AR loci. Briefly, genomic DNA was digested for 24 h at 37 °C before the *Hpa*II enzyme was heat inactivated at 65 °C. PCRs were then performed with Hex fluorescently labelled FRAXA primers or FAM fluorescently labelled AR primers. Fragments from both digested and undigested samples were sized and quantified using an ABI 3100 genotyper to detect skewing.

### Primer sequences

EcoRI-AGAP1 ORF 5′ GAATTCATGAACTACCAGCAGCAGCTGGCCAAC 3′

NotI-AGAP1 ORF 5′ GCGGCCGCTCAGATGATGGTGGGCACCCTCCCA 3′

TUBA1A TAA Fw 5′ GGATCCAATGCGTGAGTAAATCTCCATCCA 3′

TUBA1A TAA Rv 5′ GAGCTCTTAGTATTCCTCTCCTTCTTCCTC 3′

FRAXA Fw 5′ GCTCAGCTCCGTTTCGGTTTCACTTCCGGT 3′

FRAXA Rv 5′ AGCCCCGCACTTCCACCACCAGCTCCTCCA 3′

AR Fw 5′ TCCAGAATCTGTTCCAGAGCGTGC 3′

AR Rv 5′ GCTGTGAAGGTTGCTGTTCCTCAT 3′

### Reporting summary

Further information on research design is available in the Nature Research Reporting Summary linked to this article.

## Supplementary information

Supplementary Information

Supplementary Movie 1

Reporting summary

## Data Availability

Additional data and materials from this study are available from the authors on reasonable request, subject to compliance with our obligations under human research ethics.
