# Supplementary material for: Targeted resequencing identifies genes with recurrent variation in cerebral palsy
Source: NPJ Genom Med. 2019 Nov 4;4:27. doi: 10.1038/s41525-019-0101-z (PMC6828700; doi:10.1038/s41525-019-0101-z)
Supplement: Supplementary file 1 — Supplementary Information [file 41525_2019_101_MOESM1_ESM.pdf]

Supplementary Table 1 Genes included in the HaloPlex gene panel

Gene	Associated human disorders	Gene Function (OMIM and REFSEQ)	DOMINO prediction	pLI (gnomAD v2.1)	% HI	%ExAC v2 RVIS	Constraint Metric (Z-score)-Missense (gnomAD v2.1)
**CP and CP-like disorders:**							
AP4B1	Spastic paraplegia 47, autosomal recessive, Cerebral Palsy Spastic Quadriplegic Type 5, autosomal recessive	The heterotetrameric adaptor protein (AP) complexes sort integral membrane proteins at various stages of the endocytic and secretory pathways. AP4 is composed of 2 large chains, beta-4 (AP4B1) and epsilon-4 (AP4E1), a medium chain, mu-4 (AP4M1), and a small chain, sigma-4 (AP4S1).	Very likely recessive (0.10)	0	41.20	-0.34 (34.10%)	0.17
AP4E1	Spastic paraplegia 51, autosomal recessive, Cerebral Palsy Spastic Quadriplegic Type 4, autosomal recessive	As above	Likely recessive (0.38)	0.01	43.26	-0.24 (38.37%)	0.58
AP4M1	Spastic paraplegia 50, autosomal recessive, Cerebral Palsy Spastic Quadriplegic Type 3, autosomal recessive	As above	Very likely recessive (0.18)	0	45.03	-0.15 (42.34%)	-1.2
AP4S1	Spastic paraplegia 52, autosomal recessive, Cerebral Palsy Spastic Quadriplegic Type 6, autosomal recessive	As above	Very likely recessive (0.09)	0	**19.10**	0.25 (61.26%)	-0.02
ADD3	Familial cerebral palsy	Belongs to a family of membrane skeletal proteins involved in the assembly of spectrin-actin network in erythrocytes and at sites of cell-cell contact in epithelial tissues. Expression of adducin beta is restricted to brain and hematopoietic tissues.	**Likely dominant (0.78)**	**1.00**	**11.41**	-0.25 (38.02%)	0.38
ATL1	Neuropathy, hereditary sensory, type ID, Spastic paraplegia 3A, autosomal dominant	Encodes atlastin-1, a dynamin-related GTPase, which plays a role in formation of the tubular endoplasmic reticulum (ER) network and in axon elongation in neurons.	**Very likely dominant (0.88)**	**0.99**	**14.74**	**-1.04 (12.24%)**	**2.81**
COL4A1	Tortuosity of retinal arteries, Hereditary Angiopathy with nephropathy, aneurysms, and muscle cramps, Brain small vessel disease with or without ocular anomalies, Porencephaly 1 (dominant negative mutations), susceptibility to intracerebral haemorrhage.	Encodes the major type IV alpha collagen chain of basement membranes.	**Very likely dominant (0.99)**	**1.00**	33.93	**-2.00 (3.16%)**	**3.23**

DDHD2	Spastic paraplegia 54, autosomal recessive, Complex Hereditary Spastic Paraplegia, autosomal recessive	Encodes and phospholipase that hydrolyzes sn-1 ester bonds of phospholipids producing 2-acyl-lysophospholipids and fatty acids. Prefers phosphatidic acid as substrate and has role in efficient membrane trafficking from the Golgi apparatus to the plasma membrane.	Very likely recessive (0.07)	0	38.24	-0.22 (39.41%)	1.18
ENTPD1	Spastic paraplegia 64, autosomal recessive	Mediates catabolism of extracellular nucleotides.	Very likely recessive (0.18)	0.01	74.32	-0.57 (25.19%)	1.04
GAD1	Spastic quadriplegic cerebral palsy 1, autosomal recessive	Encodes one of several forms of glutamic acid decarboxylase, identified as a major autoantigen in insulin-dependent diabetes. Responsible for catalyzing the production of gamma-aminobutyric acid from L-glutamic acid. Deficiency in this enzyme has been shown to lead to pyridoxine dependency with seizures.	**Very likely dominant (0.97)**	0.07	**1.87**	**-0.68 (21.56%)**	**2.48**
KANK1	Spastic quadriplegic cerebral palsy 2	Contributes to the regulation of actin polymerization and inhibits cell migration via regulation of RHOA and RAC1.	Likely recessive (0.39)	0	49.73	1.74 (95.52%)	-4.92
HPRT1	Lesch-Nyhan syndrome (mental retardation, spastic cerebral palsy, choreoathetosis, uric acid urinary stones, and self-destructive biting of fingers and lips), X-linked recessive	Encodes hypoxanthine phosphoribosyltransferase, which catalyzes conversion of hypoxanthine to inosine monophosphate and guanine to guanosine monophosphate via transfer of the 5-phosphoribosyl group from 5-phosphoribosyl 1-pyrophosphate.	**-**	**0.94**	**8.47**	-0.17 (41.22%)	**2.41**
KIF1A	Intellectual disability, autosomal dominant 9, Neuropathy, hereditary sensory, type IIC, Spastic paraplegia 30, autosomal recessive	Encodes a motor protein involved in the anterograde transport of synaptic-vesicle precursors along axons.	**Very likely dominant (0.91)**	**1.00**	50.61	**-2.71 (1.35%)**	**3.44**
KIF1C	Spastic ataxia 2, autosomal recessive	Required for the retrograde transport of Golgi vesicles to the endoplasmic reticulum. Tyrosine phosphorylation is a putative regulator of this transport.	Likely recessive (0.283)	0.75	44.84	-0.25 (37.71%)	1.52
NT5C2	Spastic paraplegia 45, autosomal recessive, located within the Smith-Magenis syndrome region on chromosome 17.	Preferentially hydrolyzes inosine 5-prime-monophosphate (IMP) and other purine nucleotides, and is allosterically activated by various compounds, including ATP. May have a critical role in the maintenance of a constant composition of intracellular purine/pyrimidine nucleotides in cooperation with other nucleotidases.	Very likely recessive (0.07)	0	**7.85**	**-0.77 (18.92%)**	**2.75**

PLP1	X-linked Pelizaeus-Merzbacher disease, Leukodystrophy Hypomyelinating Type 1, Spastic paraplegia type 2, X-linked recessive	Transmembrane proteolipid protein which is the predominant myelin protein present in the central nervous system. Functions in myelination. May play a role in the compaction, stablization, and maintenance of myelin sheaths, as well as in oligodendrocyte development and axonal survival.	**-**	**0.93**	**1.64**	-0.39 (28.21%)	**2.09**
SPAST	Spastic paraplegia 4, autosomal dominant	Likely acts as a microtubule-severing protein. Mutant spastin colocalizes with, but does not sever, microtubules. Abnormal interaction of mutant spastin with microtubules was associated with abnormal cellular distribution of mitochondria and peroxisomes.	**Very likely dominant (0.90)**	**1.00**	**9.50**	-0.51 (27.05%)	1.36
TH	Segawa syndrome, autosomal recessive, Dopa-Responsive Dystonia, autosomal recessive	Involved in the conversion of phenylalanine to dopamine. As the rate-limiting enzyme in the synthesis of catecholamines, tyrosine hydroxylase has a key role in the physiology of adrenergic neurons.	Likely recessive (0.21)	0	**6.57**	0.31 (63.82%)	0.78
ZC4H2	Wieacker-Wolff syndrome, X-linked recessive, Arthrogryposis Multiplex Congenita and Intellectual Disability	Impaired alpha-motoneuron development in morphant zebrafish. The number of neuromuscular endplates was reduced in morphants, and endplates appeared disorganized. Transient overexpression of ZC4H2 in mouse primary hippocampal neurons increased the number of dendritic spines.	**-**	**0.91**	**20.36**	-0.07 (46.71%)	1.55
**Likely CP genes [1,2]:**							
AGAP1		Belongs to an ADP-ribosylation factor GTPase-activating (ARF-GAP) protein family involved in membrane trafficking and actin cytoskeleton dynamics.	Dominant or recessive (0.42)	**1.00**	**17.79**	**-1.14 (10.60%)**	1.41
CD99L2		Plays a role in a late step of leukocyte extravasation helping cells to overcome the endothelial basement membrane. Homophilic adhesion molecule, but these interactions may not be required for cell aggregation (by similarity).	-	0	82.41	0.82 (85.42%)	-0.02
KDM7A		Histone demethylase required for brain development. Specifically demethylates dimethylated Lys-9 and Lys-27 (H3K9me2 and H3K27me2, respectively) of histone H3 and monomethylated histone H4 Lys-20 residue	Likely recessive (0.28)	**1.00**	-	**-0.71 (20.78%)**	**2.62**

		(H4K20me1), thereby playing a central role in histone code.					
KDM5C	X-linked intellectual disability, syndromic, Claes-Jensen type, X-linked recessive	Encodes a specific H3K4me3 and H3K4me2 demethylase and acts as a transcriptional repressor through the RE-1-silencing transcription factor (REST) complex.	**-**	**1.00**	**11.71**	**-2.32 (1.57%)**	**5.1**
L1CAM	CRASH (corpus callosum hypoplasia, retardation, aphasia, spastic paraplegia and hydrocephalus), X-linked recessive, Hydrocephalus due to Stenosis of the Aqueduct of Sylvius,<br>Mental Retardation-Aphasia-Shuffling Gait-Adducted Thumbs Syndrome,<br>Partial Agenesis of the Corpus Callosum, Spastic Paraplegia X-Linked Type 1	Cell adhesion molecule which plays an important role in nervous system development, including neuronal migration and differentiation.	**-**	**1.00**	**15.58**	**-1.88 (2.66%)**	**2.76**
MAST1		Appears to link the dystrophin/utrophin network with microtubule filaments via the syntrophins (by similarity).	**Very likely dominant (0.85)**	**1.00**	44.29	**-2.71 (1.36%)**	**6.01**
NAA35		Auxillary component of the N-terminal acetyltransferase C (NatC) complex which catalyses acetylation of N-terminal methionine residues. Involved in regulation of apoptosis and proliferation of smooth muscle cells.	**Very likely dominant (0.90)**	**1.00**	**13.55**	-0.17 (41.27%)	**3.81**
PAK3	Non-syndromic X-linked intellectual disability, Agenesis of the Corpus Callosum (activating mutations)	PAK3 forms an activated complex with GTP-bound RAS-like (P21), CDC2 and RAC1 proteins which then catalyzes a variety of targets. PAK3 may be necessary for dendritic development and for the rapid cytoskeletal reorganization in dendritic spines associated with synaptic plasticity.	**-**	**0.99**	**5.06**	-0.38 (28.84%)	**3.65**
RFX2		Member of the regulatory factor X gene family, which encodes transcription factors that contain a highly-conserved winged helix DNA binding domain. Transcriptional activator that can bind DNA as a monomer or as a heterodimer with other RFX family members. This protein can bind to cis elements in the promoter of the IL-5 receptor alpha gene.	Likely recessive (0.21)	**1.00**	53.12	0.17 (57.48%)	**2.87**
SCN8A	Cognitive impairment with or without cerebellar ataxia,<br>Early infantile epileptic encephalopathy 13 (dominant negative mutations)	Encodes a member of the sodium channel alpha subunit gene family. Forms the ion pore region of the voltage-gated sodium channel and is essential for the rapid membrane depolarisation	**Very likely dominant (1.00)**	**1.00**	**5.52**	**-3.46 (0.66%)**	**7.94**

		that occurs during the formation of the action potential in excitable neurons.					
TENM1		Mediates a rapid reorganisation of actin- and tubulin-based cytoskeleton elements with an increase in dendritic arborisation and spine density formation of neurons in the hippocampus and amygdala. Induces BDNF transcription inhibition in neurons. Activates the mitogen-activated protein (MAP) kinase 2 (MEK2) and extracellular signal-regulated kinase (ERK) cascade. Acts also as a bioactive neuroprotective peptide on limbic neurons of the brain and regulates stress-induced behaviour.	-	**1.00**	**1.32**	**-1.52 (4.70%)**	**3.63**
TUBA1A	Lissencephaly 3, Intellectual disability	Alpha and beta tubulins represent the major components of microtubules, while gamma tubulin plays a critical role in the nucleation of microtubule assembly. Gene expression predominantly found in morphologically differentiated neurologic cells.	**Very likely dominant (1.00)**	**0.97**	**4.82**	0.09 (53.99%)	**5.77**
WIPI2		Early component of the autophagy machinery being involved in formation of pre-autophagosomal structures and their maturation into mature phagosomes in response to phosphatidylinositol 3-phosphate (PtdIns3P).	Likely recessive (0.21)	0.01	47.26	-0.29 (36.09%)	1.72
**Possible CP genes (identified by WES[1])**							
ABLIM2		ABLIM2 and ABLIM3 bind strongly to F-actin and localise to actin stress fibres.	Very likely recessive (0.11)	0	63.64	-0.14 (42.93%)	0.18
ACADM	Deficiency of Acyl-CoA dehydrogenase, medium chain	Encodes the medium-chain specific (C4 to C12 straight chain) acyl-Coenzyme A dehydrogenase. The homotetramer enzyme catalyzes the initial step of the mitochondrial fatty acid beta-oxidation pathway. Clinical phenotypes are associated with ACADM hereditary deficiency.	Very likely recessive (0.08)	0	29.48	-0.23 (38.64%)	0.6
ACOX1	Peroxisomal acyl-CoA oxidase deficiency, Adrenoleukodystrophy Pseudoneonatal	First enzyme of the fatty acid beta-oxidation pathway, which catalyzes the desaturation of acyl-CoAs to 2-trans-enoyl-CoAs. ACOX1 donates electrons directly to molecular oxygen, thereby producing hydrogen peroxide. Defects in the ACOX1 gene result in pseudoneonatal adrenoleukodystrophy, a disease that is	Very likely recessive (0.09)	0.1	38.85	-0.08 (45.46%)	**2.2**

		characterized by accumulation of very long chain fatty acids.					
ADCY3		Membrane-associated enzyme and catalyzes the formation of the secondary messenger cyclic adenosine monophosphate (cAMP). Widely expressed in various human tissues.	**Likely dominant (0.62)**	0	38.39	**-1.82 (3.92%)**	**2.61**
ATP11B		P-type ATPases such as ATP11B are phosphorylated in their intermediate state and drive uphill transport of ions across membranes.	Likely recessive (0.33)	0.01	**19.59**	-0.31 (35.20%)	**2.04**
AUH	3-methylglutaconic aciduria, type I	Binds to the AU-rich element (ARE), a common element found in the 3' UTR of rapidly decaying mRNA. AUH is also homologous to enol-CoA hydratase, an enzyme involved in fatty acid degradation, and has been shown to have intrinsic hydratase enzymatic activity. AUH is thus a bi-functional chimera between RNA binding and metabolic enzyme activity.	Very likely recessive (0.05)	0	64.74	0.34 (65.35%)	0.45
CDK17		Belongs to the cdc2/cdkx subfamily of the ser/thr family of protein kinases. Similarity to a rat protein that is thought to play a role in terminally differentiated neurons.	**Very likely dominant (0.90)**	**1.00**	**11.08**	-0.46 (28.98%)	**2.73**
CNDP2		Non-specific dipeptidase rather than a selective carnosinase. Capable of hydrolysing di- and tripeptides.	Very likely recessive (0.06)	0	66.22	-0.09 (45.17%)	0.09
CNKSR2	Intellectual disability with epilepsy	Encodes a multi-domain protein that functions as a scaffold protein to mediate the mitogen-activated protein kinase pathways downstream from Ras. May also play a role in ternary complex assembly of synaptic proteins at the postsynaptic membrane and coupling of signal transduction to membrane/cytoskeletal remodelling.	-	**1.00**	**7.56**	**-1.46 (5.33%)**	**3.73**
COPS3	Located within the Smith-Magenis syndrome region on chromosome 17.	Phosphorylates regulators involved in signal transduction: I kappa-Balpha, p105, and c-Jun. Acts as a docking site for complex-mediated phosphorylation.	Likely recessive (0.29)	**1.00**	**6.17**	-0.47 (28.85%)	**2.52**
CTDSPL		Recruited by REST to neuronal genes that contain RE-1 elements, leading to neuronal gene silencing in non-neuronal cells (by similarity). Preferentially catalyses the dephosphorylation of Ser-5 within the tandem 7 residue repeats in the C-terminal domain (CTD) of the largest RNA polymerase II subunit POLR2A. Negatively	Very likely recessive (0.15)	0.01	35.46	0.14 (56.32%)	**2.14**

		regulates RNA polymerase II transcription, possibly by controlling the transition from initiation/capping to processive transcript elongation.					
DHX32		DEAD box proteins, characterised by the conserved motif Asp-Glu-Ala-Asp (DEAD), are putative RNA helicases. They are implicated in a number of cellular processes involving alteration of RNA secondary structure such as translation initiation, nuclear and mitochondrial splicing and ribosome and spliceosome assembly.	Very likely recessive (0.16)	0	73.04	-0.28 (36.50%)	1.29
DNAH2		Dyneins are microtubule-associated motor protein complexes composed of several heavy, light and intermediate chains. The axonemal dyneins, found in cilia and flagella are components of the outer and inner dynein arms attached to the peripheral microtubule doublets. DNAH2 is an axonemal inner arm dynein heavy chain.	Dominant or recessive (0.46)	0	42.05	**-2.17 (2.52%)**	**2.12**
DNAH3		Dynein heavy chains are the main components of multi-subunit motor ATPase complexes called dyneins. Axonemal dyneins such as DNAH3 provide the driving force for ciliary and flagellar motility.	**Very likely dominant (0.89)**	0	5.056	2.16 (97.61%)	1.56
DYNC2H1	Short-rib thoracic dysplasia 3 with or without polydactyly, Asphyxiating Thoracic Dystrophy Type 3	Encodes a protein involved in ciliary intraflagellar transport (IFT), an evolutionarily conserved process that is essential for ciliogenesis and plays a role in cell signalling events. DYNC2H1 is the central ATPase subunit of the IFT dynein-2 complex, the principal minus-end directed microtubule motor that drives retrograde transport of the IFT-A protein complex that regulates tip-to-base transport in cilia.	Likely recessive (0.29)	0	29.65	-0.19 (40.31%)	1.38
EIF4E2		Cap-binding proteins such as EIF4E2 bind to capped RNA and facilitate processing and translation of mRNA.	Likely recessive (0.35)	0.49	**13.07**	-0.26 (37.41%)	**2.32**
ENOX2		May be involved in cell growth. Probably acts as a terminal oxidase of plasma electron transport from cytosolic NAD(P)H via hydroquinones to acceptors at the cell surface. Hydroquinone oxidase activity alternates with a protein disulphide-thiol interchange/oxidoreductase	-	0	**12.56**	**-0.59 (19.75%)**	0.88

		activity which may control physical membrane displacements associated with vesicle budding or cell enlargement.					
ENPP4		Hydrolyses extracellular Ap3A into AMP, and ADP and Ap4A into AMP and ATP. Ap3A and Ap4A are deadenosine polyphosphates thought to induce proliferation of vascular smooth muscle cells. Acts as a procoagulant, mediating platelet aggregation at the site of nascent thrombus via release of ADP from Ap3A and activation of ADP receptors.	Very likely recessive (0.10)	0.08	44.16	0.25 (61.40%)	0.89
EPHA1		Belongs to the ephrin receptor subfamily of the protein-tyrosine kinase family. EPH and EPH-related receptors have been implicated in mediating developmental events, particularly in the nervous system.	Likely recessive (0.25)	0	51.44	0.28 (62.56%)	0.33
GANC		Encodes a member of glycosyl hydrolases family 31 and is a key enzyme in glycogen metabolism. This enzyme hydrolyses terminal, non-reducing 1,4-linked alpha-D-glucose residues and releases alpha-D-glucose.	Very likely recessive (0.11)	0	57.15	1.19 (89.77%)	-0.51
GCDH	Glutaricaciduria, type I, metabolic disorder characterized by progressive dystonia and athetosis due to gliosis and neuronal loss in the basal ganglia, autosomal recessive	Responsible for the dehydrogenation and decarboxylation of glutaryl-CoA to crotonyl-CoA in the degradative pathway of L-lysine, L-hydroxylysine, and L-tryptophan metabolism. Member of the acyl-CoA dehydrogenase family.	Very likely recessive (0.09)	0	60.73	-0.45 (29.45%)	0.27
HLCS	Holocarboxylase synthetase deficiency, autosomal recessive	Encodes an enzyme that catalyses the binding of biotin to carboxylases and histones. The protein plays an important roles in gluconeogenesis, fatty acid synthesis and branched amino acid catabolism.	Likely recessive (0.22)	0	90.61	0.05 (51.78%)	0.21
INHBB		The inhibin beta B subunit joins the alpha subunit to form a pituitary FSH secretion inhibitor. Inhibin has been shown to regulate gonadal stromal cell proliferation negatively and to have tumour-suppressor activity. In addition, serum levels of inhibin have been shown to reflect the size of granulosa-cell tumors and can therefore be used as a marker for primary as well as recurrent disease.	**Very likely dominant (0.99)**	**0.97**	30.20	-0.12 (43.92%)	**2.08**

LCP2		May function as an adaptor or scaffold protein. Studies using deficient T cell lines or mice have provided strong evidence for a positive role in promoting T cell development and activation as well as mast cell and platelet function.	**Very likely dominant (0.97)**	0.52	44.22	0.08 (53.26%)	1.81
MAN2A1		Alpha-mannosidase II is a Golgi enzyme that catalyses the final hydrolytic step in the asparagine-linked oligosaccharide (N-glycan) maturation pathway, acting as the committed step in the conversion of high mannose to complex type structures.	Very likely recessive (0.17)	0	35.33	**-0.93 (14.71%)**	0.49
MAOB	Monoamine oxidase deficiency, or Brunner syndrome.	Belongs to the flavin monoamine oxidase family. Catalyzes the oxidative deamination of biogenic and xenobiotic amines and plays an important role in the metabolism of neuroactive and vasoactive amines in the central nervous sysytem and peripheral tissues.	**-**	**1.00**	**16.58**	**-0.66 (17.55%)**	1.7
MC2R	Glucocorticoid deficiency, due to ACTH, autosomal recessive	Member of the G-protein associated melanocortin receptor family. Melanocortins (melanocyte-stimulating hormones and adrenocorticotropic hormone) are peptides derived from pro-opiomelanocortin (POMC). Selectively activated by adrenocorticotropic hormone.	Very likely recessive (0.14)	0	63.21	0.27 (62.24%)	0.11
MCCC1	3-Methylcrotonyl-CoA carboxylase 1 deficiency (autosomal recessive disorder of leucine catabolism).	Encodes the large subunit of 3-methylcrotonyl-CoA carboxylase. This enzyme functions as a heterodimer and catalyzes the carboxylation of 3-methylcrotonyl-CoA to form 3-methylglutaconyl-CoA.	Very likely recessive (0.14)	0	46.36	0.55 (73.70%)	0.79
MGA		MGA is a transcriptional repressor or activator depending upon the presence of its binding partner, MAX.	Dominant or recessive (0.58)	**1.00**	26.51	**-2.18 (2.51%)**	1.41
MIIP		Encodes a protein that interacts with the oncogene protein insulin-like growth factor binding protein 2 and may function as an inhibitor of cell migration and invasion. This protein also interacts with the cell division protein 20 and may be involved in regulating mitotic progression.	Very likely recessive (0.13)	0	88.63	1.17 (89.36%)	-0.23
MTMR1		Member of the myotubularin related family of proteins.	**-**	**1.00**	31.41	-0.41 (27.27%)	**2.06**

MYO15A	Deafness, autosomal recessive 3, located within the Smith-Magenis syndrome region on chromosome 17.	Studies in mice suggest that this protein is necessary for actin organization in the hair cells of the cochlea.	Very likely recessive (0.11)	0	39.26	0.24 (60.96%)	0.85
MYO1F		Myosins are molecular motors that, upon interaction with actin filaments, utilise energy from ATP hydrolysis to generate mechanical force. Expressed predominantly in the mammalian immune system. Cells from deficient mice exhibited abnormally increased adhesion and reduced motility, resulting from augmented exocytosis of beta-2 integrin-containing granules.	Very likely recessive (0.12)	0	45.46	0.49(71.2 2%)	1.86
NEMF		Component of the ribosome quality control complex (RQC), a ribosome-associated complex that mediates ubiquitination and extraction of incompletely synthesis nascent chains for proteasomal degradation. NEMF is responsible for selective recognition of stalled 60S subunits by recognising an exposed, nascent chain-conjugated tRNA moiety.	Dominant or recessive (0.48)	0	**21.97**	**-1.63 (5.07%)**	1.23
NGFR		Plays a role in the regulation of the translocation of GLUT4 to the cell surface in adipocytes and skeletal muscle cells in response to insulin, probably by regulating RAB31 activity, and thereby contributes to the regulation of insulin-dependent glucose uptake. Can mediate cell survival as well as cell death of neural cells.	**Very likely dominant (0.94)**	0.30	**2.71**	-0.42 (30.65%)	1.31
NR1I2		Belongs to the nuclear receptor superfamily, members of which are transcription factors characterized by a ligand-binding domain and a DNA-binding domain. NR1I2 is a Xeno-sensing pregnane X receptor (PXR), chemical gatekeeper in the liver and the vasculature. The encoded protein is a transcriptional regulator of the cytochrome P450 gene CYP3A4, binding to the response element of the CYP3A4 promoter as a heterodimer with the 9-cis retinoic acid receptor RXR.	Very likely recessive (0.06)	0	48.5	**-0.61 (23.45%)**	-0.5
OFD1	Retinitis pigmentosa 2, Joubert syndrome 10, Orofaciodigital syndrome I (X-linked dominant), Simpson-Golabi-Behmel syndrome, type 2	Involved in biogenesis of the cilium, a centriole-associated function. The cilium is a cell surface projection found in many vertebrate cells required to transduce signals important for development and tissue homeostasis. Plays an	**-**	**0.97**	70.24	-0.20 (39.19%)	0.44

		important role in development by regulating Wnt signalling and the specification of the left-right axis.					
PCBP3		Member of the KH domain protein superfamily. Possesses RNA-binding properties which may be involved in post-transcriptional controls.	Likely recessive (0.39)	0.87	34.15	-0.16 (42.00%)	**2.06**
JADE1		Component of the HBO1 complex which has histone H4-specific acetyltransferase activity, a reduced activity towards histone H3 and is responsible for the bulk of histone H4 acetylation in vivo. Transcriptional coactivator, it may also promote acetylation of nucleosomal histone H4 by KAT5. Promotes apoptosis.	**Very likely dominant (0.88)**	**1.00**	-	**-1.14 (10.59%)**	**3.3**
PTGFRN		Inhibits the binding of prostaglandin F2-alpha (PGF2-alpha) to its specific FP receptor by decreasing the receptor number rather than the affinity constant.	Dominant or recessive (0.49)	0	57.84	-0.04 (47.55%)	1.75
RAD21	Cohesinopathy, autosomal dominant<br>Cornelia de Lange syndrome 4, autosomal dominant<br>Mungan syndrome, autosomal recessive	Repair of DNA double-strand breaks and chromatid cohesion during mitosis. This protein is a nuclear phospho-protein, which becomes hyperphosphorylated in cell cycle M phase.	**Very likely dominant (1.00)**	**1.00**	**4.51**	**-0.88 (16.10%)**	**2.79**
RNF214		Function unknown.	Likely recessive (0.38)	**0.92**	**25.00**	**-1.56 (5.57%)**	1.18
SREK1		Belongs to the superfamily of serine/arginine-rich (SR) splicing factors. It modulates splice site selection by regulating the activities of the other SR proteins.	**Likely dominant (0.65)**	**0.98**	**15.85**	**-0.78 (18.57%)**	**2.32**
TMEM150A		Possible role in fasting-induced catabolism.	Very likely recessive (0.16)	0.03	35.56	-0.33 (34.23%)	1.4
UBQLN3		Encodes an ubiquitin-like protein (ubiquilin) Ubiquilins physically associate with both proteasomes and ubiquitin ligases, and thus are thought to functionally link the ubiquitination machinery to the proteasome to affect in vivo protein degradation.	Very likely recessive (0.07)	**1.00**	60.94	1.07 (87.53%)	**2.47**
UBXN7		Ubiquitin-binding adaptor that links a subset of NEDD8-associated cullin ring ligases (CRLs) to the segregase VCP/p97, to regulate turnover of their ubiquitination substrates.	**Very likely dominant (0.99)**	**1.00**	**16.71**	-0.39 (31.96%)	**3.33**

USP26		Similar to a mouse gene that encodes a ubiquitin-specific protease.	-	-	95.62	-0.15 (41.69%)	0.03
ZMYM3		Plays a role in the regulation of cell morphology and cytoskeletal organisation. A chromosomal translocation involving this genes is associated with X-linked mental retardation. Most abundantly expressed in the brain.	**-**	**1.00**	26.31	**-1.21 (8.46%)**	**4.33**
**Genes associated with developmental and other relevant disorders:**							
APOE	Alzheimer disease-2, Hyperlipoproteinemia, type III , Lipoprotein glomerulopathy, Sea-blue histiocyte disease, Age-related macular degeneration, Myocardial infarction susceptibility	Chylomicron remnants and very low density lipoprotein (VLDL) remnants are rapidly removed from the circulation by receptor-mediated endocytosis in the liver. Apolipoprotein E, a main apoprotein of the chylomicron, binds to a specific receptor on liver cells and peripheral cells. Essential for the normal catabolism of triglyceride-rich lipoprotein constituents.	**Likely dominant (0.76)**	0	**2.85**	0.14 (56.32%)	0.85
BRWD3	Intellectual disability, X-linked 93	Thought to have chromatin-modifying function and may thus play a role in transcription. Plays a role in the regulation of cell morphology and cytoskeletal organisation. Required in the control of cell shape.	**-**	**1.00**	28.62	**-3.22 (0.78%)**	**3.12**
CUL4B	Intellectual disability, X-linked, syndromic 15 (Cabezas type)	Encodes a scaffold protein of the cullin 4B-RING ubiquitin ligase (E3) complex that regulates degradation of cellular proteins, signals nucleotide excision repair and is involved in DNA damage response.	**-**	**1.00**	**8.30**	**-1.41 (5.96%)**	**3.87**
DMD	Duchenne (DMD) and Becker (BMD) Muscular Dystrophies, Cardiomyopathy Dilated X-Linked Type 3B	Part of the dystrophin-glycoprotein complex (DGC), which bridges the inner cytoskeleton (F-actin) and the extra-cellular matrix.	**-**	**1.00**	**0.26**	0.61 (80.25%)	-2.11
DMPK	Myotonic dystrophy type 1 (dominant negative)	Encodes a serine-threonine kinase that is closely related to other kinases that interact with Rho family of small GTPases. Also critical to the modulation of cardiac contractility and to the maintenance of proper cardiac conduction activity probably through the regulation of cellular calcium homeostasis. Phosphorylates PLN, a regulator of calcium pumps and may regulate sarcoplasmic reticulum calcium uptake in myocytes. May also play a role in synaptic plasticity.	Very likely recessive (0.09)	0.03	49.83	-0.02 (48.28%)	0.25
ERLIN1	Associated with haemorrhagic stroke	Component of the ERLIN1/ERLIN2 complex which mediates the endoplasmic reticulum-associated	Dominant or	0.18	**19.99**	-0.06 (46.69%)	1.89

		degradation (ERAD) of inositol 1,4,5-trisphosphate receptors (IP3Rs).	recessive (0.49)				
HSPG2	Dyssegmental dysplasia, Silverman-Handmaker type, autosomal recessive, Schwartz-Jampel syndrome, type 1, autosomal recessive	Heparan sulfate proteoglycan is a major component of basement membranes, where the molecule may be involved in the stabilization of other molecules as well as being involved with glomerular permeability to macromolecules and cell adhesion.	Dominant or recessive (0.46)	0	49.80	5.11 (99.76%)	1.43
HUWE1	Intellectual disability, X-linked syndromic, Turner type	Encodes a protein containing a C-terminal HECT (E6AP type E3 ubiquitin protein ligase) domain that functions as an E3 ubiquitin ligase. The encoded protein is required for the ubiquitination and subsequent degradation of the anti-apoptotic protein Mcl1 (myeloid cell leukaemia sequence 1). Also ubiquitinates the p53 tumour suppressor, core histones and DNA polymerase beta.	-	**1.00**	**10.58**	**-7.02 (0.16%)**	**9.03**
IGSF1	X-linked central hypothyroidism and testicular enlargement	Encodes a member of the immunoglobulin-like domain-containing superfamily. Proteins in this superfamily contain varying numbers of immunoglobulin-like domains and are thought to participate in the regulation of interaction between cells.	-	**1.00**	34.71	**-1.11 (9.40%)**	1.2
IL1RAPL1	Intellectual disability, X-linked 21/34	Member of the interleukin 1 receptor family. This gene is expressed at a high level in post-natal brain structures involved in the hippocampal memory system, which suggests a specialized role in the physiological processes underlying memory and learning abilities.	-	**1.00**	**1.75**	**-1.08 (9.87%)**	**2.82**
IL6	Crohn disease-associated growth failure, susceptibility to diabetes, susceptibility to intracranial hemorrhage in brain cerebrovascular malformations, susceptibility to Kaposi sarcoma, systemic juvenile rheumatoid arthritis	Cytokine with wide variety of biological functions. Potent inducer of the acute phase response. Plays an essential role in the final differentiation of B-cells into Ig-secreting cells. Discharged into the bloodstream after muscle contraction and acts to increase the breakdown of fats and to improve insulin resistance. Induces nerve cell differentiation.	**Likely dominant (0.75)**	0.34	**1.57**	-0.19 (40.63%)	0.01
IQSEC2	Intellectual disability, X-linked 1	Encodes a guanine nucleotide exchange factor for the ARF family of GTP-binding proteins. The encoded protein is a component of the postsynaptic density at excitatory synapses and may play a critical role in cytoskeletal and	-	**1.00**	26.16	**-1.11 (9.25%)**	**5.22**

		synaptic organisation through the activation of selected ARF substrates ARF1 and ARF6.					
MCPH1	Microcephaly 1, primary, autosomal recessive	Implicated in chromosome condensation and DNA damage induced cellular responses. May play a role in neurogenesis and regulation of the size of the cerebral cortex.	Very likely recessive (0.19)	0	94.09	3.01 (99.04%)	-4.81
MECP2	Encephalopathy, neonatal severe, Intellectual disability, X-linked syndromic, Intellectual disability, X-linked, syndromic, Rett syndrome, Rett syndrome, atypical, Rett syndrome, preserved speech variant, Autism susceptibility, X-linked 3	Part of a family of nuclear proteins related by the presence in each of a methyl-CpG binding domain (MBD). Binds specifically to methylated DNA and can repress transcription from methylated gene promoters. MECP2 is X-linked and subject to X inactivation. Essential for embryonic development.	-	0.78	**1.38**	-0.40 (27.59%)	-1.44
NPC1	Niemann-Pick disease, type D and type C1	Encodes a putative integral membrane protein containing motifs consistent with a role in intracellular transport of cholesterol to post-lysosomal destinations.	Likely recessive (0.21)	0	44.17	**-0.67 (21.88%)**	1.27
PAFAH1B1	Lissencephaly 1, Subcortical laminar heterotopia (Miller-Dieker lissencephaly syndrome)	Eencodes the non-catalytic alpha subunit of the intracellular Ib isoform of platelet-activating factor acteylhydrolase, a heterotrimeric enzyme that specifically catalyzes the removal of the acetyl group at the SN-2 position of platelet-activating factor.	**Very likely dominant (1.00)**	**1.00**	**2.20**	-0.44 (29.76%)	**3.67**
PLA2G6	Infantile neuroaxonal dystrophy 1, Neurodegeneration with brain iron accumulation 2B, Parkinson disease 14, autosomal recessive	Catalyzes the release of arachidonic acid from membrane phospholipids. Arachidonic acid in turn serves as precursor for a wide spectrum of biologic effectors, collectively known as eicosanoids, that are involved in hemodynamic regulation, inflammatory responses, and other cellular processes.	Very likely recessive (0.10)	0	35.59	0.75 (80.20%)	1.42
PROC	Thrombophilia due to protein C deficiency, autosomal dominant, Thrombophilia due to protein C deficiency, autosomal recessive	Encodes protein C, a vitamin K-dependent plasma glycoprotein that is a key component of the anti-coagulant system. Protein C is cleaved to its activated form on endothelial cells by the thrombin-thrombomodulin complex and then acts as a serine protease to degrade the activated forms of coagulation factors V and VIII.	Likely recessive (0.21)	0	66.64	**-0.76 (19.23%)**	0.96
SLC16A2	Allan-Herndon-Dudley syndrome (Intellectual disability, X-linked with hypotonia), MCT8 (SLC16A2)-Specific Thyroid Hormone Cell Transporter Deficiency	Integral membrane protein that functions as a transporter of thyroid hormone. Facilitates the cellular importation of thyroxine (T4), triiodothyronine (T3), reverse triiodothyronine (rT3) and diiodothyronine (T2). Expressed in	-	**0.99**	25.72	-0.27 (35.27%)	**2.43**

		many tissues and likely plays an important role in the development of the central nervous system.					
SLC6A3	Parkinsonism-dystonia, infantile	Encodes an integral membrane protein that transports the neurotransmitter serotonin from synaptic spaces into presynaptic neurons. Terminates the action of serotonin and recycles it in a sodium-dependent manner.	**Very likely dominant (0.99)**	**1.00**	**18.92**	**-0.77 (18.80%)**	**4.16**
SYNGAP1	Intellectual disability autosomal dominant 5, Epileptic Encephalopathy, autosomal dominant	Encodes a brain-specific synaptic Ras GTPase activating protein that is largely localised to dendritic spines in neocortical pyramidal neurons, where it suppresses signalling pathways linked to NMDA receptor-mediated synaptic plasticity and AMPA receptor membrane insertion.	**Very likely dominant (0.98)**	**1.00**	**19.64**	**-2.10 (2.78%)**	**5.75**
TUBA3D		Member of the alpha tubulin family. Tubulin is a major component of microtubules which are composed of alpha- and beta-tubulin heterodimers and microtubule-associated proteins in the cytoskeleton.	**Very likely dominant (0.93)**	0	78.94	-0.28 (36.36%)	1.53
TUBA8	Polymicrogyria with optic nerve hypoplasia, autosomal recessive	Member of the alpha tubulin family. Tubulin is a major component of microtubules which are composed of alpha- and beta-tubulin heterodimers and microtubule-associated proteins in the cytoskeleton.	Likely recessive (0.33)	0	42.61	0.09 (53.89%)	0.63
TUBB	Cortical dysplasia, complex, with other brain malformations 6, autosomal recessive	Beta-tubulin; polymerizes to form microtubules; member of a family of structural proteins. Tubulin is a major component of microtubules which are composed of alpha- and beta-tubulin heterodimers and microtubule-associated proteins in the cytoskeleton.	**Very likely dominant (1.00)**	**0.98**	**11.09**	-0.53 (26.39%)	**5.71**
TUBB2B	Polymicrogyria, symmetric or asymmetric, dominant negative	Beta-tubulin; polymerizes to form microtubules; member of a family of structural proteins. Tubulin is a major component of microtubules which are composed of alpha- and beta-tubulin heterodimers and microtubule-associated proteins in the cytoskeleton.	**Likely dominant (0.78)**	**0.99**	**24.97**	-0.35 (33.63%)	**5.17**
TUBB3	Cortical dysplasia, complex, with other brain malformations 1 , Fibrosis of extraocular muscles, congenital, 3A	Beta-tubulin; polymerizes to form microtubules; member of a family of structural proteins. Tubulin is a major component of microtubules which are composed of alpha- and beta-tubulin heterodimers and microtubule-associated proteins in the cytoskeleton.	**Very likely dominant (0.99)**	0	61.92	**-0.93 (14.67%)**	1.7

TUBG1	Cortical dysplasia, complex, with other brain malformations 4, Posteriorly Predominant Pachygyria and Severe Microcephaly, autosomal dominant	Localises to the centrosome where it binds to microtubules as part of a complex referred to as the gamma-tubulin ring complex. Mediates microtubule nucleation and is required for microtubule formation and the progression of the cell cycle.	**Very likely dominant (0.97)**	0.1	**7.28**	**-0.71 (20.85%)**	**4.25**
UBE3A	Angelman syndrome, imprinted	E3 ubiquitin-protein ligase, part of the ubiquitin protein degradation system. Imprinted: maternally expressed in brain and biallelically expressed in other tissues. Maternally inherited deletion of this gene causes Angelman Syndrome, characterized by severe motor and intellectual retardation, ataxia, hypotonia, epilepsy, absence of speech, and characteristic facies.	**Very likely dominant (0.97)**	**1.00**	**2.10**	**-1.45 (6.82%)**	**4.62**
UPF3B	Intellectual disability, X-linked, syndromic 14	Part of a post-splicing multiprotein complex involved in both mRNA nuclear export and mRNA surveillance. mRNA surveillance detects exported mRNAs with truncated open reading frames and initiates nonsense-mediated mRNA decay (NMD). This protein binds to the mRNA and remains bound after nuclear export, acting as a nucleocytoplasmic shuttling protein.	**-**	**1.00**	33.03	-0.39 (28.37%)	1.9
VRK1	Pontocerebellar hypoplasia type 1A, autosomal recessive	Serine/threonine kinase involved in Golgi disassembly during the cell cycle: following phosphorylation by PLK3 during mitosis, required to induce Golgi fragmentation.	Very likely recessive (0.18)	0	27.43	-0.34 (34.02%)	1.19
ZDHHC9	Intellectual disability, X-linked syndromic, Raymond type	Palmitoyltransferase that catalyzes the posttranslational modification of RAS oncogenes, NRAS and HRAS.	-	0.74	**13.32**	**-0.88 (14.42%)**	**2.64**

Abbreviations: pLI; Probability of loss of function intolerance, %HI; Haploinsufficiency rank, RVIS; Residual Variation Intolerance Score. Details of methods to predict intolerance of genes: DOMINO prediction [3]; HI% [4]; pLI and missense constraint metric [5]; RVIS [6].

Supplementary Table 2 Median coverage of exons targeted in the CP HaloPlex gene panel across all samples

Gene	Exon	Median bases >20x (%)	SD
**ABLIM2**	NM_001130083.ex.12	100	20.36
	NM_001130085.ex.12	100	5.39
	NM_001130085.ex.13	100	2.36
	NM_001130086.ex.13	100	4.22
	NM_001130086.ex.14	100	9.71
	NM_001130086.ex.15	100	8.75
	NM_001130088.ex.11	100	4.75
	NM_001130088.ex.14	100	37.12
	NM_032432.ex.2	100	2.27
	NM_032432.ex.3	100	18.94
	NM_032432.ex.4	100	0.59
	NM_032432.ex.5	100	0.74
	NM_032432.ex.7	68.54	33.98
	NM_032432.ex.9	100	2.76
	NM_032432.ex.10	100	0.11
	NM_032432.ex.11	100	17.72
	NM_032432.ex.12	100	29.94
	NM_032432.ex.13	100	4.22
	NM_032432.ex.15	100	1.34
	NM_032432.ex.16	100	27.39
**ACADM**	NM_000016.ex.2	100	26.46
	NM_000016.ex.3	100	9.61
	NM_000016.ex.4	100	8.17
	NM_000016.ex.5	100	26.76
	NM_000016.ex.6	100	14.21
	NM_000016.ex.7	100	13.77
	NM_000016.ex.8	100	9.72
	NM_000016.ex.9	100	19.51
	NM_000016.ex.10	100	29.02
	NM_000016.ex.11	100	8.63
	NM_000016.ex.12	100	45.19
	NM_001127328.ex.2	100	26.46
**ACOX1**	NM_001185039.ex.1	100	8.92
	NM_001185039.ex.3	100	1.63
	NM_007292.ex.1	100	9.00
	NM_007292.ex.2	100	5.55
	NM_007292.ex.3	100	2.21
	NM_007292.ex.4	100	0.55
	NM_007292.ex.5	100	2.59
	NM_007292.ex.6	100	4.59
	NM_007292.ex.7	100	8.05
	NM_007292.ex.8	100	24.53
	NM_007292.ex.9	100	0.03
	NM_007292.ex.10	100	8.06
	NM_007292.ex.11	100	12.48
	NM_007292.ex.12	100	8.10
	NM_007292.ex.13	100	6.39
	NM_007292.ex.14	100	21.39
**ADCY3**	NM_004036.ex.1	100	12.50
	NM_004036.ex.2	100	17.13
	NM_004036.ex.3	100	0.84
	NM_004036.ex.4	100	1.79
	NM_004036.ex.5	100	6.09
	NM_004036.ex.6	100	2.20
	NM_004036.ex.7	92.18	4.59
	NM_004036.ex.8	100	4.45
	NM_004036.ex.9	100	2.53
	NM_004036.ex.10	100	2.07
	NM_004036.ex.11	100	6.09

	NM_004036.ex.12	100	0.73
	NM_004036.ex.13	100	4.29
	NM_004036.ex.14	100	10.64
	NM_004036.ex.15	100	0.18
	NM_004036.ex.17	100	4.37
	NM_004036.ex.18	100	1.29
	NM_004036.ex.19	100	3.57
	NM_004036.ex.21	100	25.20
**ADD3**	NM_016824.ex.2	100	8.67
	NM_016824.ex.3	100	23.07
	NM_016824.ex.4	100	12.95
	NM_016824.ex.5	100	28.09
	NM_016824.ex.6	100	18.03
	NM_016824.ex.7	100	12.64
	NM_016824.ex.8	100	15.40
	NM_016824.ex.9	100	13.45
	NM_016824.ex.10	100	13.13
	NM_016824.ex.11	100	16.79
	NM_016824.ex.12	100	22.60
	NM_016824.ex.13	100	12.13
	NM_016824.ex.14	81.44	18.44
	NM_016824.ex.15	100	43.31
**AGAP1**	NM_001037131.ex.12	100	4.31
	NM_001244888.ex.10	100	10.66
	NM_014914.ex.1	100	35.83
	NM_014914.ex.5	100	4.95
	NM_014914.ex.6	100	7.13
	NM_014914.ex.7	100	18.04
	NM_014914.ex.8	100	2.05
	NM_014914.ex.9	100	2.88
	NM_014914.ex.10	100	18.38
	NM_014914.ex.11	100	4.55
	NM_014914.ex.12	100	10.05
	NM_014914.ex.13	100	10.56
	NM_014914.ex.14	100	4.80
	NM_014914.ex.15	100	3.67
	NM_014914.ex.16	100	11.74
	NM_014914.ex.17	100	9.08
**AP4B1**	NM_001253852.ex.1	100	11.60
	NM_001253852.ex.2	100	4.23
	NM_001253852.ex.3	100	12.88
	NM_001253852.ex.4	100	9.55
	NM_001253852.ex.5	100	9.74
	NM_001253852.ex.6	100	13.53
	NM_001253852.ex.7	100	3.97
	NM_001253852.ex.8	100	4.03
	NM_001253852.ex.9	100	6.29
	NM_001253852.ex.10	100	19.58
	NM_001253853.ex.2	100	11.60
	NM_001253853.ex.3	100	3.42
**AP4E1**	NM_001252127.ex.1	94.19	14.89
	NM_001252127.ex.2	84.93	8.08
	NM_001252127.ex.3	15.54	46.47
	NM_001252127.ex.4	100	17.19
	NM_001252127.ex.5	100	6.42
	NM_001252127.ex.6	100	9.92
	NM_001252127.ex.7	100	15.98
	NM_001252127.ex.8	100	31.35
	NM_001252127.ex.9	100	34.20
	NM_001252127.ex.10	100	20.76
	NM_001252127.ex.11	100	16.58
	NM_001252127.ex.12	100	24.11
	NM_001252127.ex.13	100	12.64
	NM_001252127.ex.14	100	18.09
	NM_001252127.ex.15	100	14.21
	NM_001252127.ex.16	100	22.76

	NM_001252127.ex.17	99.22	22.47
	NM_001252127.ex.18	100	10.71
	NM_001252127.ex.19	100	17.89
	NM_001252127.ex.20	79.87	26.01
	NM_001252127.ex.21	100	42.27
	NM_007347.ex.3	18.4	46.17
**AP4M1**	NM_004722.ex.1	100	16.20
	NM_004722.ex.2	100	17.90
	NM_004722.ex.3	100	4.52
	NM_004722.ex.5	100	16.98
	NM_004722.ex.7	100	3.30
	NM_004722.ex.9	100	5.21
	NM_004722.ex.12	100	37.25
	NM_004722.ex.13	100	7.49
	NM_004722.ex.14	100	2.56
	NM_004722.ex.15	100	19.33
**AP4S1**	NM_001254727.ex.6	100	42.54
	NM_001254729.ex.5	100	7.99
	NM_001254729.ex.6	9.14	43.68
	NM_007077.ex.2	91.9	11.54
	NM_007077.ex.3	100	0.12
	NM_007077.ex.4	100	1.85
	NM_007077.ex.5	100	8.17
	NM_007077.ex.6	100	14.76
**APOE**	NM_000041.ex.1	100	18.67
	NM_000041.ex.2	100	1.36
	NM_000041.ex.3	100	3.76
	NM_000041.ex.4	97.1	6.37
**ATL1**	NM_015915.ex.2	100	8.41
	NM_015915.ex.3	100	16.14
	NM_015915.ex.4	100	37.59
	NM_015915.ex.5	100	34.74
	NM_015915.ex.6	100	4.54

	NM_015915.ex.7	100	13.31
	NM_015915.ex.8	100	2.35
	NM_015915.ex.9	100	20.13
	NM_015915.ex.10	100	16.35
	NM_015915.ex.11	100	0.42
	NM_015915.ex.12	100	4.96
	NM_015915.ex.13	100	7.64
	NM_015915.ex.14	100	24.56
	NM_181598.ex.12	100	4.96
	NM_181598.ex.13	100	24.59
**ATP11B**	NM_014616.ex.1	100	3.97
	NM_014616.ex.2	100	9.91
	NM_014616.ex.3	54.95	37.24
	NM_014616.ex.4	100	30.16
	NM_014616.ex.5	100	16.04
	NM_014616.ex.6	100	16.46
	NM_014616.ex.7	100	21.34
	NM_014616.ex.8	48.98	29.28
	NM_014616.ex.9	100	12.96
	NM_014616.ex.10	100	24.13
	NM_014616.ex.11	77.63	32.97
	NM_014616.ex.12	100	31.29
	NM_014616.ex.13	100	24.02
	NM_014616.ex.14	100	18.27
	NM_014616.ex.15	100	19.65
	NM_014616.ex.16	32	29.46
	NM_014616.ex.17	100	31.80
	NM_014616.ex.18	100	32.71
	NM_014616.ex.19	100	16.96
	NM_014616.ex.20	100	20.06
	NM_014616.ex.21	60.58	38.04
	NM_014616.ex.22	100	13.31
	NM_014616.ex.23	100	13.17

	NM_014616.ex.24	89.54	39.61
	NM_014616.ex.25	100	16.51
	NM_014616.ex.26	100	24.10
	NM_014616.ex.27	100	28.71
	NM_014616.ex.28	100	18.07
	NM_014616.ex.29	100	22.63
	NM_014616.ex.30	100	26.90
**AUH**	NM_001698.ex.1	100	13.28
	NM_001698.ex.2	100	12.85
	NM_001698.ex.3	88.76	26.92
	NM_001698.ex.4	0	27.19
	NM_001698.ex.5	100	14.17
	NM_001698.ex.6	100	5.18
	NM_001698.ex.7	100	1.27
	NM_001698.ex.8	100	16.20
	NM_001698.ex.9	100	13.87
	NM_001698.ex.10	100	19.33
**BRWD3**	NM_153252.ex.1	12.88	45.04
	NM_153252.ex.3	100	19.67
	NM_153252.ex.4	100	7.13
	NM_153252.ex.5	100	18.48
	NM_153252.ex.6	100	22.60
	NM_153252.ex.7	66.67	25.39
	NM_153252.ex.8	100	11.34
	NM_153252.ex.9	100	28.24
	NM_153252.ex.10	100	18.16
	NM_153252.ex.11	100	16.71
	NM_153252.ex.12	100	16.08
	NM_153252.ex.13	100	36.08
	NM_153252.ex.14	100	25.31
	NM_153252.ex.15	91.91	27.57
	NM_153252.ex.16	100	11.81
	NM_153252.ex.17	100	18.22
	NM_153252.ex.18	100	15.39
	NM_153252.ex.19	100	9.00
	NM_153252.ex.20	100	24.30
	NM_153252.ex.21	100	25.29
	NM_153252.ex.22	100	5.41
	NM_153252.ex.23	50	41.96
	NM_153252.ex.24	100	20.92
	NM_153252.ex.25	100	15.82
	NM_153252.ex.26	100	17.30
	NM_153252.ex.27	94.05	37.02
	NM_153252.ex.28	100	24.02
	NM_153252.ex.29	100	30.20
	NM_153252.ex.30	100	38.67
	NM_153252.ex.31	100	20.37
	NM_153252.ex.32	100	28.67
	NM_153252.ex.33	100	23.86
	NM_153252.ex.34	100	15.55
	NM_153252.ex.35	100	22.65
	NM_153252.ex.36	53.95	34.90
	NM_153252.ex.37	57.79	28.31
	NM_153252.ex.38	100	10.94
	NM_153252.ex.39	100	20.49
	NM_153252.ex.40	71.25	38.23
	NM_153252.ex.41	55.435	46.10
**CD99L2**	NM_001242614.ex.4	100	14.84
	NM_001242614.ex.10	100	4.55
	NM_031462.ex.1	100	18.68
	NM_031462.ex.2	100	22.19
	NM_031462.ex.3	100	3.43
	NM_031462.ex.4	100	14.84
	NM_031462.ex.5	100	10.18
	NM_031462.ex.6	100	9.47
	NM_031462.ex.7	76.12	29.53

	NM_031462.ex.9	100	3.34
	NM_031462.ex.10	52.24	27.24
**CDK17**	NM_001170464.ex.16	100	21.93
	NM_002595.ex.2	0	32.53
	NM_002595.ex.3	100	12.67
	NM_002595.ex.4	100	28.81
	NM_002595.ex.5	100	3.89
	NM_002595.ex.6	100	28.11
	NM_002595.ex.7	29.31	33.95
	NM_002595.ex.8	100	23.89
	NM_002595.ex.9	100	13.33
	NM_002595.ex.10	100	8.73
	NM_002595.ex.11	98.36	25.27
	NM_002595.ex.12	100	22.86
	NM_002595.ex.13	90.65	25.40
	NM_002595.ex.14	100	14.09
	NM_002595.ex.15	100	10.41
	NM_002595.ex.16	100	6.84
	NM_002595.ex.17	100	20.15
**CNDP2**	NM_001168499.ex.1	68.9	29.15
	NM_018235.ex.2	97.39	23.31
	NM_018235.ex.4	100	2.73
	NM_018235.ex.5	100	2.89
	NM_018235.ex.7	100	6.46
	NM_018235.ex.8	83.33	15.92
	NM_018235.ex.9	100	2.43
	NM_018235.ex.12	5.96	47.35
**CNKSR2**	NM_001168648.ex.9	100	13.60
	NM_001168649.ex.1	100	10.95
	NM_001168649.ex.2	66.67	24.24
	NM_001168649.ex.3	98.53	30.58
	NM_001168649.ex.4	100	30.93
	NM_001168649.ex.5	23.26	44.23
	NM_001168649.ex.6	100	21.70
	NM_001168649.ex.7	100	25.97
	NM_001168649.ex.8	0	33.10
	NM_001168649.ex.9	100	30.86
	NM_001168649.ex.10	100	31.42
	NM_001168649.ex.11	100	38.69
	NM_001168649.ex.12	82.41	34.97
	NM_001168649.ex.13	100	14.99
	NM_001168649.ex.14	100	6.10
	NM_001168649.ex.15	100	29.55
	NM_001168649.ex.16	100	29.15
	NM_001168649.ex.17	60.87	36.12
	NM_001168649.ex.18	100	22.38
	NM_001168649.ex.19	100	9.19
	NM_001168649.ex.20	100	47.06
	NM_014927.ex.21	100	5.24
	NM_014927.ex.22	8.78	39.57
**COL4A1**	NM_001845.ex.1	100	21.47
	NM_001845.ex.3	100	4.67
	NM_001845.ex.4	100	6.82
	NM_001845.ex.5	100	10.48
	NM_001845.ex.6	100	0.38
	NM_001845.ex.7	83.64	16.56
	NM_001845.ex.8	64.29	46.37
	NM_001845.ex.9	100	6.94
	NM_001845.ex.10	100	31.98
	NM_001845.ex.13	100	9.86
	NM_001845.ex.14	100	26.51
	NM_001845.ex.15	100	22.35
	NM_001845.ex.16	100	5.41
	NM_001845.ex.17	100	16.72
	NM_001845.ex.18	100	5.94
	NM_001845.ex.19	100	15.71

	NM_001845.ex.20	100	18.77
	NM_001845.ex.21	100	5.23
	NM_001845.ex.22	100	26.33
	NM_001845.ex.23	100	7.57
	NM_001845.ex.24	100	6.39
	NM_001845.ex.25	100	3.61
	NM_001845.ex.26	100	29.54
	NM_001845.ex.27	100	0.23
	NM_001845.ex.28	100	3.27
	NM_001845.ex.29	100	8.68
	NM_001845.ex.30	100	24.46
	NM_001845.ex.32	100	3.86
	NM_001845.ex.33	100	13.15
	NM_001845.ex.34	100	1.34
	NM_001845.ex.37	82.27	22.67
	NM_001845.ex.38	100	0.28
	NM_001845.ex.39	100	3.87
	NM_001845.ex.40	100	9.30
	NM_001845.ex.41	100	4.33
	NM_001845.ex.42	100	8.39
	NM_001845.ex.43	100	18.48
	NM_001845.ex.46	58.46	21.06
	NM_001845.ex.47	100	0.41
	NM_001845.ex.48	100	4.42
	NM_001845.ex.49	100	3.83
	NM_001845.ex.50	100	0.47
	NM_001845.ex.51	100	0.72
	NM_001845.ex.52	100	7.20
**COPS3**	NM_001199125.ex.1	100	1.34
	NM_003653.ex.2	100	7.74
	NM_003653.ex.3	100	2.62
	NM_003653.ex.4	100	27.08
	NM_003653.ex.5	100	11.77

	NM_003653.ex.6	100	8.71
	NM_003653.ex.7	100	21.61
	NM_003653.ex.8	100	17.04
	NM_003653.ex.9	100	22.35
	NM_003653.ex.10	100	9.47
	NM_003653.ex.11	100	40.28
	NM_003653.ex.12	100	18.41
**CTDSPL**	NM_001008392.ex.1	78.43	0.00
	NM_001008392.ex.3	100	30.31
	NM_001008392.ex.4	100	9.53
	NM_001008392.ex.5	100	34.31
	NM_001008392.ex.7	100	0.92
	NM_001008392.ex.8	100	30.23
**CTNND2**	NM_001332.ex.1	100	8.40
	NM_001332.ex.2	100	3.46
	NM_001332.ex.3	100	13.36
	NM_001332.ex.4	100	29.06
	NM_001332.ex.5	84.75	39.14
	NM_001332.ex.6	100	10.43
	NM_001332.ex.7	71.2	6.05
	NM_001332.ex.8	100	0.25
	NM_001332.ex.9	100	1.61
	NM_001332.ex.10	99.25	3.82
	NM_001332.ex.11	100	0.05
	NM_001332.ex.12	100	1.25
	NM_001332.ex.13	100	4.60
	NM_001332.ex.14	100	3.96
	NM_001332.ex.15	100	5.47
	NM_001332.ex.16	100	8.67
	NM_001332.ex.17	100	7.13
	NM_001332.ex.18	100	5.59
	NM_001332.ex.19	100	8.17
	NM_001332.ex.20	100	8.61

	NM_001332.ex.21	100	12.35
	NM_001332.ex.22	100	10.98
**CUL4B**	NM_001079872.ex.1	90.99	24.19
	NM_003588.ex.2	48.15	34.66
	NM_003588.ex.3	90.99	24.19
	NM_003588.ex.4	56.41	26.94
	NM_003588.ex.5	100	11.77
	NM_003588.ex.6	100	26.78
	NM_003588.ex.7	100	36.67
	NM_003588.ex.8	100	20.33
	NM_003588.ex.9	100	11.45
	NM_003588.ex.10	100	4.76
	NM_003588.ex.11	57.97	35.48
	NM_003588.ex.12	100	18.04
	NM_003588.ex.13	100	30.77
	NM_003588.ex.14	100	20.46
	NM_003588.ex.15	100	37.17
	NM_003588.ex.16	100	40.94
	NM_003588.ex.17	100	26.97
	NM_003588.ex.18	100	20.19
	NM_003588.ex.19	100	41.86
	NM_003588.ex.20	100	14.28
	NM_003588.ex.21	100	15.56
	NM_003588.ex.22	100	42.43
**DDHD2**	NM_001164232.ex.5	100	8.93
	NM_001164232.ex.6	100	17.43
	NM_001164232.ex.7	100	8.83
	NM_001164232.ex.8	100	10.47
	NM_001164232.ex.9	100	28.14
	NM_001164232.ex.10	100	38.25
	NM_001164232.ex.11	100	19.19
	NM_001164232.ex.12	100	13.24
	NM_001164232.ex.13	100	13.91

	NM_001164232.ex.14	100	0.51
	NM_001164232.ex.15	100	3.95
	NM_001164232.ex.16	100	13.62
	NM_001164232.ex.17	100	30.37
	NM_001164234.ex.2	100	24.72
	NM_001164234.ex.3	84.9	9.63
	NM_001164234.ex.4	100	22.67
	NM_001164234.ex.5	100	17.61
**DHX32**	NM_018180.ex.1	100	25.17
	NM_018180.ex.2	100	0.22
	NM_018180.ex.3	100	10.81
	NM_018180.ex.4	38.93	17.57
	NM_018180.ex.5	100	7.59
	NM_018180.ex.6	100	7.02
	NM_018180.ex.7	100	7.86
	NM_018180.ex.8	100	1.15
	NM_018180.ex.9	100	2.87
	NM_018180.ex.10	100	8.04
	NM_018180.ex.11	100	23.12
**DMD**	NM_000109.ex.1	100	21.76
	NM_004006.ex.1	100	15.13
	NM_004009.ex.1	100	29.84
	NM_004010.ex.1	100	27.77
	NM_004010.ex.2	100	26.72
	NM_004010.ex.3	61.7	34.29
	NM_004010.ex.4	100	28.28
	NM_004010.ex.5	100	19.74
	NM_004010.ex.6	100	9.97
	NM_004010.ex.7	100	27.09
	NM_004010.ex.8	82.51	21.16
	NM_004010.ex.9	90.77	31.48
	NM_004010.ex.10	100	12.14
	NM_004010.ex.11	100	16.37

NM_004010.ex.12	100	25.36
NM_004010.ex.13	84.3	27.24
NM_004010.ex.14	94.17	34.76
NM_004010.ex.15	93.58	31.03
NM_004010.ex.16	100	17.59
NM_004010.ex.17	100	19.37
NM_004010.ex.18	100	10.43
NM_004010.ex.19	100	10.50
NM_004010.ex.20	98.77	7.22
NM_004010.ex.21	100	20.85
NM_004010.ex.22	100	25.73
NM_004010.ex.23	100	17.06
NM_004010.ex.24	100	12.97
NM_004010.ex.25	100	17.61
NM_004010.ex.26	100	23.42
NM_004010.ex.27	100	10.39
NM_004010.ex.28	91.18	9.97
NM_004010.ex.29	100	24.46
NM_004011.ex.1	100	36.79
NM_004012.ex.1	54.43	31.65
NM_004012.ex.2	100	15.69
NM_004012.ex.3	44.64	20.11
NM_004012.ex.4	100	35.81
NM_004012.ex.5	100	13.22
NM_004012.ex.6	88.95	20.03
NM_004012.ex.7	100	8.82
NM_004012.ex.8	100	7.80
NM_004012.ex.9	100	5.20
NM_004012.ex.10	100	43.40
NM_004012.ex.11	100	23.46
NM_004012.ex.12	100	32.24
NM_004012.ex.13	100	2.67
NM_004012.ex.14	100	25.38

NM_004012.ex.15	100	18.55
NM_004012.ex.16	100	35.00
NM_004012.ex.17	100	10.62
NM_004012.ex.18	100	13.22
NM_004012.ex.19	100	3.92
NM_004012.ex.20	100	18.86
NM_004012.ex.21	100	9.58
NM_004012.ex.22	100	19.87
NM_004012.ex.23	73.08	17.66
NM_004012.ex.24	100	11.32
NM_004012.ex.25	100	10.89
NM_004012.ex.26	100	10.45
NM_004012.ex.27	100	22.54
NM_004012.ex.28	100	9.48
NM_004012.ex.29	100	9.30
NM_004012.ex.30	99.18	15.98
NM_004012.ex.31	100	10.35
NM_004012.ex.32	100	16.46
NM_004012.ex.33	100	12.48
NM_004012.ex.34	100	15.03
NM_004012.ex.35	100	34.87
NM_004012.ex.36	100	14.87
NM_004012.ex.37	100	5.35
NM_004012.ex.38	100	23.19
NM_004012.ex.39	100	13.97
NM_004012.ex.40	100	9.31
NM_004012.ex.41	100	16.08
NM_004012.ex.42	100	20.96
NM_004012.ex.43	100	9.84
NM_004012.ex.44	100	16.56
NM_004012.ex.45	100	35.00
NM_004012.ex.46	100	17.03
NM_004012.ex.47	100	2.42

	NM_004012.ex.48	100	2.77
	NM_004012.ex.49	100	6.71
	NM_004012.ex.50	100	31.61
	NM_004012.ex.51	100	46.26
	NM_004014.ex.1	100	28.15
	NM_004019.ex.1	100	4.04
	NM_004019.ex.9	100	30.58
**DMPK**	NM_001081560.ex.1	100	15.58
	NM_001081560.ex.2	100	1.00
	NM_001081560.ex.3	100	3.62
	NM_001081560.ex.5	100	6.24
	NM_001081560.ex.6	100	1.14
	NM_001081560.ex.7	100	1.42
	NM_001081560.ex.8	100	6.95
	NM_001081560.ex.11	100	1.20
	NM_001081560.ex.12	100	5.33
	NM_001081560.ex.13	100	2.55
	NM_001081560.ex.14	100	7.64
	NM_001081560.ex.15	33.66	33.88
	NM_001081562.ex.14	100	7.64
	NM_001081563.ex.1	100	1.00
	NM_001081563.ex.7	100	6.56
**DNAH2**	NM_020877.ex.1	100	2.51
	NM_020877.ex.2	100	16.66
	NM_020877.ex.3	100	0.82
	NM_020877.ex.4	100	2.67
	NM_020877.ex.5	100	2.28
	NM_020877.ex.6	100	2.91
	NM_020877.ex.7	100	4.84
	NM_020877.ex.8	100	4.31
	NM_020877.ex.9	96.95	26.65
	NM_020877.ex.11	100	1.43
	NM_020877.ex.12	100	15.53
	NM_020877.ex.13	100	26.58
	NM_020877.ex.14	100	6.68
	NM_020877.ex.15	95.26	20.83
	NM_020877.ex.16	100	0.91
	NM_020877.ex.17	100	4.58
	NM_020877.ex.18	100	8.13
	NM_020877.ex.19	100	0.39
	NM_020877.ex.20	100	13.99
	NM_020877.ex.21	100	7.11
	NM_020877.ex.23	100	10.07
	NM_020877.ex.24	100	2.75
	NM_020877.ex.25	100	1.67
	NM_020877.ex.26	100	3.39
	NM_020877.ex.27	100	2.31
	NM_020877.ex.28	100	7.53
	NM_020877.ex.30	100	2.26
	NM_020877.ex.31	100	15.23
	NM_020877.ex.32	100	9.75
	NM_020877.ex.33	100	3.71
	NM_020877.ex.34	91.62	4.31
	NM_020877.ex.35	100	3.62
	NM_020877.ex.36	100	0.59
	NM_020877.ex.37	100	2.74
	NM_020877.ex.38	100	9.48
	NM_020877.ex.39	100	0.54
	NM_020877.ex.40	100	3.31
	NM_020877.ex.42	100	18.80
	NM_020877.ex.43	100	1.71
	NM_020877.ex.44	100	0.87
	NM_020877.ex.45	100	4.83
	NM_020877.ex.46	100	0.76
	NM_020877.ex.47	100	0.20
	NM_020877.ex.51	100	3.95

	NM_020877.ex.52	90.4	25.19
	NM_020877.ex.53	100	6.99
	NM_020877.ex.54	100	1.43
	NM_020877.ex.55	100	3.18
	NM_020877.ex.56	100	5.97
	NM_020877.ex.57	100	1.75
	NM_020877.ex.58	100	5.99
	NM_020877.ex.59	100	0.09
	NM_020877.ex.61	100	3.32
	NM_020877.ex.62	100	3.82
	NM_020877.ex.63	100	2.16
	NM_020877.ex.64	100	10.48
	NM_020877.ex.65	100	2.37
	NM_020877.ex.66	100	7.93
	NM_020877.ex.67	100	4.80
	NM_020877.ex.69	100	0.96
	NM_020877.ex.70	100	3.03
	NM_020877.ex.71	100	0.16
	NM_020877.ex.72	100	5.19
	NM_020877.ex.73	100	0.83
	NM_020877.ex.75	100	0.06
	NM_020877.ex.76	100	1.07
	NM_020877.ex.77	100	5.35
	NM_020877.ex.78	100	5.94
	NM_020877.ex.79	100	4.61
	NM_020877.ex.80	100	2.62
	NM_020877.ex.82	100	5.84
	NM_020877.ex.83	100	8.54
	NM_020877.ex.84	100	1.10
	NM_020877.ex.85	100	0.75
**DNAH3**	NM_017539.ex.1	100	3.36
	NM_017539.ex.3	100	7.20
	NM_017539.ex.4	100	16.18

	NM_017539.ex.5	100	2.22
	NM_017539.ex.6	100	0.08
	NM_017539.ex.7	100	5.25
	NM_017539.ex.8	98.43	16.96
	NM_017539.ex.9	100	1.77
	NM_017539.ex.10	100	6.14
	NM_017539.ex.11	100	1.46
	NM_017539.ex.12	100	31.60
	NM_017539.ex.13	100	35.33
	NM_017539.ex.14	100	5.27
	NM_017539.ex.15	100	8.41
	NM_017539.ex.16	100	5.66
	NM_017539.ex.18	100	9.90
	NM_017539.ex.19	100	4.37
	NM_017539.ex.20	100	21.00
	NM_017539.ex.21	100	2.68
	NM_017539.ex.22	100	0.14
	NM_017539.ex.23	100	9.84
	NM_017539.ex.24	100	6.60
	NM_017539.ex.25	100	7.66
	NM_017539.ex.26	100	16.91
	NM_017539.ex.27	100	9.24
	NM_017539.ex.29	100	0.54
	NM_017539.ex.30	100	2.21
	NM_017539.ex.31	100	12.15
	NM_017539.ex.32	100	7.29
	NM_017539.ex.34	100	3.55
	NM_017539.ex.35	100	20.99
	NM_017539.ex.36	100	6.46
	NM_017539.ex.37	100	22.58
	NM_017539.ex.38	100	5.27
	NM_017539.ex.39	100	24.56
	NM_017539.ex.40	100	19.58

	NM_017539.ex.41	100	1.43
	NM_017539.ex.42	100	8.15
	NM_017539.ex.43	100	3.68
	NM_017539.ex.44	100	1.32
	NM_017539.ex.45	100	2.50
	NM_017539.ex.46	100	0.44
	NM_017539.ex.47	100	3.05
	NM_017539.ex.48	100	8.41
	NM_017539.ex.49	100	3.49
	NM_017539.ex.50	100	1.76
	NM_017539.ex.51	100	2.32
	NM_017539.ex.53	99.58	7.64
	NM_017539.ex.54	100	0.84
	NM_017539.ex.55	100	2.04
	NM_017539.ex.56	77.12	26.87
	NM_017539.ex.57	100	6.15
	NM_017539.ex.58	100	10.09
	NM_017539.ex.59	100	3.08
	NM_017539.ex.60	100	10.14
	NM_017539.ex.61	100	9.53
	NM_017539.ex.62	100	4.19
**DYNC2H1**	NM_001080463.ex.1	100	4.42
	NM_001080463.ex.2	100	14.91
	NM_001080463.ex.3	100	7.25
	NM_001080463.ex.4	92.5	20.83
	NM_001080463.ex.5	100	26.48
	NM_001080463.ex.6	100	17.94
	NM_001080463.ex.7	100	12.36
	NM_001080463.ex.8	100	25.55
	NM_001080463.ex.9	84.075	37.96
	NM_001080463.ex.10	100	17.32
	NM_001080463.ex.11	100	17.67
	NM_001080463.ex.12	100	15.46

	NM_001080463.ex.13	100	21.53
	NM_001080463.ex.14	100	22.07
	NM_001080463.ex.15	100	13.90
	NM_001080463.ex.16	100	20.52
	NM_001080463.ex.17	100	26.96
	NM_001080463.ex.18	82.17	11.28
	NM_001080463.ex.19	100	14.02
	NM_001080463.ex.20	100	29.91
	NM_001080463.ex.21	100	23.15
	NM_001080463.ex.22	70.05	14.82
	NM_001080463.ex.23	100	21.85
	NM_001080463.ex.24	64.66	32.44
	NM_001080463.ex.25	100	17.71
	NM_001080463.ex.26	87.76	25.95
	NM_001080463.ex.27	100	22.85
	NM_001080463.ex.28	100	17.53
	NM_001080463.ex.29	100	30.67
	NM_001080463.ex.30	100	12.52
	NM_001080463.ex.31	98.68	32.10
	NM_001080463.ex.32	85.99	12.34
	NM_001080463.ex.33	100	15.24
	NM_001080463.ex.34	100	13.08
	NM_001080463.ex.35	100	14.07
	NM_001080463.ex.36	96.58	34.67
	NM_001080463.ex.37	100	9.08
	NM_001080463.ex.38	100	6.90
	NM_001080463.ex.39	98.09	22.53
	NM_001080463.ex.40	100	25.18
	NM_001080463.ex.41	100	17.10
	NM_001080463.ex.42	100	10.86
	NM_001080463.ex.43	100	20.39
	NM_001080463.ex.44	100	29.91
	NM_001080463.ex.45	100	38.13

	NM_001080463.ex.46	100	15.24
	NM_001080463.ex.47	100	14.13
	NM_001080463.ex.48	100	18.00
	NM_001080463.ex.49	100	12.46
	NM_001080463.ex.50	100	14.97
	NM_001080463.ex.51	100	21.91
	NM_001080463.ex.52	100	28.80
	NM_001080463.ex.53	94.19	48.99
	NM_001080463.ex.54	79.49	18.65
	NM_001080463.ex.55	100	17.07
	NM_001080463.ex.56	0	35.15
	NM_001080463.ex.57	100	16.57
	NM_001080463.ex.58	100	13.20
	NM_001080463.ex.59	100	27.15
	NM_001080463.ex.60	100	12.66
	NM_001080463.ex.61	100	18.40
	NM_001080463.ex.62	100	27.52
	NM_001080463.ex.63	100	22.35
	NM_001080463.ex.64	100	26.54
	NM_001080463.ex.65	93	23.92
	NM_001080463.ex.66	84	10.02
	NM_001080463.ex.67	100	13.29
	NM_001080463.ex.68	80.99	34.82
	NM_001080463.ex.69	100	14.04
	NM_001080463.ex.70	97.93	23.41
	NM_001080463.ex.71	100	29.75
	NM_001080463.ex.72	100	26.04
	NM_001080463.ex.73	100	21.05
	NM_001080463.ex.74	100	13.49
	NM_001080463.ex.75	100	26.15
	NM_001080463.ex.76	100	6.02
	NM_001080463.ex.77	82.72	26.70
	NM_001080463.ex.78	100	14.93
	NM_001080463.ex.79	100	32.39
	NM_001080463.ex.80	100	20.18
	NM_001080463.ex.81	100	34.53
	NM_001080463.ex.82	100	29.31
	NM_001080463.ex.83	100	8.35
	NM_001080463.ex.84	100	19.87
	NM_001080463.ex.85	100	9.77
	NM_001080463.ex.86	100	2.41
	NM_001080463.ex.87	100	11.50
	NM_001080463.ex.88	100	20.69
	NM_001080463.ex.89	100	16.53
	NM_001080463.ex.90	100	39.34
**EIF4E2**	NM_004846.ex.2	100	30.58
	NM_004846.ex.3	100	3.58
	NM_004846.ex.4	100	10.93
	NM_004846.ex.5	100	18.00
	NM_004846.ex.6	73.91	15.40
**ENOX2**	NM_006375.ex.3	100	1.31
	NM_006375.ex.4	100	10.25
	NM_006375.ex.5	100	24.92
	NM_006375.ex.6	100	7.13
	NM_006375.ex.7	95.74	19.41
	NM_006375.ex.8	100	6.86
	NM_006375.ex.9	100	11.56
	NM_006375.ex.10	100	18.53
	NM_006375.ex.11	98.91	21.39
	NM_006375.ex.12	100	27.23
	NM_006375.ex.13	100	12.16
	NM_006375.ex.14	100	14.39
	NM_006375.ex.15	100	44.22
**ENPP4**	NM_014936.ex.2	100	9.76
	NM_014936.ex.3	91.28	19.85
	NM_014936.ex.4	100	33.54

**ENTPD1**	NM_001164179.ex.7	100	28.53
	NM_001164181.ex.1	100	10.14
	NM_001164181.ex.2	100	27.77
	NM_001164181.ex.3	84.87	39.28
	NM_001164181.ex.4	100	3.81
	NM_001164181.ex.5	100	6.26
	NM_001164181.ex.6	100	15.94
	NM_001164181.ex.7	100	4.89
	NM_001164181.ex.8	100	2.62
	NM_001164181.ex.9	100	13.86
	NM_001164182.ex.1	100	11.31
	NM_001164182.ex.2	100	2.98
**EPHA1**	NM_005232.ex.1	75.29	23.76
	NM_005232.ex.2	100	14.38
	NM_005232.ex.3	100	3.15
	NM_005232.ex.4	100	2.72
	NM_005232.ex.5	100	5.52
	NM_005232.ex.6	100	2.83
	NM_005232.ex.7	100	0.88
	NM_005232.ex.8	100	1.50
	NM_005232.ex.9	100	3.14
	NM_005232.ex.10	100	5.14
	NM_005232.ex.11	100	5.49
	NM_005232.ex.12	100	8.56
	NM_005232.ex.13	100	1.03
	NM_005232.ex.14	100	0.57
	NM_005232.ex.15	100	0.04
	NM_005232.ex.16	100	2.66
	NM_005232.ex.17	100	13.98
	NM_005232.ex.18	100	12.58
**ERLIN1**	NM_001100626.ex.2	100	30.40
	NM_006459.ex.1	100	30.40
	NM_006459.ex.3	100	9.15
	NM_006459.ex.4	100	26.60
	NM_006459.ex.5	100	9.48
	NM_006459.ex.6	100	11.11
	NM_006459.ex.7	100	17.85
	NM_006459.ex.8	100	14.52
	NM_006459.ex.9	100	6.50
	NM_006459.ex.11	100	4.60
**GAD1**	NM_000817.ex.7	100	8.73
	NM_000817.ex.8	100	1.58
	NM_000817.ex.9	100	3.98
	NM_000817.ex.10	100	3.40
	NM_000817.ex.11	100	27.19
	NM_000817.ex.12	43.94	40.95
	NM_000817.ex.13	100	0.94
	NM_000817.ex.14	100	4.07
	NM_000817.ex.15	100	29.02
	NM_000817.ex.17	100	35.74
	NM_013445.ex.4	96.88	23.98
	NM_013445.ex.5	100	3.85
	NM_013445.ex.6	100	33.67
	NM_013445.ex.7	100	6.46
**GANC**	NM_198141.ex.1	100	18.74
	NM_198141.ex.2	100	35.74
	NM_198141.ex.3	100	18.38
	NM_198141.ex.4	100	17.33
	NM_198141.ex.5	100	7.94
	NM_198141.ex.6	100	9.92
	NM_198141.ex.7	100	14.76
	NM_198141.ex.8	100	19.01
	NM_198141.ex.9	100	10.69
	NM_198141.ex.10	100	11.64
	NM_198141.ex.11	100	4.02
	NM_198141.ex.12	100	18.34

	NM_198141.ex.13	100	17.45
	NM_198141.ex.14	100	9.51
	NM_198141.ex.15	100	26.01
	NM_198141.ex.16	100	13.74
	NM_198141.ex.17	100	3.48
	NM_198141.ex.18	100	20.36
	NM_198141.ex.19	100	18.93
	NM_198141.ex.20	100	22.74
	NM_198141.ex.21	100	0.76
	NM_198141.ex.22	100	6.46
	NM_198141.ex.23	100	0.95
	NM_198141.ex.24	100	34.08
**GCDH**	NM_013976.ex.2	100	4.83
	NM_013976.ex.4	100	2.66
	NM_013976.ex.5	100	17.71
	NM_013976.ex.7	100	7.66
	NM_013976.ex.10	100	2.32
	NM_013976.ex.11	100	4.73
**HLCS**	NM_000411.ex.4	100	43.18
	NM_000411.ex.5	98.1	3.50
	NM_000411.ex.6	100	2.77
	NM_000411.ex.7	100	0.83
	NM_000411.ex.8	100	7.57
	NM_000411.ex.9	100	5.85
	NM_000411.ex.10	100	16.21
	NM_000411.ex.11	100	12.48
**HPRT1**	NM_000194.ex.1	100	17.07
	NM_000194.ex.2	100	23.85
	NM_000194.ex.3	100	17.72
	NM_000194.ex.4	100	20.37
	NM_000194.ex.5	100	12.73
	NM_000194.ex.6	100	13.26
	NM_000194.ex.7	100	11.51
	NM_000194.ex.8	100	20.67
	NM_000194.ex.9	100	27.47
**HSPG2**	NM_005529.ex.1	0	23.31
	NM_005529.ex.3	100	2.64
	NM_005529.ex.5	100	0.52
	NM_005529.ex.6	100	2.82
	NM_005529.ex.7	100	0.21
	NM_005529.ex.8	100	5.39
	NM_005529.ex.10	100	1.82
	NM_005529.ex.11	100	0.26
	NM_005529.ex.12	100	0.71
	NM_005529.ex.13	100	14.99
	NM_005529.ex.14	100	2.88
	NM_005529.ex.15	100	0.26
	NM_005529.ex.16	100	5.43
	NM_005529.ex.17	100	4.67
	NM_005529.ex.18	100	0.50
	NM_005529.ex.19	100	0.61
	NM_005529.ex.20	100	2.29
	NM_005529.ex.21	100	1.66
	NM_005529.ex.23	100	13.83
	NM_005529.ex.24	100	4.56
	NM_005529.ex.27	100	2.50
	NM_005529.ex.28	100	8.54
	NM_005529.ex.29	100	0.17
	NM_005529.ex.30	100	1.11
	NM_005529.ex.31	100	5.54
	NM_005529.ex.33	100	2.82
	NM_005529.ex.34	68.09	11.68
	NM_005529.ex.35	100	14.19
	NM_005529.ex.36	100	11.33
	NM_005529.ex.37	70.43	16.08
	NM_005529.ex.38	100	3.63

	NM_005529.ex.39	100	3.74
	NM_005529.ex.40	100	5.53
	NM_005529.ex.41	100	4.03
	NM_005529.ex.43	100	1.84
	NM_005529.ex.44	100	12.82
	NM_005529.ex.46	100	5.25
	NM_005529.ex.48	100	3.81
	NM_005529.ex.50	100	7.29
	NM_005529.ex.51	100	8.25
	NM_005529.ex.52	100	3.00
	NM_005529.ex.53	100	0.75
	NM_005529.ex.54	100	0.27
	NM_005529.ex.55	100	17.85
	NM_005529.ex.56	100	4.17
	NM_005529.ex.57	100	14.28
	NM_005529.ex.58	100	3.12
	NM_005529.ex.59	100	3.12
	NM_005529.ex.60	100	3.20
	NM_005529.ex.62	100	0.60
	NM_005529.ex.64	100	0.40
	NM_005529.ex.65	100	2.18
	NM_005529.ex.66	100	6.14
	NM_005529.ex.67	100	12.99
	NM_005529.ex.71	90.32	4.60
	NM_005529.ex.73	100	1.88
	NM_005529.ex.74	100	5.29
	NM_005529.ex.75	100	3.98
	NM_005529.ex.76	100	4.67
	NM_005529.ex.77	100	1.44
	NM_005529.ex.78	77.48	14.75
	NM_005529.ex.82	95.89	6.68
	NM_005529.ex.83	100	5.42
	NM_005529.ex.84	100	1.09
	NM_005529.ex.85	100	1.59
	NM_005529.ex.86	100	1.18
	NM_005529.ex.87	100	2.08
	NM_005529.ex.88	100	1.11
	NM_005529.ex.89	100	4.44
	NM_005529.ex.90	100	2.92
	NM_005529.ex.92	100	3.52
	NM_005529.ex.94	100	5.18
	NM_005529.ex.95	100	2.25
	NM_005529.ex.96	100	6.07
	NM_005529.ex.97	29.86	10.71
**HUWE1**	NM_031407.ex.4	100	20.20
	NM_031407.ex.5	100	13.79
	NM_031407.ex.6	100	10.37
	NM_031407.ex.7	48.05	33.74
	NM_031407.ex.8	100	1.28
	NM_031407.ex.9	100	13.78
	NM_031407.ex.10	100	46.17
	NM_031407.ex.11	100	13.48
	NM_031407.ex.12	100	25.93
	NM_031407.ex.13	100	20.22
	NM_031407.ex.14	100	9.31
	NM_031407.ex.15	100	10.70
	NM_031407.ex.16	100	32.39
	NM_031407.ex.17	100	37.26
	NM_031407.ex.18	100	5.42
	NM_031407.ex.19	100	15.81
	NM_031407.ex.20	100	14.12
	NM_031407.ex.21	100	12.54
	NM_031407.ex.22	100	34.56
	NM_031407.ex.23	100	16.21
	NM_031407.ex.24	100	25.00
	NM_031407.ex.25	50.56	33.78

	NM_031407.ex.26	100	23.46
	NM_031407.ex.27	100	10.93
	NM_031407.ex.28	100	11.68
	NM_031407.ex.29	96.89	18.49
	NM_031407.ex.30	100	10.89
	NM_031407.ex.31	84.68	11.50
	NM_031407.ex.32	100	12.18
	NM_031407.ex.33	100	15.45
	NM_031407.ex.34	100	18.13
	NM_031407.ex.35	100	11.15
	NM_031407.ex.36	89.3	12.45
	NM_031407.ex.37	100	13.26
	NM_031407.ex.38	100	12.81
	NM_031407.ex.39	100	12.52
	NM_031407.ex.40	68.54	28.36
	NM_031407.ex.41	100	19.77
	NM_031407.ex.42	100	6.91
	NM_031407.ex.43	100	4.54
	NM_031407.ex.44	86.98	32.60
	NM_031407.ex.45	100	24.48
	NM_031407.ex.46	100	18.29
	NM_031407.ex.47	100	4.43
	NM_031407.ex.48	100	12.59
	NM_031407.ex.49	98.37	10.21
	NM_031407.ex.50	100	9.69
	NM_031407.ex.51	100	5.71
	NM_031407.ex.52	100	7.88
	NM_031407.ex.53	100	9.97
	NM_031407.ex.54	100	13.57
	NM_031407.ex.55	100	9.67
	NM_031407.ex.56	100	5.62
	NM_031407.ex.57	100	5.03
	NM_031407.ex.58	100	10.82
	NM_031407.ex.59	100	8.20
	NM_031407.ex.60	100	14.39
	NM_031407.ex.61	100	15.26
	NM_031407.ex.62	100	3.59
	NM_031407.ex.63	91.67	26.98
	NM_031407.ex.64	100	9.34
	NM_031407.ex.65	100	10.96
	NM_031407.ex.66	100	4.35
	NM_031407.ex.67	100	9.31
	NM_031407.ex.68	92.86	21.65
	NM_031407.ex.69	100	4.86
	NM_031407.ex.70	100	22.33
	NM_031407.ex.71	100	13.23
	NM_031407.ex.72	100	8.49
	NM_031407.ex.73	77.52	30.99
	NM_031407.ex.74	100	14.22
	NM_031407.ex.75	100	10.37
	NM_031407.ex.76	100	11.98
	NM_031407.ex.77	100	11.53
	NM_031407.ex.78	100	8.92
	NM_031407.ex.79	100	3.49
	NM_031407.ex.80	100	18.91
	NM_031407.ex.81	100	16.55
	NM_031407.ex.82	100	6.67
	NM_031407.ex.83	100	9.52
**IGSF1**	NM_001170962.ex.2	100	6.86
	NM_001170962.ex.3	100	7.42
	NM_001170962.ex.4	100	4.33
	NM_001170962.ex.5	100	9.95
	NM_001170962.ex.6	98.31	11.22
	NM_001170962.ex.7	97.86	4.78
	NM_001170962.ex.8	100	2.88
	NM_001170962.ex.9	100	5.54

	NM_001170962.ex.11	100	2.54
	NM_001170962.ex.12	100	11.26
	NM_001170962.ex.13	100	0.86
	NM_001170962.ex.14	100	13.28
	NM_001170962.ex.15	100	15.60
	NM_001170962.ex.16	100	9.63
	NM_001170962.ex.17	100	9.92
	NM_001170962.ex.18	100	0.72
	NM_001170962.ex.19	100	20.81
	NM_001170963.ex.3	100	5.41
	NM_001170963.ex.5	100	21.35
**IL1RAPL1**	NM_014271.ex.2	100	29.22
	NM_014271.ex.3	100	16.33
	NM_014271.ex.4	100	24.37
	NM_014271.ex.5	100	21.40
	NM_014271.ex.6	100	28.70
	NM_014271.ex.7	100	29.40
	NM_014271.ex.8	74.15	15.64
	NM_014271.ex.9	100	7.73
	NM_014271.ex.10	100	30.13
	NM_014271.ex.11	100	3.86
**IL6**	NM_000600.ex.2	80.73	29.86
	NM_000600.ex.3	100	17.58
	NM_000600.ex.4	91.89	15.75
	NM_000600.ex.5	100	15.59
**INHBB**	NM_002193.ex.1	89.09	6.40
	NM_002193.ex.2	100	35.63
**IQSEC2**	NM_001111125.ex.1	91.75	13.71
	NM_001243197.ex.1	100	24.73
	NM_001243197.ex.2	100	4.88
	NM_001243197.ex.3	100	28.02
	NM_015075.ex.3	100	2.93
	NM_015075.ex.4	100	5.60
	NM_015075.ex.5	100	4.98
	NM_015075.ex.6	100	4.87
	NM_015075.ex.7	100	5.00
	NM_015075.ex.8	100	13.75
	NM_015075.ex.9	100	21.94
	NM_015075.ex.10	100	4.20
	NM_015075.ex.11	100	29.83
	NM_015075.ex.12	100	10.21
	NM_015075.ex.13	100	1.47
	NM_015075.ex.14	49.18	11.96
**JHDM1D**	NM_030647.ex.1	100	5.43
	NM_030647.ex.2	100	3.67
	NM_030647.ex.3	100	10.25
	NM_030647.ex.4	100	17.29
	NM_030647.ex.5	100	17.75
	NM_030647.ex.6	100	15.09
	NM_030647.ex.7	100	18.91
	NM_030647.ex.8	85.39	32.33
	NM_030647.ex.9	100	19.87
	NM_030647.ex.10	100	5.00
	NM_030647.ex.11	100	19.37
	NM_030647.ex.12	100	6.25
	NM_030647.ex.13	100	3.18
	NM_030647.ex.14	100	18.86
	NM_030647.ex.15	100	24.57
	NM_030647.ex.16	99.33	24.94
	NM_030647.ex.17	100	12.34
	NM_030647.ex.18	100	31.10
	NM_030647.ex.19	100	5.36
	NM_030647.ex.20	2.76	47.01
**KANK1**	NM_001256876.ex.6	100	6.63
	NM_153186.ex.2	97.9	2.80
	NM_153186.ex.3	100	2.19

	NM_153186.ex.4	100	14.55
	NM_153186.ex.5	100	1.28
	NM_153186.ex.6	93.26	9.66
	NM_153186.ex.7	100	4.73
	NM_153186.ex.8	100	6.05
	NM_153186.ex.9	100	4.49
	NM_153186.ex.10	100	23.55
	NM_153186.ex.11	100	12.75
**KDM5C**	NM_001146702.ex.22	100	16.86
	NM_001146702.ex.23	100	6.77
	NM_001146702.ex.24	100	21.48
	NM_004187.ex.1	100	7.88
	NM_004187.ex.2	100	0.55
	NM_004187.ex.3	100	12.00
	NM_004187.ex.4	100	7.57
	NM_004187.ex.5	100	16.29
	NM_004187.ex.6	100	8.97
	NM_004187.ex.7	82.51	19.97
	NM_004187.ex.8	100	11.80
	NM_004187.ex.9	100	11.31
	NM_004187.ex.10	100	3.65
	NM_004187.ex.11	100	1.51
	NM_004187.ex.12	100	22.90
	NM_004187.ex.13	95.87	17.92
	NM_004187.ex.14	99.49	11.00
	NM_004187.ex.15	100	4.14
	NM_004187.ex.16	100	6.07
	NM_004187.ex.17	100	7.92
	NM_004187.ex.18	100	0.13
	NM_004187.ex.19	100	0.19
	NM_004187.ex.20	100	5.56
	NM_004187.ex.21	91.71	11.90
	NM_004187.ex.22	100	3.07

	NM_004187.ex.23	100	0.81
	NM_004187.ex.24	100	16.16
	NM_004187.ex.25	100	4.80
	NM_004187.ex.26	100	42.37
**KIF1A**	NM_001244008.ex.14	100	19.50
	NM_001244008.ex.27	100	8.79
	NM_004321.ex.1	100	0.51
	NM_004321.ex.2	100	2.09
	NM_004321.ex.3	100	2.52
	NM_004321.ex.5	100	3.75
	NM_004321.ex.6	100	0.14
	NM_004321.ex.9	100	13.15
	NM_004321.ex.10	100	10.76
	NM_004321.ex.11	100	5.20
	NM_004321.ex.12	100	6.17
	NM_004321.ex.15	100	2.67
	NM_004321.ex.17	100	5.25
	NM_004321.ex.19	100	12.47
	NM_004321.ex.20	100	14.10
	NM_004321.ex.21	100	1.93
	NM_004321.ex.22	100	0.77
	NM_004321.ex.23	100	0.77
	NM_004321.ex.24	100	2.88
	NM_004321.ex.25	100	0.11
	NM_004321.ex.27	100	2.38
	NM_004321.ex.28	100	0.34
	NM_004321.ex.29	100	7.20
	NM_004321.ex.31	100	0.47
	NM_004321.ex.32	100	2.33
	NM_004321.ex.33	100	6.57
	NM_004321.ex.35	100	0.30
	NM_004321.ex.39	100	2.78
	NM_004321.ex.40	100	8.08

	NM_004321.ex.42	100	0.09
	NM_004321.ex.43	100	1.46
	NM_004321.ex.44	100	2.27
	NM_004321.ex.45	100	0.08
	NM_004321.ex.46	100	17.43
**KIF1C**	NM_006612.ex.3	100	1.14
	NM_006612.ex.4	100	1.08
	NM_006612.ex.5	100	3.11
	NM_006612.ex.6	100	10.15
	NM_006612.ex.8	100	5.78
	NM_006612.ex.9	100	2.81
	NM_006612.ex.10	100	4.81
	NM_006612.ex.11	100	7.23
	NM_006612.ex.12	100	12.69
	NM_006612.ex.13	100	4.02
	NM_006612.ex.14	100	0.80
	NM_006612.ex.15	100	8.02
	NM_006612.ex.16	100	1.76
	NM_006612.ex.17	100	8.17
	NM_006612.ex.19	100	4.13
	NM_006612.ex.20	81.38	14.90
	NM_006612.ex.21	100	6.74
	NM_006612.ex.22	100	4.41
	NM_006612.ex.23	100	33.03
**L1CAM**	NM_000425.ex.27	69.23	35.18
	NM_024003.ex.1	100	19.24
	NM_024003.ex.2	100	4.20
	NM_024003.ex.3	100	21.01
	NM_024003.ex.4	100	2.01
	NM_024003.ex.5	100	6.91
	NM_024003.ex.6	100	5.47
	NM_024003.ex.7	100	7.30
	NM_024003.ex.8	100	11.22
	NM_024003.ex.9	100	1.26
	NM_024003.ex.10	100	2.99
	NM_024003.ex.11	100	1.73
	NM_024003.ex.12	100	7.85
	NM_024003.ex.13	100	2.45
	NM_024003.ex.14	100	4.17
	NM_024003.ex.15	100	0.56
	NM_024003.ex.16	100	2.63
	NM_024003.ex.18	100	4.15
	NM_024003.ex.19	100	12.49
	NM_024003.ex.20	100	7.21
	NM_024003.ex.21	100	0.39
	NM_024003.ex.22	100	0.62
	NM_024003.ex.23	90.08	17.00
	NM_024003.ex.25	99.26	9.47
	NM_024003.ex.26	100	12.20
	NM_024003.ex.27	100	16.66
**LCP2**	NM_005565.ex.1	100	6.96
	NM_005565.ex.2	100	4.64
	NM_005565.ex.3	100	7.07
	NM_005565.ex.5	100	2.95
	NM_005565.ex.7	100	1.56
	NM_005565.ex.8	100	19.01
	NM_005565.ex.9	17.65	48.24
	NM_005565.ex.10	100	20.93
	NM_005565.ex.11	100	14.11
	NM_005565.ex.12	100	7.64
	NM_005565.ex.13	100	4.91
	NM_005565.ex.15	100	3.48
	NM_005565.ex.16	100	1.59
	NM_005565.ex.17	100	4.51
	NM_005565.ex.18	100	15.72
	NM_005565.ex.19	100	10.09

	NM_005565.ex.20	100	5.67
	NM_005565.ex.21	100	27.74
**MAN2A1**	NM_002372.ex.1	100	13.87
	NM_002372.ex.2	100	10.53
	NM_002372.ex.3	100	11.82
	NM_002372.ex.4	100	12.71
	NM_002372.ex.5	100	14.26
	NM_002372.ex.6	100	11.62
	NM_002372.ex.7	100	27.38
	NM_002372.ex.8	100	13.92
	NM_002372.ex.9	100	17.33
	NM_002372.ex.10	100	24.95
	NM_002372.ex.11	100	14.42
	NM_002372.ex.12	100	26.03
	NM_002372.ex.13	100	12.13
	NM_002372.ex.14	100	23.51
	NM_002372.ex.15	100	12.40
	NM_002372.ex.16	70.69	39.23
	NM_002372.ex.17	60	20.94
	NM_002372.ex.18	100	15.71
	NM_002372.ex.19	100	31.88
	NM_002372.ex.20	100	24.36
	NM_002372.ex.21	100	2.59
	NM_002372.ex.22	100	38.47
**MAOB**	NM_000898.ex.1	100	9.34
	NM_000898.ex.2	100	14.28
	NM_000898.ex.3	100	17.13
	NM_000898.ex.4	100	9.65
	NM_000898.ex.5	100	27.68
	NM_000898.ex.6	100	17.51
	NM_000898.ex.7	100	13.85
	NM_000898.ex.8	100	24.71
	NM_000898.ex.9	52.04	45.97
	NM_000898.ex.10	100	9.23
	NM_000898.ex.11	100	15.41
	NM_000898.ex.12	100	2.48
	NM_000898.ex.13	100	16.85
	NM_000898.ex.14	100	9.35
	NM_000898.ex.15	100	25.64
**MAST1**	NM_014975.ex.1	100	5.53
	NM_014975.ex.2	100	15.34
	NM_014975.ex.3	100	15.32
	NM_014975.ex.6	100	26.94
	NM_014975.ex.7	100	4.86
	NM_014975.ex.8	100	9.29
	NM_014975.ex.9	100	4.34
	NM_014975.ex.10	100	12.06
	NM_014975.ex.11	100	2.69
	NM_014975.ex.15	100	11.00
	NM_014975.ex.17	100	0.31
	NM_014975.ex.18	100	2.20
	NM_014975.ex.19	100	4.51
	NM_014975.ex.20	100	1.89
	NM_014975.ex.21	100	4.34
	NM_014975.ex.22	100	4.66
	NM_014975.ex.23	100	0.28
	NM_014975.ex.24	100	5.00
	NM_014975.ex.25	100	0.94
	NM_014975.ex.26	98.29	4.61
**MC2R**	NM_000529.ex.2	100	29.39
**MCCC1**	NM_020166.ex.1	100	15.24
	NM_020166.ex.2	100	18.97
	NM_020166.ex.3	100	20.14
	NM_020166.ex.4	100	10.78
	NM_020166.ex.5	100	7.42
	NM_020166.ex.6	85.91	10.38

	NM_020166.ex.7	100	11.91
	NM_020166.ex.8	100	0.34
	NM_020166.ex.9	100	4.29
	NM_020166.ex.10	100	5.73
	NM_020166.ex.11	100	13.14
	NM_020166.ex.12	100	3.40
	NM_020166.ex.13	100	2.63
	NM_020166.ex.14	87.5	36.48
	NM_020166.ex.15	100	26.47
	NM_020166.ex.16	100	18.35
	NM_020166.ex.17	100	26.01
	NM_020166.ex.18	100	33.14
	NM_020166.ex.19	100	26.12
**MCPH1**	NM_001172574.ex.1	100	8.90
	NM_001172574.ex.2	100	10.92
	NM_001172574.ex.3	100	6.81
	NM_001172574.ex.4	100	7.28
	NM_001172574.ex.5	88.79	16.16
	NM_001172574.ex.6	84.14	14.66
	NM_001172574.ex.7	100	21.32
	NM_001172574.ex.8	97.84	13.25
	NM_024596.ex.8	97.84	13.25
	NM_024596.ex.9	100	21.18
	NM_024596.ex.10	100	20.52
	NM_024596.ex.11	100	4.35
	NM_024596.ex.14	100	33.66
**MECP2**	NM_001110792.ex.1	100	10.06
	NM_001110792.ex.2	100	16.43
	NM_001110792.ex.3	87.26	8.74
	NM_004992.ex.2	100	2.06
	NM_004992.ex.4	12.54	2.05
**MGA**	NM_001080541.ex.2	95.32	13.85
	NM_001080541.ex.3	96.53	21.84
	NM_001080541.ex.4	100	13.01
	NM_001080541.ex.5	100	42.62
	NM_001080541.ex.6	100	28.87
	NM_001080541.ex.7	100	9.76
	NM_001080541.ex.8	92.88	22.91
	NM_001080541.ex.9	100	11.25
	NM_001080541.ex.10	100	17.63
	NM_001080541.ex.11	66.84	20.23
	NM_001080541.ex.12	100	9.10
	NM_001080541.ex.13	100	13.55
	NM_001080541.ex.14	100	0.77
	NM_001080541.ex.15	100	10.89
	NM_001080541.ex.16	93.09	10.29
	NM_001080541.ex.17	100	12.64
	NM_001080541.ex.18	100	24.34
	NM_001080541.ex.19	100	12.78
	NM_001080541.ex.20	100	16.62
	NM_001080541.ex.21	100	9.61
	NM_001080541.ex.22	100	15.13
	NM_001080541.ex.23	100	31.40
	NM_001164273.ex.15	100	6.17
**MIIP**	NM_021933.ex.2	100	3.71
	NM_021933.ex.3	100	0.04
	NM_021933.ex.4	100	0.19
	NM_021933.ex.6	100	13.43
	NM_021933.ex.9	100	1.54
	NM_021933.ex.10	90.88	18.66
**MTMR1**	NM_003828.ex.1	83.69	8.61
	NM_003828.ex.2	100	27.46
	NM_003828.ex.3	100	8.46
	NM_003828.ex.4	25	41.62
	NM_003828.ex.5	100	25.02
	NM_003828.ex.6	100	17.80

	NM_003828.ex.7	100	30.69
	NM_003828.ex.8	67.55	41.48
	NM_003828.ex.9	100	25.51
	NM_003828.ex.10	100	8.11
	NM_003828.ex.11	100	11.79
	NM_003828.ex.12	100	16.00
	NM_003828.ex.13	100	26.53
	NM_003828.ex.14	100	29.35
	NM_003828.ex.15	100	6.52
**MYO15A**	NM_016239.ex.2	95.38	5.12
	NM_016239.ex.3	100	3.25
	NM_016239.ex.5	100	0.83
	NM_016239.ex.7	100	2.97
	NM_016239.ex.8	100	6.70
	NM_016239.ex.10	100	2.71
	NM_016239.ex.11	100	6.80
	NM_016239.ex.12	100	1.16
	NM_016239.ex.15	100	6.94
	NM_016239.ex.16	100	10.68
	NM_016239.ex.18	100	5.55
	NM_016239.ex.19	100	10.05
	NM_016239.ex.20	100	9.16
	NM_016239.ex.21	100	5.43
	NM_016239.ex.22	100	5.57
	NM_016239.ex.23	100	12.20
	NM_016239.ex.25	100	0.10
	NM_016239.ex.26	100	5.25
	NM_016239.ex.27	100	4.07
	NM_016239.ex.28	100	16.51
	NM_016239.ex.29	100	0.37
	NM_016239.ex.30	100	0.89
	NM_016239.ex.31	100	18.12
	NM_016239.ex.32	100	1.91
	NM_016239.ex.33	100	2.68
	NM_016239.ex.34	100	14.90
	NM_016239.ex.35	100	0.64
	NM_016239.ex.36	100	5.99
	NM_016239.ex.39	100	8.46
	NM_016239.ex.40	100	0.66
	NM_016239.ex.41	100	3.47
	NM_016239.ex.42	100	5.27
	NM_016239.ex.43	100	14.53
	NM_016239.ex.45	100	3.41
	NM_016239.ex.47	100	1.12
	NM_016239.ex.48	100	6.87
	NM_016239.ex.49	100	12.45
	NM_016239.ex.50	100	10.02
	NM_016239.ex.51	100	6.80
	NM_016239.ex.52	100	4.78
	NM_016239.ex.53	100	1.89
	NM_016239.ex.54	100	9.58
	NM_016239.ex.55	100	8.07
	NM_016239.ex.57	100	1.35
	NM_016239.ex.58	100	6.78
	NM_016239.ex.60	100	3.50
	NM_016239.ex.61	100	3.85
	NM_016239.ex.63	100	1.24
	NM_016239.ex.64	100	0.23
**MYO1F**	NM_012335.ex.1	100	7.69
	NM_012335.ex.2	100	1.61
	NM_012335.ex.3	100	1.19
	NM_012335.ex.4	100	4.71
	NM_012335.ex.6	100	3.40
	NM_012335.ex.7	100	22.04
	NM_012335.ex.9	100	0.32
	NM_012335.ex.11	100	5.30

	NM_012335.ex.12	100	1.10
	NM_012335.ex.14	100	4.07
	NM_012335.ex.15	100	33.45
	NM_012335.ex.17	100	11.00
	NM_012335.ex.18	100	3.25
	NM_012335.ex.19	100	0.18
	NM_012335.ex.20	100	9.26
	NM_012335.ex.21	100	9.11
	NM_012335.ex.22	100	2.17
	NM_012335.ex.23	88.51	10.70
	NM_012335.ex.25	100	8.42
	NM_012335.ex.26	100	4.28
	NM_012335.ex.27	100	4.10
	NM_012335.ex.28	100	15.21
**NAA35**	NM_024635.ex.2	100	9.72
	NM_024635.ex.3	94.29	45.80
	NM_024635.ex.4	100	12.40
	NM_024635.ex.5	100	24.59
	NM_024635.ex.6	100	19.07
	NM_024635.ex.7	100	29.21
	NM_024635.ex.8	100	21.62
	NM_024635.ex.9	100	10.77
	NM_024635.ex.10	100	13.87
	NM_024635.ex.11	100	12.00
	NM_024635.ex.12	84.44	22.55
	NM_024635.ex.13	77.05	46.76
	NM_024635.ex.14	100	26.57
	NM_024635.ex.16	100	37.00
	NM_024635.ex.17	100	7.17
	NM_024635.ex.18	100	17.66
	NM_024635.ex.19	100	7.99
	NM_024635.ex.20	100	10.05
	NM_024635.ex.21	100	23.72
	NM_024635.ex.22	100	39.38
	NM_024635.ex.23	100	28.37
**NEMF**	NM_004713.ex.1	100	2.58
	NM_004713.ex.2	100	26.97
	NM_004713.ex.3	100	11.87
	NM_004713.ex.4	100	23.04
	NM_004713.ex.5	100	21.27
	NM_004713.ex.6	100	7.25
	NM_004713.ex.7	100	5.70
	NM_004713.ex.8	100	18.32
	NM_004713.ex.9	100	11.08
	NM_004713.ex.10	100	38.83
	NM_004713.ex.11	81.25	39.27
	NM_004713.ex.12	100	15.08
	NM_004713.ex.13	100	11.64
	NM_004713.ex.14	100	5.29
	NM_004713.ex.15	100	7.82
	NM_004713.ex.16	23.33	27.46
	NM_004713.ex.17	100	19.19
	NM_004713.ex.18	100	10.98
	NM_004713.ex.19	100	2.42
	NM_004713.ex.20	62.71	36.73
	NM_004713.ex.21	100	10.11
	NM_004713.ex.22	100	17.18
	NM_004713.ex.23	100	13.84
	NM_004713.ex.24	100	15.37
	NM_004713.ex.25	100	21.88
	NM_004713.ex.26	100	16.60
	NM_004713.ex.27	51.9	38.69
	NM_004713.ex.28	100	6.28
	NM_004713.ex.29	100	12.06
	NM_004713.ex.30	100	10.58
	NM_004713.ex.31	100	40.61

	NM_004713.ex.32	100	10.90
	NM_004713.ex.33	100	31.13
**NGFR**	NM_002507.ex.1	93.23	11.45
	NM_002507.ex.2	100	18.39
	NM_002507.ex.3	97.23	1.61
	NM_002507.ex.4	100	12.52
	NM_002507.ex.6	100	17.41
**NPC1**	NM_000271.ex.1	100	27.33
	NM_000271.ex.2	100	8.48
	NM_000271.ex.3	100	6.54
	NM_000271.ex.4	100	9.22
	NM_000271.ex.5	87.57	7.56
	NM_000271.ex.6	100	2.37
	NM_000271.ex.7	100	7.25
	NM_000271.ex.8	100	3.84
	NM_000271.ex.9	100	10.92
	NM_000271.ex.10	100	9.24
	NM_000271.ex.11	100	8.78
	NM_000271.ex.12	98.95	17.35
	NM_000271.ex.13	100	0.67
	NM_000271.ex.14	100	5.39
	NM_000271.ex.15	100	5.64
	NM_000271.ex.17	100	38.37
	NM_000271.ex.18	100	7.72
	NM_000271.ex.19	100	29.90
	NM_000271.ex.20	100	8.63
	NM_000271.ex.21	100	5.07
	NM_000271.ex.24	100	18.62
**NR1I2**	NM_022002.ex.1	100	6.11
	NM_022002.ex.2	100	1.50
	NM_022002.ex.3	100	1.23
	NM_022002.ex.4	100	1.97
	NM_022002.ex.5	100	10.14

	NM_022002.ex.6	100	3.23
	NM_022002.ex.7	100	0.50
	NM_022002.ex.8	100	1.89
	NM_022002.ex.9	100	11.91
	NM_033013.ex.5	100	3.58
**NT5C2**	NM_012229.ex.3	100	7.85
	NM_012229.ex.4	100	23.53
	NM_012229.ex.5	100	6.45
	NM_012229.ex.6	100	10.80
	NM_012229.ex.7	100	36.42
	NM_012229.ex.8	100	30.51
	NM_012229.ex.9	100	13.83
	NM_012229.ex.10	100	8.37
	NM_012229.ex.11	100	10.96
	NM_012229.ex.12	100	30.50
	NM_012229.ex.13	100	1.13
	NM_012229.ex.14	100	4.61
	NM_012229.ex.15	100	15.38
	NM_012229.ex.16	100	6.09
	NM_012229.ex.17	100	27.94
	NM_012229.ex.18	100	4.84
	NM_012229.ex.19	22.03	11.56
**ODZ1**	NM_001163278.ex.6	100	7.48
	NM_001163279.ex.1	100	6.56
	NM_001163279.ex.2	100	6.04
	NM_001163279.ex.3	100	15.90
	NM_001163279.ex.4	100	31.74
	NM_001163279.ex.5	100	6.58
	NM_001163279.ex.6	100	7.48
	NM_001163279.ex.7	99	17.46
	NM_001163279.ex.8	100	7.26
	NM_001163279.ex.9	100	6.51
	NM_001163279.ex.10	100	14.26

	NM_001163279.ex.11	100	4.38
	NM_001163279.ex.12	100	5.75
	NM_001163279.ex.13	100	42.38
	NM_001163279.ex.15	89.91	9.55
	NM_001163279.ex.16	100	7.98
	NM_001163279.ex.17	100	5.74
	NM_001163279.ex.18	100	4.16
	NM_001163279.ex.19	100	23.92
	NM_001163279.ex.20	100	16.28
	NM_001163279.ex.21	100	22.99
	NM_001163279.ex.22	65.38	32.68
	NM_001163279.ex.23	100	7.31
	NM_001163279.ex.24	100	9.27
	NM_001163279.ex.25	97.27	18.12
	NM_001163279.ex.26	100	28.65
	NM_001163279.ex.27	100	10.49
	NM_001163279.ex.28	100	8.99
	NM_001163279.ex.29	100	17.12
	NM_001163279.ex.30	95.34	13.83
	NM_001163279.ex.31	100	16.61
	NM_001163279.ex.32	100	37.34
**OFD1**	NM_003611.ex.1	100	7.23
	NM_003611.ex.2	100	13.91
	NM_003611.ex.3	100	11.38
	NM_003611.ex.4	100	23.10
	NM_003611.ex.5	100	36.19
	NM_003611.ex.6	100	21.39
	NM_003611.ex.7	97.1	22.96
	NM_003611.ex.8	100	15.60
	NM_003611.ex.9	100	19.78
	NM_003611.ex.10	100	22.66
	NM_003611.ex.11	100	3.32
	NM_003611.ex.12	100	40.65
	NM_003611.ex.13	61.78	26.45
	NM_003611.ex.14	96.97	21.47
	NM_003611.ex.15	100	26.57
	NM_003611.ex.16	100	12.61
	NM_003611.ex.17	73.44	37.24
	NM_003611.ex.18	100	17.29
	NM_003611.ex.19	100	1.44
	NM_003611.ex.20	66.67	32.44
	NM_003611.ex.21	96.51	37.17
	NM_003611.ex.22	100	18.46
	NM_003611.ex.23	100	35.24
**PAFAH1B1**	NM_000430.ex.2	100	6.00
	NM_000430.ex.3	100	28.71
	NM_000430.ex.4	100	37.08
	NM_000430.ex.5	88.46	14.25
	NM_000430.ex.6	100	16.03
	NM_000430.ex.7	100	9.64
	NM_000430.ex.8	100	13.69
	NM_000430.ex.9	100	10.96
	NM_000430.ex.10	100	22.60
	NM_000430.ex.11	100	10.56
**PAK3**	NM_001128172.ex.3	100	33.78
	NM_001128173.ex.1	100	17.08
	NM_001128173.ex.2	100	16.31
	NM_001128173.ex.3	100	33.69
	NM_001128173.ex.4	100	6.32
	NM_001128173.ex.5	100	27.67
	NM_001128173.ex.6	100	29.24
	NM_001128173.ex.7	100	13.75
	NM_001128173.ex.8	100	18.80
	NM_001128173.ex.9	100	23.51
	NM_001128173.ex.10	100	41.22
	NM_001128173.ex.11	100	4.73

	NM_001128173.ex.12	100	1.73
	NM_001128173.ex.13	100	8.30
	NM_001128173.ex.14	100	15.26
	NM_001128173.ex.15	100	34.82
**PCBP3**	NM_001130141.ex.1	0	44.71
	NM_001130141.ex.2	100	1.10
	NM_001130141.ex.3	100	5.41
	NM_001130141.ex.4	100	8.79
	NM_001130141.ex.5	100	7.58
	NM_001130141.ex.6	100	8.55
	NM_001130141.ex.7	100	0.04
	NM_001130141.ex.9	100	0.70
	NM_001130141.ex.11	100	7.68
	NM_001130141.ex.12	100	2.29
	NM_020528.ex.10	100	0.88
**PHF17**	NM_024900.ex.2	100	19.32
	NM_024900.ex.3	100	1.43
	NM_024900.ex.4	100	8.69
	NM_024900.ex.5	100	6.07
	NM_024900.ex.6	100	7.41
	NM_024900.ex.7	100	6.84
	NM_024900.ex.8	100	1.19
	NM_024900.ex.9	100	29.79
	NM_199320.ex.9	100	22.32
	NM_199320.ex.10	100	4.96
	NM_199320.ex.11	100	29.70
**PLA2G6**	NM_003560.ex.2	100	3.74
	NM_003560.ex.3	100	8.88
	NM_003560.ex.5	100	11.17
	NM_003560.ex.6	100	3.50
	NM_003560.ex.7	100	0.50
	NM_003560.ex.8	100	12.28
	NM_003560.ex.10	100	0.14
	NM_003560.ex.11	100	1.51
	NM_003560.ex.12	100	1.22
	NM_003560.ex.13	100	0.35
	NM_003560.ex.14	100	19.11
	NM_003560.ex.15	100	0.74
	NM_003560.ex.16	100	4.26
**PLP1**	NM_000533.ex.3	100	17.72
	NM_001128834.ex.2	100	20.84
	NM_199478.ex.1	100	21.80
	NM_199478.ex.2	100	3.51
	NM_199478.ex.3	100	17.72
	NM_199478.ex.4	78.82	31.87
	NM_199478.ex.5	100	1.56
	NM_199478.ex.6	100	9.14
	NM_199478.ex.7	100	45.92
**PROC**	NM_000312.ex.2	100	2.69
	NM_000312.ex.5	100	1.61
	NM_000312.ex.6	100	10.31
	NM_000312.ex.7	100	1.31
	NM_000312.ex.8	100	8.52
	NM_000312.ex.9	100	3.86
**PTGFRN**	NM_020440.ex.1	100	10.07
	NM_020440.ex.2	100	1.52
	NM_020440.ex.3	100	1.21
	NM_020440.ex.4	100	2.30
	NM_020440.ex.5	100	2.69
	NM_020440.ex.6	100	2.41
	NM_020440.ex.7	100	10.66
	NM_020440.ex.8	100	1.30
**RAD21**	NM_006265.ex.2	100	13.78
	NM_006265.ex.3	83.97	16.93
	NM_006265.ex.4	100	9.09
	NM_006265.ex.5	100	19.54

	NM_006265.ex.6	80.77	23.59
	NM_006265.ex.7	82.68	24.48
	NM_006265.ex.8	100	12.23
	NM_006265.ex.9	100	6.00
	NM_006265.ex.10	100	13.81
	NM_006265.ex.11	100	7.43
	NM_006265.ex.12	100	15.00
	NM_006265.ex.13	100	24.42
	NM_006265.ex.14	20.01	4.82
**RFX2**	NM_000635.ex.6	100	19.51
	NM_134433.ex.2	100	0.93
	NM_134433.ex.3	100	1.37
	NM_134433.ex.5	100	3.08
	NM_134433.ex.6	100	2.69
	NM_134433.ex.7	100	5.06
	NM_134433.ex.8	100	3.65
	NM_134433.ex.9	100	0.99
	NM_134433.ex.10	100	0.14
	NM_134433.ex.11	100	8.40
	NM_134433.ex.12	100	0.82
	NM_134433.ex.14	100	7.49
	NM_134433.ex.15	100	3.26
	NM_134433.ex.17	100	13.16
**RNF214**	NM_001077239.ex.2	75.44	5.05
	NM_001077239.ex.3	97.07	9.48
	NM_001077239.ex.4	100	11.31
	NM_001077239.ex.5	100	8.39
	NM_001077239.ex.6	100	0.84
	NM_001077239.ex.7	100	5.73
	NM_001077239.ex.8	100	7.64
	NM_001077239.ex.9	100	20.24
	NM_001077239.ex.10	100	12.07
	NM_001077239.ex.11	100	3.64
	NM_001077239.ex.12	100	0.30
	NM_001077239.ex.13	100	5.81
	NM_001077239.ex.14	100	17.74
	NM_001077239.ex.15	100	22.77
**SCN8A**	NM_014191.ex.2	100	1.49
	NM_014191.ex.3	100	16.17
	NM_014191.ex.4	100	3.26
	NM_014191.ex.5	100	5.17
	NM_014191.ex.6	100	6.16
	NM_014191.ex.7	100	1.35
	NM_014191.ex.8	100	14.69
	NM_014191.ex.9	100	5.63
	NM_014191.ex.10	100	4.61
	NM_014191.ex.11	100	10.68
	NM_014191.ex.12	100	0.06
	NM_014191.ex.13	73.13	8.32
	NM_014191.ex.14	100	8.92
	NM_014191.ex.15	100	9.63
	NM_014191.ex.16	99.72	11.98
	NM_014191.ex.17	100	0.52
	NM_014191.ex.19	97.44	3.63
	NM_014191.ex.20	68.57	15.81
	NM_014191.ex.21	100	7.81
	NM_014191.ex.22	100	7.24
	NM_014191.ex.23	100	1.38
	NM_014191.ex.24	100	12.71
	NM_014191.ex.25	100	2.27
	NM_014191.ex.26	98.53	15.11
	NM_014191.ex.27	100	40.64
**SLC16A2**	NM_006517.ex.1	100	14.13
	NM_006517.ex.3	99.56	4.90
	NM_006517.ex.4	100	6.29
	NM_006517.ex.5	100	13.04

	NM_006517.ex.6	100	19.38
**SLC6A3**	NM_001044.ex.2	100	9.62
	NM_001044.ex.3	100	0.83
	NM_001044.ex.7	100	0.10
	NM_001044.ex.9	100	2.58
	NM_001044.ex.10	100	5.12
	NM_001044.ex.11	100	1.73
	NM_001044.ex.12	100	1.59
	NM_001044.ex.14	100	8.14
	NM_001044.ex.15	100	11.86
**SPAST**	NM_014946.ex.1	80.85	6.98
	NM_014946.ex.2	100	28.70
	NM_014946.ex.3	100	20.04
	NM_014946.ex.4	100	23.46
	NM_014946.ex.5	100	9.36
	NM_014946.ex.6	100	23.43
	NM_014946.ex.7	100	18.56
	NM_014946.ex.8	100	5.22
	NM_014946.ex.9	100	17.65
	NM_014946.ex.10	100	25.49
	NM_014946.ex.11	100	23.66
	NM_014946.ex.12	100	14.90
	NM_014946.ex.13	100	37.30
	NM_014946.ex.14	100	26.31
	NM_014946.ex.15	88.195	22.13
	NM_014946.ex.16	100	29.17
	NM_014946.ex.17	100	22.76
**SREK1**	NM_001077199.ex.1	100	11.40
	NM_139168.ex.2	100	6.60
	NM_139168.ex.5	100	23.20
	NM_139168.ex.6	100	10.14
	NM_139168.ex.7	100	14.30
	NM_139168.ex.8	100	3.67
	NM_139168.ex.9	100	20.89
	NM_139168.ex.10	70.88	20.70
	NM_139168.ex.11	8.25	45.52
	NM_139168.ex.12	100	15.85
	NM_139168.ex.13	100	32.37
**SYNGAP1**	NM_006772.ex.1	27	0.00
	NM_006772.ex.2	99.19	12.43
	NM_006772.ex.3	100	1.65
	NM_006772.ex.4	100	2.62
	NM_006772.ex.5	100	8.39
	NM_006772.ex.6	100	2.71
	NM_006772.ex.8	100	6.52
	NM_006772.ex.10	100	0.89
	NM_006772.ex.11	100	5.31
	NM_006772.ex.12	100	5.44
	NM_006772.ex.13	100	4.39
	NM_006772.ex.14	100	1.74
	NM_006772.ex.15	96.74	4.69
	NM_006772.ex.16	100	7.49
	NM_006772.ex.17	100	8.99
	NM_006772.ex.19	2.95	10.75
**TH**	NM_199292.ex.1	100	4.69
	NM_199292.ex.3	100	4.26
	NM_199292.ex.4	100	0.09
	NM_199292.ex.9	100	22.31
	NM_199292.ex.11	70.69	7.09
	NM_199292.ex.12	100	0.89
	NM_199292.ex.13	100	12.66
	NM_199292.ex.14	100	17.92
	NM_199293.ex.1	100	5.21
**TUBA1A**	NM_006009.ex.2	29.46	26.32
	NM_006009.ex.3	56.67	21.81
	NM_006009.ex.4	35.65	22.55

**TUBA3D**	NM_080386.ex.1	0	49.13
	NM_080386.ex.2	0	37.32
	NM_080386.ex.3	61.33	23.93
	NM_080386.ex.4	16.86	29.67
	NM_080386.ex.5	32.81	22.56
**TUBA8**	NM_001193414.ex.2	100	1.91
	NM_001193414.ex.3	100	9.65
	NM_001193414.ex.4	100	6.64
	NM_001193414.ex.5	100	23.27
	NM_018943.ex.1	100	16.87
**TUBB**	NM_178014.ex.1	100	10.12
	NM_178014.ex.2	36.36	27.98
	NM_178014.ex.3	100	12.44
	NM_178014.ex.4	36.13	15.07
**TUBB2B**	NM_178012.ex.2	100	1.62
	NM_178012.ex.3	100	0.15
	NM_178012.ex.4	21.04	9.28
**TUBB3**	NM_001197181.ex.4	82.26	5.03
	NM_006086.ex.1	100	20.73
**TUBG1**	NM_001070.ex.1	100	8.99
	NM_001070.ex.2	21.05	10.23
	NM_001070.ex.3	43.2	5.64
	NM_001070.ex.4	100	9.55
	NM_001070.ex.5	100	16.06
	NM_001070.ex.6	100	7.14
	NM_001070.ex.7	62.5	19.19
	NM_001070.ex.8	85.1	21.91
	NM_001070.ex.9	0	35.13
	NM_001070.ex.10	26.075	39.86
	NM_001070.ex.11	100	8.38
**UBE3A**	NM_000462.ex.4	100	27.52
	NM_130838.ex.1	100	32.56
	NM_130838.ex.2	100	9.58
	NM_130838.ex.3	79.17	12.05
	NM_130838.ex.4	100	13.44
	NM_130838.ex.5	100	21.57
	NM_130838.ex.6	90.36	37.77
	NM_130838.ex.7	100	12.70
	NM_130838.ex.8	100	7.65
	NM_130838.ex.9	100	8.35
	NM_130838.ex.10	8.26	5.21
	NM_130839.ex.3	100	20.88
	NM_130839.ex.4	100	32.56
**UBQLN3**	NM_017481.ex.2	100	2.09
**UBXN7**	NM_015562.ex.1	100	10.45
	NM_015562.ex.2	100	5.65
	NM_015562.ex.3	100	18.83
	NM_015562.ex.4	100	4.18
	NM_015562.ex.5	100	20.52
	NM_015562.ex.6	100	0.51
	NM_015562.ex.7	100	1.57
	NM_015562.ex.8	100	24.00
	NM_015562.ex.9	100	13.38
	NM_015562.ex.10	100	9.09
	NM_015562.ex.11	100	39.48
**UPF3B**	NM_080632.ex.1	71.98	12.98
	NM_080632.ex.2	100	31.48
	NM_080632.ex.3	100	3.92
	NM_080632.ex.4	100	30.73
	NM_080632.ex.5	94.64	29.95
	NM_080632.ex.6	100	29.76
	NM_080632.ex.7	63.04	38.04
	NM_080632.ex.8	100	21.36
	NM_080632.ex.9	100	8.35
	NM_080632.ex.10	100	18.05
	NM_080632.ex.11	100	10.92

**USP26**	NM_031907.ex.1	97.6	12.12
**VRK1**	NM_003384.ex.2	100	11.96
	NM_003384.ex.3	100	26.12
	NM_003384.ex.4	100	34.12
	NM_003384.ex.5	100	21.13
	NM_003384.ex.6	100	15.43
	NM_003384.ex.7	100	19.07
	NM_003384.ex.8	100	5.41
	NM_003384.ex.9	100	7.36
	NM_003384.ex.10	100	11.31
	NM_003384.ex.11	100	24.40
	NM_003384.ex.12	100	7.76
	NM_003384.ex.13	100	22.62
**WIPI2**	NM_001033520.ex.1	79.18	12.11
	NM_001033520.ex.2	100	2.53
	NM_001033520.ex.3	100	13.10
	NM_001033520.ex.4	100	1.73
	NM_001033520.ex.5	100	14.42
	NM_001033520.ex.7	100	2.68
	NM_015610.ex.2	67.27	33.45
	NM_016003.ex.1	45.36	38.15
	NM_016003.ex.2	100	4.40
	NM_016003.ex.3	100	2.58
**ZC4H2**	NM_001243804.ex.2	100	2.22
**ZDHHC9**	NM_001008222.ex.2	100	19.06
	NM_001008222.ex.3	100	16.18
	NM_001008222.ex.4	100	17.42
	NM_001008222.ex.5	100	22.77
	NM_001008222.ex.6	100	5.62
	NM_001008222.ex.7	100	23.89
	NM_001008222.ex.8	100	5.16
	NM_001008222.ex.9	100	5.43
	NM_001008222.ex.10	100	16.77
**ZMYM3**	NM_001171162.ex.2	98.84	7.78
	NM_001171162.ex.3	100	12.23
	NM_001171162.ex.4	64.71	36.33
	NM_001171162.ex.5	100	2.68
	NM_001171162.ex.6	75.98	13.90
	NM_001171162.ex.7	100	1.92
	NM_001171162.ex.8	100	6.95
	NM_001171162.ex.9	100	5.51
	NM_001171162.ex.10	100	12.47
	NM_001171162.ex.11	100	6.45
	NM_001171162.ex.12	100	6.23
	NM_001171162.ex.13	100	8.45
	NM_001171162.ex.14	100	7.00
	NM_001171162.ex.15	83.23	12.66
	NM_001171162.ex.16	100	8.72
	NM_001171162.ex.17	100	20.09
	NM_001171162.ex.18	100	3.78
	NM_001171162.ex.19	100	22.42
	NM_001171162.ex.20	100	2.89
	NM_001171162.ex.21	100	3.34
	NM_001171162.ex.22	98.28	3.49
	NM_001171162.ex.23	96.09	12.69
	NM_001171162.ex.24	100	19.12
	NM_001171162.ex.25	23.69	33.21
	NM_001171163.ex.7	100	7.56
	NM_005096.ex.14	100	12.26

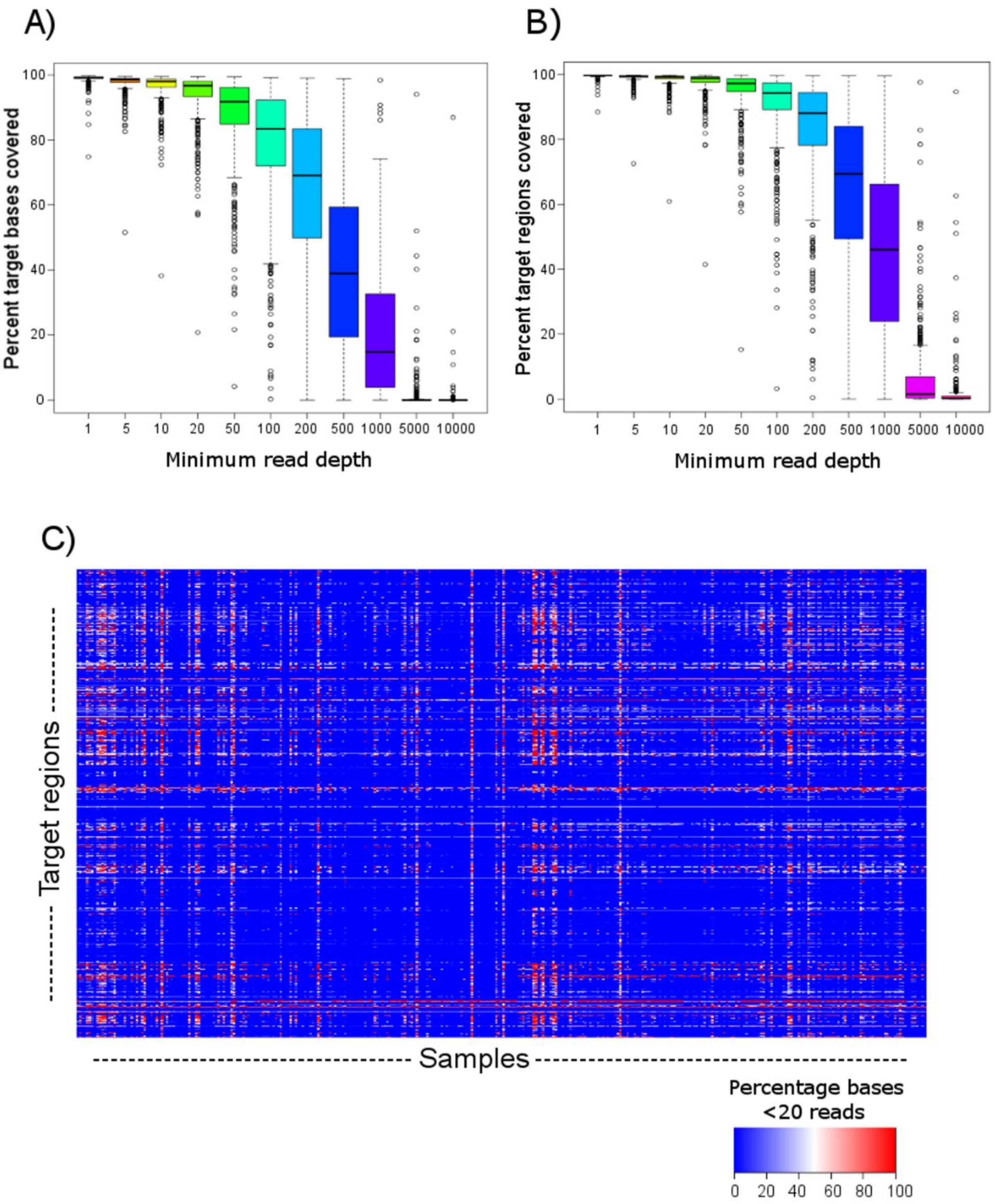

Supplementary Figure 1: Performance of HaloPlex gene panel. A) Percentage of target bases covered at minimum read depths. A median of 96.675% (minimum 86.530%, maximum 99.190%) of targeted bases were covered by at least 20 reads across all samples. **B)** Percentage of target regions covered at minimum read depths. A median of 98.875% (minimum 95.160%, maximum

99.700%) of targeted regions were covered by at least 20 reads across all samples. **C)** Heatmap of all samples analysed showing level of coverage of exons. Median percentage coverage per exon is listed in Supplementary Table 2.

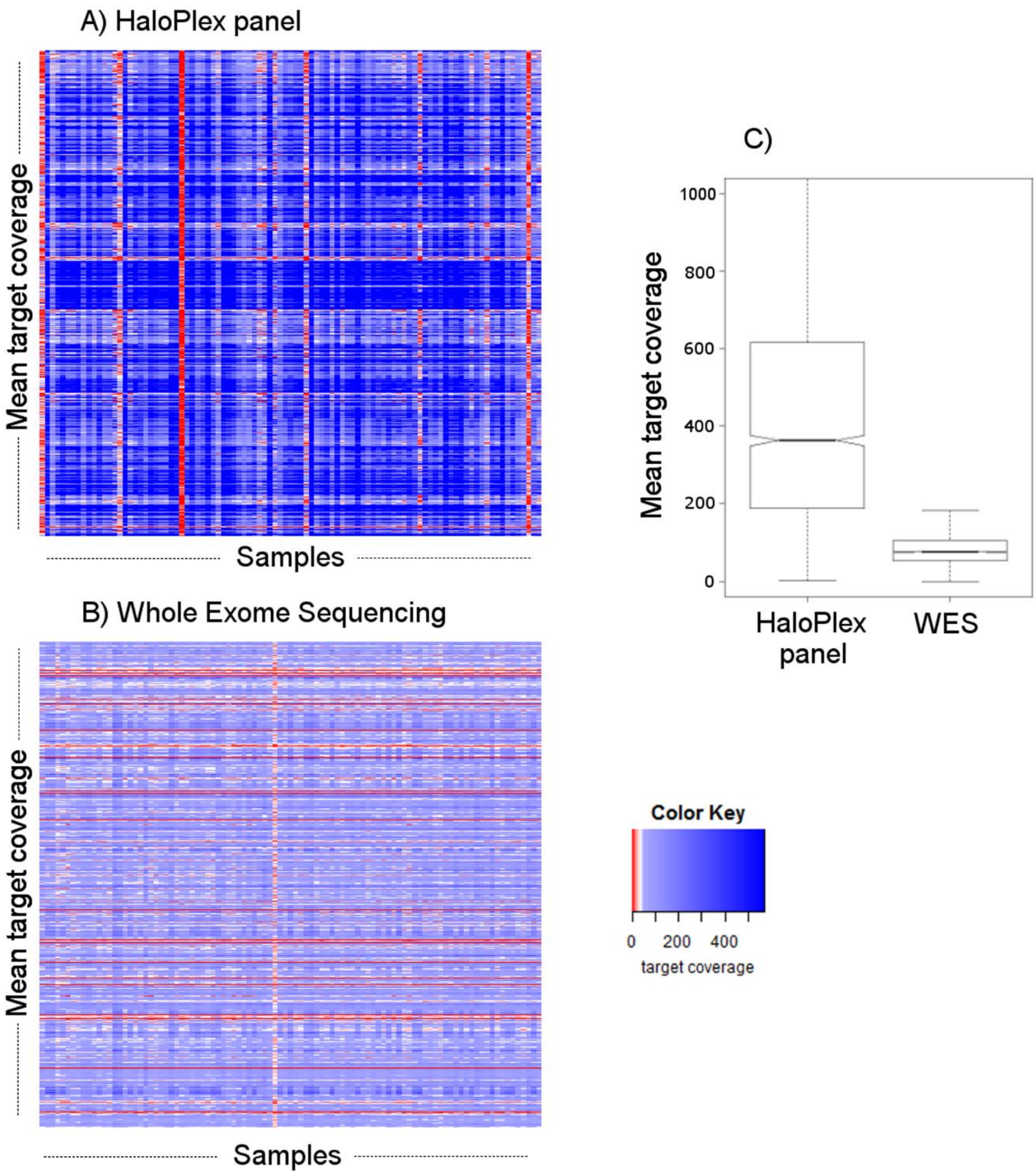

Supplementary Figure 2: Comparison of coverage achieved for HaloPlex target regions in HaloPlex and WES of a subset of 97 samples which were tested on both platforms. A) Heatmap of mean target coverage of HaloPlex targeted regions for each sample using HaloPlex. **B)** Heatmap of mean target coverage of HaloPlex targeted regions for each sample by whole exome sequencing. **C)** Boxplot of mean target coverage of HaloPlex targeted regions using each technology, demonstrating the difference in read depth achieved and the difference in sample to sample variability using each technology.

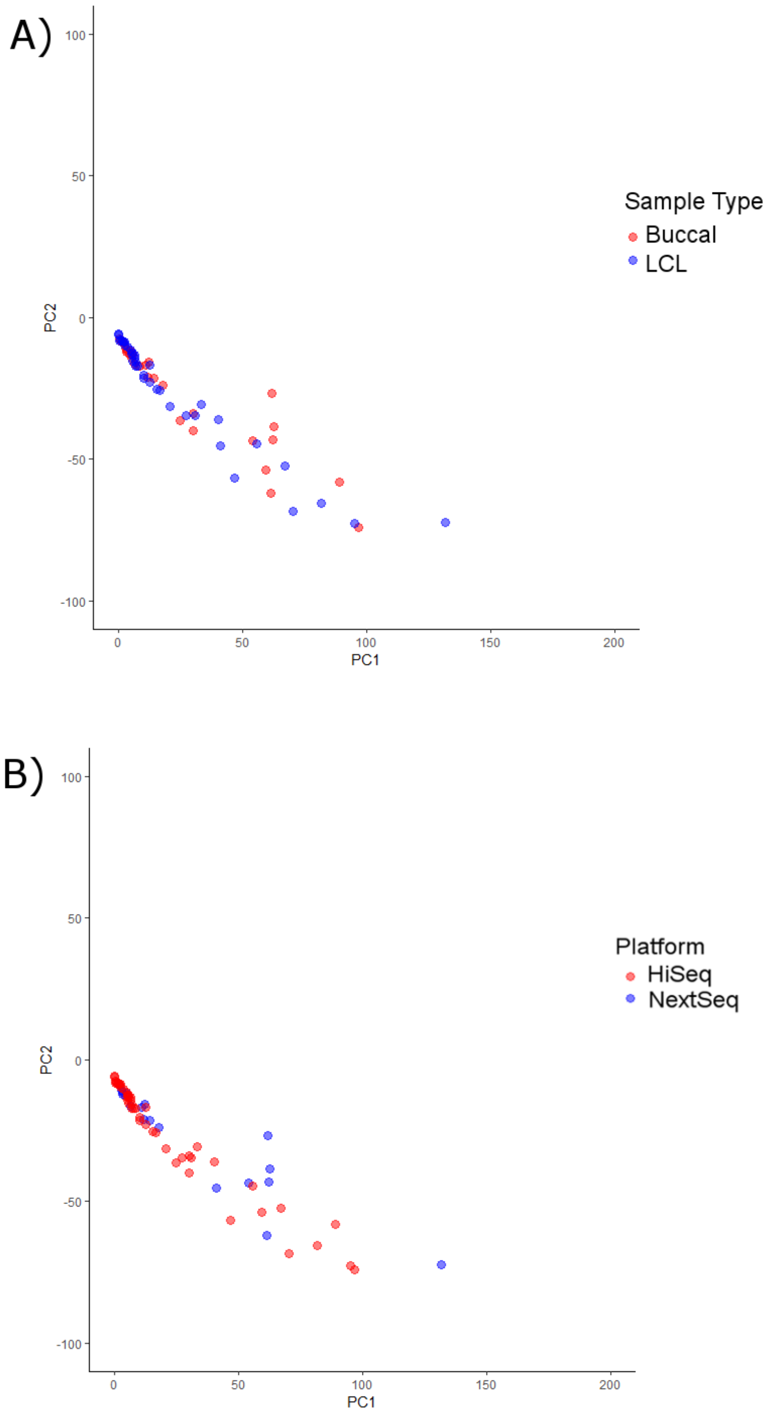

Supplementary Figure 3: Principal Component Analysis of target coverage for samples tested using the cerebral palsy HaloPlex gene panel. A) Samples coloured according to sample type (red, buccal; blue, Lymphoblastoid cell line). **B)** Samples coloured according to sequencing platform (red, Illumina HiSeq 2500 platform, 100bp paired end reads; blue, Illumina NextSeq 500 with 150bp paired end reads).

Supplementary Table 3 Variants detected in HaloPlex gene panel by gene

Gene	Sample	Inheritance	gnomAD	Variant	Predicted effect	CADD Phred	GERP++	MTR %ile	Poly-Phen2 HVAR	Mut. Taster	Clinical notes
**ABLIM2**	**P707**	Unknown	0	Chr4: 8037968delT	F/shift del NM_001286688.1: c.226del: p.Ser76Valfs*12	.	.	**.**	.	.	Female, spastic/dyskinetic quad, born 24 weeks, hyperkinetic, Hyaline Membrane Disease (Respiratory Distress Syndrome) after birth; required surgery for patent ductus. Necrotising enterocolitis as infant
	**P1143**	Maternal	3.29E-05	Chr4:8108337G>C	NM_001130083.2: c.38C>G: p.P13R	24.1	3.22	**25.08**	**D**	**D**	Male, spastic hemi, born 40 weeks, DD, Ep, left homonymous hemianopia. MRI: Extensive changes of gliosis with cystic encephalomalacia involving almost the entire right cerebral hemisphere with associated ex vacuo dilatation of the right lateral ventricle and changes of Wallerian degeneration in the right cerebral peduncle. Some non specific gliotic changes are also noted in the left periventricular white matter especially in the peritrigonal region.
**ADCY3**	**P443**[1]	Paternal	0	Chr2: 25064186G>A[2]	NM_004036.4: c.1138C>T: p.R380W	**33**	4.6	**0.786**	**D**	**D**	Female, spastic hemi, born 39 weeks, maternal uncle severe physical and mental disability.
	**P947**	Unknown	4.07E-06	Chr2:25057679G>A	Stopgain NM_004036.4: c.1789C>T: p.R597*	**43**	4.21	.	.	**A**	Male, born 25 weeks, spastic/dystonic quad, DD, ventricular bleed at 6 weeks of age, uncle with ASD, MRI: PVL and IVH.
	**16922**	Not maternal	2.04E-05	Chr2:25054544G>T	NM_004036.4: c.2042C>A: p.A681D	**26.7**	**5.4**	**49.08**	**D**	**D**	Male, born 38 weeks, maternal great aunt CP.
**AGAP1**	**P033**[1]	De novo	0	Chr2: 236708167G>A[2]	Splicing NM_014914.4: c.957+1G>A	**27.3**	**5.08**	.	.	**D**	Male, spastic quad with generalised hypotonia, dystonic posturing and myoclonic jerks, born 37 weeks severe ID, Ep, reduced oral control and tongue movement, MRI: PVL in frontal and parietal lobes (L>R), absent L caudate nucleus, putamen and globus pallidus, short stature at 4 years, died at 7 years.

	**P738**	De novo	1.94E-04	Chr2: 236877181C>G	NM_014914.4: c.1400C>G: p.P467R	**27.4**	4.21	77.01	P	**D**	Female, born 28 weeks, spastic diplegia, mild dystonic, TTTS, DD, identical twin (status unknown). MRI: Posterior periventricular changes consistent with PVL. A hypogenetic corpus callosum with an absent splenium. Linear lipoma over the corpus callosum.
	**P1126**	Not maternal	0	Chr2:236817458C>T	NM_001037131.3: c.1232C>T: p.P411L	**33**	4.66	**11.42**	**D**	**D**	Male, spastic quad, born 30 weeks, DD, Ep, squint nystagmus, feeding difficulties, cholestasis, both parents and sister visual problems, brother ADHD. MRI: Bilateral grade III intraventricular haemorrhage with moderate asymmetric ventriculomegaly with mild rightward subfalcine herniation. Large-volume, bilateral periventricular cystic encephalomalacic cavities. Persistent haemorrhages within both cystic cavities is noted. Persistent bilateral large-volume expanded periventricular leukomalacic cysts which appear to communicate directly with the lateral ventricles. Moderate dilation of the lateral and third ventricles appears secondary to an acquired aqueduct stenosis; in addition, features of a prior basilar arachnoiditis may reflect abnormal CSF dynamics at the 4th ventricular outlet foramina and basilar cisterns.
	**10222**	Unknown	1.82E-05	Chr2: 237028942G>A	NM_014914.4: c.2062G>A: p.A688T	22.8	4.66	64.34	**D**	**D**	Male, born 40 weeks, DD, Ep
**AP4E1**	**P119**[1]	Paternal	0	Chr15: 51242290C>T	NM_001252127.1: c.1247C>T: p.A416V	**26.3**	4.86	**2.63**	P	**D**	Female, spastic hemi, born 39 weeks, BW: 9th percentile IUGR, Ep. US: appearances consistent with mature left MCA territory infarct. MRI: dilatation of the left lateral ventricle, left hemisphere is reduced in volume when compared with the normal right side. There is an area of encephalomalacia in the left posterior frontal and anterior parietal

											regions most marked in the vicinity of the left sylvian fissure.
	**P783**	Unknown	3.29E-05	Chr15: 51233946C>T	NM_001252127.1: c.925C>T: p.H309Y	**26.7**	**5.39**	77.33	**D**	**D**	Male, spastic hemi, born 38 weeks, BW: 2nd percentile IUGR, moderate DD, seizures at birth, tachycardia, hypoglycaemia, parents cousins
	**P934**	Not maternal	0	Chr15: 51201028A>T	NM_007347.5: c.53A>T: p.Q18L	21.6	**5.39**	56.77	B	**D**	Male, spastic hemi, born 41 weeks. MRI: Focal area of gliosis involving the posterior limb of the right internal capsule and the right centrum semiovale.
	**P950**	Maternal	2.84E-05	Chr15:51250786A>G	NM_001252127.1: c.1421A>G: p.E474G	**25**	4.48	**38.29**	B	**D**	Male, spastic hemi, born 40 weeks, ASD, visual problems, antenatal seizures, parents related, relationship not specified, transposition of great arterial vessels, stroke as baby during heart operation.
**AP4M1**	**P758**	Not maternal	0	Chr7:99699541T>C	NM_004722.3: c.97T>C: p.F33L	23	**5.26**	**35.68**	B	**D**	Male, spastic diplegia, born 40 weeks, ASD (cousin also), hearing loss (grandmother also), MRI: open lipped schizencephaly on the right and either transmantle heterotopia or closed lip schizencephaly demonstrated on the left, associated with large extra-axial CSF spaces. Unclear whether these are simply secondary to the malformation or represent arachnoid cysts. Suggestion of some mass effect on the left side with enlargement of the middle cranial fossa. Multiple areas of polymicrogyria, absence of the septum pellucidum and small optic nerves.
**AP4S1**	**P928**	Unknown	0	Chr14:31535491T>A	NM_001128126.2: c.89T>A: p.L30H	24.4	**5.99**	98.62	**D**	N	Female, spastic diplegia, born 29 weeks, antepartum haemorrhage.
**APOE**	**P729**	Maternal	4.54E-04, familial hyperlipo-proteinemia	Chr19: 45412314T>A	NM_000041.4: c.761T>A: p.V254E	**27.6**	3.56	95.91	P	N	Male, spastic diplegia, born 33 weeks, anxiety. MRI and US: loss of white matter volume in the brain and periventricular white matter changes.
**ATP11B**	**10786**	Unknown	1.63E-05	Chr3:182575775T>C	NM_014616.3: c.961T>C: p.W321R	**26.6**	2.61	64.61	B	**D**	Male, born 36 weeks, chronic lung disease, moderate-severe hearing loss

	**P800**	Unknown	0	Chr3:182607310G>T	NM_014616.3: c.2956G>T: p.D986Y	**28.7**	**5.7**	66.52	**D**	**D**	Male, spastic hemi, born 39 weeks, BW: 7th percentile IUGR, Factor V Leiden deficiency, hearing loss (cousin also), use of right hand has declined.
**AUH**	**P907**	Not maternal	6.49E-05	Chr9:94118175T>C	NM_001306190.1: c.408A>G: p.I136M	24.8	2.61	87.95	**D**	**D**	Male, spastic diplegia, born 28 weeks, BW: 9th percentile IUGR, twin, non-identical, DD. MRI: PVL.
	**10749**	Unknown	0	Chr9:94124018G>A	Stopgain NM_001306190.1: c. 154C>T: p. Q52*	**33**	1.68	76.87	.	**A**	Female, born 38 weeks, Ep
**BRWD3**	**P968**	Unknown	0	ChrX:79951470C>T	NM_153252.4: c.3088G>A: p.V1030I	**33**	**5.45**	**10.54**	**D**	**D**	Female, dyskinetic/ spastic hemi, born 40 weeks, diagnosed neonatal encephalopathy, learning difficulty, ADHD, OCD.
	**10941**	Maternal	5.95E-06	ChrX:80064053T>G	NM_153252.4: c.165A>C: p.R55S	23.2	4.97	**16.00**	B	**D**	Female, born 27 weeks, GORD
**CNDP2**	**P109**[1]	De novo	0	Chr18:72173219dupAGC	NM_018235.3: c.504_506dupAGC: p.E114_P115insQ	.	.	.	.	.	Male, spastic hemi, born 35 weeks, BW: 2nd percentile IUGR, history of in utero CVA, Ep, obesity at 8 yrs; DD, significant learning difficulties, left homonymous hemianopia. CT: large right MCA territory infarct, in utero CVA. Extensive hypodensity involving vast majority of under developed right hemisphere and associated ex-vacuo dilatation of the right lateral ventricle. Right side of the calvarium is underdeveloped compared to the left. Extensive change to the right hemisphere.
	**P043**[1]	Maternal	4.10E-06	Chr18:72173171G>A	NM_018235.3: c.292G>A: p.G98R	**26.6**	4.52	**18.07**	**D**	**D**	Female, spastic hemi, born 41 weeks, mild ID, elbow flexion deformity. CT: Abnormality in left hemisphere, likely consistent with an old MCA territory infarct. Left ventricle dilated, low white matter and a calcium focus. Small left thalamus, smaller left brain in general.
**CNKSR2**	**P115**[1]	X-linked	2.02E-05	ChrX:21508607A>G[2]	NM_001168647.2: c.592A>G: p.I198V	15.67	**5.83**	50.43	**D**	**D**	Male, spastic diplegia, born 41 weeks, moderate ID; Ep (mostly complex partial seizures, 3-5 per month); ASD; expressive speech delay; severe visual

											impariment, optic nerve atrophy; adducted thumbs; behavioural problems but generally sociable and happy. US: congenital tetraventricular hydrocephalus, MRIs: hydrocephalus communicating with persistent Blake's pouch cyst, cerebellar folia disorganisation, white matter volume loss, partial agenesis of the corpus callosum, abnormal positioning of thalamus and lentiform nucleus, incomplete hippocampal inversion bilaterally, hypoplasia of the optic nerve, hypoplasia of the cerebellar vermis and asymmetry of the pons.
**COL4A1**	**P033**[1]	Paternal	0	Chr13:110831315C>T	NM_001845.6: c.2413G>A: p.G805R	24.6	4.7	84.91	**D**	**D**	Male, spastic quad with generalised hypotonia, dystonic posturing and myoclonic jerks, born 37 weeks severe ID, Ep, reduced oral control and tongue movement, MRI: PVL in frontal and parietal lobes (L>R), absent L caudate nucleus, putamen and globus pallidus, short stature at 4 years, died at 7 years.
	**P035**[1]	Not maternal	8.12E-06	Chr13:110895030C>T	NM_001303110.1: c.136G>A: p.G46R	**25.7**	4.26	64.81	**D**	**D**	Female, spastic hemi, born 27 weeks, BW: <1 percentile IUGR, mild ID, impaired hearing, divergent squint, Ep, thyroid agenesis, deterioration of motor function and bladder control; at 5 years, sleep apnoea, recurrent ear infections, PEG feeding, short stature and underweight at 16 years, US: L PVL, CT: mild ex-vacuo ventricular dilation.
	**P106**[1]	Paternal	0	Chr13:110813663T>C	NM_001845.6: c.4516A>G: p.N1506D	20.3	4.56	**22.10**	P	**D**	Male, spastic hemi, born 39 weeks, bilateral femoral anteversion
	**P204**	Not maternal	0	Chr13:110830543C>T	NM_001845.6: c.2494G>A: p.G832R	**27.8**	**5.52**	**43.59**	**D**	**D**	Male, choreoathetoid quad, born 40 weeks, MRI: haemorrhagic gliosis, known COL4A1 mutation from diagnostic WES, cystic porencephaly involving basal ganglia; damage to small blood vessels with increased risk of haemorrhage; Epileptic encephalopathy,

											infantile spasms onset 1 year, evolution into mixed seizure types now; unexplained irritability; no speech or communication, global DD; tracheobronchomalacia; laryngomalacia; gastrostomy; fundoplication; scoliosis; left ventricular hypertrophy and hypertension; enlarged left kidney >10% discrepancy; dental caries; sleep apnoea; hip surgery, past femoral osteostomies; chronic lung disease; failure to thrive; hypoplastic optic nerves, MRI: haemorrhagic gliosis.
	**P981**	Not maternal	6.63E-05	Chr13:110831281G>A	NM_001845.6: c.2447C>T: p.P816L	22.7	4.89	59.22	**D**	**D**	Male, spastic diplegia, born 36 weeks, BW: 4th percentile IUGR, neonatal seizures, DD, visual problems, MRI and CT: Grade 2 sub-ependymal haemorrhage. CT demonstrates a germinal matrix haemorrhage with intraventricular extension.
	**P1116**	Maternal	7.21E-05	Chr13:110804753C>T	NM_001845.6: c.4856G>A: p.R1619H	**26**	**5.51**	**5.51**	**D**	**D**	Female, spastic hemi, born 27 weeks, BW: 8th percentile IUGR, neonatal lung disease, subgliotic stenosis requiring tracheostomy, developed IVH grade IV soon after birth, hydrocephalus with papilledema developed at 12 months, pyloric stenosis, mild hearing loss, esotropia corrected by glasses, DD, MRI: R grade 4 and L grade 2 IVH. Persistent hydrocephalus, right porencephalic cyst, mild PVL posteriorly.
**COPS3**	**16458**	Not maternal	0	Chr17:17174300T>A	NM_001316356.1: c.7A>T: p.S3C	24.6	**5.52**	92.08	P	**D**	Male, twin, born 35 weeks, hydrocephalus
**CTDSPL**	**P030**[1]	De novo	0	Chr3:38009345C>T[2]	NM_005808.2: c.365C>T: p.P122L	**34**	**5.25**	**5.26**	**D**	**D**	Male, spastic quad, born 37 weeks, BW: <1 percentile IUGR, DD, Ep with myoclonic seizures, cortical visual impairment, cataracts, scoliosis thoraco-lumbar and kyphosis mid thoracic; bilateral undescended testes, non-verbal, communicates with communicator. Experienced episodes of

											severe bradycardia before onset of labour. Placenta showed multiple infarcts and there was a velamentous insertion of the cord. Had some limb contractures at delivery associated with oligohydramnios. At birth required sedation and ventilation and had evidence of cerebral oedema in neonatal period. MRI: Scaphocephaly of the skull with an elongated narrowed brain. Moderate ventricular dilation more marked on the left with enlarged subarachnoid space. Immaturity in myelination, particularly in temporal lobes and parietal regions. High signal periventricular white matter changes suggesting PVL. Probably subependymal grey matter heterotopias. Hypoplastic thin corpus callosum.
**CTNND2**	**P008**[1]	Paternal	0	Chr5:11111031T>C	NM_001288716.1: c.1391A>G: p.K464R	18.23	**5.72**	**15.52**	B	**D**	Male, spastic triplegia, born 35 weeks, right orchidopexy, constipation.
	**P099**[1]	Unknown	3.97E-05	Chr5:11022927C>T	NM_001288716.1: c.1942G>A: p.G648S	**26.9**	**5.64**	55.00	**D**	**D**	Variant not detected by WES. Male, spastic diplegia, born 31 weeks, twin, convergent squint paternal cousin CP. Hyaline membrane disease, ventilated for first 3 days, therafter 5 days CPAP and treated with caffeine for apnoeas and bradycardias. Jaundice with a max bilirubin of 188 led to phototherapy. Strabismus surgery, esotropia, mild optic nerve dysfunction and inferior visual field defect; equinus gait pattern. US: day 2 normal, day 8; visible flare in the frontal and occipital regions on the left and evidence of a small resolving haemorrhage in the right lateral ventricle. PVL with cystic encephalomalacia in the areas of the left frontal region and left occipital region have progressed.

	**P739**	Maternal	2.76E-05	Chr5:11346700C>T	NM_001288716.1: c.401G>A: p.R134H	**34**	**5.79**	90.10	**D**	**D**	Male, spastic/dystonic quad, born 36 weeks, BW: <1 percentile IUGR. MRI: Bilateral nearly symmetrical periventricular white matter hyperintensity, predominantly involving the deep white matter, suggestive of a demyelination/dysmyelination disorder.
**DDHD2**	**P104**[1]	Paternal	0	Chr8:38092037T>G	NM_001164232.1: c.346T>G: p.F116V	**28.5**	**5.79**	**29.35**	**D**	**D**	Male, spastic hemiplegia, born 40 weeks, BW: <1 percentile IUGR, hydrocephalus, congenital hyper-insulinaemia, severe hypoglycaemia and thrombocytopenia, basal ganglia haemorrhages, accommodative estropia. US: bilateral grade III IVH, ventriculomegaly. Later US suggest progression of IVH involving both lateral ventricles, with associated dilatation of both lateral ventricles, when compared to first US. MRI (at day 12 following severe hypoglycaemia and sepsis, severe thrombocytopenia): extensive bilateral basal ganglia, intraventricular and basal cistern haemorrhage with evolving
	**10397**	Not maternal	3.25E-06	Chr8:38109665C>T	NM_001164232.1: c.1477C>T: p.P493S	24.2	**5.17**	**49.72**	P	**D**	Female, born 41 weeks, hydrocephalus, DD, Ep, vision impairment
**DHX32**	**10135**	Not maternal	4.72E-05	Chr10: 127548237G>A	NM_018180.2: c.784C>T: p.L262F	**25.8**	4.84	95.43	**D**	**D**	Female, possible Adams Oliver syndrome
**DMD**	**P202**	X-linked	0	ChrX:31144772G>A	NM_004015.2: c.1790A>G: p.K597R	22.9	**6.02**	97.19	**D**	**D**	Male, spastic diplegia, born 39 weeks, hydrocephalus secondary to aqueduct stenosis, benign mature teratoma excised, normal cognition and behaviour, considerable lower limb spasticity that impacts on gross motor function, particularly gait quality, reasonable to good selective motor control and strength. MRI: High signal on flair in the region trigones is suggestive of PVL. Absent septum pellucidum and thinning / irregularity of corpus

											callosum. Right parietal approach shunt in situ.
	**P788**	Unknown	5.60E-06	ChrX:31222094C>T	NM_004015.2: c. 587A>G: p.R196Q	**29.8**	**5.35**	**27.27**	**D**	**D**	Male, dystonic quad, severe dysphagia, Ep, DD (brother/sister also), mother, brother visual problems, CT/MRI: postnatal herpetic meningoencephalitis
	**P968**	Unknown	1.68E-05	ChrX:31222190C>T	NM_004015.2: c.491G>A: p.R164H	**34**	4.47	53.39	**D**	**D**	Female, dyskinetic/ spastic hemi, born 40 weeks, diagnosed neonatal encephalopathy, learning difficulty, ADHD, OCD.
	**10744**	Unknown	0	ChrX:31196915G>T	Stopgain NM_004015.2: c.C890A: p.S297*	**57**	**5.45**	.	.	**A**	Female, born 40 weeks, twin
**DNAH2**	**P015**[1]	Unknown	0	Chr17:7643054T>C	NM_020877.3: c.1174T>C: p.C392R	**26.8**	**5.09**	88.21	**D**	**D**	Variant not detected in WES, Male, dystonic spastic hemiplegia, born 29 weeks, BW: <1 percentile IUGR Ep, cognitive ability upper limit of low-average range, developed hydrocephalus, MRI: grade 4 IVH, right sided and grade 2 IVH left sided
	**P068**[1]	Paternal	2.03E-05	Chr17:7643207delG	F/shift del NM_020877.2: c.1327delG: p.Gly444Valfs*26	.	.	.	.	.	Variant not detected by WES. Male, spastic hemi, born 39 weeks, induced at term because of unusual abdominal pain. Hypoglycaemia, mild asthma, mild scaphocephaly consistent with intrauterine moulding, possible left septal hypertrophy and RVH with borderline ECG, likely pre-natal CVA. US: ex-vacuo dilation of right ventricle. MRI: right PVL and cortical involvement in region of right pre-central gyrus. CT: ex-vacuo dilation of right ventricle and long-standing changes consistent with hypoxic ischaemic injury.
	**P103**[1]	Maternal	0	Chr17:7644290C>T	NM_020877.3: c.1669C>T: p.R557C	**31**	**5.23**	**27.55**	**D**	**D**	Male, spastic hemi, Ep, ID, ASD, significant behaviour problems , attention seeking behaviour, interruptive and manipulative at a young age, speech problems, VSD spontaneously closed

**P116**[1]	Maternal	0	Chr17:7734801C>A	NM_020877.3 c.12553C>A pS3698Y	21.6	**5.3**	**47.29**	P	**D**	Female, spastic quad, born 26 weeks, DD, ID, short stature (3rd percentile), chronic lung disease requiring home oxygen. US: IVH grade II left, grade II/III right, then ventriculomegaly, porencephaly
**P204**	Maternal	2.89E-05	Chr17:7671564G>A	NM_001845.6: c.2494G>A: p.G832R	**32**	3.58	89.07	**D**	**D**	Male, choreoathetoid quad, born 40 weeks, MRI: haemorrhagic gliosis, known COL4A1 mutation from diagnostic WES, cystic porencephaly involving basal ganglia; damage to small blood vessels with increased risk of haemorrhage; Epileptic encephalopathy, infantile spasms onset 1 year, evolution into mixed seizure types now; unexplained irritability; no speech or communication, global DD; tracheobronchomalacia; laryngomalacia; gastrostomy; fundoplication; scoliosis; left ventricular hypertrophy and hypertension; enlarged left kidney >10% discrepancy; dental caries; sleep apnoea; hip surgery, past femoral osteostomies; chronic lung disease; failure to thrive; hypoplastic optic nerves, MRI: haemorrhagic gliosis.
**P215**	Maternal	0	Chr17:7680779A>G	NM_020877.3: c.5074A>G: p.K1692E	23.8	**5.51**	73.89	P	**D**	Female, spastic diplegia, born 31 weeks, DD, visual problems, Noonan syndrome, cousin ASD, uncle ADHD, squint; short stature, faints; insomnia; GORD; dental caries related to chronological hypoplasia and enamel development defect; pulmonary valve stenosis; IgA deficiency; hypoglycaemia; early iron deficiency with microcytosis but not anaemia. MRI: evidence of PVL.
**P427**[1]	Compound het/ Maternal	4.06E-06	Chr17:7660536G>A	NM_020877.3: c.2032G>A: p.V678I	20.6	3.98	57.43	**D**	**D**	Male, spastic hemi, born 38 weeks.
**P427**[1]	Compound het/ Paternal	0	Chr17:7696064T>G	NM_020877.3: c.7235T>G: p.I2412S	21.9	3.27	**20.53**	B	N	As above

	**10749**	Unknown	3.97E-05	Chr17:7661890C>T	NM_020877.3: c.2129C>T: p.P710L	**34**	**5.91**	**4.61**	**D**	**D**	Female, born 38 weeks, Ep
**DNAH3**	**P026**[1]	Maternal	3.97E-05	Chr16:21060927C>G	NM_017539.2: c.4424G>C: p.G1475A	**27.5**	**5.88**	**9.67**	**D**	**D**	Female, spastic quad with greater involvement of lower limbs, born 39 weeks, BW: 5th percentile IUGR, small stature, convergent strabismus, ridging metopic suture, mild trigonocephaly, severe behaviour problems, speech dyspraxia, photophobia
	**P049**[1]	Maternal	0	Chr16:20970654T>C	NM_017539.2: c.10673A>G: p.Q3558R	**26.8**	**5.41**	86.79	P	**D**	Male, spastic hemi, born 35 weeks, BW: 5th percentile IUGR, MRI: Slight ventricle asymmetry, two small subependymal nodules on the lateral wall of the right lateral ventricle pointing to minor variant neuronal migration disorder (subependymal heterotopia), some asymmetry of silvian fissures but no definite polymicrogyria or pachygyria.
	**P174**	Not maternal	6.50E-05	Chr16:20976075G>A	NM_017539.2: c.9131C>T: p.T3044M	24.7	**6.04**	55.11	P	**D**	Male, spastic quad, born 40 weeks, partial dysgenesis of corpus callosum, global DD, Ep (first seizure at 12 months), optic atrophy (cortically blind); type 1 diabetes, scoliosis in the lumbar region and an oblique pelvis, incontinent of faeces and urine, cortical atrophy has been progressive.
	**P707**	Unknown	8.31E-05	Chr16:21008634G>A	NM_017539.2: c.6572C>T: p.P2191L	**28.3**	**5.23**	**49.84**	**D**	**D**	Female, spastic/dyskinetic quad, born 24 weeks, hyperkinetic, Hyaline Membrane Disease (Respiratory Distress Syndrome) after birth; required surgery for patent ductus. Necrotising enterocolitis as infant
	**P721**	Maternal	2.03E-05	Chr16:21008662C>T	NM_017539.2: c.6544G>A: p.D2182N	**32**	**5.23**	**40.71**	**D**	**D**	Male, spastic/dystonic quad, dysphonia, breathing difficulty, progressive planovalgus deformity
	**P745**	Unknown	5.78E-05	Chr16:20986640G>A	NM_017539.2: c.8174C>T: p.T2725M	**26.9**	**5.73**	**46.44**	**D**	**D**	Male, spastic hemi
	**P750**	Maternal	2.03E-05	Chr16:21049282G>A	NM_017539.2: c.4751C>T: p.A1584V	**31**	**5.76**	90.11	**D**	**D**	Male, dystonic diplegia, born 42 weeks, previous iron deficiency anaemia

											(resolved); has pulmonary stenosis (balloon dilation, aged 3 months).
	**P934**	Not maternal	3.26E-05	Chr16:21133303A>G	NM_017539.2: c.1547T>C: p.F516S	**27.6**	**5.21**	97.43	**D**	**D**	Male, spastic hemi, born 41 weeks. MRI: Focal area of gliosis involving the posterior limb of the right internal capsule and the right centrum semiovale.
	**P980**	Not maternal	8.30E-05	Chr16:20975977G>A	NM_017539.2: c.9229C>T: p.R3077C	**29.7**	4.88	60.55	**D**	**D**	Male, spastic quad, born 26 weeks, DD, Ep, OCD. MRI: Grade IV germinal matrix haemorrhage with IVH.
	**P1116**	Maternal	3.66E-05	Chr16:21086770T>C	NM_017539.2: c.3082A>G: p.T1028A	**25.5**	**6.02**	**13.68**	**D**	**D**	Female, spastic hemi, born 27 weeks, BW: 8th percentile IUGR, neonatal lung disease, subgliotic stenosis requiring tracheostomy, developed IVH grade IV soon after birth, hydrocephalus with papilledema developed at 12 months, pyloric stenosis, mild hearing loss, esotropia corrected by glasses, DD, MRI: R grade 4 and L grade 2 IVH. Persistent hydrocephalus, right porencephalic cyst, mild PVL posteriorly.
	**10275**	Unknown	1.63E-05	Chr16:20981231C>T	NM_017539.2: c.8341G>A p.D2781N	**29.5**	**5.97**	66.00	**D**	**D**	Female, born 28 weeks, BW: 1st percentile IUGR
	**10763**	Unknown	4.06E-06	Chr16:21139102C>T	NM_017539.2: 1114G>A: p.E372K	23.6	**5.42**	**4.76**	P	**D**	Female, born 39 weeks
	**11014**	Unknown	0	Chr16:21145680G>A	NM_017539.2: c.982C>T: p.P328S	**27.9**	**5.85**	80.52	**D**	**D**	Male, born 40 weeks, ASD
	**11111**	Not maternal	5.78E-05	Chr16:20986640G>A	NM_017539.2: c.8174C>T: p.T2725M	**26.9**	**5.73**	**46.44**	**D**	**D**	Female, born 35 weeks, BW: <1 percentile IUGR, DD, Ep
**DYNC2H1**	**P003**[1]	Paternal	0	Chr11:103058091T>C	NM_001080463.1: c.6916T>C: p.S2306P	21.2	**5.12**	53.21	P	**D**	Female, spastic diplegia with mild dystonia especially in upper limbs, twin, born 32 weeks, cortical visual impairment, DD, Ep, convergent squint, some cognitive impairment, MRI: IVH, Irregular enlargement of ventricles particularly posteriorly with reduction in white matter volume and mild delay in myelination.

**P188**	Unknown	2.97E-04	Chr11:103027166G>A	NM_001080463.1: c.3794G>A: p.R1265H	23.1	**5.27**	**29.25**	B	**D**	Female, spastic quad with some evidence of dyskinesia, born 37 weeks, BW: 1st percentile IUGR, induced birth due to oligohydramnios and IUGR, diagnosed neonatal encephalopathy, hypoglycaemic day 2 and lactic acidosis on initial cord bloods; apnoeic episodes/seizures day 9; stiff abnormal flexion/extension and fine tremor; difficulties maintaining midline orientation. No evidence of encephalopathy until onset of apnoea and seizures. Moderate hearing deficits, squint and cortical visual impairment; cranial nerve palsies, GORD, feeding difficulties; vomiting and slow weight gain, lactose intolerant, recurrent otitis media and tonsillitis, BPD (maternal Grandmother also). MRI: extensive injury of frontoparietal white matter basal ganglia and thalamic nuclei; symmetric hypoxic brain injury.
**P206**	Maternal	3.22E-05	Chr11:103041621G>A	NM_001080463.1: c.5158G>A: p.D1720N	**34**	4.92	**46.46**	**D**	**D**	Female, spastic hemi, born 43 weeks, BW: <1 percentile IUGR, DD, microcephaly, asthma and eczema. Mild spastic left arm with elbow contracture; poor dexterity with the left hand. Mild increased tone in her left leg and wears an orthotic. MRI: Nonspecific white matter lesions bilaterally. CT: Possible generalised cerebral atrophy with prominence of the ventricular system, suggestive of possible PVL. There are also small areas of calcification adjacent to the right lateral ventricle.
**P209**	Maternal	1.87E-05	Chr11:103114464A>T	NM_001377.3: c.9862A>T: p.T3288S	22.7	**5.66**	77.60	P	**D**	Male, spastic quad, born 38 weeks, diagnosed neonatal encephalopathy, ID, global DD, GORD, hearing impairment, gastrostromy, drooling. EEG: very abnormal with very asymmetric electroencephalogram. MRI: Early neuroimaging showed diffuse cerebral

										oedema, a severe global brain injury with severe white and grey matter changes. Evolution of extensive cystic encephalomalacia changes both within a watershed distribution in the cerebral hemispheres bilaterally, but also involving most of the parenchymal cortex with sparing of a small portion of the left frontal and left medial temporal cortices. Brainstem and cerebellum are normal. Basal ganglia are affected bilaterally, more severely on the right, with some sparing of the left thalamus and left globus pallidus.
	**P740**	Not paternal	0	Chr11:103116029C>G	NM_001377.3: c.9968C>G: p.T3323S	23	**5.64**	**36.17**	**D**	**D**	Male, spastic quad, born 28 weeks, lower limbs significantly more affected than the upper limbs.
	**10941**	Not maternal	4.25E-06	Chr11:103043968A>G	NM_001080463.1: c.5492A>G: p.Y1831C	**26.8**	**5.65**	**36.11**	**D**	**D**	Female, born 27 weeks, GORD
**EIF4E2**	**P111**[1]	De novo	0	Chr2:233422672C>G[2]	NM_001276336.1: c.214C>G: p.P72A	16.93	**5.27**	**44.44**	B	**D**	Male, spastic diplegia, born 41 weeks, hypoglycaemia, seizures day 3, thrombocytopenia, high pitch cry as neonate, mild squint and hypermetropia. US day 3: increased periventricular echogenicity, with left frontal and right parietal oedema. MRI: 1 week, restricted diffusion with parasagittal and basal ganglia involvement and PVL. MRI 2 months, shows residual changes of PVL only.
	**P800**	Unknown	0	Chr2:233431575C>T	NM_001276337.1: c.277C>T: p.R93W	**25.4**	**5.53**	85.54	P	**D**	Male, spastic hemi, born 39 weeks, BW: 7th percentile IUGR, Factor V Leiden deficiency, hearing loss (cousin also), use of right hand has declined.
**ENOX2**	**P405**[1]	X-linked	0	ChrX:129822993C>G[2]	Splicing NM_006375.3: c.98-1G>C	19.47	**5.1**	.	.	.	Male, spastic diplegia, born 30 weeks
**ENPP4**	**P172**	Unknown	4.13E-06	Chr6:46111046G>C	NM_014936.5: c.1031G>C: p.S344T	19.71	**5.01**	**44.71**	P	**D**	Female, spastic diplegia, born 35 weeks, acute disseminated encephalomyelitis; CNS demyelination in infancy (leukodystrophy/

											leukoencephalyopathy), obstructive sleep apnoea - adenoids removed.
	**P204**	Maternal	7.29E-06	Chr6:46111054C>A	NM_014936.5: c.1039C>A: p.P347T	**29.3**	**5.9**	**39.47**	**D**	**D**	Male, choreoathetoid quad, born 40 weeks, MRI: haemorrhagic gliosis, known COL4A1 mutation from diagnostic WES, cystic porencephaly involving basal ganglia; damage to small blood vessels with increased risk of haemorrhage; Epileptic encephalopathy, infantile spasms onset 1 year, evolution into mixed seizure types now; unexplained irritability; no speech or communication, global DD; tracheobronchomalacia; laryngomalacia; gastrostomy; fundoplication; scoliosis; left ventricular hypertrophy and hypertension; enlarged left kidney >10% discrepancy; dental caries; sleep apnoea; hip surgery, past femoral osteostomies; chronic lung disease; failure to thrive; hypoplastic optic nerves, MRI: haemorrhagic gliosis.
**EPHA1**	**P744**	Maternal	1.63E-05	Chr7:143096819C>T	NM_005232.5: c.760G>A: p.E254K	**26.7**	4.33	50.99	**D**	**D**	Female, spastic/dystonic quad, born 41 weeks, BW: 1st percentile IUGR, antenatal seizures, brain malformation, ID, Ep, cataracts both eyes. MRI: Extensive right perisylvian, posterior frontal and parietal polymicrogyria. Polymicrogyria involving the parietal lobe and sylvian fissure on the left with superimposed previous insult, presumed ischaemia to the thalamus and external capsule on this side with generalised reduction in volume and size of the supplying vessels.
	**P789**	Not maternal	3.66E-05	Chr7:143095028G>A	NM_005232.5: c.1600C>T: p.R534W	**34**	3.82	**47.13**	**D**	**D**	Male, spastic hemi, born 40 weeks, antenatal seizures, Ep with complex partial seizures, right homonymous hemianopia; right sided visual defects; learning difficulties, mother polycystic kidney disease. CT: large old infarct involving the left MCA territory with

											associated ex vacuo dilatation of the left lateral ventricle. No identifiable hydrocephalus.
**ERLIN1**	**10411**	Unknown	0	Chr10:101937925C>T	NM_006459.4: c.269G>A: p.R90Q	**32**	**5.23**	**23.40**	P	**D**	Male, born 38 weeks, cousin with CP
**GAD1**	**P176**	Not maternal	4.06E-06	Chr2:171702037A>G	NM_000817.3: c.773A>G: p.Y258C	**26.3**	**5.67**	**33.11**	**D**	**D**	Male, spastic hemi, born 24 weeks, porencephalic cyst, Ep, global DD, mild ASD, limited speech, Left hand with increased tone; left ankle/foot orthotic; independent walker. MRI: IVH grade IV.
	**10635**	Not maternal	1.65E-05	Chr2:171709247C>T	NM_000817.3: c.1208C>T: p.P403L	**34**	**5.91**	**20.17**	**D**	**D**	Female, born 39 weeks, Ep, severe ID, congenital bilateral cataracts, soft cleft palate.
**GANC**	**P172**	Unknown	0	Chr15:42618608G>T	NM_198141.2: c.1406G>T: p.G469V	**27.6**	**5.38**	**39.89**	**D**	**D**	Female, spastic diplegia, born 35 weeks, acute disseminated encephalomyelitis; CNS demyelination in infancy (leukodystrophy/ leukoencephalyopathy), obstructive sleep apnoea - adenoids removed.
**GCDH**	**P720**	Not maternal	4.06E-06	Chr19:13002704G>A	NM_000159.4: c.187G>A: p.D63N	**32**	**5.17**	85.19	P	**D**	Male, spastic hemi with some dystonia in left upper limb, born 40 weeks, intermittent right knee pain and headaches. MRI: Periventricular white matter loss and areas of abnormal signal in the periventricular white matter, consistent with PVL. Asymmetry in the size of the thalami.
**HSPG2**	**P008**[1]	Maternal	0	Chr1:22182033G>T	NM_005529.7: c.837C>A: p.A1946D	**29.3**	**5.12**	**7.39**	**D**	**D**	Male, spastic triplegia, born 35 weeks, right orchidopexy, constipation.
	**P049**[1]	Compound het/ Paternal	0	Chr1:22157706A>T[2]	Splicing NM_005529.7: c.11562+2T>A	24.7	**5.46**	.	.	**D**	Male, spastic hemi, born 35 weeks, BW: 5th percentile IUGR, MRI: Slight ventricle asymmetry, two small subependymal nodules on the lateral wall of the right lateral ventricle pointing to minor variant neuronal migration disorder (subependymal heterotopia), some asymmetry of silvian fissures but no definite polymicrogyria or pachygyria.
		Compound het/	4.07E-06	Chr1:22170696G>T[2]	NM_005529.7: c.8561C>A:	24.3	4.43	55.91	P	**D**	As above

	Maternal			p.A2854D						
**P067**[1]	Maternal	4.07E-06	Chr1:22181376G>A	NM_005529.7: c.6098C>T: p.T2033I	**25.6**	4.58	**20.84**	P	**D**	Female, spastic diplegia, born 27 weeks, US: bilateral grade II IVH, then PVL. MRI: PVL. Bilateral increase in white matter signal in the periventricular regions most marked in the occipital areas and centrum semiovale regions consistent with mild changes of periventricular leukomalacia with minor thinning of the corpus callosum posteriorly.
**P120**	Maternal	0	Chr1:22206710A>C	NM_005529.7: c.2233T>G: p.C745G	**25.1**	**5.38**	88.73	**D**	**D**	Male, spastic quad, DD, born 38 weeks, BW: 9th percentile IUGR, Ep, Visual impairment, physical impairment, hypothyroidism, inability to control body temperature
**P200**	Unknown	4.71E-05	Chr1:22172639G>A	NM_005529.7: c.8426C>T: p.T2809I	23.7	4.29	81.15	P	N	Female, spastic hemi, born 36 weeks, mother auto-immune thrombocytopenia, IVH with hydrocephalus diagnosed at 32 weeks, high frequency hearing loss, expressive speech and language delay; gross/fine motor deficit; asthma; lazy eye; bilateral nasolacrimal duct obstruction. US at birth: dilatation of both lateral ventricles, the left more than the right. Third and fourth ventricles are of normal calibre. No IVH identified. Frontal horn of left lateral ventricle dilated and small cystic structure seen superior to the frontal horn with a membrane adjacent to it in keeping with a periventricular cyst. Lateral to the frontal horn of the left lateral ventricles are some smaller cystic areas, these appearances are suggestive of some PVL.
**P212**	Maternal	0	Chr1:22200903G>A	NM_005529.7: c.3652G>A: p.G1218S	22.7	4.14	63.72	**D**	**D**	Female, spastic hemi, born 25 weeks, chorioamnioitis, PDA, mild thoracic scoliosis. MRI: Focal periventricular leucoencephalomalacia in the posterior right frontal lobe. Adjacent dilatation of the lateral ventricle but no

										hydrocephalus or other signal changes within the periventricular white matter.
**P215**	Paternal	6.52E-05	Chr1:22188281C>T	NM_005529.7: c.4924G>A: p.E1642K	24.6	**5.14**	**30.31**	**D**	**D**	Female, spastic diplegia, born 31 weeks, DD, visual problems, Noonan syndrome, cousin ASD, uncle ADHD, squint; short stature, faints; insomnia; GORD; dental caries related to chronological hypoplasia and enamel development defect; pulmonary valve stenosis; IgA deficiency; hypoglycaemia; early iron deficiency with microcytosis but not anaemia. MRI: evidence of PVL.
**P772**	Maternal	8.31E-05	Chr1:22178609C>T	NM_005529.7: c.6842G>A: p.R2281H	**28.5**	**5.34**	88.52	**D**	**D**	Male, spastic/dystonic quadriplegic CP - very dystonic with lots of involuntary dsykinetic movements, born 37 weeks, DD, arthrogryposis affecting his lower limbs (mother also has arthrogryposis)
**P795**	Unknown	7.82E-06	Chr1:22181916C>G	NM_005529.7: c.5878G>C: p.V1960L	**25**	4.17	**27.91**	P	**D**	Female, spastic/dystonic diplegia, born 25 weeks, chronic lung disease from prematurity which required home oxygen; Ep; auditory processing and language problems; anxiety disorder, Continues to have trend for internal rotation at hips with some knee flexion and anterior pelvic tilt bilaterally; quite significant dystonia in calves.
**P1146**	Maternal	3.81E-05	Chr1:22190679C>T	NM_005529.7: c.4654G>A: p.G1552R	**29.7**	**5.1**	**0.57**	**D**	**D**	Male, spastic quad, twin, born 27 weeks, Ep (grandmother also), DD, anxiety/depression, visual problems (mother and grandparent also). CT/MRI: Neonatal ventricular haemorrhage. Shunted hydrocephalus. The changes are consistent with parenchymal gliosis particularly involving the right cerebral hemisphere and to a lesser extent involving the left cerebral hemisphere deep white matter and the left cerebellar hemisphere.
**10301**	Unknown	8.66E-05	Chr1:22168076T>C	NM_005529.7: c.9284A>G: p.N3095S	23.8	4.92	82.38	**D**	**D**	Female, born 39 weeks, BW: 2nd percentile IUGR

	**10941**	Not maternal	1.81E-05	Chr1: 22156014G>A	NM_005529.7: c.11854C>T: p.R3952C	**35**	**5.37**	**18.62**	**D**	**D**	Female, born 27 weeks, GORD
**HUWE1**	**P915**	Not maternal, also present in twin	5.60E-06	ChrX:53596709A>T	NM_031407.7: c.6391T>A: p.L2131M	23.4	4.38	60.72	**D**	**D**	Female, spastic hemiplegia, born 35 weeks, BW: 6th percentile IUGR, identical twin, Ep, visual problems (Mother also), TTTS, plethora, phototherapy, polycythaemia. MRI: decreased volume of the right cerebral hemisphere and dilation of the posterior bodies of both lateral ventricles. Focal area of encephalomalacia in the right cerebral hemisphere in the right frontal parietal region adjacent to the central sulcus.
	**P1102**	Paternal	1.69E-05	ChrX:53579791T>A	NM_031407.7: c.8558A>T: p.E2853V	24.6	**5.88**	87.60	**D**	**D**	Female, spastic quad, born 40 weeks, BW: 5th percentile IUGR, neonatal seizures, OCD (mother also), parents both visual problems, hearing loss (grandparents also), father mainstream school in special class, MRI and US: remote parenchymal insult sustained in the perinatal period.
	**10249**	Unknown	5.60E-06	ChrX:53596600G>A	NM_031407.7: c.6500C>T: p.A2167V	**25.1**	**5.24**	81.23	B	**D**	Female, hemiplegia, born 42 weeks, BW: 1st percentile IUGR, Ep, dandy walker malformation, Mother respiratory arrest at 16 weeks (asthma).
**IGSF1**	**P051**[1]	X-linked	6.15E-05	ChrX:130413296T>A[2]	NM_001170962.1: c.1639A>T: p.T547S	22.5	**5.04**	73.72	**D**	N	Male, mild spastic hemi, born 39 weeks, some articulation problems. US: Mild dilation of the anterior horn of the right lateral ventricle, consistent with a porencephalic cyst; area of the cystic encephalomalacia which has communicated with the anterior horn of the right lateral ventricle.
**INHBB**	**P058**[1]	De novo	8.21E-06	Chr2:121106698C>T	NM_002193.4: c.472C>T: p.R158C	**26.2**	**5.09**	**30.77**	B	**D**	Male, spastic quad, born 38 weeks, mild ID, Epilepsy, DD, congenital CMV infection, generalized polymicrogyria, several febrile seizures
**IQSEC2**	**P1147**	Paternal	0	ChrX:53280005G>A	NM_001111125.3: c.1753C>T: p.R585W	**34**	**5.37**	84.99	**D**	**D**	Female, spastic quad, born 24 weeks, BW: 2nd percentile IUGR, dizygotic twin, hydrocephalus with VP shunt, Ep with

											seizure onset at 9 years, visual problems (both parents also).
**KANK1**	**P188**	Unknown	1.22E-05	Chr9:732430G>A	NM_153186.5: c.2584G>A: p.E862K	24.3	**5.64**	64.27	**D**	**D**	Female, spastic quad with some evidence of dyskinesia, born 37 weeks, BW: 1st percentile IUGR, induced birth due to oligohydramnios and IUGR, diagnosed neonatal encephalopathy, hypoglycaemic day 2 and lactic acidosis on initial cord bloods; apnoeic episodes/seizures day 9; stiff abnormal flexion/extension and fine tremor; difficulties maintaining midline orientation. No evidence of encephalopathy until onset of apnoea and seizures. Moderate hearing deficits, squint and cortical visual impairment; cranial nerve palsies, GORD, feeding difficulties; vomiting and slow weight gain, lactose intolerant, recurrent otitis media and tonsillitis, BPD (maternal Grandmother also). MRI: extensive injury of frontoparietal white matter basal ganglia and thalamic nuclei; symmetric hypoxic brain injury.
	**P200**	Unknown	0	Chr9:731250G>C	NM_153186.5: c.2515G>C: p.V839L	22.9	4.62	58.66	B	P	Female, spastic hemi, born 36 weeks, mother auto-immune thrombocytopenia, IVH with hydrocephalus diagnosed at 32 weeks, high frequency hearing loss, expressive speech and language delay; gross/fine motor deficit; asthma; lazy eye; bilateral nasolacrimal duct obstruction. US at birth: dilatation of both lateral ventricles, the left more than the right. Third and fourth ventricles are of normal calibre. No IVH identified. Frontal horn of left lateral ventricle dilated and small cystic structure seen superior to the frontal horn with a membrane adjacent to it in keeping with a periventricular cyst. Lateral to the frontal horn of the left lateral ventricles are some smaller

										cystic areas, these appearances are suggestive of some PVL.
	**P701**	Not maternal	2.89E-05	Chr9:730224G>A	NM_153186.5: c.2398G>A: p.D800N	23.5	**5.91**	**4.94**	**D**	**D**	Male, spastic/dystonic diplegia, born 40 weeks, cerebral oedema, diagnosed neonatal encephalopathy, required anticonvulsants as neonate, DD, partial Ep seizure at 3yo, non-verbal. US: ventricles and the extra axial spaces are decreased in size most likely due to generalised cerebral oedema. No intracranial haemorrhage demonstrated.
	**P721**	Maternal	0	Chr9:738430G>A	NM_153186.5: c.3005G>A: p.G1002D	**33**	**5.37**	**39.50**	**D**	**D**	Male, spastic/dystonic quad, dysphonia, breathing difficulty, progressive planovalgus deformity
	**10528**	Maternal	8.14E-06	Chr9:711556C>T	NM_153186.5: c.316C>T: p.R106C	**33**	4.89	**5.02**	**D**	**D**	Male, born 40 weeks, DD, Ep
	**10572**	Unknown	3.23E-05	Chr9:742272C>T	NM_153186.5: c.3290C>T: p.A1097V	**33**	**5.38**	65.02	**D**	**D**	Female, born 39 weeks, DD, developmental motor dyspraxia, scoliosis, ASD, depression
	**15065**	Not maternal	9.02E-05	Chr9:745219G>A	NM_153186.5: c.3569G>A: p.R1190Q	**35**	**5.79**	**34.56**	**D**	**D**	Male, born 26 weeks, twin
**KDM5C**	**P026**[1]	De novo	0	ChrX:53240002G>A[2]	NM_001146702.1: c.1238C>T: p.P413L	**28.6**	**5.42**	**3.28**	**D**	**D**	Female, spastic quad with greater involvement of lower limbs, born 39 weeks, BW: 5th percentile IUGR, small stature, convergent strabismus, ridging metopic suture, mild trigonocephaly, severe behaviour problems, speech dyspraxia, photophobia
**KDM7A**	**P105**[1]	De novo	0	Chr7:139796549G>C[2]	NM_030647.2: c.C2180C>G: p.S727W	**34**	**5.85**	**36.31**	**D**	**D**	Female, spastic/dystonic quad, born 38 weeks, BW: 5th percentile IUGR, more signs on right than left, more dystonia than spasticity, born at 38 weeks after IUGR, neonatal hypoglycaemia, and subsequent neonatal seizures. ID, Ep with focal epileptiform abnormality, focal seizures starting at day 7, febrile clonic tonic seizure at 2 years. US: echogenicity of periventricular white matter and left thalamus. MRI: extensive

											PVL, Haemorrhagic foci as well as foci of restricted diffusion, majority of the pathology is likely to have occurred prior to delivery.
	**11348**	Unknown	1.22E-05	Chr7:139829368C>A	NM_030647.2: c.484G>T: p.V162F	**32**	**5.37**	**20.59**	**D**	**D**	Male, born 39 weeks, DD, Ep, visual loss (optic nerve loss)
**KIF1A**	**P174**	Not Maternal	0	Chr2:241727535G>A	NM_004321.7: c.296C>T: p.T99M	**31**	4.79	**5.96**	**D**	**D**	Male, spastic quad, born 40 weeks, partial dysgenesis of corpus callosum, global DD, Ep (first seizure at 12 months), optic atrophy (cortically blind); type 1 diabetes, scoliosis in the lumbar region and an oblique pelvis, incontinent of faeces and urine, cortical atrophy has been progressive.
	**P781**	De novo	0	Chr2:241715280G>A	NM_004321.7: c.C946T: p.R316W	**32**	2.35	**1.63**	**D**	**D**	Female, spastic/dystonic diplegia, more dystonic than spastic with intermittent hypertonicity, born 35 weeks, BW: 10th percentile, SGA, microcephaly, developmental disorders and cortical visual impairment, MRI and X-Ray: Non-specific hyperintensity within the posterior parietal deep white matter, and in the cerebelli, posterior to the dentate nuclei. Bilateral cortical heterotopia in the inferior occipital horns.
	**10078**	Not maternal	0	Chr2:241656789G>A	NM_004321.7: c.5065C>T: p.R1689W	**28.5**	-8.94	89.47	**D**	**D**	Female, father's second cousin CP, asthma and eczema.
**KIF1C**	**P177**	Paternal	5.05E-05	Chr17:4927405C>T	NM_006612.6: c.3271C>T: p.R1091C	**32**	**5.04**	96.60	**D**	**D**	Male, spastic/dyskinetic hemi, born 41 weeks, right affected leg; dragging it; 1cm difference in length, has difficulties with balance on the right hand side. MRI: evidence of gliosis/encephalomalacia in the left perisylvian region involving the left lentiform nucleus; caudate; and corona radiata. Ex-vacuo dilation of left lateral ventricle and diminuition of periventricular white matter, consistent with old area of infarct in the left

											MCA/lateral lenticulostriate territory.gliosis/encephalomalacia in left perisylvian region involving the left lentiform nucleus, caudate, and corona radiata
	**P215**	Paternal	8.941E-05	Chr17:4924169G>A	NM_006612.6: c.2006G>A: p.R669Q	**34**	**5.68**	75.36	**D**	**D**	Female, spastic diplegia, born 31 weeks, DD, visual problems, Noonan syndrome, cousin ASD, uncle ADHD, squint; short stature, faints; insomnia; GORD; dental caries related to chronological hypoplasia and enamel development defect; pulmonary valve stenosis; IgA deficiency; hypoglycaemia; early iron deficiency with microcytosis but not anaemia. MRI: evidence of PVL.
	**P758**	Not maternal	5.69E-05	Chr17:4905376G>A	NM_006612.6: c.386G>A: p.R129H	**27.7**	**5.45**	**20.11**	**D**	**D**	Male, spastic diplegia, born 40 weeks, ASD (cousin also), hearing loss (grandmother also), MRI: open lipped schizencephaly on the right and either transmantle heterotopia or closed lip schizencephaly demonstrated on the left, associated with large extra-axial CSF spaces. Unclear whether these are simply secondary to the malformation or represent arachnoid cysts. Suggestion of some mass effect on the left side with enlargement of the middle cranial fossa. Multiple areas of polymicrogyria, absence of the septum pellucidum and small optic nerves.
	**P759**	Maternal, no sample for Father	3.17E-05	Chr17:4927186C>T homozygous	NM_006612.6: c.3052C>T: p.R1018C	**29.6**	3.76	89.22	**D**	N	Male, spastic hemi, born 37 weeks.
	**P1108**	Not maternal	8.94E-05	Chr17:4924169G>A	NM_006612.6: c.2006G>A: p.R669Q	**34**	**5.68**	75.36	**D**	**D**	Female, spastic/dystonic hemi, born 39 weeks, Focal dyscognitive seizures, left convergent squint. MRI/CT: appearance is in keeping with a large porencephalic cyst in the left middle cerebral artery. White matter loss with dilated left lateral ventricle consistent with a remote insult.

**L1CAM**	**P724**	X-linked	0	ChrX:153132565G>A	NM_001143963.2: c.2137C>T: p.P713S	**27.2**	**5.77**	65.89	**D**	**D**	Male, spastic diplegia, born 40 weeks, BW: 2nd percentile IUGR, spastic paraparesis and agenesis of the corpus callosum, macrocephaly, X-linked hydrocephalus, Mother and 3 aunts are carriers, MRI and CT: Absent corpus callosum with associated colpocephaly, hypoplasia of the cortical mantle and gross ventriculomegaly. The cerebral aqueduct is narrow, and the dilated third and lateral ventricles are suggestive of aqueduct stenosis.
**MAN2A1**	**P414**[1]	De novo	0	Chr5:109159498T>A[2]	NM_002372.3: c.2526T>A: p.F842L	17.4	4.37	69.52	B	**D**	Male, spastic hemi, born 40 weeks, Von Willebrand disease, Ep.
**MAOB**	**P025**[1]	X-linked	0	ChrX:43634520C>T[2]	Splicing NM_000898.5: c.1138-1G>A	23.8	**6.17**	.	.	**D**	Male, spastic hemi, born 40 weeks, severe sleep disturbance, anxiety issues, behaviour problems, Irregularity and dilation of the left lateral ventricle associated with periventricular white matter volume loss and thinning of the corpus callosum.
	**P216**	X-linked	0	ChrX:43640740G>A	NM_000898.5: c.980C>T: p.T327M	**32**	**5.47**	**25.58**	**D**	**D**	Male, spastic quad, born 40 weeks, BW: 9th percentile IUGR, DD, kyphoscoliosis, visual problems (mother also), progressive muscle weakening, seizures as neonate, Ep as child.
	**P915**	Not maternal, also present in twin	0	ChrX:43640740G>A	NM_000898.5: c.980C>T: p.T327M	**32**	**5.47**	**25.58**	**D**	**D**	Female, spastic hemiplegia, born 35 weeks, BW: 7th percentile IUGR, identical twin, Ep, visual problems (Mother also), TTTS, plethora, phototherapy, polycythaemia. MRI: decreased volume of the right cerebral hemisphere and dilation of the posterior bodies of both lateral ventricles. Focal area of encephalomalacia in the right cerebral hemisphere in the right frontal parietal region adjacent to the central sulcus.
**MAST1**	**P009**[1]	De novo	0	Chr19:12975755C>T[2]	NM_014975.3: c.1499C>T: p.P500L	**32**	4.39	**3.63**	**D**	**D**	Male, spastic diplegia, born 33 weeks, resolved atrial septal defect, alternating squint and longsighted, white matter

											damage, mild learning problem, behavioural problems, sensitive hearing, drooling; dis/subluxation hips; cardiac incomplete right bundle branch block; mild pulmonary stenosis; asymmetrical gait pattern and short stride length, MRI: mild generalised volume loss, PVL, cystic encephalomalacia.
	**P773**	Not maternal	8.12E-06	Chr19:12963198G>A	NM_014975.3: c.1066G>A: p.D356N	23.2	**5.6**	70.89	P	**D**	Male, spastic diplegia, clumsy gait, born 28 weeks, ADHD, anxiety/depression (father also), bipolar disorder in 2x paternal aunts and grandmother, mother's cousin spina bifida, maternal aunt cleft lip.
	**14986**	Paternal	1.22E-105	Chr19:12958197G>A	NM_014975.3: c.421G>A: p.E141K	**28.3**	**5.01**	80.57	**D**	**D**	Male, ataxic CP, born 39 weeks, BW: 4th percentile IUGR, DD, speech difficulties, intention tremor, OCD, ASD features, anxiety, paternal grandfather early onset alzheimers, father possible milder speech and coordination problems.
**MCCC1**	**P016**[1]	Paternal	7.22E-06	Chr3:182755152T>C	NM_001293273.1: c.1097A>G: p.N366S	24.4	4.75	**12.26**	P	**D**	Male, spastic quad, born 40 weeks, BW: 2nd percentile IUGR, Ep onset at 6 mths, tonic-clonic seizures 2-3 times per day; swallowing difficulties; severe ID; communicates non-verbally, swallowing difficulties, delivery complicated by significant foetal distress resulting in precipitous delivery using forceps with poor apgars.
	**P719**	Not maternal	0	Chr3:182763308C>T	NM_001293273.1: c.625G>A: p.D209N	**27.9**	**5.63**	**24.24**	**D**	**D**	Female, spastic hemi with dysarthria, born 36 weeks, BW: 2nd percentile IUGR, Ep, severe drooling, polycythaemia, DD and right visual field defect, unilateral cerebral thrombotic infarction. MRI: Large area of cystic encephalomalacia in the left MCA territory with significant attenuation of the left MCA and its branches. Small area of cystic encephalomalacia in the right MCA territory. Severe PVL with evidence of middle cerebral artery occlusion on the left side.

	**P785**	Unknown	0	Chr3:182733339G>C	NM_001293273.1: c.1714C>G: p.P572A	24.5	4.84	61.30	**D**	**D**	Female, spastic hemi, poor balance, speech delay, swallowing difficulties, vomits easily, leg lengths differ by 1cm
	**10049**	Unknown	3.25E-05	Chr3:182756852C>A	NM_001293273.1: c.988G>T: p.A330S	**29.2**	**5.82**	**31.96**	**D**	**D**	Female
	**10768**	Unknown	3.25E-05	Chr3:182756852C>A	NM_001293273.1: c.988G>T: p.A330S	**29.2**	**5.82**	**31.96**	**D**	**D**	Male, born 40 weeks, DD, Ep, hypospadias and chordee of penis
**MCPH1**	**P010**[1]	Paternal	3.61E-05	Chr8:6479017G>A	NM_024596.5: c.2257G>A: p.G753R	**27.9**	4.45	**4.31**	**D**	**D**	Male, spastic diplegia, born 40 weeks, severe ID, ASD, global DD, visual impairment suspected of cerebral origin with roving eyes and visual inattention slightly down-slanting palperbral fissures, self-injuring behaviour, sleep disturbances, non-verbal apart from echolalia with bizarre vocalisations, Mother Ep. MRI: Congenital hydrocephalus associated with aqueduct stenosis. Significant loss of white matter presumable secondary to hydrocephalus. Cortical visual impairment. X-ray: abnormal appearance of skull with a copper-beaten appearance, narrowing of the foramen magnum and impression of superior migration of the posterior portion of the occipital bone.
	**P027**[1]	Maternal	2.03E-05	Chr8:6357439C>T	NM_024596.5: c.2203C>T: p.P735S	**34**	**5.78**	**14.59**	**D**	**D**	Male, spastic quad, born 29 weeks, mother had pre-eclampsia. DD, mild ID, BW: <1 percentile IUGR, respiratory distress syndrome, impaired hearing; umbilical and inguinal hernias; short stature (<3rd percentile) from birth onwards and underweight (3rd-10th percentile). At birth head circumference was 3.9th percentile for gestational age, and 5th percentile at 7 years. US: Initial ultrasounds at 2 months were normal, but subsequent cranial ultrasounds showed bilateral parietal-occipital PVL (L>R).

**MGA**	**P427**[1]	Maternal	6.79E-04	Chr15:42035028A>G	NM_001164273.1: c.4870A>G: p.K1624E	**25.2**	4.96	**31.20**	P	**D**	Male, spastic hemi, born 38 weeks.
	**P707**	Unknown	1.66E-04	Chr15:42034996C>T	NM_001164273.1: c.4838C>T: p.T1613I	23.4	4.96	50.50	P	**D**	Female, spastic/dyskinetic quad, born 24 weeks, hyperkinetic, Hyaline Membrane Disease (Respiratory Distress Syndrome) after birth; required surgery for patent ductus. Necrotising enterocolitis as infant
		Unknown	1.12E-04	Chr15:42019504T>C	NM_001080541.2: c.3557T>C: p.L1186S	22.8	4.71	**18.94**	P	N	As above
	**P776**	Unknown	4.06E-05	Chr15:42040936C>T	NM_001080541.2: c.4687C>T: p.P1563S	24.3	4.76	93.24	B	**D**	Male, asymmetric spastic diplegia, born 36 weeks, BW: 5th percentile IUGR, DD, ASD, ID and seizure disorder with consequent behaviour disturbances and aggressive behaviour. MRI: Bilateral periventricular white matter volume loss with signal changes as desribed, consistent with PVL. Mild cerebral cerebellar degenerative changes noted.
	**10596**	Unknown	4.06E-05	Chr15:42040936C>T	NM_001080541.2: c.4687C>T: p.P1563S	24.3	4.76	93.24	B	**D**	Male, born 29 weeks, Mother rheumatic fever during pregnancy
	**10908**	Unknown	8.16E-06	Chr15:42035281C>T	NM_001164273.1: c.5123C>T: p.S1708F	**26.8**	**5.11**	53.31	**D**	**D**	Female, born 29 weeks, nerve deafness, Father's first cousin mild CP
	**11039**	Maternal	4.88E-05	Chr15:42034954C>T	NM_001164273.1: c.4796C>T: p.T1599I	**26.8**	4.96	84.86	**D**	**D**	Male, spastic hemi, born 34 weeks, DD, Ep, velocardiofacial syndrome, tetralogy of fallot
**MIIP**	**P059**[1]	Maternal	0	Chr1:12082422delG	F/shift del NM_021933.3: c.385delG: p.Arg130Glyfs*28	.	.	.	.	.	Male, spastic diplegia, born 25 weeks, ASD, DD, vision impaired, mild ID, anxiety, severe language disorder characterised by difficulties understanding and using language, echolalia, short stature and low weight at 1 year, expression of MIIP downregulated [7], US: bilateral germinal matrix haemorrhages, probably extending into the ventricle on both side, with cystic change. CT: ventricular dilatation and irregularity of the

										posterior aspects of the lateral ventricles in keeping with end stage PVL.
**P454**[1]	De novo	0	Chr1:12082151G>A[2]	Splicing NM_021933.3: c.115-1G>A, d/stream cryptic splice site used, delTCAGG in cDNA, f/shift del	15.47	3.47	.	.	.	Male, spastic diplegia, born 41 weeks, paternal cousin with spastic diplegia.
**P719**	Maternal	0	Chr1:12090147G>T	NM_021933.4: c.908G>T: p.S303I	**26**	4.01	81.23	P	**D**	Female, spastic hemi with dysarthria, born 36 weeks, BW: 2nd percentile IUGR, Ep, severe drooling, polycythaemia, DD and right visual field defect, unilateral cerebral thrombotic infarction. MRI: Large area of cystic encephalomalacia in the left MCA territory with significant attenuation of the left MCA and its branches. Small area of cystic encephalomalacia in the right MCA territory. Severe PVL with evidence of middle cerebral artery occlusion on the left side.
**P746**	Unknown	8.13E-06	Chr1:12089176G>A	NM_021933.4: c.634G>A: p.E212K	**33**	**5.28**	**41.90**	**D**	**D**	Female, spastic diplegia, difficulty with distant vision, planovalgus foot position
**P788**	Unknown	0	Chr1:12090086G>C	NM_021933.4: c.847G>T: p.V283L	24.8	**5.15**	**27.76**	P	**D**	Male, dystonic quad, severe dysphagia, Ep, DD (brother/sister also), mother, brother visual problems, CT/MRI: postnatal herpetic meningoencephalitis
**P799**	Maternal	0	Chr1:12090086G>C	NM_021933.4: c.847G>T: p.V283L	24.8	**5.15**	**27.76**	P	**D**	Male, spastic hemi, born 38 weeks, Ep, ASD, ADHD, OCD, anxiety/depression, increasing aggression, microcephaly, DD, various visual problems including hemianopia and optic nerve damage. MRI 2 months: Right middle cerebral artery infarct with changes of porencephaly. Smaller capsular and basal ganglionic infarcts on the left side. Diffuse and generalised cortical volume loss. Thin and hypoplastic right MCA. MRI 4 years: gliotic change in the left periventricular region surrounding the

											encephalomalacic cavity has increased and evidence of wallerian degeneration has developed in the right thalamus. Extensive right middle artery terriotry cystic encephalomalacia and small right middle cerebral artery changes are stable. MRI 11 years: Extensive cystic encephalomalacia and gliosis in the right middle cerebral artery territory does not show significant interval change. Wallerian degeneration involving the bilateral thalami and brainstem as described. Bilateral paranasal sinus disease.
	**P1108**	Not maternal	4.51E-05	Chr1:12082359C>T	Stopgain NM_021933.4: c.322C>T: p.R108*	**35**	1.32	**10.80**	.	**A**	Female, spastic/dystonic hemi, born 39 weeks, Focal dyscognitive seizures, left convergent squint. MRI/CT: appearance is in keeping with a large porencephalic cyst in the left middle cerebral artery. White matter loss with dilated left lateral ventricle consistent with a remote insult.
**MTMR1**	**P203**	X-linked	0	ChrX:149898672C>G	NM_001306145.1: c.623C>G: p.S208C	24	**5.35**	**13.74**	P	**D**	Male, spastic hemi, born 38 weeks, congenital microcephaly, plagiocephaly (moderate), cortical dysplasia; failure to thrive; microcrania, primordial smallness, sensorineural hearing loss; developmental delay; prominent expressive language delay; aspiration; lung disease (resolved); swallowing difficulties. MRI at 3 years: Extensive cortical dysplasia, with white matter changes likely to be associated with underlying dysplasia rather than representing a dysmyelinating process. MRI at 6 years: Appearances favour a low-grade glioma involving the left cerebellar hemisphere in a parasagittal location in the peri-dentate white matter on a background of bifrontoparietal polymicrogyria. MRI at 9 years: Cerebellar lesion excised at 8

											years. Cystic cavity within the left cerebellar hemisphere has decreased in size; alteration in enhancement pattern of this resected section is noted, was previously rim enhancing, however, now demonstrates nodular enhancement. No haemorrhage or mass identified.
**MYO15A**	**P109**[1]	Maternal	2.51E-05	Chr17:18070991G>A	NM_016239.4: c.10036G>A: p.A3346T	**26**	**5.61**	53.26	**D**	**D**	Male, spastic hemi, born 35 weeks, BW: 2nd percentile IUGR, history of in utero CVA, Ep, obesity at 8 yrs; DD, significant learning difficulties, left homonymous hemianopia. CT: large right MCA territory infarct, in utero CVA. Extensive hypodensity involving vast majority of under developed right hemisphere and associated ex-vacuo dilatation of the right lateral ventricle. Right side of the calvarium is underdeveloped compared to the left. Extensive change to the right hemisphere.
	**P113**[1]	Paternal	0	Chr17:18051498T>A	NM_016239.4: c.6665T>A: p.M2222K	24.1	4.1	**2.36**	**D**	**D**	Male, spastic/dyskinetic quad, born 40 weeks, hypotonia in all 4 limbs, mother collapsed with possible amniotic fluid embolus, child delivered somewhat hypoxic. Kyphosis, sialorrhea with drooling, nose bleeds, undescended testes, ID, DD, gastrostromy fed, short stature (<3rd percentile at 6 years). US: normal. MRI: bilateral symmetric signal changes in the lentiform nuclei and perirolandic cortex.
	**P115**[1]	De novo	0	Chr17:18051510T>C[2]	NM_016239.4: c.6677T>C: p.V2226A	23.6	4.1	**5.41**	**D**	**D**	Male, spastic diplegia, born 41 weeks, moderate ID; Ep (mostly complex partial seizures, 3-5 per month); ASD; expressive speech delay; severe visual impairment, optic nerve atrophy; adducted thumbs; behavioural problems but generally sociable and happy. US: congenital tetraventricular hydrocephalus, MRIs: hydrocephalus communicating with persistent Blake's pouch cyst, cerebellar folia

										disorganisation, white matter volume loss, partial agenesis of the corpus callosum, abnormal positioning of thalamus and lentiform nucleus, incomplete hippocampal inversion bilaterally, hypoplasia of the optic nerve, hypoplasia of the cerebellar vermis and asymmetry of the pons.
**P743**	Not maternal	1.44E-05	Chr17:18042884C>T	NM_016239.4: c.5170C>T: p.R1724C	**27.9**	3.56	57.27	**D**	**D**	Female, dyskinetic quad, born 26 weeks, DD, hearing loss/deafness, chronic lung problems, progressive scoliosis. MRI: generalised bilateral cerebral atrophy.
**P915**	Unknown	0	Chr17:18045504C>T	Stopgain NM_016239.4: c.5761C>T: p.R1921*	**39**	4.7	.	.	**A**	Female, spastic hemiplegia, born 35 weeks, BW: 7th percentile IUGR, identical twin, Ep, visual problems (Mother also), TTTS, plethora, phototherapy, polycythaemia. MRI: decreased volume of the right cerebral hemisphere and dilation of the posterior bodies of both lateral ventricles. Focal area of encephalomalacia in the right cerebral hemisphere in the right frontal parietal region adjacent to the central sulcus.
**P950**	Maternal	0	Chr17:18077163 delCAGCT	F/shift del NM_016239.3: c.10419_10423 delCAGCT S3474Pfs*42	.	.	.	.	.	Male, spastic hemi, born 40 weeks, ASD, visual problems, antenatal seizures, parents related, relationship not specified, transposition of great arterial vessels, stroke as baby during heart operation.
**P959**	Unknown	2.74E-05	Chr17:18047111G>A	Splicing NM_016239.3: c.6046+1G>A	24.6	4.13	.	.	**D**	Male, spastic/dyskinetic quad, born 39 weeks, BW: <1 percentile IUGR, placenta infarcted. MRI: PVL.
**P963**	Unknown	0	Chr17:18045504C>T	Stopgain NM_016239.3: c.5761C>T: p.R1921*	**39**	4.7	.	.	**A**	Female, spastic hemi, born 29 weeks, CPAP in neonatal period.
**P1131**	Unknown	0	Chr17:18023972G>T	Stopgain NM_016239.4: c.1858G>T: p.E620*	**34**	3.51	**14.55**	.	**A**	Female, spastic triplegic, born 24 weeks, twin, required resuscitation at birth, IQ 52, IVH and secondary hydrocephalus requiring shunting before 6 months.

	**10218**	Unknown	3.61E-05	Chr17:18066565G>A	NM_016239.4: c.9620G>A: p.R3207H	**25.1**	**5.25**	93.60	**D**	**D**	Female, born 32 weeks, BW: <1 percentile IUGR, twin
**MYO1F**	**P030**[1]	Compound het/ Maternal	8.14E-06	Chr19:8601410C>T	NM_012335.3: c.1871G>A: p.R624H	**35**	4.83	**37.40**	**D**	**D**	Male, spastic quad, born 37 weeks, BW: <1 percentile IUGR, DD, Ep with myoclonic seizures, cortical visual impairment, cataracts, scoliosis thoraco-lumbar and kyphosis mid thoracic; bilateral undescended testes, non-verbal, communicates with communicator. Experienced episodes of severe bradycardia before onset of labour. Placenta showed multiple infarcts and there was a velamentous insertion of the cord. Had some limb contractures at delivery associated with oligohydramnios. At birth required sedation and ventilation and had evidence of cerebral oedema in neonatal period. MRI: Scaphocephaly of the skull with an elongated narrowed brain. Moderate ventricular dilation more marked on the left with enlarged subarachnoid space. Immaturity in myelination, particularly in temporal lobes and parietal regions. High signal periventricular white matter changes suggesting PVL. Probably subependymal grey matter heterotopias. Hypoplastic thin corpus callosum.
		Compound het/ Paternal	2.54E-03	Chr19:8587401C>T	NM_012335.3: c.3080G>A: p.R1027Q	**25.2**	**5.37**	93.09	**D**	**D**	As above
	**P936**	Not maternal	7.22E-06	Chr19:8618281T>C	NM_012335.3: c.367A>G: p.I123V	23.5	4.35	**25.48**	P	**D**	Male, Spastic diplegia, born 33 weeks, BW: 8th percentile IUGR, 2 sisters with ADHD and anxiety/depression.
	**P1136**	Unknown	0	Chr19:8586455C>T	NM_012335.3: c.3254G>A: p.G1085D	**31**	4.92	51.87	**D**	**D**	Female, spastic hemi, born 40 weeks, DD, Ep, visual impairment. MRI: encephalomalacia involving the left posterior temporal and frontal lobes secondary to old left MCA distribution infarction. This could be as a result of a

											perinatal injury. Wallerian degeneration involving the mid brain and pons.
**NAA35**	**P117**[1]	De novo	0	Chr9:88631481G>A[2]	NM_024635.3: c.1596G>T: p.W532C	**33**	**5.4**	**20.14**	**D**	**D**	Male, spastic hemi, born 39 weeks, borderline ID, refractory focal seizures and a left homonymous hemianopia, unilateral cerebral thrombotic infarction. US: increased echogenicity right thalamus, basal ganglia and deep white matter. MRI: extensive right MCA territory perinatal infarct with extensive encephalomalacia involving the central regions, frontal, parietal and occipital lobes. Right brain size smaller. Cystic changes. Right lateral ventricle dilated. Temporal lobe is of a reduced volume.
	**P783**	Unknown	0	Chr9:88571284T>C	NM_024635.3: c.134T>C: p.L45S	**27**	3.94	**28.97**	**D**	**D**	Male, spastic hemi, born 38 weeks, BW: 2nd percentile IUGR, moderate DD, seizures at birth, tachycardia, hypoglycaemia, parents cousins
**NGFR**	**P101**[1]	De novo	3.28E-05	Chr17: 47589394C>T[2]	NM_002507.4: c.962C>T: p.T321M	**25.3**	**5.18**	70.17	B	**D**	Male, spastic quad, born 40 weeks, parents first cousins, dysphagia, dysarthria, severe dystonia, ID, no speech but communicates well with computer. MRI: Cortical dysplasia. Fronto parietal temporal dysplasia, suggestive of bilateral frontoparietal polymicrogyria.
	**P403**[1]	Paternal	6.48E-05	Chr17:47590297G>A	NM_002507.4: c.1210G>A: p.A404T	**34**	3.49	76.13	**D**	**D**	Male, spastic diplegia, born at 35 weeks, BW: 4th percentile IUGR, strabismus.
**NPC1**	**10697**	Unknown	4.07E-06	Chr18:21115499insT	F/shift ins NM_000271.5: c.3410dupA: p.N1137fs*121	.	.	.	.	.	Male, born 31 weeks, executive function disorder, semantic pragmatic disorder
	**10750**	Not maternal	4.06E-05	Chr18:21112188C>T	NM_000271.5: c.3815G>A: p.R1272H	**28.9**	**5.2**	95.47	**D**	**D**	Male, born 40 weeks, BW: 7th percentile IUGR
**NR1I2**	**P084**[1]	De novo	8.19E-06	Chr3:119534605G>A	NM_003889.3: c.1087G>A: p.D363N	**25.8**	**5.04**	**42.76**	**D**	**D**	Variant not detected by WES, Female, dyskinetic mixed diplegia, born 40 weeks, right side more affected than left. Dyskinetic mixed: bilateral, low apgars at birth. Multi organ failure,

											seizures and need for ventilation at birth, Ep. US: mild cerebral oedema, then increased echogenicity of white matter. MRI: changes of gliosis in thalami bilaterally, posterior limb of the left external capsule and high subcortical white matter of the parietal lobes/corticospinal tract. Appearances are consistent with gliosis from previous hypoxic ischaemic encephalopathy.
	**P443**[1]	De novo	0	Chr3:119534572G>T[2]	Splicing NM_033013.2: c.944-1G>T	24.8	**5.04**	.	.	**D**	Female, spastic hemi, born 39 weeks, maternal uncle severe physical and mental disability.
**NT5C2**	**P718**	Hom - IBD	0	Chr10:104899223G>A	Stopgain NM_001134373.2: c.115C>T: p.R39*	**36**	4.99	.	.	**A**	Female, spastic diplegia, born 36 weeks, advanced juvenile glaucoma, parents first cousins, DD, ASD, moderate ID, ADHD (Brother also), OCD, advanced juvenile glaucoma, severe behavioural problems including unmanageable oppositional behaviour, anxiety, bilateral paranasal sinus disease, MRI: PVL.
**PAK3**	**P015**[1]	X-linked	0	ChrX:110459718C>T[2]	NM_002578.5: c.1477C>T: p.R493C	**34**	**5.54**	97.68	**D**	**D**	Male, dystonic spastic hemiplegia, born 29 weeks, BW: <1 percentile IUGR Ep, cognitive ability upper limit of low-average range, developed hydrocephalus, MRI: grade 4 IVH, right sided and grade 2 IVH left sided
**PCBP3**	**P117**[1]	De novo	4.08E-06	Chr21:47355203T>C[2]	NM_001130141.1: c.815T>C: p.L272P	**29.3**	3.72	70.81	**D**	**D**	Male, spastic hemi, born 39 weeks, borderline ID, refractory focal seizures and a left homonymous hemianopia, unilateral cerebral thrombotic infarction. US: increased echogenicity right thalamus, basal ganglia and deep white matter. MRI: extensive right MCA territory perinatal infarct with extensive encephalomalacia involving the central regions, frontal, parietal and occipital lobes. Right brain size smaller. Cystic changes. Right lateral ventricle dilated. Temporal lobe is of a reduced volume.

	**P802**	Unknown	0	Chr21:47329390A>G	NM_001130141.1: c.461A>G: p.E154G	**33**	4.99	**15.32**	**D**	**D**	Female, spastic/dystonic hemi, born 40 weeks, visual problems.
	**15410**	Maternal	3.97E-05	Chr21:47320983G>A	NM_001130141.1: c.295G>A: p.A99T	**25.9**	4.86	86.13	B	**D**	Female, born 30 weeks, DD
**PROC**	**P052**[1]	Compound het/ Paternal	2.53E-05	Chr2:128178957C>T	NM_000312.3: c.169C>T: p.R57W	**32**	3.33	.	**D**	**D**	Female, spastic/dyskinetic quad, born 38 weeks, ID, Ep, probable Gardner-Diamond syndrome, mild dysphagia, gradual but progressive decline neuro-developmentally, 4th child, 1st and 3rd female siblings deceased from obscure degenerative leukoencephalopathy, MRI: PVL, porencephaly, partial agenesis of corpus callosum (absent genu, rostrum, body with splenium present).
		Compound het/ Maternal	2.53E-05	Chr2:128185950C>T	NM_000312.3: c.814C>T: p.R272C	.	.	**18.40**	**D**	**A**	As above
	**P739**	Not maternal	3.26E-05	Chr2:128186457T>C	NM_000312.3: c.1321T>C: p.Y441H	**27**	**5.16**	68.83	**D**	**D**	Male, spastic/dystonic quad, born 36 weeks, BW: <1 percentile IUGR. MRI: Bilateral nearly symmetrical periventricular white matter hyperintensity, predominantly involving the deep white matter, suggestive of a demyelination/dysmyelination disorder.
**PTGFRN**	**P465**[1]	De novo	2.85E-05	Chr1:117509692A>G[2]	NM_020440.4: c.1799A>G: p.N600S	23.1	**5.56**	71.02	P	**D**	Female, spastic diplegia, born 34 weeks, vanishing twin syndrome, mother placental abruption due to previously unknown clotting disorder.
	**10635**	Not maternal	1.02E-04	Chr1:117491978C>T	NM_020440.4: c.997C>T: p.R333W	**33**	4.59	**48.18**	**D**	N	Female, born 39 weeks, Ep, severe ID, congenital bilateral cataracts, soft cleft palate.
**RFX2**	**P443**[1]	De novo	0	Chr19:6040241T>C[2]	NM_000635.4: c.272A>G: p.Y91C	23.9	**5.36**	80.53	**D**	**D**	Female, spastic hemi, born 39 weeks, maternal uncle severe physical and mental disability.
**RNF214**	**P007**[1]	De novo	0	Chr11:117152637C>T[2]	NM_001077239.1: c.1363C>T: p.P455S	23.4	4.76	97.16	**D**	**D**	Female, mild ataxic / spastic diplegia, born 32 weeks, BW: <1 percentile IUGR, borderline IQ, twin, occipital horns are mildly plump (colpocephaly), behavioural outbursts and temper tantrums.

**SCN8A**	**P904**	De novo, also present in identical twin	4.06E-06	Chr12:52188354C>T	NM_014191.3: c.4724C>T: p.A1575V	**34**	4.94	80.81	**D**	**D**	Male, spastic diplegia, born 28 weeks, identical twin with no known NDD, shared placenta, TTTS, ASD (cousin also), ADHD (cousin also), anxiety/depression, visual problems, MRI: mild PVL.
**SLC6A3**	**P042**[1]*	Not maternal	0	Chr5:1411428G>A	NM_001044.5: c.1199C>T: p.T400M	18.55	4.69	**11.78**	**D**	**D**	Female, spastic hemi, born 30 weeks, BW: <1 percentile IUGR, DD, mild ID, Ep (focal seizures), respiratory distress syndrome, behaviour problems.
	**P082**[1]	Paternal	0	Chr5:1443060G>A	NM_001044.5: c.253C>T: p.R85W	**32**	1.46	**8.54**	**D**	**D**	Female, spastic quad, born 38 weeks, BW: 2nd percentile IUGR, hearing & vision loss, microcephaly, swallowing issues (at risk of aspiration), CMV diagnosed by blood test at 1 year old
**SREK1**	**P436**[1]	De novo	4.09E-06	Chr5:65449298C>T[2]	NM_001077199.3: c.169C>T: p.P57S	18.21	**5.61**	**7.2**	P	**D**	Female, spastic diplegia, BW: 4th percentile IUGR, twin, born 29 weeks
	**10894**	Not maternal	8.12E-06	Chr5:65460661C>T	NM_001077199.3: c.937C>T: p.R313C	24.3	**5.4**	91.68	**D**	**D**	Female, DD, microcephaly, ID, mother tested positive for CMV postnatally, reported IUGR and foetal distress
**SYNGAP1**	**10894**	Not maternal	0	Chr6:33412248C>G	NM_006772.2: c.3436C>G: p.P1146A	23.8	4.75	97.59	**D**	**D**	Female, DD, microcephaly, ID, mother tested positive for CMV postnatally, reported IUGR and foetal distress
**TENM1**	**P026**[1]	Paternal	0	ChrX:123518642C>G[2]	NM_014253.2: c.6118G>C: p.D2040H	23.6	**5.44**	**14.87**	**D**	**D**	Female, spastic quad with greater involvement of lower limbs, born 39 weeks, BW: 5th percentile IUGR, small stature, convergent strabismus, ridging metopic suture, mild trigonocephaly, severe behaviour problems, speech dyspraxia, photophobia
	**10397**	Not maternal	0	ChrX:123518176C>T	NM_014253.2: c.6584G>A: p.R2195Q	**25.4**	**5.52**	88.08	**D**	**D**	Female, born 41 weeks, hydrocephalus, DD, Ep, vision impairment
**TH**	**P067**[1]	Paternal	3.64E-05	Chr11:2192973C>A	NM_000360.3: c.44G>T: p.R15L	**34**	3.87	82.73	**D**	**D**	Female, spastic diplegia, born 27 weeks, US: bilateral grade II IVH, then PVL. MRI: PVL. Bilateral increase in white matter signal in the periventricular regions most marked in the occipital areas and centrum semiovale regions consistent with mild changes of periventricular leukomalacia with minor thinning of the corpus callosum posteriorly.

	**P087**	Paternal, affected sibling does not have variant	8.33E-06	Chr11:2189774A>T	NM_000360.3: c.434A>T: p.L145H	24.2	3.67		**D**	**D**	Male, ataxic CP, born 38 weeks, father and sister also. Mild heart murmur, vitiligo.
**TUBA8**	**P455**[1]	Paternal	0	Chr22:18604342G>C	NM_018943.3: c.100G>C: p.G34R	**31**	**5**	**29.33**	**D**	**D**	Male, spastic diplegia, born 26 weeks.
**UBQLN3**	**P428**[1]	De novo	0	Chr11:5530219del CAGCTGG[2]	F/shift del NM_017481.4: c.564_570del: p.Q188Ffs*10	.	.	.	.	.	Male, spastic diplegia, born 29 weeks, triplet, mild ID.
	**P740**	Not paternal	0	Chr11:5530781T>G	NM_017481.4: c.8A>C: p.K3T	24.8	**5.38**	**41.16**	P	**D**	Male, spastic quad, born 28 weeks, lower limbs significantly more affected than the upper limbs.
	**P919**	Not paternal	4.70E-05	Chr11:5530127A>G	NM_017481.4: c.662T>C: p.L221P	**25**	**5.53**	73.17	P	P	Spastic hemi, born 28 weeks, left grade IV IVH.
**UBXN7**	**P067**[1]	De novo	0	Chr3: 196120470C>T[2]	NM_015562.2: c.310G>A: p.A104T	20.1	4.47	62.04	P	**D**	Female, spastic diplegia, born 27 weeks, US: bilateral grade II IVH, then PVL. MRI: PVL. Bilateral increase in white matter signal in the periventricular regions most marked in the occipital areas and centrum semiovale regions consistent with mild changes of periventricular leukomalacia with minor thinning of the corpus callosum posteriorly.
**USP26**	**P117**[1]	X-linked	0	ChrX:132161343G>C[2]	NM_031907.1: c.906C>G: p.N302K	23.9	0.143	**1.97**	**D**	N	Male, spastic hemi, born 39 weeks, borderline ID, refractory focal seizures and a left homonymous hemianopia, unilateral cerebral thrombotic infarction. US: increased echogenicity right thalamus, basal ganglia and deep white matter. MRI: extensive right MCA territory perinatal infarct with extensive encephalomalacia involving the central regions, frontal, parietal and occipital lobes. Right brain size smaller. Cystic changes. Right lateral ventricle dilated. Temporal lobe is of a reduced volume.
**VRK1**	**P192**	Unknown	8.14E-06	Chr14:97299816G>T	NM_003384.3: c.8G>T:	23.5	**5.03**	79.35	B	**D**	Male, spastic diplegia, born 29 weeks, IVH day 1, respiratory distress, squint

					p.R3L						(estropia) and visual impairment (Mother also), mother thalassemia minor, developing some foot deformities as he is growing. MRI: mild prominence of ventricles, in particular the posterior atria and mild colpocephaly. Periventricular white matter hyperintensity on T2 and FLAIR imaging, with diminution of white matter volume, consistent with white matter changes of prematurity or PVL. Thinning of the posterior body and splenium of the corpus callosum in keeping with diminution of transcolossal axonal material.
**WIPI2**	**P044**[1]	De novo	0	Chr7:5266876A>G[2]	NM_001033520.1: c.737A>G: p.Y246C	**25**	**5.93**	63.64	B	P	Male, spastic diplegia, born 29 weeks, IVH day 1, respiratory distress, squint (estropia) and visual impairment (Mother also), mother thalassemia minor, developing some foot deformities as he is growing. MRI: mild prominence of ventricles, in particular the posterior atria and mild colpocephaly. Periventricular white matter hyperintensity on T2 and FLAIR imaging, with diminution of white matter volume, consistent with white matter changes of prematurity or PVL. Thinning of the posterior body and splenium of the corpus callosum in keeping with diminution of transcolossal axonal material.
**ZMYM3**	**11451**	Not maternal	0	ChrX:70470498C>T	NM_001171162.1: c.857G>A: p.R286H	**34**	4.9	**8.38**	B	P	Female, born 29 weeks, twin, both with CP.

[1], Case reported in [1], [2], Variant reported in [1]. Abbreviations: A: Automatically annotated Disease causing, ADHD: Attention Deficit Hyperactivity Disorder, ASD: Autism Spectrum Disorder, BW: Birth weight, CADD Phred; Combined Annotation Dependent Depletion scaled score, CT: Computed Tomography scan, D: Damaging(PolyPhen2)/ Disease causing (MutationTaster), DD: Developmental Delay, Ep: Epilepsy, GERP++; Genomic

Evolutionary Rate Profiling, gnomAD; genome aggregation database frequency, ID: Intellectual Disability, IUGR: Intrauterine Growth Restriction, IVH: Intraventricular haemorrhage, MCA: Middle Cerebral Artery, MRI: Magnetic Resonance Imaging, MTR centile; Missense Tolerance Ratio percentile, Mut Taster; Mutation Taster prediction, NDD: Neurodevelopmental Disorder, OCD: Obsessive Compulsive Disorder, PolyPhen2 HVAR; Polymorphism Phenotyping v2 Human Variation based prediction, PVL: Periventricular Leukomalacia, SGA: Small for gestational age, TTTS: Twin to Twin Transfusion Syndrome, US: Ultrasound.

Supplementary Table 4 Genetic variants detected by HaloPlex in rescreened cases

Sample	Gene	Inheritance	gnomAD	Variant	Predicted effect	CADD Phred	GERP++	MTR %ile	PolyPhen2 HVAR	Mut. Taster	Clinical notes
**P003**	**DYNC2H1**	Paternal	0	Chr11:103058091T>C	NM_001080463.1: c.6916T>C: p.S2306P	21.2	**5.12**	53.21	P	**D**	Female, spastic diplegia with mild dystonia especially in upper limbs, twin, born 32 weeks, cortical visual impairment, DD, Ep, convergent squint, some cognitive impairment, MRI: IVH, Irregular enlargement of ventricles particularly posteriorly with reduction in white matter volume and mild delay in myelination.
**P007**	**RNF214**	De novo	0	Chr11:117152637C>T	NM_001077239.1: c.1363C>T: p.P455S	23.4	4.76	97.16	**D**	**D**	Female, mild ataxic / spastic diplegia, born 32 weeks, BW: <1 percentile IUGR, borderline IQ, twin, occipital horns are mildly plump (colpocephaly), behavioural outbursts and temper tantrums.
**P008**	**CTNND2**	Paternal	0	Chr5:11111031T>C	NM_001288716.1: c.1391A>G: p.K464R	18.23	**5.72**	**15.52**	B	**D**	Male, spastic triplegia, born 35 weeks, right orchidopexy, constipation.
	**HSPG2**	Maternal	0	Chr1:22182033G>T	NM_005529.7: c.837C>A: p.A1946D	**29.3**	**5.12**	**7.39**	**D**	**D**	
**P009**	**MAST1**	De novo	0	Chr19:12975755C>T[1]	NM_014975.3: c.1499C>T: p.P500L	**32**	4.39	**3.63**	**D**	**D**	Male, spastic diplegia, born 33 weeks, resolved atrial septal defect, alternating squint and longsighted, white matter damage, mild learning problem, behavioural problems, sensitive hearing, drooling, dis/subluxation hips, cardiac incomplete right bundle branch block, mild pulmonary stenosis, asymmetrical gait pattern and short stride length, MRI: mild generalised volume loss, PVL, cystic encephalomalacia.
**P010**	**MCPH1**	Paternal	3.61E-05	Chr8:6479017G>A	NM_024596.5: c.2257G>A: p.G753R	**27.9**	4.45	**4.31**	**D**	**D**	Male, spastic diplegia, born 40 weeks, severe ID, ASD, global DD, visual impairment suspected of

											cerebral origin with roving eyes and visual inattention slightly down-slanting palperbral fissures, self-injuring behaviour, sleep disturbances, non-verbal apart from echolalia with bizarre vocalisations, Mother Ep. MRI: Congenital hydrocephalus associated with aqueduct stenosis. Significant loss of white matter presumable secondary to hydrocephalus. Cortical visual impairment. X-ray: abnormal appearance of skull with a copper-beaten appearance, narrowing of the foramen magnum and impression of superior migration of the posterior portion of the occipital bone.
**P015**	**PAK3**	X-linked	0	ChrX:110459718C>T[1]	NM_001128167: c.C1477T: p.R493C	**34**	**5.54**	97.68	**D**	**D**	Male, dystonic spastic hemiplegia, born 29 weeks, BW: <1 percentile IUGR Ep, cognitive ability upper limit of low-average range, developed hydrocephalus, MRI: grade 4 IVH, right sided and grade 2 IVH left sided
	**DNAH2**	Unknown	0	Chr17:7643054T>C	NM_020877.3: c.1174T>C: p.C392R	**26.8**	**5.09**	88.21	**D**	**D**	Variant not in WES
**P016**	**MCCC1**	Paternal	7.22E-06	Chr3:182755152T>C	NM_001293273.1: c.1097A>G: p.N366S	24.4	4.75	**12.26**	P	**D**	Male, spastic quad, born 40 weeks, BW: 2nd percentile IUGR, Ep onset at 6 mths, tonic-clonic seizures 2-3 times per day; swallowing difficulties; severe ID; communicates non-verbally, swallowing difficulties, delivery complicated by significant foetal distress resulting in precipitous delivery using forceps with poor apgars.
**P025**	**MAOB**	X-linked	0	ChrX:43634520C>T[1]	Splicing NM_000898.5:	23.8	**6.17**	.	.	**D**	Male, spastic hemi, born 40 weeks, severe sleep disturbance, anxiety

					c.1138-1G>A						issues, behaviour problems, Irregularity and dilation of the left lateral ventricle associated with periventricular white matter volume loss and thinning of the corpus callosum.
**P026**	**DNAH3**	Maternal	3.97E-05	Chr16:21060927C>G	NM_017539.2: c.4424G>C: p.G1475A	27.5	5.88		D	D	Female, spastic quad with greater involvement of lower limbs, born 39 weeks, BW: 5th percentile IUGR, small stature, convergent strabismus, ridging metopic suture, mild trigonocephaly, severe behaviour problems, speech dyspraxia, photophobia
	**KDM5C**	De novo	0	ChrX:53240002G>A[1]	NM_001146702.1: c.1238C>T: p.P413L	**28.6**	**5.42**	**3.28**	**D**	**D**	
	**TENM1**	Paternal	0	ChrX:123518642C>G	NM_014253.2: c.6118G>C: p.D2040H	23.6	**5.44**	**14.87**	**D**	**D**	
**P027**	**MCPH1**	Maternal	2.03E-05	Chr8:6357439C>T	NM_024596.5: c.2203C>T: p.P735S	**34**	**5.78**	**14.59**	**D**	**D**	Male, spastic quad, born 29 weeks, mother had pre-eclampsia. DD, mild ID, BW: <1 percentile IUGR, respiratory distress syndrome, impaired hearing; umbilical and inguinal hernias; short stature (<3rd percentile) from birth onwards and underweight (3rd-10th percentile). At birth head circumference was 3.9^{th} percentile for gestational age, and 5th percentile at 7 years. US: Initial ultrasounds at 2 months were normal, but subsequent cranial ultrasounds showed bilateral parietal-occipital PVL (L>R).
**P030**	**MYO1F**	Compound het/ Maternal	8.14E-06	Chr19:8601410C>T	NM_012335.3: c.1871G>A: p.R624H	35	4.83		D	D	Male, spastic quad, born 37 weeks, BW: <1 percentile IUGR, DD, Ep with myoclonic seizures, cortical visual impairment, cataracts, scoliosis thoraco-lumbar and kyphosis mid thoracic; bilateral

											undescended testes, non-verbal, communicates with communicator. Experienced episodes of severe bradycardia before onset of labour. Placenta showed multiple infarcts and there was a velamentous insertion of the cord. Had some limb contractures at delivery associated with oligohydramnios. At birth required sedation and ventilation and had evidence of cerebral oedema in neonatal period. MRI: Scaphocephaly of the skull with an elongated narrowed brain. Moderate ventricular dilation more marked on the left with enlarged subarachnoid space. Immaturity in myelination, particularly in temporal lobes and parietal regions. High signal periventricular white matter changes suggesting PVL. Probably subependymal grey matter heterotopias. Hypoplastic thin corpus callosum.
		Compound het/ Paternal	2.54E-03	Chr19:8587401C>T	NM_012335.3: c.3080G>A: p.R1027Q	25.2	5.37		D	D	
	**CTDSPL**	De novo	0	Chr3:38009345C>T[1]	NM_005808.2: c.365C>T: p.P122L	**34**	**5.25**	**5.26**	**D**	**D**	
**P033**	**AGAP1**	De novo	0	Chr2: 236708167G>A[1]	Splicing NM_014914.4: c.957+1G>A	**27.3**	**5.08.**	.	.	**D**	Male, spastic quad with generalised hypotonia, dystonic posturing and myoclonic jerks, born 37 weeks severe ID, Ep, reduced oral control and tongue movement, MRI: PVL in frontal and parietal lobes (L>R), absent L caudate nucleus, putamen and globus pallidus, short stature at 4 years, died at 7 years.
	**COL4A1**	Paternal	0	Chr13:110831315C>T	NM_001845.6:	24.6	4.7	84.91	**D**	**D**	

					c.2413G>A: p.G805R						
**P035**	**COL4A1**	Not maternal	8.12E-06	Chr13:110895030C>T	NM_001303110.1: c.136G>A: p.G46R	**25.7**	4.26	64.81	**D**	**D**	Female, spastic hemi, born 27 weeks, BW: <1 percentile IUGR, mild ID, impaired hearing, divergent squint, Ep, thyroid agenesis, deterioration of motor function and bladder control; at 5 years, sleep apnoea, recurrent ear infections, PEG feeding, short stature and underweight at 16 years, US: L PVL, CT: mild ex-vacuo ventricular dilation.
**P042**	**SLC6A3**	Not maternal	0	Chr5:1411428G>A	NM_001044.5: c.1199C>T: p.T400M	18.55	4.69	**11.78**	**D**	**D**	Female, spastic hemi, born 30 weeks, BW: <1 percentile IUGR, DD, mild ID, Ep (focal seizures), respiratory distress syndrome, behaviour problems.
**P043**	**CNDP2**	Maternal	4.10E-06	Chr18:72173171G>A	NM_018235.3: c.292G>A: p.G98R	**26.6**	4.52	**18.07**	**D**	**D**	Female, spastic hemi, born 41 weeks, mild ID, elbow flexion deformity. CT: Abnormality in left hemisphere, likely consistent with an old MCA territory infarct. Left ventricle dilated, low white matter and a calcium focus. Small left thalamus, smaller left brain in general.
**P044**	**WIPI2**	De novo	0	Chr7:5266876A>G[1]	NM_001033520.1: c.737A>G: p.Y246C	**25**	**5.93**	63.64	B	P	Female, triplegia, motor vehicle accident at 29 weeks resulting in head injury in utero, born 30 weeks. US: Non-shunted hydrocephalus and right temporoparietal porencephalic cyst-secondary to infarct. MRI: right PVL, bilateral ventriculomegaly, thin corpus callosum, old bilateral IVH. No evidence of progressive hydocephalus.
**P049**	**DNAH3**	Maternal	0	Chr16:20970654T>C	NM_017539.2: c.10673A>G: p.Q3558R	26.8	5.41		P	D	Male, spastic hemi, born 35 weeks, BW: 5th percentile IUGR, MRI: Slight ventricle asymmetry, two

											small subependymal nodules on the lateral wall of the right lateral ventricle pointing to minor variant neuronal migration disorder (subependymal heterotopia), some asymmetry of silvian fissures but no definite polymicrogyria or pachygyria.
	**HSPG2**	Compound het/ Paternal	0	Chr1:22157706A>T[1]	Splicing NM_005529.7: c.11562+2T>A	24.7	**5.46**	.	.	**D**	
		Compound het/ Maternal	4.07E-06	Chr1:22170696G>T[1]	NM_005529.7: c.8561C>A: p.A2854D	24.3	4.43	55.91	P	**D**	
**P051**	**IGSF1**	X-linked	6.15E-05	ChrX:130413296T>A[1]	NM_001170962.1: c.1639A>T: p.T547S	22.5	**5.04**	73.72	**D**	N	Male, mild spastic hemi, born 39 weeks, some articulation problems. US: Mild dilation of the anterior horn of the right lateral ventricle, consistent with a porencephalic cyst; area of the cystic encephalomalacia which has communicated with the anterior horn of the right lateral ventricle.
**P052**	**PROC**	Compound het/ Paternal	2.53E-05	Chr2:128178957C>T[1]	NM_000312.3: c.169C>T: p.R57W	**32**	3.33	.	**D**	**D**	Female, spastic/dyskinetic quad, born 38 weeks, ID, Ep, probable Gardner-Diamond syndrome, mild dysphagia, gradual but progressive decline neuro-developmentally, 4th child, 1st and 3rd female siblings deceased from obscure degenerative leukoencephalopathy, MRI: PVL, porencephaly, partial agenesis of corpus callosum (absent genu, rostrum, body with splenium present).
		Compound het/ Maternal	2.53E-05	Chr2:128185950C>T[1]	NM_000312.3: c.814C>T: p.R272C	.	.	**18.40**	**D**	**A**	As above
**P058**	**INHBB**	De novo	8.21E-06	Chr2:121106698C>T[1]	NM_002193.4: c.472C>T: p.R158C	**26.2**	**5.09**	**30.77**	B	**D**	Male, spastic quad, born 38 weeks, mild ID, Epilepsy, DD, congenital CMV infection, generalized

											polymicrogyria, several febrile seizures
**P059**	**MIIP**	Maternal	0	Chr1:12082422delG	F/shift del NM_021933.3: c.385delG: p.Arg130Glyfs*28	.	.	.	.	.	Male, spastic diplegia, born 25 weeks, ASD, DD, vision impaired, mild ID, anxiety, severe language disorder characterised by difficulties understanding and using language, echolalia, short stature and low weight at 1 year, expression of MIIP downregulated [7], US: bilateral germinal matrix haemorrhages, probably extending into the ventricle on both side, with cystic change. CT: ventricular dilatation and irregularity of the posterior aspects of the lateral ventricles in keeping with end stage PVL.
**P067**	**UBXN7**	De novo	0	Chr3: 196120470C>T[1]	NM_015562.2: c.310G>A: p.A104T	20.1	4.47	62.04	P	**D**	Female, spastic diplegia, born 27 weeks, US: bilateral grade II IVH, then PVL. MRI: PVL. Bilateral increase in white matter signal in the periventricular regions most marked in the occipital areas and centrum semiovale regions consistent with mild changes of periventricular leukomalacia with minor thinning of the corpus callosum posteriorly.
	**HSPG2**	Maternal	4.07E-06	Chr1:22181376G>A	NM_005529.7: c.6098C>T: p.T2033I	**25.6**	4.58	**20.84**	P	**D**	
	**TH**	Paternal	3.64E-05	Chr11:2192973C>A	NM_000360.3: c.44G>T: p.R15L	**34**	3.87	82.73	**D**	**D**	
**P068**	**DNAH2**	Paternal	2.03E-05	Chr17:7643207delG	F/shift del NM_020877.2: c.1327delG: p.Gly444Valfs*26	.	.	.	.	.	Variant not detected by WES. Male, spastic hemi, born 39 weeks, induced at term because of unusual abdominal pain. Hypoglycaemia, mild asthma, mild scaphocephaly consistent with intrauterine moulding, possible left

											septal hypertrophy and RVH with borderline ECG, likely pre-natal CVA. US: ex-vacuo dilation of right ventricle. MRI: right PVL and cortical involvement in region of right pre-central gyrus. CT: ex-vacuo dilation of right ventricle and long-standing changes consistent with hypoxic ischaemic injury.
**P082**	**SLC6A3**	Paternal	0	Chr5:1443060G>A	NM_001044.5: c.253C>T: p.R85W	**32**	1.46	**8.54**	**D**	**D**	Female, spastic quad, born 38 weeks, BW: 2nd percentile IUGR, hearing & vision loss, microcephaly, swallowing issues (at risk of aspiration), CMV diagnosed by blood test at 1 year old
**P084**	**NR1I2**	De novo	8.19E-06	Chr3:119534605G>A	NM_003889.3: c.1087G>A: p.D363N	**25.8**	**5.04**	**42.76**	**D**	**D**	Variant not detected by WES, Female, dyskinetic mixed diplegia, born 40 weeks, right side more affected than left. Dyskinetic mixed: bilateral, low apgars at birth. Multi organ failure, seizures and need for ventilation at birth, Ep. US: mild cerebral oedema, then increased echogenicity of white matter. MRI: changes of gliosis in thalami bilaterally, posterior limb of the left external capsule and high subcortical white matter of the parietal lobes/corticospinal tract. Appearances are consistent with gliosis from previous hypoxic ischaemic encephalopathy.
**P099**	**CTNND2**	Unknown	3.97E-05	Chr5:11022927C>T	NM_001288716.1: c.1942G>A: p.G648S	**26.9**	**5.64**	55.00	**D**	**D**	Variant not detected by WES. Male, spastic diplegia, born 31 weeks, twin, convergent squint paternal cousin CP. Hyaline membrane disease, ventilated for first 3 days, therafter 5 days CPAP and treated with caffeine for apnoeas and bradycardias. Jaundice with a max bilirubin of 188 led to phototherapy. Strabismus surgery,

											esotropia, mild optic nerve dysfunction and inferior visual field defect; equinus gait pattern. US: day 2 normal, day 8; visible flare in the frontal and occipital regions on the left and evidence of a small resolving haemorrhage in the right lateral ventricle. PVL with cystic encephalomalacia in the areas of the left frontal region and left occipital region have progressed.
**P101**	**NGFR**	De novo	3.28E-05	Chr17: 47589394[1]	NM_002507.4: c.962C>T: p.T321M	**25.3**	**5.18**	70.17	B	**D**	Male, spastic quad, born 40 weeks, parents first cousins, dysphagia, dysarthria, severe dystonia, ID, no speech but communicates well with computer. MRI: Cortical dysplasia. Fronto parietal temporal dysplasia, suggestive of bilateral frontoparietal polymicrogyria.
**P103**	**DNAH2**	Maternal	0	Chr17:7644290C>T	NM_020877.3: c.1669C>T: p.R557C	**31**	**5.23**	**27.55**	**D**	**D**	Male, spastic hemi, born 35 weeks, Ep (on EEG at age 4 yr: prominent epileptic foci in both posterior regions with a right sided emphasis), ID, ASD, significant behaviour problems, attention seeking behaviour, interruptive and manipulative at a young age, speech problems, right inguinal hernia; one spot of depigmentation of 2.5 cm on left side of neck. VSD spontaneously closed
**P104**	**DDHD2**	Paternal	0	Chr8:38092037T>G	NM_001164232.1: c.346T>G: p.F116V	**28.5**	**5.79**	**29.35**	**D**	**D**	Male, spastic hemiplegia, born 40 weeks, BW: <1 percentile IUGR, hydrocephalus, congenital hyper-insulinaemia, severe hypoglycaemia and thrombocytopenia, basal ganglia haemorrhages, accommodative estropia. US: bilateral grade III IVH, ventriculomegaly. Later US suggest progression of IVH involving both lateral ventricles, with associated

											dilatation of both lateral ventricles, when compared to first US. MRI (at day 12 following severe hypoglycaemia and sepsis, severe thrombocytopenia): extensive bilateral basal ganglia, intraventricular and basal cistern haemorrhage with evolving moderate hydrocephalus, subacute spinal cord infarct.
**P105**	**KDM7A**	De novo	0	Chr7:139796549G>C[1]	NM_030647.2: c.C2180C>G: p.S727W	**34**	**5.85**	**36.31**	**D**	**D**	Female, spastic/dystonic quad, born 38 weeks, BW: 5th percentile IUGR, more signs on right than left, more dystonia than spasticity, born at 38 weeks after IUGR, neonatal hypoglycaemia, and subsequent neonatal seizures. ID, Ep with focal epileptiform abnormality, focal seizures starting at day 7, febrile clonic tonic seizure at 2 years. US: echogenicity of periventricular white matter and left thalamus. MRI: extensive PVL, Haemorrhagic foci as well as foci of restricted diffusion, majority of the pathology is likely to have occurred prior to delivery.
**P106**	**COL4A1**	Paternal	0	Chr13:110813663T>C	NM_001845.6: c.4516A>G: p.N1506D	20.3	4.56	**22.10**	P	**D**	Male, spastic hemi, born 39 weeks, bilateral femoral anteversion
**P109**	**CNDP2**	De novo	0	Chr18:72173219insACC	NM_018235.3: c.504_506dupAGC: p.E114_P115insQ	.	.	.	.	.	Male, spastic hemi, born 35 weeks, BW: 2nd percentile IUGR, history of in utero CVA, Ep, obesity at 8 yrs; DD, significant learning difficulties, left homonymous hemianopia. CT: large right MCA territory infarct, in utero CVA. Extensive hypodensity involving vast majority of under developed right hemisphere and associated ex-vacuo dilatation of the right lateral ventricle. Right side of the calvarium is

											underdeveloped compared to the left. Extensive change to the right hemisphere.
	**MYO15A**	Maternal	2.51E-05	Chr17:18070991G>A	NM_016239.4: c.10036G>A: p.A3346T	**26**	**5.61**	53.26	**D**	**D**	
**P111**	**EIF4E2**	De novo	0	Chr2:233422672C>G[1]	NM_001276336.1: c.214C>G: p.P72A	16.93	**5.27**	**44.44**	B	**D**	Male, spastic diplegia, born 41 weeks, hypoglycaemia, seizures day 3, thrombocytopenia, high pitch cry as neonate, mild squint and hypermetropia. US day 3: increased periventricular echogenicity, with left frontal and right parietal oedema. MRI: 1 week, restricted diffusion with parasagittal and basal ganglia involvement and PVL. MRI 2 months, shows residual changes of PVL only.
**P113**	**MYO15A**	Paternal	0	Chr17:18051498T>A	NM_016239.4: c.6665T>A: p.M2222K	24.1	4.1	**2.36**	**D**	**D**	Male, spastic/dyskinetic quad, born 40 weeks, hypotonia in all 4 limbs, mother collapsed with possible amniotic fluid embolus, child delivered somewhat hypoxic. Kyphosis, sialorrhea with drooling, nose bleeds, undescended testes, ID, DD, gastrostromy fed, short stature (<3rd percentile at 6 years). US: normal. MRI: bilateral symmetric signal changes in the lentiform nuclei and perirolandic cortex.
**P115**	**CNKSR2**	X-linked	2.02E-05	ChrX:21508607A>G[1]	NM_001168647.2: c.592A>G: p.I198V	15.67	**5.83**	50.43	**D**	**D**	Male, spastic diplegia, born 41 weeks, moderate ID; Ep (mostly complex partial seizures, 3-5 per month); ASD; expressive speech delay; severe visual impariment, optic nerve atrophy; adducted thumbs; behavioural problems but generally sociable and happy. US: congenital tetraventricular hydrocephalus, MRIs:

										hydrocephalus communicating with persistent Blake's pouch cyst, cerebellar folia disorganisation, white matter volume loss, partial agenesis of the corpus callosum, abnormal positioning of thalamus and lentiform nucleus, incomplete hippocampal inversion bilaterally, hypoplasia of the optic nerve, hypoplasia of the cerebellar vermis and asymmetry of the pons.
	**MYO15A**	De novo	0	Chr17:18051510[1]	NM_016239.4: c.6677T>C: p.V2226A	23.6	4.1	**5.41**	**D**	**D**	
**P116**	**DNAH2**	Maternal	0	Chr17:7734801C>A	NM_020877.3 c.12553C>A pS3698Y	21.6	**5.3**	**47.29**	P	**D**	Female, spastic quad, born 26 weeks, DD, ID, short stature (3rd percentile), chronic lung disease requiring home oxygen. US: IVH grade II left, grade II/III right, then ventriculomegaly, porencephaly
**P117**	**NAA35**	De novo	0	Chr9:88631481G>A[1]	NM_024635.3: c.1596G>T: p.W532C	**33**	**5.4**	**20.14**	**D**	**D**	Male, spastic hemi, born 39 weeks, borderline ID, refractory focal seizures and a left homonymous hemianopia, unilateral cerebral thrombotic infarction. US: increased echogenicity right thalamus, basal ganglia and deep white matter. MRI: extensive right MCA territory perinatal infarct with extensive encephalomalacia involving the central regions, frontal, parietal and occipital lobes. Right brain size smaller. Cystic changes. Right lateral ventricle dilated. Temporal lobe is of a reduced volume.
	**PCBP3**	De novo	4.08E-06	Chr21:47355203T>C[1]	NM_001130141.1: c.815T>C: p.L272P	**29.3**	3.72	70.81	**D**	**D**	
	**USP26**	X-linked	0	ChrX:132161343G>C[1]	NM_031907.1: c.906C>G: p.N302K	23.9	0.143	**1.97**	**D**	N	

**P119**	**AP4E1**	Paternal	0	Chr15: 51242290C>T	NM_001252127.1: c.1247C>T: p.A416V	**26.3**	4.86	**2.63**	P	**D**	Female, spastic hemi, born 39 weeks, BW: 9th percentile IUGR, Ep. US: appearances consistent with mature left MCA territory infarct. MRI: dilatation of the left lateral ventricle, left hemisphere is reduced in volume when compared with the normal right side. There is an area of encephalomalacia in the left posterior frontal and anterior parietal regions most marked in the vicinity of the left sylvian fissure.
**P403**	**NGFR**	Paternal	6.48E-05	Chr17:47590297G>A	NM_002507.4: c.1210G>A: p.A404T	**34**	3.49	76.13	**D**	**D**	Male, spastic diplegia, born at 35 weeks, BW: 4th percentile IUGR, strabismus.
**P405**	**ENOX2**	X-linked	0	ChrX:129822993C>G[1]	Splicing NM_006375.3: c.98-1G>C	19.47	**5.1**	.	.	.	Male, spastic diplegia, born 30 weeks
**P414**	**MAN2A1**	De novo	0	Chr5:109159498T>A[1]	NM_002372.3: c.2526T>A: p.F842L	17.4	4.37	69.52	B	**D**	Male, spastic hemi, born 40 weeks, Von Willebrand disease, Ep.
**P427**	**DNAH2**	Compound het/ Maternal	4.06E-06	Chr17:7660536G>A	NM_020877.3: c.2032G>A: p.V678I	20.6	3.98	57.43	**D**	**D**	Male, spastic hemi, born 38 weeks.
		Compound het/ Paternal	0	Chr17:7696064T>G	NM_020877.3: c.7235T>G: p.I2412S	21.9	3.27	**20.53**	B	N	
	**MGA**	Maternal	6.79E-04	Chr15:42035028A>G	NM_001164273.1: c.4870A>G: p.K1624E	**25.2**	4.96	**31.20**	P	**D**	
**P428**	**UBQLN3**	De novo	0	Chr11:5530219del CAGCTGG[1]	F/shift del NM_017481.4: c.564_570del: p.Q188Ffs*10	.	.	.	.	.	Male, spastic diplegia, born 29 weeks, triplet, mild ID.
**P436**	**SREK1**	De novo	4.09E-06	Chr5:65449298C>T[1]	NM_001077199.3: c.169C>T: p.P57S	18.21	**5.61**	**7.2**	P	**D**	Female, spastic diplegia, BW: 4th percentile IUGR, twin, born 29 weeks
**P443**	**ADCY3**	Paternal	0	Chr2: 25064186G>A[1]	NM_004036.4: c.1138C>T: p.R380W	**33**	4.6	**0.786**	**D**	**D**	Female, spastic hemi, born 39 weeks, maternal uncle severe physical and mental disability.
	**NR1I2**	De novo	0	Chr3:119534572G>T[1]	Splicing	24.8	**5.04**	.	.	**D**	

					NM_033013.2: c.944-1G>T						
	**RFX2**	De novo	0	Chr19:6040241T>C[1]	NM_000635.4: c.272A>G: p.Y91C	23.9	**5.36**	80.53	**D**	**D**	
**P454**	**MIIP**	De novo	0	Chr1:12082151G>A[1]	Splicing NM_021933.3: c.115-1G>A, d/stream cryptic splice site used, delTCAGG in cDNA, f/shift del	15.47	3.47	.	.	.	Male, spastic diplegia, born 41 weeks, paternal cousin with spastic diplegia.
**P455**	**TUBA8**	Paternal	0	Chr22:18604342G>C	NM_018943.3: c.100G>C: p.G34R	**31**	**5**	**29.33**	**D**	**D**	Male, spastic diplegia, born 26 weeks.
**P465**	**PTGFRN**	De novo	2.85E-05	Chr1:117509692A>G[1]	NM_020440: c.A1799G: p.N600S	23.1	**5.56**	71.02	P	**D**	Female, spastic diplegia, born 34 weeks, vanishing twin syndrome, mother placental abruption due to previously unknown clotting disorder.

[1,] Variant reported in [1]. Abbreviations: A: Automatically annotated Disease causing, ADHD: Attention Deficit Hyperactivity Disorder, ASD: Autism Spectrum Disorder, BW: Birth weight, CADD Phred; Combined Annotation Dependent Depletion scaled score, CT: Computed Tomography scan, D: Damaging(PolyPhen2)/ Disease causing (MutationTaster), DD: Developmental Delay, Ep: Epilepsy, GERP++; Genomic Evolutionary Rate Profiling, gnomAD; genome aggregation database frequency, ID: Intellectual Disability, IUGR: Intrauterine Growth Restriction, IVH: Intraventricular haemorrhage, MCA: Middle Cerebral Artery, MRI: Magnetic Resonance Imaging, MTR centile; Missense Tolerance Ratio percentile, Mut Taster; Mutation Taster prediction, NDD: Neurodevelopmental Disorder, OCD: Obsessive Compulsive Disorder, PolyPhen2 HVAR; Polymorphism Phenotyping v2 Human Variation based prediction, PVL: Periventricular Leukomalacia, SGA: Small for gestational age, TTTS: Twin to Twin Transfusion Syndrome, US: Ultrasound.

Supplementary Table 5 Validated variants detected by HaloPlex in naïve samples

Sample	Gene	Inheritance	gnomAD	Variant	Predicted effect	CADD Phred	GERP++	MTR %ile	PolyPhen2 HVAR	Mut. Taster	Clinical notes
**P087**	**TH**	Paternal, affected sibling does not have variant	8.33E-06	Chr11:2189774A>T	NM_000360.3: c.434A>T: p.L145H	24.2	3.67		D	D	Male, ataxic CP, born 38 weeks, father and sister also. Mild heart murmur, vitiligo.
**P120**	**HSPG2**	Maternal	0	Chr1:22206710A>C	NM_005529.7: c.2233T>G: p.C745G	**25.1**	**5.38**	88.73	**D**	**D**	Male, spastic quad, DD, born 38 weeks, BW: 9th percentile IUGR, Ep, Visual impairment, physical impairment, hypothyroidism, inability to control body temperature
**P172**	**ENPP4**	Unknown	4.13E-06	Chr6:46111046G>C	NM_014936.5: c.1031G>C: p.S344T	19.71	**5.01**	**44.71**	P	**D**	Female, spastic diplegia, born 35 weeks, acute disseminated encephalomyelitis; CNS demyelination in infancy (leukodystrophy/ leukoencephalyopathy), obstructive sleep apnoea - adenoids removed.
	**GANC**	Unknown	0	Chr15:42618608G>T	NM_198141.2: c.1406G>T: p.G469V	**27.6**	**5.38**	**39.89**	**D**	**D**	
**P174**	**DNAH3**	Not maternal	6.50E-05	Chr16:20976075G>A	NM_017539.2: c.9131C>T: p.T3044M	24.7	**6.04**	55.11	P	**D**	Male, spastic quad, born 40 weeks, partial dysgenesis of corpus callosum, global DD, Ep (first seizure at 12 months), optic atrophy (cortically blind); type 1 diabetes, scoliosis in the lumbar region and an oblique pelvis, incontinent of faeces and urine, cortical atrophy has been progressive.
	**KIF1A**	Not Maternal	0	Chr2:241727535G>A	NM_004321.7: c.296C>T: p.T99M	**31**	4.79	**5.96**	**D**	**D**	
**P176**	**GAD1**	Not maternal	4.06E-06	Chr2:171702037A>G	NM_000817.3: c.773A>G: p.Y258C	**26.3**	**5.67**	**33.11**	**D**	**D**	Male, spastic hemi, born 24 weeks, porencephalic cyst, Ep, global DD, mild ASD, limited speech, Left hand with increased tone; left ankle/foot orthotic; independent walker. MRI: IVH grade IV.
**P177**	**KIF1C**	Paternal	5.05E-05	Chr17:4927405C>T	NM_006612.6: c.3271C>T: p.R1091C	**32**	**5.04**	96.60	**D**	**D**	Male, spastic/dyskinetic hemi, born 41 weeks, right affected leg; dragging it; 1cm difference in length, has difficulties

											with balance on the right hand side. MRI: evidence of gliosis/encephalomalacia in the left perisylvian region involving the left lentiform nucleus; caudate; and corona radiata. Ex-vacuo dilation of left lateral ventricle and diminuition of periventricular white matter, consistent with old area of infarct in the left MCA/lateral lenticulostriate territory.gliosis/encephalomalacia in left perisylvian region involving the left lentiform nucleus, caudate, and corona radiata
**P188**	**DYNC2H1**	Unknown	2.97E-04	Chr11:103027166G>A	NM_001080463.1: c.3794G>A: p.R1265H	23.1	**5.27**	**29.25**	B	**D**	Female, spastic quad with some evidence of dyskinesia, born 37 weeks, BW: 1st percentile IUGR, induced birth due to oligohydramnios and IUGR, diagnosed neonatal encephalopathy, hypoglycaemic day 2 and lactic acidosis on initial cord bloods; apnoeic episodes/seizures day 9; stiff abnormal flexion/extension and fine tremor; difficulties maintaining midline orientation. No evidence of encephalopathy until onset of apnoea and seizures. Moderate hearing deficits, squint and cortical visual impairment; cranial nerve palsies, GORD, feeding difficulties; vomiting and slow weight gain, lactose intolerant, recurrent otitis media and tonsillitis, BPD (maternal Grandmother also). MRI: extensive injury of frontoparietal white matter basal ganglia and thalamic nuclei; symmetric hypoxic brain injury.
	**KANK1**	Unknown	1.22E-05	Chr9:732430G>A	NM_153186.5: c.2584G>A: p.E862K	24.3	**5.64**	64.27	**D**	**D**	
**P192**	**VRK1**	Unknown	8.14E-06	Chr14:97299816G>T	NM_003384.3: c.8G>T: p.R3L	23.5	**5.03**	79.35	B	**D**	Male, spastic diplegia, born 29 weeks, IVH day 1, respiratory distress, squint (estropia) and visual impairment

											(Mother also), mother thalassemia minor, developing some foot deformities as he is growing. MRI: mild prominence of ventricles, in particular the posterior atria and mild colpocephaly. Periventricular white matter hyperintensity on T2 and FLAIR imaging, with diminution of white matter volume, consistent with white matter changes of prematurity or PVL. Thinning of the posterior body and splenium of the corpus callosum in keeping with diminution of transcolossal axonal material.
**P200**	**HSPG2**	Unknown	4.71E-05	Chr1:22172639G>A	NM_005529.7: c.8426C>T: p.T2809I	23.7	4.29	81.15	P	N	Female, spastic hemi, born 36 weeks, mother auto-immune thrombocytopenia, IVH with hydrocephalus diagnosed at 32 weeks, high frequency hearing loss, expressive speech and language delay; gross/fine motor deficit; asthma; lazy eye; bilateral nasolacrimal duct obstruction. US at birth: dilatation of both lateral ventricles, the left more than the right. Third and fourth ventricles are of normal calibre. No IVH identified. Frontal horn of left lateral ventricle dilated and small cystic structure seen superior to the frontal horn with a membrane adjacent to it in keeping with a periventricular cyst. Lateral to the frontal horn of the left lateral ventricles are some smaller cystic areas, these appearances are suggestive of some PVL.
	**KANK1**	Unknown	0	Chr9:731250G>C	NM_153186.5: c.2515G>C: p.V839L	22.9	4.62	58.66	B	P	
**P202**	**DMD**	X-linked	0	ChrX:31144772G>A	NM_004015.2: c.1790A>G: p.K597R	22.9	**6.02**	97.19	**D**	**D**	Male, spastic diplegia, born 39 weeks, hydrocephalus secondary to aqueduct stenosis, benign mature teratoma excised, normal cognition and behaviour, considerable lower limb

										spasticity that impacts on gross motor function, particularly gait quality, reasonable to good selective motor control and strength. MRI: High signal on flair in the region trigones is suggestive of PVL. Absent septum pellucidum and thinning / irregularity of corpus callosum. Right parietal approach shunt in situ.
**P203**	**MTMR1**	X-linked	0	ChrX:149898672C>G	NM_001306145.1: c.623C>G: p.S208C	24	**5.35**	**13.74**	P	**D**	Male, spastic hemi, born 38 weeks, congenital microcephaly, plagiocephaly (moderate), cortical dysplasia; failure to thrive; microcrania, primordial smallness, sensorineural hearing loss; developmental delay; prominent expressive language delay; aspiration; lung disease (resolved); swallowing difficulties. MRI at 3 years: Extensive cortical dysplasia, with white matter changes likely to be associated with underlying dysplasia rather than representing a dysmyelinating process. MRI at 6 years: Appearances favour a low-grade glioma involving the left cerebellar hemisphere in a parasagittal location in the peri-dentate white matter on a background of bifrontoparietal polymicrogyria. MRI at 9 years: Cerebellar lesion excised at 8 years. Cystic cavity within the left cerebellar hemisphere has decreased in size; alteration in enhancement pattern of this resected section is noted, was previously rim enhancing, however, now demonstrates nodular enhancement. No haemorrhage or mass identified.
**P204**	**COL4A1**	Not maternal	0	Chr13:110830543C>T	NM_001845: c.G2494A: p.G832R	**27.8**	**5.52**	**43.59**	**D**	**D**	Male, choreoathetoid quad, born 40 weeks, MRI: haemorrhagic gliosis, known COL4A1 mutation from diagnostic WES, cystic porencephaly involving basal ganglia; damage to small blood vessels with increased risk of

											haemorrhage; Epileptic encephalopathy, infantile spasms onset 1 year, evolution into mixed seizure types now; unexplained irritability; no speech or communication, global DD; tracheobronchomalacia; laryngomalacia; gastrostomy; fundoplication; scoliosis; left ventricular hypertrophy and hypertension; enlarged left kidney >10% discrepancy; dental caries; sleep apnoea; hip surgery, past femoral osteostomies; chronic lung disease; failure to thrive; hypoplastic optic nerves, MRI: haemorrhagic gliosis.
	**DNAH2**	Maternal	2.89E-05	Chr17:7671564G>A	NM_020877: c.G3920A: p.R1307Q	**32**	3.58	89.07	**D**	**D**	
	**ENPP4**	Maternal	7.29E-06	Chr6:46111054C>A	NM_014936: c.C1039A: p.P347T	**29.3**	**5.9**	**39.47**	**D**	**D**	
**P206**	**DYNC2H1**	Maternal	3.22E-05	Chr11:103041621G >A	NM_001080463: c.G5158A: p.D1720N	**34**	4.92	**46.46**	**D**	**D**	Female, spastic hemi, born 43 weeks, BW: <1 percentile IUGR, DD, microcephaly, asthma and eczema. Mild spastic left arm with elbow contracture; poor dexterity with the left hand. Mild increased tone in her left leg and wears an orthotic. MRI: Nonspecific white matter lesions bilaterally. CT: Possible generalised cerebral atrophy with prominence of the ventricular system, suggestive of possible PVL. There are also small areas of calcification adjacent to the right lateral ventricle.
**P209**	**DYNC2H1**	Maternal	1.87E-05	Chr11:103114464A >T	NM_001377: c.A9862T: p.T3288S	22.7	**5.66**	77.60	P	**D**	Male, spastic quad, born 38 weeks, diagnosed neonatal encephalopathy, ID, global DD, GORD, hearing impairment, gastrostromy, drooling. EEG: very abnormal with very asymmetric electroencephalogram. MRI: Early neuroimaging showed diffuse cerebral oedema, a severe global brain injury with severe white and grey matter

											changes. Evolution of extensive cystic encephalomalacia changes both within a watershed distribution in the cerebral hemispheres bilaterally, but also involving most of the parenchymal cortex with sparing of a small portion of the left frontal and left medial temporal cortices. Brainstem and cerebellum are normal. Basal ganglia are affected bilaterally, more severely on the right, with some sparing of the left thalamus and left globus pallidus.
**P212**	**HSPG2**	Maternal	0	Chr1:22200903G>A	NM_005529: c.G3652A: p.G1218S	22.7	4.14	63.72	**D**	**D**	Female, spastic hemi, born 25 weeks, chorioamnioitis, PDA, mild thoracic scoliosis. MRI: Focal periventricular leucoencephalomalacia in the posterior right frontal lobe. Adjacent dilatation of the lateral ventricle but no hydrocephalus or other signal changes within the periventricular white matter.
**P215**	**DNAH2**	Maternal	0	Chr17:7680779A>G	NM_020877.3: c.5074A>G: p.K1692E	23.8	**5.51**	73.89	P	**D**	Female, spastic diplegia, born 31 weeks, DD, visual problems, Noonan syndrome, cousin ASD, uncle ADHD, squint; short stature, faints; insomnia; GORD; dental caries related to chronological hypoplasia and enamel development defect; pulmonary valve stenosis; IgA deficiency; hypoglycaemia; early iron deficiency with microcytosis but not anaemia. MRI: evidence of PVL.
	**HSPG2**	Paternal	6.52E-05	Chr1:22188281C>T	NM_005529.7: c.4924G>A: p.E1642K	24.6	**5.14**	**30.31**	**D**	**D**	
	**KIF1C**	Paternal	8.941E-05	Chr17:4924169G>A	NM_006612.6: c.2006G>A: p.R669Q	**34**	**5.68**	75.36	**D**	**D**	
**P216**	**MAOB**	X-linked	0	ChrX:43640740G>A	NM_000898.5: c.980C>T: p.T327M	**32**	**5.47**	**25.58**	**D**	**D**	Male, spastic quad, born 40 weeks, BW: 9th percentile IUGR, DD, kyphoscoliosis, visual problems (mother also), progressive muscle weakening, seizures as neonate, Ep as child

**P701**	**KANK1**	Not maternal	2.89E-05	Chr9:730224G>A	NM_153186.5: c.2398G>A: p.D800N	23.5	**5.91**	4.94	**D**	**D**	Male, spastic/dystonic diplegia, born 40 weeks, cerebral oedema, diagnosed neonatal encephalopathy, required anticonvulsants as neonate, DD, partial Ep seizure at 3yo, non-verbal. US: ventricles and the extra axial spaces are decreased in size most likely due to generalised cerebral oedema. No intracranial haemorrhage demonstrated.
**P707**	**ABLIM2**	Unknown	0	Chr4: 8037968delT	F/shift del NM_001286688.1: c.226del: p.Ser76Valfs*12	.	.	.	.	.	Female, spastic/dyskinetic quad, born 24 weeks, hyperkinetic, Hyaline Membrane Disease (Respiratory Distress Syndrome) after birth; required surgery for patent ductus. Necrotising enterocolitis as infant
	**DNAH3**	Unknown	8.31E-05	Chr16:21008634G>A	NM_017539.2: c.6572C>T: p.P2191L	**28.3**	**5.23**	**49.84**	**D**	**D**	
	**MGA**	Unknown	1.66E-04	Chr15:42034996C>T	NM_001164273.1: c.4838C>T: p.T1613I	23.4	4.96	50.50	P	**D**	
		Unknown	1.12E-04	Chr15:42019504T>C	NM_001080541.2: c.3557T>C: p.L1186S	22.8	4.71		P	N	
**P718**	**NT5C2**	Hom - IBD	0	Chr10:104899223G>A	Stopgain NM_001134373.2: c.115C>T: p.R39*	**36**	4.99	.	.	**A**	Female, spastic diplegia, born 36 weeks, advanced juvenile glaucoma, parents first cousins, DD, ASD, moderate ID, ADHD (Brother also), OCD, advanced juvenile glaucoma, severe behavioural problems including unmanageable oppositional behaviour, anxiety, bilateral paranasal sinus disease, MRI: PVL.
**P719**	**MCCC1**	Not maternal	0	Chr3:182763308C>T	NM_001293273.1: c.625G>A: p.D209N	**27.9**	**5.63**	**24.24**	**D**	**D**	Female, spastic hemi with dysarthria, born 36 weeks, BW: 2nd percentile IUGR, Ep, severe drooling, polycythaemia, DD and right visual field defect, unilateral cerebral thrombotic infarction. MRI: Large area of cystic encephalomalacia in the left MCA territory with significant attenuation of the left MCA and its branches. Small

											area of cystic encephalomalacia in the right MCA territory. Severe PVL with evidence of middle cerebral artery occlusion on the left side.
	**MIIP**	Maternal	0	Chr1:12090147G>T	NM_021933.4: c.908G>T: p.S303I	**26**	4.01	81.23	P	**D**	
**P720**	**GCDH**	Not maternal	4.06E-06	Chr19:13002704G>A	NM_000159.4: c.187G>A: p.D63N	**32**	**5.17**	85.19	P	**D**	Male, spastic hemi with some dystonia in left upper limb, born 40 weeks, intermittent right knee pain and headaches. MRI: Periventricular white matter loss and areas of abnormal signal in the periventricular white matter, consistent with PVL. Asymmetry in the size of the thalami.
**P721**	**DNAH3**	Maternal	2.03E-05	Chr16:21008662C>T	NM_017539.2: c.6544G>A: p.D2182N	**32**	**5.23**	**40.71**	**D**	**D**	Male, spastic/dystonic quad, dysphonia, breathing difficulty, progressive planovalgus deformity
	**KANK1**	Maternal	0	Chr9:738430G>A	NM_153186.5: c.3005G>A: p.G1002D	**33**	**5.37**	**39.50**	**D**	**D**	
**P724**	**L1CAM**	X-linked	0	ChrX:153132565G>A	NM_001143963.2: c.2137C>T: p.P713S	**27.2**	**5.77**	65.89	**D**	**D**	Male, spastic diplegia, born 40 weeks, BW: 2nd percentile IUGR, spastic paraparesis and agenesis of the corpus callosum, macrocephaly, X-linked hydrocephalus, Mother and 3 aunts are carriers, MRI and CT: Absent corpus callosum with associated colpocephaly, hypoplasia of the cortical mantle and gross ventriculomegaly. The cerebral aqueduct is narrow, and the dilated third and lateral ventricles are suggestive of aqueduct stenosis.
**P729**	**APOE**	Maternal	4.54E-04, familial hyperlipo-proteinemia	Chr19: 45412314T>A	NM_000041.4: c.761T>A: p.V254E	**27.6**	3.56	95.91	P	N	Male, spastic diplegia, born 33 weeks, anxiety. MRI and US: loss of white matter volume in the brain and periventricular white matter changes.
**P738**	**AGAP1**	De novo	1.94E-04	Chr2: 236877181C>G	NM_014914.4: c.1400C>G: p.P467R	**27.4**	4.21	77.01	P	**D**	Female, born 28 weeks, spastic diplegia, mild dystonic, TTTS, DD, identical twin (status unknown). MRI: Posterior periventricular changes consistent with PVL. A hypogenetic corpus callosum with

											an absent splenium. Linear lipoma over the corpus callosum.
**P739**	**CTNND2**	Maternal	2.76E-05	Chr5:11346700C>T	NM_001288716.1: c.401G>A: p.R134H	**34**	**5.79**	90.10	**D**	**D**	Male, spastic/dystonic quad, born 36 weeks, BW: <1 percentile IUGR. MRI: Bilateral nearly symmetrical periventricular white matter hyperintensity, predominantly involving the deep white matter, suggestive of a demyelination/dysmyelination disorder.
	**PROC**	Not maternal	3.26E-05	Chr2:128186457T>C	NM_000312.3: c.1321T>C: p.Y441H	**27**	**5.16**	68.83	**D**	**D**	
**P740**	**DYNC2H1**	Not paternal	0	Chr11:103116029C>G	NM_001377.3: c.9968C>G: p.T3323S	23	**5.64**	**36.17**	**D**	**D**	Male, spastic quad, born 28 weeks, lower limbs significantly more affected than the upper limbs.
	**UBQLN3**	Not paternal	0	Chr11:5530781T>G	NM_017481.4: c.8A>C: p.K3T	24.8	**5.38**	**41.16**	P	**D**	
**P743**	**MYO15A**	Not maternal	1.44E-05	Chr17:18042884C>T	NM_016239.4: c.5170C>T: p.R1724C	**27.9**	3.56	57.27	**D**	**D**	Female, dyskinetic quad, born 26 weeks, DD, hearing loss/deafness, chronic lung problems, progressive scoliosis. MRI: generalised bilateral cerebral atrophy.
**P744**	**EPHA1**	Maternal	1.63E-05	Chr7:143096819C>T	NM_005232.5: c.760G>A: p.E254K	**26.7**	4.33	50.99	**D**	**D**	Female, spastic/dystonic quad, born 41 weeks, BW: 1st percentile IUGR, antenatal seizures, brain malformation, ID, Ep, cataracts both eyes. MRI: Extensive right perisylvian, posterior frontal and parietal polymicrogyria. Polymicrogyria involving the parietal lobe and sylvian fissure on the left with superimposed previous insult, presumed ischaemia to the thalamus and external capsule on this side with generalised reduction in volume and size of the supplying vessels.
**P745**	**DNAH3**	Unknown	5.78E-05	Chr16:20986640G>A	NM_017539.2: c.8174C>T: p.T2725M	**26.9**	**5.73**	**46.44**	**D**	**D**	Male, spastic hemi.
**P746**	**MIIP**	Unknown	8.13E-06	Chr1:12089176G>A	NM_021933.4: c.634G>A: p.E212K	**33**	**5.28**	**41.90**	**D**	**D**	Female, spastic diplegia, difficulty with distant vision, planovalgus foot position

**P750**	**DNAH3**	Maternal	2.03E-05	Chr16:21049282G>A	NM_017539.2: c.4751C>T: p.A1584V	**31**	**5.76**	90.11	**D**	**D**	Male, dystonic diplegia, born 42 weeks, previous iron deficiency anaemia (resolved); has pulmonary stenosis (balloon dilation, aged 3 months).
**P758**	**KIF1C**	Not maternal	5.69E-05	Chr17:4905376G>A	NM_006612.6: c.386G>A: p.R129H	**27.7**	**5.45**	**20.11**	**D**	**D**	Male, spastic diplegia, born 40 weeks, ASD (cousin also), hearing loss (grandmother also), MRI: open lipped schizencephaly on the right and either transmantle heterotopia or closed lip schizencephaly demonstrated on the left, associated with large extra-axial CSF spaces. Unclear whether these are simply secondary to the malformation or represent arachnoid cysts. Suggestion of some mass effect on the left side with enlargement of the middle cranial fossa. Multiple areas of polymicrogyria, absence of the septum pellucidum and small optic nerves.
	**AP4M1**	Not maternal	0	Chr7:99699541T>C	NM_004722.3: c.97T>C: p.F33L	23	**5.26**	**35.68**	B	**D**	
**P759**	**KIF1C**	Maternal, no sample for Father	3.17E-05	Chr17:4927186C>T homozygous	NM_006612.6: c.3052C>T: p.R1018C	**29.6**	3.76	89.22	**D**	N	Male, spastic hemi, born 37 weeks.
**P772**	**HSPG2**	Maternal	8.31E-05	Chr1:22178609C>T	NM_005529.7: c.6842G>A: p.R2281H	**28.5**	**5.34**	88.52	**D**	**D**	Male, spastic/dystonic quadriplegic CP - very dystonic with lots of involuntary dsykinetic movements, born 37 weeks, DD, arthrogryposis affecting his lower limbs (mother also has arthrogryposis)
**P773**	**MAST1**	Not maternal	8.12E-06	Chr19:12963198G>A	NM_014975.3: c.1066G>A: p.D356N	23.2	**5.6**	70.89	P	**D**	Male, spastic diplegia, clumsy gait, born 28 weeks, ADHD, anxiety/depression (father also), bipolar disorder in 2x paternal aunts and grandmother, mother's cousin spina bifida, maternal aunt cleft lip.
**P776**	**MGA**	Unknown	4.06E-05	Chr15:42040936C>T	NM_001080541.2: c.4687C>T: p.P1563S	24.3	4.76	93.24	B	**D**	Male, asymmetric spastic diplegia, born 36 weeks, BW: 5th percentile IUGR, DD, ASD, ID and seizure disorder with consequent behaviour disturbances and aggressive behaviour. MRI: Bilateral periventricular white matter volume loss

											with signal changes as desribed, consistent with PVL. Mild cerebral cerebellar degenerative changes noted.
**P781**	**KIF1A**	De novo	0	Chr2:241715280G>A	NM_004321.7: c.C946T: p.R316W	**32**	2.35	**1.63**	**D**	**D**	Female, spastic/dystonic diplegia, more dystonic than spastic with intermittent hypertonicity, born 35 weeks, BW: 10th percentile, SGA, microcephaly, developmental disorders and cortical visual impairment, MRI and X-Ray: Non-specific hyperintensity within the posterior parietal deep white matter, and in the cerebelli, posterior to the dentate nuclei. Bilateral cortical heterotopia in the inferior occipital horns.
**P783**	**AP4E1**	Unknown	3.29E-05	Chr15: 51233946C>T	NM_001252127.1: c.925C>T: p.H309Y	**26.7**	**5.39**	77.33	**D**	**D**	Male, spastic hemi, born 38 weeks, BW: 2nd percentile IUGR, moderate DD, seizures at birth, tachycardia, hypoglycaemia, parents cousins
	**NAA35**	Unknown	0	Chr9:88571284T>C	NM_024635.3: c.134T>C: p.L45S	**27**	3.94	**28.97**	**D**	**D**	
**P785**	**MCCC1**	Unknown	0	Chr3:182733339G>C	NM_001293273.1: c.1714C>G: p.P572A	24.5	4.84	61.30	**D**	**D**	Female, spastic hemi, poor balance, speech delay, swallowing difficulties, vomits easily, leg lengths differ by 1cm
**P788**	**MIIP**	Unknown	0	Chr1:12090086G>C	NM_021933.4: c.847G>T: p.V283L	24.8	**5.15**	**27.76**	P	**D**	Male, dystonic quad, severe dysphagia, Ep, DD (brother/sister also), mother, brother visual problems, CT/MRI: postnatal herpetic meningoencephalitis
	**DMD**	Unknown	5.60E-06	ChrX:31222094C>T	NM_004015.2: c. 587A>G: p.R196Q	**29.8**	**5.35**	**27.27**	**D**	**D**	
**P789**	**EPHA1**	Not maternal	6.31E-05	Chr7:143095028G>A	NM_005232.5: c.1600C>T: p.R534W	**34**	3.82	**47.13**	**D**	**D**	Male, spastic hemi, born 40 weeks, antenatal seizures, Ep with complex partial seizures, right homonymous hemianopia; right sided visual defects; learning difficulties, mother polycystic kidney disease. CT: large old infarct involving the left MCA territory with associated ex vacuo dilatation of the left lateral ventricle. No identifiable hydrocephalus.

**P795**	**HSPG2**	Unknown	7.82E-06	Chr1:22181916C>G	NM_005529.7: c.5878G>C: p.V1960L	**25**	4.17	**27.91**	P	**D**	Female, spastic/dystonic diplegia, born 25 weeks, chronic lung disease from prematurity which required home oxygen; Ep; auditory processing and language problems; anxiety disorder, Continues to have trend for internal rotation at hips with some knee flexion and anterior pelvic tilt bilaterally; quite significant dystonia in calves.
**P799**	**MIIP**	Maternal	0	Chr1:12090086G>C	NM_021933.4: c.847G>T: p.V283L	24.8	**5.15**	**27.76**	P	**D**	Male, spastic hemi, born 38 weeks, Ep, ASD, ADHD, OCD, anxiety/depression, increasing aggression, microcephaly, DD, various visual problems including hemianopia and optic nerve damage. MRI 2 months: Right middle cerebral artery infarct with changes of porencephaly. Smaller capsular and basal ganglionic infarcts on the left side. Diffuse and generalised cortical volume loss. Thin and hypoplastic right MCA. MRI 4 years: gliotic change in the left periventricular region surrounding the encephalomalacic cavity has increased and evidence of wallerian degeneration has developed in the right thalamus. Extensive right middle artery terriotry cystic encephalomalacia and small right middle cerebral artery changes are stable. MRI 11 years: Extensive cystic encephalomalacia and gliosis in the right middle cerebral artery territory does not show significant interval change. Wallerian degeneration involving the bilateral thalami and brainstem as described. Bilateral paranasal sinus disease.
**P800**	**EIF4E2**	Unknown	0	Chr2:233431575C>T	NM_001276337.1: c.277C>T: p.R93W	**25.4**	**5.53**	85.54	P	**D**	Male, spastic hemi, born 39 weeks, BW: 7th percentile IUGR, Factor V Leiden deficiency, hearing loss (cousin also), use of right hand has declined.
	**ATP11B**	Unknown	0	Chr3:182607310G>T	NM_014616.3: c.2956G>T:	**28.7**	**5.7**	66.52	**D**	**D**	

					p.D986Y						
**P802**	**PCBP3**	Unknown	0	Chr21:47329390A>G	NM_001130141.1: c.461A>G: p.E154G	**33**	4.99	**15.32**	**D**	**D**	Female, spastic/dystonic hemi, born 40 weeks, visual problems.
**P904**	**SCN8A**	De novo, also present in identical twin	4.06E-06	Chr12:52188354C>T	NM_014191.3: c.4724C>T: p.A1575V	**34**	4.94	80.81	**D**	**D**	Male, spastic diplegia, born 28 weeks, identical twin with no known NDD, shared placenta, TTTS, ASD (cousin also), ADHD (cousin also), anxiety/depression, visual problems, MRI: mild PVL.
**P907**	**AUH**	Not maternal	6.49E-05	Chr9:94118175T>C	NM_001306190.1: c.408A>G: p.I136M	24.8	2.61	87.95	**D**	**D**	Male, spastic diplegia, born 28 weeks, BW: 9th percentile IUGR, twin, non-identical, DD. MRI: PVL.
**P915**	**HUWE1**	Not maternal, also present in identical twin	5.60E-06	ChrX:53596709A>T	NM_031407.7: c.6391T>A: p.L2131M	23.4	4.38	60.72	**D**	**D**	Female, spastic hemiplegia, born 35 weeks, BW: 6th percentile IUGR, identical twin, Ep, visual problems (Mother also), TTTS, plethora, phototherapy, polycythaemia. MRI: decreased volume of the right cerebral hemisphere and dilation of the posterior bodies of both lateral ventricles. Focal area of encephalomalacia in the right cerebral hemisphere in the right frontal parietal region adjacent to the central sulcus.
	**MAOB**	Not maternal, also present in identical twin	0	ChrX:43640740G>A	NM_000898.5: c.980C>T: p.T327M	**32**	**5.47**	**25.58**	**D**	**D**	
	**MYO15A**	Unknown	0	Chr17:18045504C>T	Stopgain NM_016239.4: c.5761C>T: p.R1921*	**39**	4.7	.	.	**A**	
**P919**	**UBQLN3**	Not paternal	4.70E-05	Chr11:5530127A>G	NM_017481.4: c.662T>C: p.L221P	**25**	**5.53**	73.17	P	P	Spastic hemi, born 28 weeks, left grade IV IVH.
**P928**	**AP4S1**	Unknown	0	Chr14:31535491T>A	NM_001128126.2: c.89T>A: p.L30H	24.4	**5.99**	98.62	**D**	N	Female, spastic diplegia, born 29 weeks, antepartum haemorrhage.
**P934**	**AP4E1**	Not maternal	0	Chr15: 51201028A>T	NM_007347.5: c.53A>T:	21.6	**5.39**	56.77	B	**D**	Male, spastic hemi, born 41 weeks. MRI: Focal area of gliosis involving the

					p.Q18L						posterior limb of the right internal capsule and the right centrum semiovale.
	**DNAH3**	Not maternal	3.26E-05	Chr16:21133303A>G	NM_017539.2: c.1547T>C: p.F516S	**27.6**	**5.21**	97.43	**D**	**D**	
**P936**	**MYO1F**	Not maternal	7.22E-06	Chr19:8618281T>C	NM_012335.3: c.367A>G: p.I123V	23.5	4.35		P	D	Male, Spastic diplegia, born 33 weeks, BW: 8th percentile IUGR, 2 sisters with ADHD and anxiety/depression.
**P947**	**ADCY3**	Unknown	4.07E-06	Chr2:25057679G>A	Stopgain NM_004036.4: c.1789C>T: p.R597*	**43**	4.21	.	.	**A**	Male, born 25 weeks, spastic/dystonic quad, DD, ventricular bleed at 6 weeks of age, uncle with ASD, MRI: PVL and IVH.
**P950**	**AP4E1**	Maternal	2.84E-05	Chr15:51250786A>G	NM_001252127.1: c.1421A>G: p.E474G	**25**	4.48	**38.29**	B	**D**	Male, spastic hemi, born 40 weeks, ASD, visual problems, antenatal seizures, parents related, relationship not specified, transposition of great arterial vessels, stroke as baby during heart operation.
	**MYO15A**	Maternal	0	Chr17:18077163 delCAGCT	F/shift del NM_016239.3: c.10419_10423 delCAGCT S3474Pfs*42	.	.	.	.	.	
**P959**	**MYO15A**	Unknown	2.74E-05	Chr17:18047111G>A	Splicing NM_016239.3: c.6046+1G>A	24.6	4.13	.	.	D	Male, spastic/dyskinetic quad, born 39 weeks, BW: <1 percentile IUGR, placenta infarcted. MRI: PVL.
**P963**	**MYO15A**	Unknown	0	Chr17:18045504C>T	Stopgain NM_016239.3: c.5761C>T: p.R1921*	**39**	4.7	.	.	**A**	Female, spastic hemi, born 29 weeks, CPAP in neonatal period.
**P968**	**BRWD3**	Unknown	0	ChrX:79951470C>T	NM_153252.4: c.3088G>A: p.V1030I	**33**	**5.45**	**10.54**	**D**	**D**	Female, dyskinetic/ spastic hemi, born 40 weeks, diagnosed neonatal encephalopathy, learning difficulty, ADHD, OCD.
	**DMD**	Unknown	1.68E-05	ChrX:31222190C>T	NM_004015.2: c.491G>A: p.R164H	**34**	4.47	53.39	**D**	**D**	
**P980**	**DNAH3**	Not maternal	8.30E-05	Chr16:20975977G>A	NM_017539: c.C9229T: p.R3077C	**29.7**	4.88	60.55	**D**	**D**	Male, spastic quad, born 26 weeks, DD, Ep, OCD. MRI: Grade IV germinal matrix haemorrhage with IVH.

**P981**	**COL4A1**	Not maternal	6.63E-05	Chr13:110831281G>A	NM_001845.6: c.2447C>T: p.P816L	22.7	4.89	59.22	**D**	**D**	Male, spastic diplegia, born 36 weeks, BW: 4th percentile IUGR, neonatal seizures, DD, visual problems, MRI and CT: Grade 2 sub-ependymal haemorrhage. CT demonstrates a germinal matrix haemorrhage with intraventricular extension.
**P1102**	**HUWE1**	Paternal	1.69E-05	ChrX:53579791T>A	NM_031407.7: c.8558A>T: p.E2853V	24.6	**5.88**	87.60	**D**	**D**	Female, spastic quad, born 40 weeks, BW: 5th percentile IUGR, neonatal seizures, OCD (mother also), parents both visual problems, hearing loss (grandparents also), father mainstream school in special class, MRI and US: remote parenchymal insult sustained in the perinatal period.
**P1108**	**KIF1C**	Not maternal	8.94E-05	Chr17:4924169G>A	NM_006612.6: c.2006G>A: p.R669Q	**34**	**5.68**	75.36	**D**	**D**	Female, spastic/dystonic hemi, born 39 weeks, Focal dyscognitive seizures, left convergent squint. MRI/CT: appearance is in keeping with a large porencephalic cyst in the left middle cerebral artery. White matter loss with dilated left lateral ventricle consistent with a remote insult.
	**MIIP**	Not maternal	4.51E-05	Chr1:12082359C>T	Stopgain NM_021933.4: c.322C>T: p.R108*	**35**	1.32	**10.80**	.	**A**	
**P1116**	**COL4A1**	Maternal	7.21E-05	Chr13:110804753C>T	NM_001845.6: c.4856G>A: p.R1619H	**26**	**5.51**	**5.51**	**D**	**D**	Female, spastic hemi, born 27 weeks, BW: 8th percentile IUGR, neonatal lung disease, subgliotic stenosis requiring tracheostomy, developed IVH grade IV soon after birth, hydrocephalus with papilledema developed at 12 months, pyloric stenosis, mild hearing loss, esotropia corrected by glasses, DD, MRI: R grade 4 and L grade 2 IVH. Persistent hydrocephalus, right porencephalic cyst, mild PVL posteriorly.
	**DNAH3**	Maternal	3.66E-05	Chr16:21086770T>C	NM_017539.2: c.3082A>G: p.T1028A	**25.5**	**6.02**	**13.68**	**D**	**D**	

**P1126**	**AGAP1**	Not maternal	0	Chr2:236817458C>T	NM_001037131.3: c.1232C>T: p.P411L	**33**	4.66	**11.42**	**D**	**D**	Male, spastic quad, born 30 weeks, DD, Ep, squint nystagmus, feeding difficulties, cholestasis, both parents and sister visual problems, brother ADHD. MRI: Bilateral grade III intraventricular haemorrhage with moderate asymmetric ventriculomegaly with mild rightward subfalcine herniation. Large-volume, bilateral periventricular cystic encephalomalacic cavities. Persistent haemorrhages within both cystic cavities is noted. Persistent bilateral large-volume expanded periventricular leukomalacic cysts which appear to communicate directly with the lateral ventricles. Moderate dilation of the lateral and third ventricles appears secondary to an acquired aqueduct stenosis; in addition, features of a prior basilar arachnoiditis may reflect abnormal CSF dynamics at the 4th ventricular outlet foramina and basilar cisterns.
**P1131**	**MYO15A**	Unknown	0	Chr17:18023972G>T	Stopgain NM_016239.4: c.1858G>T: p.E620*	**34**	3.51	**14.55**	.	**A**	Female, spastic triplegic, born 24 weeks, twin, required resuscitation at birth, IQ 52, IVH and secondary hydrocephalus requiring shunting before 6 months.
**P1136**	**MYO1F**	Unknown	0	Chr19:8586455C>T	NM_012335.3: c.3254G>A: p.G1085D	31	4.92		D	D	Female, spastic hemi, born 40 weeks, DD, Ep, visual impairment. MRI: encephalomalacia involving the left posterior temporal and frontal lobes secondary to old left MCA distribution infarction. This could be as a result of a perinatal injury. Wallerian degeneration involving the mid brain and pons.
**P1143**	**ABLIM2**	Maternal	3.29E-05	Chr4:8108337G>C	NM_001130083.2: c.38C>G: p.P13R	24.1	3.22	**25.08**	**D**	**D**	Male, spastic hemi, born 40 weeks, DD, Ep, left homonymous hemianopia. MRI: Extensive changes of gliosis with cystic encephalomalacia involving almost the entire right cerebral hemisphere with associated ex vacuo dilatation of the right lateral ventricle and changes of

											Wallerian degeneration in the right cerebral peduncle. Some non specific gliotic changes are also noted in the left periventricular white matter especially in the peritrigonal region.
**P1146**	**HSPG2**	Maternal	3.81E-05	Chr1:22190679C>T	NM_005529.7: c.4654G>A: p.G1552R	**29.7**	**5.1**	**0.57**	**D**	**D**	Male, spastic quad, twin, born 27 weeks, Ep (grandmother also), DD, anxiety/depression, visual problems (mother and grandparent also). CT/MRI: Neonatal ventricular haemorrhage. Shunted hydrocephalus. The changes are consistent with parenchymal gliosis particularly involving the right cerebral hemisphere and to a lesser extent involving the left cerebral hemisphere deep white matter and the left cerebellar hemisphere.
**P1147**	**IQSEC2**	Paternal	0	ChrX:53280005G>A	NM_001111125.3: c.1753C>T: p.R585W	**34**	**5.37**	84.99	**D**	**D**	Female, spastic quad, born 24 weeks, BW: 2nd percentile IUGR, dizygotic twin, hydrocephalus with VP shunt, Ep with seizure onset at 9 years, visual problems (both parents also).
**10078**	**KIF1A**	Not maternal	0	Chr2:241656789G>A	NM_004321.7: c.5065C>T: p.R1689W	**28.5**	-8.94	89.47	**D**	**D**	Female, born 40 weeks, father's second cousin CP, asthma and eczema.
**10135**	**DHX32**	Not maternal	4.72E-05	Chr10: 127548237G>A	NM_018180.2: c.784C>T: p.L262F	**25.8**	4.84	95.43	**D**	**D**	Female, possible Adams Oliver syndrome.
**10218**	**MYO15A**	Unknown	3.61E-05	Chr17:18066565G>A	NM_016239.4: c.9620G>A: p.R3207H	**25.1**	**5.25**	93.60	**D**	**D**	Female, born 32 weeks, BW: <1 percentile IUGR, twin
**10222**	**AGAP1**	Unknown	1.82E-05	Chr2: 237028942G>A	NM_014914.4: c.2062G>A: p.A688T	22.8	4.66	64.34	**D**	**D**	Male, born 40 weeks, DD, Ep
**10249**	**HUWE1**	Unknown	5.60E-06	ChrX:53596600G>A	NM_031407.7: c.6500C>T: p.A2167V	**25.1**	**5.24**	81.23	B	**D**	Female, hemiplegia, born 42 weeks, BW: 1st percentile IUGR, Ep, dandy walker malformation, Mother respiratory arrest at 16 weeks (asthma).
**10275**	**DNAH3**	Unknown	1.63E-05	Chr16:20981231C>T	NM_017539.2: c.8341G>A p.D2781N	**29.5**	**5.97**	66.00	**D**	**D**	Female, born 28 weeks, BW: 1st percentile IUGR

**10301**	**HSPG2**	Unknown	8.66E-05	Chr1:22168076T>C	NM_005529.7: c.9284A>G: p.N3095S	23.8	4.92	82.38	**D**	**D**	Female, born 39 weeks, BW: 2nd percentile IUGR
**10397**	**DDHD2**	Not maternal	3.25E-06	Chr8:38109665C>T	NM_001164232.1: c.1477C>T: p.P493S	24.2	**5.17**	**49.72**	P	**D**	Female, born 41 weeks, hydrocephalus, DD, Ep, vision impairment
	**TENM1**	Not maternal	0	ChrX:123518176C>T	NM_014253.2: c.6584G>A: p.R2195Q	**25.4**	**5.52**	88.08	**D**	**D**	
**10411**	**ERLIN1**	Unknown	0	Chr10:101937925C>T	NM_006459.4: c.269G>A: p.R90Q	**32**	**5.23**	**23.40**	P	**D**	Male, born 38 weeks, cousin with CP
**10528**	**KANK1**	Maternal	8.14E-06	Chr9:711556C>T	NM_153186.5: c.316C>T: p.R106C	**33**	4.89	**5.02**	**D**	**D**	Male, born 40 weeks, DD, Ep
**10572**	**KANK1**	Unknown	3.23E-05	Chr9:742272C>T	NM_153186.5: c.3290C>T: p.A1097V	**33**	**5.38**	65.02	**D**	**D**	Female, born 39 weeks, DD, developmental motor dyspraxia, scoliosis, ASD, depression
**10596**	**MGA**	Unknown	4.06E-05	Chr15:42040936C>T	NM_001080541.2: c.4687C>T: p.P1563S	24.3	4.76	93.24	B	**D**	Male, born 29 weeks, Mother rheumatic fever during pregnancy
**10635**	**PTGFRN**	Not maternal	1.02E-04	Chr1:117491978C>T	NM_020440.4: c.997C>T: p.R333W	**33**	4.59	**48.18**	**D**	N	Female, born 39 weeks, Ep, severe ID, congenital bilateral cataracts, soft cleft palate.
	**GAD1**	Not maternal	1.65E-05	Chr2:171709247C>T	NM_000817.3: c.1208C>T: p.P403L	**34**	**5.91**	**20.17**	**D**	**D**	
**10697**	**NPC1**	Unknown	4.07E-06	Chr18:21115499insT	F/shift ins NM_000271.5: c.3410dupA: p.N1137fs*121	.	.	.	.	.	Male, born 31 weeks, executive function disorder, semantic pragmatic disorder
**10744**	**DMD**	Unknown	0	ChrX:31196915G>T	Stopgain NM_004015.2: c.C890A: p.S297*	**57**	**5.45**	.	.	**A**	Female, born 40 weeks, twin
**10749**	**AUH**	Unknown	0	Chr9:94124018G>A	Stopgain NM_001306190.1: c. 154C>T: p. Q52*	**33**	1.68	76.87	.	**A**	Female, born 38 weeks, Ep
	**DNAH2**	Unknown	3.97E-05	Chr17:7661890C>T	NM_020877.3: c.2129C>T:	**34**	**5.91**	**4.61**	**D**	**D**	

					p.P710L						
**10750**	**NPC1**	Not maternal	4.06E-05	Chr18:21112188C>T	NM_000271.5: c.3815G>A: p.R1272H	**28.9**	**5.2**	95.47	**D**	**D**	Male, born 40 weeks, BW: 7th percentile IUGR
**10763**	**DNAH3**	Unknown	4.06E-06	Chr16:21139102C>T	NM_017539.2: 1114G>A: p.E372K	23.6	**5.42**	4.76	P	**D**	Female, born 39 weeks
**10768**	**MCCC1**	Unknown	1.58E-05	Chr3:182756852C>C	NM_001293273.1: c.988G>T: p.A330S	**29.2**	**5.82**	**31.96**	**D**	**D**	Male, born 40 weeks, DD, Ep, hypospadias and chordee of penis
**10786**	**ATP11B**	Unknown	1.63E-05	Chr3:182575775T>C	NM_014616.3: c.961T>C: p.W321R	**26.6**	2.61	64.61	B	**D**	Male, born 36 weeks, chronic lung disease, moderate-severe hearing loss
**10894**	**SREK1**	Not maternal	8.12E-06	Chr5:65460661C>T	NM_001077199.3: c.937C>T: p.R313C	24.3	**5.4**	91.68	**D**	**D**	Female, DD, microcephaly, ID, mother tested positive for CMV postnatally, reported IUGR and foetal distress
	**SYNGAP1**	Not maternal	0	Chr6:33412248C>G	NM_006772.2: c.3436C>G: p.P1146A	23.8	4.75	97.59	**D**	**D**	
**10908**	**MGA**	Unknown	8.16E-06	Chr15:42035281C>T	NM_001164273.1: c.5123C>T: p.S1708F	**26.8**	**5.11**	53.31	**D**	**D**	Female, born 29 weeks, nerve deafness, Father's first cousin mild CP
**10941**	**BRWD3**	Maternal	5.95E-06	ChrX:80064053T>G	NM_153252.4: c.165A>C: p.R55S	23.2	4.97	**16.00**	B	**D**	Female, born 27 weeks, GORD
	**DYNC2H1**	Not maternal	4.25E-06	Chr11:103043968A>G	NM_001080463.1: c.5492A>G: p.Y1831C	**26.8**	**5.65**	**36.11**	**D**	**D**	
	**HSPG2**	Not maternal	1.81E-05	Chr1:22156014G>A	NM_005529.7: c.11854C>T: p.R3952C	**35**	**5.37**	**18.62**	**D**	**D**	
**11014**	**DNAH3**	Unknown	0	Chr16:21145680G>A	NM_017539.2: c.982C>T: p.P328S	**27.9**	**5.85**	80.52	**D**	**D**	Male, born 40 weeks, ASD
**11039**	**MGA**	Maternal	4.88E-05	Chr15:42034954C>T	NM_001164273.1: c.4796C>T: p.T1599I	**26.8**	4.96	84.86	**D**	**D**	Male, spastic hemi, born 34 weeks, DD, Ep, velocardiofacial syndrome, tetralogy of fallot
**11111**	**DNAH3**	Not maternal	5.78E-05	Chr16:20986640G>A	NM_017539.2: c.8174C>T: p.T2725M	**26.9**	**5.73**	**46.44**	**D**	**D**	Female, born 35 weeks, BW: <1 percentile IUGR, DD, Ep

**11348**	**KDM7A**	Unknown	1.22E-05	Chr7:139829368C>A	NM_030647: c.G484T: p.V162F	**32**	**5.37**	**20.59**	**D**	**D**	Male, born 39 weeks, DD, Ep, visual loss (optic nerve loss)
**11451**	**ZMYM3**	Not maternal	0	ChrX:70470498C>T	NM_001171162.1: c.857G>A: p.R286H	**34**	4.9	**8.38**	B	P	Female, born 29 weeks, twin, both with CP.
**14986**	**MAST1**	Paternal	1.22E-05	Chr19:12958197G>A	NM_014975.3: c.421G>A: p.E141K	**28.3**	**5.01**	80.57	**D**	**D**	Male, ataxic CP, born 39 weeks, BW: 4th percentile IUGR, DD, speech difficulties, intention tremor, OCD, ASD features, anxiety, paternal grandfather early onset alzheimers, father possible milder speech and coordination problems.
**15065**	**KANK1**	Not maternal	9.02E-05	Chr9:745219G>A	NM_153186.5: c.3569G>A: p.R1190Q	**35**	**5.79**	**34.56**	**D**	**D**	Male, born 26 weeks, twin
**15410**	**PCBP3**	Maternal	3.97E-05	Chr21:47320983G>A	NM_001130141.1: c.295G>A: p.A99T	**25.9**	4.86	86.13	B	**D**	Female, born 30 weeks, DD
**16458**	**COPS3**	Not maternal	0	Chr17:17174300T>A	NM_001316356.1: c.7A>T: p.S3C	24.6	**5.52**	92.08	P	**D**	Male, twin, born 35 weeks, hydrocephalus
**16922**	**ADCY3**	Not maternal	2.04E-05	Chr2:25054544G>T	NM_004036.4: c.2042C>A: p.A681D	**26.7**	**5.4**	**49.08**	**D**	**D**	Male, born 38 weeks, maternal great aunt CP.

Abbreviations: A: Automatically annotated Disease causing, ADHD: Attention Deficit Hyperactivity Disorder, ASD: Autism Spectrum Disorder, BW: Birth weight, CADD Phred; Combined Annotation Dependent Depletion scaled score, CT: Computed Tomography scan, D: Damaging(PolyPhen2)/ Disease causing (MutationTaster), DD: Developmental Delay, Ep: Epilepsy, GERP++; Genomic Evolutionary Rate Profiling, gnomAD; genome aggregation database frequency, ID: Intellectual Disability, IUGR: Intrauterine Growth Restriction, IVH: Intraventricular haemorrhage, MCA: Middle Cerebral Artery, MRI: Magnetic Resonance Imaging, MTR centile; Missense Tolerance Ratio percentile, Mut Taster; Mutation Taster prediction, NDD: Neurodevelopmental Disorder, OCD: Obsessive Compulsive Disorder, PolyPhen2 HVAR; Polymorphism Phenotyping v2 Human Variation based prediction, PVL: Periventricular Leukomalacia, SGA: Small for gestational age, TTTS: Twin to Twin Transfusion Syndrome, US: Ultrasound.

Supplementary Table 6 Cases with variants of possible clinical significance in known disease genes

Sample	Gene	Inheritance	gnomAD	Variant (hg19)/Predicted effect	CADD Phred	GERP++	MTR centile	Poly-Phen2 HVAR	Mut. Taster	dbSNP ID, ACMG classification	Associated disorders (OMIM)	Clinical notes
**P009[1]**	***MAST1***	*De novo*	0	Chr19:12975755C>T NM_014975.3: c.1499C>T: p.P500L[2]	**32**	4.39	**3.63**	**D**	**D**	Likely pathogenic	Mega-corpus-callosum syndrome with cerebellar hypoplasia and cortical malformations (618273)	Male, spastic diplegia, born 33 weeks, resolved atrial septal defect, alternating squint and longsighted, white matter damage, mild learning problem, behavioural problems, sensitive hearing, drooling, dis/subluxation hips, cardiac incomplete right bundle branch block, mild pulmonary stenosis, asymmetrical gait pattern and short stride length, MRI: mild generalised volume loss, PVL, cystic encephalomalacia.
**P015[1]**	***PAK3***	X-linked	0	ChrX:110459718C>T NM_002578.5: c.1477C>T: p.R493C[2]	**34**	**5.54**	97.68	**D**	**D**	Uncertain significance	Non-syndromic X-linked intellectual disability (300558), Agenesis of the Corpus Callosum (activating mutations)	Male, dystonic spastic hemiplegia, born 29 weeks, BW: <1 percentile IUGR Ep, cognitive ability upper limit of low-average range, developed hydrocephalus, MRI: grade 4 IVH, right sided and grade 2 IVH left sided
**P026[1]**	***KDM5C***	De novo	0	ChrX:53240002G>A NM_001146702.1: c.1238C>T: p.P413L[2]	**28.6**	**5.42**	**3.28**	**D**	**D**	rs1057518697 Pathogenic	X-linked intellectual disability, syndromic, Claes-Jensen type, X-linked recessive (300534)	Female, spastic quad with greater involvement of lower limbs, born 39 weeks, BW: 5th percentile IUGR, small stature, convergent strabismus, ridging metopic suture, mild trigonocephaly, severe behaviour problems, speech dyspraxia, photophobia
**P033[1]**	***COL4A1***	Paternal	0	Chr13:110831315C>T NM_001845.5: c.2413G>A: p.G805R	24.6	4.70	84.91	**D**	**D**	Pathogenic	Porencephaly 1 (175780), Brain small vessel disease with or without ocular anomalies (607595), Angiopathy, hereditary, with nephropathy, aneurysms, and muscle cramps (611773)	Male, spastic quad with generalised hypotonia, dystonic posturing and myoclonic jerks, born 37 weeks severe ID, Ep, reduced oral control and tongue movement, MRI: PVL in frontal and parietal lobes (L>R), absent L caudate nucleus, putamen and globus pallidus, short stature at 4 years, died at 7 years.
**P035[1]**	***COL4A1***	Not maternal	8.12E-06	Chr13:110895030C>T NM_001303110.1: c.136G>A: p.G46R	**25.7**	4.26	73.83	**D**	**D**	Uncertain significance	Porencephaly 1 (175780), Brain small vessel disease with or without ocular	Female, spastic hemi, born 27 weeks, BW: <1 percentile IUGR, mild ID, impaired hearing, divergent squint, Ep, thyroid

											anomalies (607595), Angiopathy, hereditary, with nephropathy, aneurysms, and muscle cramps (611773)	agenesis, deterioration of motor function and bladder control; at 5 years, sleep apnoea, recurrent ear infections, PEG feeding, short stature and underweight at 16 years, US: L PVL, CT: mild ex-vacuo ventricular dilation.
**P052**[1]	***PROC***	Paternal	2.53E-05	Chr2:128178957C>T NM_000312.3: c.169C>T: p.R57W	**32**	3.33	.	**D**	**D**	rs757583846 Pathogenic	Thrombophilia due to protein C deficiency, autosomal recessive (612304)	Female, spastic/dyskinetic quad, born 38 weeks, ID, Ep, probable Gardner-Diamond syndrome, mild dysphagia, gradual but progressive decline neuro-developmentally, 4th child, 1st and 3rd female siblings deceased from obscure degenerative leukoencephalopathy, MRI: PVL, porencephaly, partial agenesis of corpus callosum (absent genu, rostrum, body with splenium present).
		Maternal	2.53E-05	Chr2:128185950C>T NM_000312.3: c.814C>T: p.R272C	.	.	**18.40**	**D**	**A**	rs121918154 Pathogenic	Thrombophilia due to protein C deficiency, autosomal recessive (612304)	
**P106**[1]	***COL4A1***	Paternal	0	Chr13:110813663T>C NM_001845.5: c.4516A>G: p.N1506D	20.3	4.56	**22.10**	P	**D**	Uncertain significance	Porencephaly 1 (175780), Brain small vessel disease with or without ocular anomalies (607595), Angiopathy, hereditary, with nephropathy, aneurysms, and muscle cramps (611773)	Male, spastic hemi, born 39 weeks, bilateral femoral anteversion
**P174**	***KIF1A***	Not maternal, Father unavailable	0	Chr2:241727535G>A NM_004321.7: c.296C>T: p.T99M	**31**	4.79	**5.96**	**D**	**D**	rs387906799 Pathogenic	ID, autosomal dominant 9 (614255)	Male, spastic quad, born 40 weeks, partial dysgenesis of corpus callosum, global DD, Ep (first seizure at 12 months), optic atrophy (cortically blind); type 1 diabetes, scoliosis in the lumbar region and an oblique pelvis, incontinent of faeces and urine, cortical atrophy has been progressive.
**P204**	***COL4A1***	Not maternal	0	Chr13:110830543C>T NM_001845.5: c.2494G>A: p.G832R	**27.8**	**5.52**	43.59	**D**	**D**	rs797044867 Pathogenic	Porencephaly 1 (175780), Brain small vessel disease with or without ocular anomalies (607595), Angiopathy, hereditary, with nephropathy,	Male, choreoathetoid quad, born 40 weeks, MRI: haemorrhagic gliosis, known COL4A1 mutation from diagnostic WES, cystic porencephaly involving basal ganglia; damage to small blood

											aneurysms, and muscle cramps (611773)	vessels with increased risk of haemorrhage; Epileptic encephalopathy, infantile spasms onset 1 year, evolution into mixed seizure types now; unexplained irritability; no speech or communication, global DD; tracheobronchomalacia; laryngomalacia; gastrostomy; fundoplication; scoliosis; left ventricular hypertrophy and hypertension; enlarged left kidney >10% discrepancy; dental caries; sleep apnoea; hip surgery, past femoral osteostomies; chronic lung disease; failure to thrive; hypoplastic optic nerves, MRI: haemorrhagic gliosis.
**P718**	***NT5C2***	Homozygous – Identical by descent	0	Chr10:104899223G>A Stopgain NM_001134373.2: c.115C>T: p.R39*	**36**	4.99	.	.	**A**	Pathogenic	Spastic paraplegia 45, autosomal recessive (613162)	Female, spastic diplegia, born 36 weeks, advanced juvenile glaucoma, parents first cousins, DD, ASD, moderate ID, ADHD (Brother also), OCD, advanced juvenile glaucoma, severe behavioural problems including unmanageable oppositional behaviour, anxiety, bilateral paranasal sinus disease, MRI: PVL.
**P724**	***L1CAM***	X-linked	0	ChrX:153132565G>A NM_001143963.2: c.2137C>T: p.P713S	**27.2**	**5.77**	65.89	**D**	**D**	Likely pathogenic	Partial agenesis of Corpus callosum (304100), CRASH syndrome (303350), Hydrocephalus due to aqueductal stenosis (307000)	Male, spastic diplegia, born 40 weeks, BW: 2nd percentile IUGR, spastic paraparesis and agenesis of the corpus callosum, macrocephaly, X-linked hydrocephalus, Mother and 3 aunts are carriers, MRI and CT: Absent corpus callosum with associated colpocephaly, hypoplasia of the cortical mantle and gross ventriculomegaly. The cerebral aqueduct is narrow, and the dilated third and lateral ventricles are suggestive of aqueduct stenosis.
**P773**	***MAST1***	Not maternal	8.12E-06	Chr19:12963198G>A NM_014975.3: c.1066G>A: p.D356N	23.2	**5.60**	70.89	P	**D**	Uncertain significance	Mega-corpus-callosum syndrome with cerebellar hypoplasia and cortical	Male, spastic diplegia, clumsy gait, born 28 weeks, ADHD, anxiety/depression (father also), bipolar disorder in 2x paternal aunts and

											malformations (618273)	grandmother, mother's cousin spina bifida, maternal aunt cleft lip.
**P781**	***KIF1A***	De novo	0	Chr2:241715280G>A NM_004321.7: c.946C>T: p.R316W	**32**	2.35	**1.63**	**D**	**D**	rs672601370 Pathogenic	ID, autosomal dominant 9 (614255)	Female, spastic/dystonic diplegia, more dystonic than spastic with intermittent hypertonicity, born 35 weeks, BW: 10th percentile, SGA, microcephaly, developmental disorders and cortical visual impairment, MRI and X-Ray: Non-specific hyperintensity within the posterior parietal deep white matter, and in the cerebelli, posterior to the dentate nuclei. Bilateral cortical heterotopia in the inferior occipital horns.
**P904**	***SCN8A***	De novo, also present in identical twin	4.06E-06	Chr12:52188354C>T NM_014191.3: c.4724C>T: p.A1575V	**34**	4.94	80.81	**D**	**D**	Uncertain significance	Epileptic encephalopathy, early infantile, 13 (614558)	Male, spastic diplegia, born 28 weeks, identical twin with no known NDD, shared placenta, TTTS, ASD (cousin also), ADHD (cousin also), anxiety/depression, visual problems, MRI: mild PVL.
**P915**	***HUWE1***	Not maternal, also present in twin	5.60E-06	ChrX:53596709A>T NM_031407.7: c.6391T>A: p.L2131M	23.4	4.38	60.72	**D**	**D**	Likely benign	Intellectual disability, X-linked syndromic, Turner type (300706)	Female, spastic hemiplegia, born 35 weeks, BW: 6th percentile IUGR, identical twin, Ep, visual problems (Mother also), TTTS, plethora, phototherapy, polycythaemia. MRI: decreased volume of the right cerebral hemisphere and dilation of the posterior bodies of both lateral ventricles. Focal area of encephalomalacia in the right cerebral hemisphere in the right frontal parietal region adjacent to the central sulcus.
**P968**	***BRWD3***	Unknown	0	ChrX:79951470C>T NM_153252.4: c.3088G>A: p.V1030I	**33**	**5.45**	**10.54**	**D**	**D**	Uncertain significance	Intellectual disability, X-linked 93 (300659)	Female, dyskinetic/ spastic hemi, born 40 weeks, diagnosed neonatal encephalopathy, learning difficulty, ADHD, OCD.
**P981**	***COL4A1***	Not maternal	6.63E-05	Chr13:110831281G>A NM_001845.5: c.2447C>T: p.P816L	22.7	4.89	59.22	**D**	**D**	Uncertain significance	Porencephaly 1 (175780), Brain small vessel disease with or without ocular anomalies (607595), Angiopathy, hereditary, with nephropathy,	Male, spastic diplegia, born 36 weeks, BW: 4th percentile IUGR, neonatal seizures, DD, visual problems, MRI and CT: Grade 2 sub-ependymal haemorrhage. CT demonstrates

											aneurysms, and muscle cramps (611773)	a germinal matrix haemorrhage with intraventricular extension.
**P1102**	***HUWE1***	Paternal	1.69E-05	ChrX:53579791T>A NM_031407.7: c.8558A>T: p.E2853V	**24.6**	**5.88**	87.60	**D**	**D**	Uncertain significance	Intellectual disability, X-linked syndromic, Turner type (300706)	Female, spastic quad, born 40 weeks, BW: 5th percentile IUGR, neonatal seizures, OCD (mother also), parents both visual problems, hearing loss (grandparents also), father mainstream school in special class, MRI and US: remote parenchymal insult sustained in the perinatal period.
**P1116**	***COL4A1***	Maternal	7.21E-05	Chr13:110804753C>T NM_001845.5: c.4856G>A: p.R1619H	**26**	**5.51**	**5.51**	**D**	**D**	Uncertain significance	Porencephaly 1 (175780), Brain small vessel disease with or without ocular anomalies (607595), Angiopathy, hereditary, with nephropathy, aneurysms, and muscle cramps (611773)	Female, spastic hemi, born 27 weeks, BW: 8th percentile IUGR, neonatal lung disease, subgliotic stenosis requiring tracheostomy, developed IVH grade IV soon after birth, hydrocephalus with papilledema developed at 12 months, pyloric stenosis, mild hearing loss, esotropia corrected by glasses, DD, MRI: R grade 4 and L grade 2 IVH. Persistent hydrocephalus, right porencephalic cyst, mild PVL posteriorly.
**P1147**	***IQSEC2***	Paternal	0	ChrX:53280005G>A NM_001111125.3: c.1753C>T: p.R585W	**34**	**5.37**	84.99	**D**	**D**	Likely benign	X-linked ID (309530)	Female, spastic quad, born 24 weeks, BW: 2nd percentile IUGR, dizygotic twin, hydrocephalus with VP shunt, Ep with seizure onset at 9 years, visual problems (both parents also).
**10249**	***HUWE1***	Unknown	5.60E-06	ChrX:53596600G>A NM_031407.7: c.6500C>T: p.A2167V	**25.1**	**5.24**	81.23	B	**D**	Uncertain significance	Intellectual disability, X-linked syndromic, Turner type (300706)	Female, hemiplegia, born 42 weeks, BW: 1st percentile IUGR, Ep, dandy walker malformation, Mother respiratory arrest at 16 weeks (asthma).
**10894**	***SYNGAP1***	Not maternal	0	Chr6:33412248C>G NM_006772.2: c.3436C>G: p.P1146A	23.8	4.75	97.59	**D**	**D**	Uncertain significance	ID, Autosomal dominant (612621)	Female, DD, microcephaly, ID, mother tested positive for CMV postnatally, reported IUGR and foetal distress
**14986**	***MAST1***	Paternal	1.44E-05	Chr19:12958197G>A NM_014975.3: c.421G>A: p.E141K	**28.3**	**5.01**	80.57	**D**	**D**	Uncertain significance	Mega-corpus-callosum syndrome with cerebellar hypoplasia and cortical malformations (618273)	Male, ataxic CP, born 39 weeks, BW: 4th percentile IUGR, DD, speech difficulties, intention tremor, OCD, ASD features, anxiety, paternal grandfather early onset alzheimers, father possible milder speech and coordination problems.

[1,] Case reported in [1], [2,] Variant reported in [1]. Abbreviations: A: Automatically annotated Disease causing, ADHD: Attention Deficit Hyperactivity Disorder, ASD: Autism Spectrum Disorder, BW: Birth weight, CADD Phred; Combined Annotation Dependent Depletion scaled score, CT: Computed Tomography scan, D: Damaging(PolyPhen2)/ Disease causing (MutationTaster), DD: Developmental Delay, Ep: Epilepsy, GERP++; Genomic Evolutionary Rate Profiling, gnomAD; genome aggregation database frequency, ID: Intellectual Disability, IUGR: Intrauterine Growth Restriction, IVH: Intraventricular haemorrhage, MRI: Magnetic Resonance Imaging, MTR centile; Missense Tolerance Ratio percentile, Mut Taster; Mutation Taster prediction, NDD: Neurodevelopmental Disorder, OCD: Obsessive Compulsive Disorder, PolyPhen2 HVAR; Polymorphism Phenotyping v2 Human Variation based prediction, PVL: Periventricular Leukomalacia, SGA: Small for gestational age, TTTS: Twin to Twin Transfusion Syndrome, US: Ultrasound.

Supplementary Table 7 Variation intolerant CP candidate genes harbouring variants of potential clinical significance (this study and [1])

Gene	Sample	Inheritance	gnomAD frequency	Variant (hg19)/ Predicted effect	CADD Phred	GERP++	MTR centile	Poly-Phen2 HVAR	Mut. Taster	dbSNP ID, ACMG classification	Clinical notes
ADCY3	**P443[1]**	Paternal	0	Chr2: 25064186G>A NM_004036.4: c.1138C>T: p.R380W[2]	**33**	4.60	**0.79**	**D**	**D**	Uncertain significance	Female, spastic hemi, born 39 weeks, maternal uncle severe physical and mental disability.
	**P947**	Unknown	4.07E-06	Chr2:25057679G>A Stopgain NM_004036.4: c.1789C>T: p.R597*	**43**	4.21	.	.	**A**	Uncertain significance	Male, born 25 weeks, spastic/dystonic quad, DD, ventricular bleed at 6 weeks of age, uncle with ASD, MRI: PVL and IVH.
	**16922**	Not maternal	2.04E-05	Chr2:25054544G>T NM_004036.4: c.2042C>A: p.A681D	**26.7**	**5.40**	49.08	**D**	**D**	Uncertain significance	Male, born 38 weeks, maternal great aunt CP.
AGAP1	**P033[1]**	**De novo**	0	Chr2: 236708167G>A Splicing NM_014914.4: c.957+1G>A[2]	**27.3**	**5.08**	.	.	**D**	Uncertain significance	Male, spastic quad with generalised hypotonia, dystonic posturing and myoclonic jerks, born 37 weeks, severe ID, Ep, reduced oral control and tongue movement, MRI: PVL in frontal and parietal lobes (L>R), absent L caudate nucleus, putamen and globus pallidus, short stature at 4 years, died at 7 years
	**P738**	**De novo**	1.94E-04	Chr2: 236877181C>G NM_014914.4: c.1400C>G: p.P467R	**27.4**	4.21	77.01	**P**	**D**	Uncertain significance	Female, born 28 weeks, spastic diplegia, mild dystonic, TTTS, DD, identical twin (status unknown). MRI: Posterior periventricular changes consistent with PVL. A hypogenetic corpus callosum with an absent splenium. Linear lipoma over the corpus callosum.
	**P1126**	Not maternal	0	Chr2:236817458C>T NM_001037131.3: c.1232C>T: p.P411L	**33**	4.66	**11.42**	**D**	**D**	Uncertain significance	Male, spastic quad, born 30 weeks, DD, Ep, squint nystagmus, feeding difficulties, cholestasis, both parents and sister visual problems, brother ADHD. MRI: Bilateral grade III intraventricular haemorrhage with moderate asymmetric ventriculomegaly with mild rightward subfalcine herniation. Large-volume, bilateral periventricular cystic encephalomalacic cavities. Persistent haemorrhages within both cystic cavities is noted. Persistent bilateral large-volume expanded periventricular leukomalacic cysts which appear to communicate directly with the lateral ventricles. Moderate dilation of the lateral and third ventricles appears secondary to an acquired aqueduct stenosis; in addition, features of a prior basilar arachnoiditis may reflect abnormal CSF dynamics at the 4th ventricular outlet foramina and basilar cisterns.
COPS3	**16458**	Not maternal	0	Chr17:17174300T>A NM_001316356.1:	**24.6**	**5.52**	92.08	**P**	**D**	Uncertain significance	Male, twin, born 35 weeks, hydrocephalus

				c.7A>T: p.S3C							
GAD1	**P176**	Not maternal	4.06E-06	Chr2:171702037A>G NM_000817.3: c.773A>G: p.Y258C	**26.3**	**5.67**	33.11	**D**	**D**	Uncertain significance	Male, spastic hemi, born 24 weeks, porencephalic cyst, Ep, global DD, mild ASD, limited speech, Left hand with increased tone; left ankle/foot orthotic; independent walker. MRI: IVH grade IV.
	**10635**	Not maternal	1.65E-05	Chr2:171709247C>T NM_000817.3: c.1208C>T: p.P403L	**34**	**5.91**	**20.17**	**D**	**D**	Uncertain significance	Female, born 39 weeks, Ep, severe ID, congenital bilateral cataracts, soft cleft palate.
INHBB	**P058[1]**	**De novo**	8.21E-06	Chr2:121106698C>T NM_002193.4: c.472C>T: p.R158C[2]	**26.2**	**5.09**	30.77	B	**D**	Uncertain significance	Male, spastic quad, born 38 weeks, mild ID, Epilepsy, DD, congenital CMV infection, generalized polymicrogyria, several febrile seizures
KDM7A	**P105[1]**	**De novo**	0	Chr7:139796549G>C NM_030647.2: c.2180C>G: p.S727W[2]	**34**	**5.85**	36.31	**D**	**D**	Uncertain significance	Female, spastic/dystonic quad, born 38 weeks, BW: 5th percentile IUGR, more signs on right than left, more dystonia than spasticity, born at 38 weeks after IUGR, neonatal hypoglycaemia, and subsequent neonatal seizures. ID, Ep with focal epileptiform abnormality, focal seizures starting at day 7, febrile clonic tonic seizure at 2 years. US: echogenicity of periventricular white matter and left thalamus. MRI: extensive PVL, Haemorrhagic foci as well as foci of restricted diffusion, majority of the pathology is likely to have occurred prior to delivery.
	**11348**	Unknown	1.22E-05	Chr7:139829368C>A NM_030647.2: c.484G>T: p.V162F	**32**	**5.37**	**20.59**	**D**	**D**	Uncertain significance	Male, born 39 weeks, DD, Ep, visual loss (optic nerve loss)
MAOB	**P025[1]**	X-linked	0	ChrX:43634520C>T Splicing NM_000898.5: c.1138-1G>A[2]	23.8	**6.17**	.	.	**D**	Uncertain significance	Male, spastic hemi, severe sleep disturbance, anxiety issues, behaviour problems, Irregularity and dilation of the left lateral ventricle associated with periventricular white matter volume loss and thinning of the corpus callosum.
	**P216**	X-linked	0	ChrX:43640740G>A NM_000898.5: c.980C>T: p.T327M	**32**	**5.47**	**25.58**	**D**	**D**	Uncertain significance	Male, spastic quad, born 40 weeks, BW: 9th percentile IUGR, DD, kyphoscoliosis, visual problems (mother also), progressive muscle weakening, seizures as neonate, Ep as child
	**P915**	Not maternal, also present in twin	0	ChrX:43640740G>A NM_000898.5: c.980C>T: p.T327M	**32**	**5.47**	**25.58**	**D**	**D**	Uncertain significance	Female, spastic diplegia, born 35 weeks, BW: 7th percentile IUGR, identical twin, Ep, visual problems (Mother also), TTTS, MRI: decreased volume of the right cerebral hemisphere and dilation of the posterior bodies of both lateral ventricles. Focal area of encephalomalacia in the right cerebral hemisphere in the right frontal parietal region adjacent to the central sulcus.
NAA35	**P117[1]**	**De novo**	0	Chr9:88631481G>A NM_024635.3: c.1596G>T: p.W532C[#]	**33**	**5.40**	**20.14**	**D**	**D**	Uncertain significance	Male, spastic hemi, born 39 weeks, borderline ID, refractory focal seizures and a left homonymous hemianopia, unilateral cerebral thrombotic infarction. US: increased echogenicity right thalamus, basal ganglia and deep white matter.

											MRI: extensive right MCA territory perinatal infarct with extensive encephalomalacia involving the central regions, frontal, parietal and occipital lobes. Right brain size smaller. Cystic changes. Right lateral ventricle dilated. Temporal lobe is of a reduced volume.
	**P783**	Unknown	0	Chr9:88571284T>C NM_024635.3: c.134T>C: p.L45S	**27**	3.94	28.97	**D**	**D**	Uncertain significance	Male, spastic hemi, born 38 weeks, BW: 2nd percentile IUGR, moderate DD, seizures at birth, tachycardia, hypoglycaemia, parents cousins
RNF214	**P007**[1]	**De novo**	0	Chr11:117152637C>T NM_001077239.1: c.1363C>T: p.P455S[2]	**23.4**	4.76	97.16	**D**	**D**	Uncertain significance	Female, mild ataxic / spastic diplegia, born 32 weeks, BW: <1 percentile IUGR, borderline IQ, twin, occipital horns are mildly plump (colpocephaly), behavioural outbursts and temper tantrums.
SLC6A3	**P042**[1]	Not maternal	0	Chr5:1411428G>A NM_001044.5: c.1199C>T: p.T400M	18.55	4.69	**11.78**	**D**	**D**	Uncertain significance	Female, spastic hemi, born 30 weeks, BW: <1 percentile IUGR, DD, mild ID, Ep (focal seizures), respiratory distress syndrome, behaviour problems.
	**P082**[1]	Paternal	0	Chr5:1443060G>A NM_001044.5: c.253C>T: p.R85W	**32**	1.46	**8.54**	**D**	**D**	rs1064795122 Likely Pathogenic	Female, spastic quad, born 38 weeks, BW: 2nd percentile IUGR, hearing & vision loss, microcephaly, swallowing issues (at risk of aspiration), CMV diagnosed by blood test at 1 year old
SREK1	**P436**[1]	**De novo**	4.09E-06	Chr5:65449298C>T NM_001077199.3: c.169C>T: p.P57S[2]	18.21	**5.61**	**7.20**	P	**D**	Uncertain significance	Female, spastic diplegia, BW: 4th percentile IUGR, twin, born 29 weeks
	**10894**	Not maternal	8.12E-06	Chr5:65460661C>T NM_001077199.3: c.937C>T: p.R313C	**24.3**	**5.40**	91.68	**D**	**D**	Uncertain significance	Female, DD, microcephaly, ID, mother tested positive for CMV postnatally, reported IUGR and foetal distress
TENM1	**P026**[1]	Paternal	0	ChrX:123518642C>G NM_014253.3: c.6118G>C: p.D2040H[2]	**23.6**	**5.44**	**14.87**	**D**	**D**	Uncertain significance	Female, spastic diplegia/spastic quadriparesis with greater involvement of lower limbs, born 39 weeks, BW: 5th percentile IUGR, small stature, convergent strabismus, ridging metopic suture, mild trigonocephaly, severe behaviour problems, speech dyspraxia, photophobia
	**10397**	Not maternal	0	ChrX:123518176C>T NM_014253.3: c.6584G>A: p.R2195Q	**25.4**	**5.52**	88.08	**D**	**D**	Uncertain significance	Female, born 41 weeks, hydrocephalus, DD, Ep, vision impairment
UBXN7	**P067**[1]	**De novo**	0	Chr3: 196120470C>T NM_015562.2: c.310G>A: p.A104T	**20.1**	4.47	62.04	P	**D**	Uncertain significance	Female, spastic diplegia, born 27 weeks, US: bilateral grade II IVH, then PVL. MRI: PVL. Bilateral increase in white matter signal in the periventricular regions most marked in the occipital areas and centrum semiovale regions consistent with mild changes of periventricular leukomalacia with minor thinning of the corpus callosum posteriorly.
ZMYM3	**11451**	Not maternal	0	ChrX:70470498C>T NM_001171162.1:	**34**	4.90	**8.38**	B	**P**	Uncertain significance	Female, born 29 weeks, twin, both with CP.

c.857G>A: p.R286H

[1,] Case reported in [1], [2,] Variant reported in [1]. Abbreviations: Abbreviations: A: Automatically annotated Disease causing, ADHD: Attention Deficit Hyperactivity Disorder, ASD: Autism Spectrum Disorder, BW: Birth weight, CADD Phred; Combined Annotation Dependent Depletion scaled score, CT: Computed Tomography scan, D: Damaging(PolyPhen2)/ Disease causing (MutationTaster), DD: Developmental Delay, Ep: Epilepsy, GERP++; Genomic Evolutionary Rate Profiling, gnomAD; genome aggregation database frequency, ID: Intellectual Disability, IUGR: Intrauterine Growth Restriction, IVH: Intraventricular haemorrhage, MCA: Middle Cerebral Artery, MRI: Magnetic Resonance Imaging, MTR centile; Missense Tolerance Ratio percentile, Mut Taster; Mutation Taster prediction, NDD: Neurodevelopmental Disorder, OCD: Obsessive Compulsive Disorder, PolyPhen2 HVAR; Polymorphism Phenotyping v2 Human Variation based prediction, PVL: Periventricular Leukomalacia, SGA: Small for gestational age, TTTS: Twin to Twin Transfusion Syndrome, US: Ultrasound.

Supplementary Table 8 Statistical significance of rare variants identified in candidate genes compared to controls.

Gene	MAF <0.001 in 271 cases	Number confirmed de novo	Frequency of variants with MAF<0.001 in EUR	Genome Wide Significance (Bonferroni Correction for number of genes tested)
**AGAP1**	8	1	0.006	**3.33E-04**
**ERLIN1**	3	0	0.0002	**4.03E-03**
**ZDHHC9**	3	0	0.0002	**4.03E-03**
**PROC**	5	0	0.002	**4.59E-02**
**KIF1A**	9	1	0.03	0.075
**SCN8A**	4	1	0.02	0.128
**TENM1**	11	0	0.001	0.146
**MYO1F**	11	0	0.0001	0.147
**ENPP4**	5	0	0.004	0.9
**ABLIM2**	3	0	0.02	1
**ACADM**	3	0	0.01	1
**ACOX1**	3	0	0.008	1
**ADCY3**	6	0	0.02	1
**ADD3**	2	0	0.01	1
**AP4B1**	2	0	0.01	1
**AP4E1**	6	0	0.02	1
**AP4M1**	5	0	0.01	1
**AP4S1**	2	0	0.004	1
**APOE**	2	0	0.01	1
**ATL1**	1	0	0.002	1
**ATP11B**	6	0	0.01	1
**AUH**	4	0	0.006	1
**BRWD3**	3	0	0.006	1
**CD99L2**	3	0	0.004	1
**CDK17**	2	0	0.006	1
**CNDP2**	2	0	0.008	1
**CNKSR2**	2	0	0.002	1
**COL4A1**	12	0	0.02	1

Gene	MAF <0.001 in 271 cases	Number confirmed de novo	Frequency of variants with MAF<0.001 in EUR	Genome Wide Significance (Bonferroni Correction for number of genes tested)
**COPS3**	3	0	0.008	1
**CTDSPL**	2	0	0.004	1
**CTNND2**	5	0	0.02	1
**CUL4B**	1	0	0.002	1
**DDHD2**	4	0	0.02	1
**DHX32**	2	0	0.01	1
**DMD**	19	0	0.05	1
**DMPK**	7	0	0.02	1
**DNAH2**	29	0	0.08	1
**DNAH3**	30	0	0.1	1
**DYNC2H1**	17	0	0.07	1
**EIF4E2**	3	0	0.006	1
**ENOX2**	0	0	0.008	1
**ENTPD1**	2	0	0.01	1
**EPHA1**	6	0	0.03	1
**GAD1**	4	0	0.006	1
**GANC**	13	0	0.02	1
**GCDH**	3	0	0.02	1
**HLCS**	3	0	0.01	1
**HPRT1**	1	0	0.0002	1
**HSPG2**	41	0	0.2	1
**HUWE1**	10	0	0.02	1
**IGSF1**	4	0	0.01	1
**IL6**	0	0	0.006	1
**IL1RAPL1**	3	0	0.004	1
**IQSEC2**	3	0	0.008	1
**INHBB**	0	0	0.006	1
**JADE1**	2	0	0.002	1

Gene	MAF <0.001 in 271 cases	Number confirmed de novo	Frequency of variants with MAF<0.001 in EUR	Genome Wide Significance (Bonferroni Correction for number of genes tested)
**KANK1**	11	0	0.04	1
**KDM5C**	3	0	0.004	1
**KDM7A**	4	0	0.01	1
**KIF1C**	9	0	0.05	1
**L1CAM**	6	0	0.02	1
**LCP2**	2	0	0.01	1
**MAN2A1**	6	0	0.02	1
**MAOB**	0	0	0.008	1
**MAST1**	4	0	0.02	1
**MC2R**	3	0	0.01	1
**MCCC1**	7	0	0.02	1
**MCPH1**	9	0	0.03	1
**MECP2**	1	0	0.01	1
**MGA**	12	0	0.05	1
**MIIP**	9	0	0.02	1
**MTMR1**	2	0	0.002	1
**MYO15A**	31	0	0.1	1
**NAA35**	2	0	0.01	1
**NEMF**	2	0	0.03	1
**NGFR**	0	0	0.008	1
**NPC1**	12	0	0.02	1
**NR1I2**	0	0	0.006	1
**NT5C2**	1	0	0.008	1
**OFD1**	1	0	0.01	1
**PAFAH1B1**	2	0	0.002	1
**PAK3**	3	0	0.006	1
**PCBP3**	3	0	0.008	1
**PLA2G6**	6	0	0.02	1
**PLP1**	0	0	0.0002	1
**PTGFRN**	6	0	0.03	1
**RAD21**	2	0	0.02	1
**RFX2**	5	0	0.05	1
**RNF214**	3	0	0.02	1
**SLC16A2**	3	0	0.002	1
**SLC6A3**	7	0	0.02	1
**SPAST**	4	0	0.01	1
**SREK1**	3	0	0.008	1
**SYNGAP1**	3	0	0.02	1
**TH**	3	0	0.02	1
**TMEM150A**	1	0	0.0002	1
**TUBA1A**	0	0	0.0002	1
**TUBA3D**	2	0	0.006	1
**TUBA8**	3	0	0.006	1
**TUBB**	0	0	0.0002	1
**TUBB2B**	0	0	0.0002	1
**TUBB3**	0	0	0.02	1
**TUBG1**	0	0	0.002	1
**UBE3A**	0	0	0.008	1
**UBQLN3**	5	0	0.04	1
**UBXN7**	1	0	0.002	1
**UPF3B**	1	0	0.002	1
**USP26**	4	0	0.006	1
**VRK1**	3	0	0.006	1
**WIPI2**	2	0	0.008	1
**ZC4H2**	1	0	0.002	1
**ZMYM3**	3	0	0.002	1

Significance was calculated using SORVA [8] for cases not previously analysed by whole exome sequencing as described in methods.

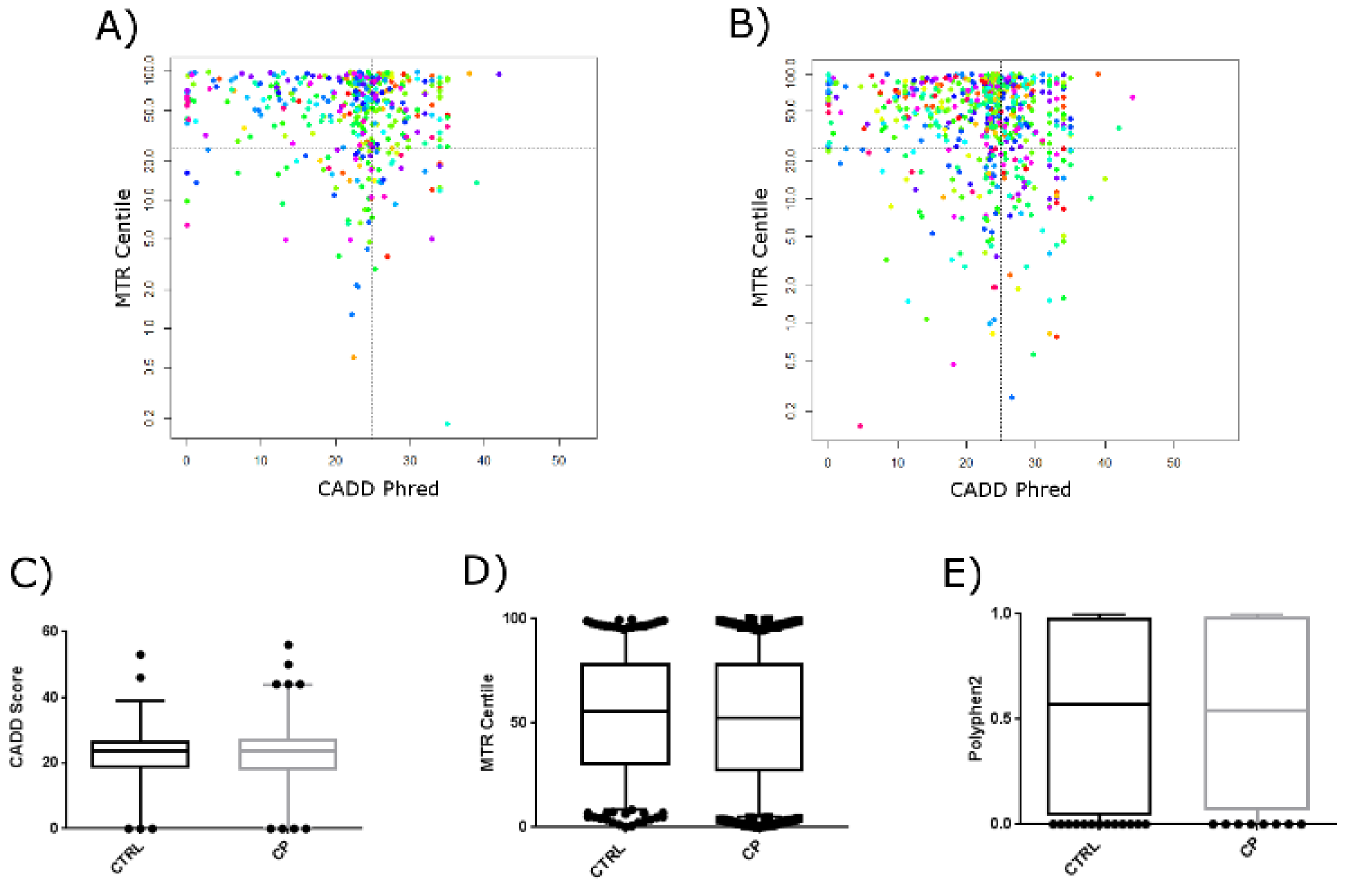

Supplementary Figure 4: Comparison of predicted pathogenicity of rare variants in cerebral palsy cases compared to controls from the EUR subset of 1000 genomes data. A-B) Scatterplot of CADD Phred scores vs MTR centile for rare (ExAC frequency <0.0001) variants in EUR 1000 genome cases (A) compared to rare variants in cerebral palsy cases (B). Plots are coloured by gene. **C-E)** Boxplots showing the distribution of predicted pathogenicity of variants in 1000 genome controls compared to cerebral palsy cases. Whiskers show 5th and 95th percentile in each case. There is no significant difference in the distribution of CADD Phred scores (C), MTR centile (D) or PolyPhen2 scores (E) of rare variants in controls compared to CP cases (P>0.05, unpaired t-test).

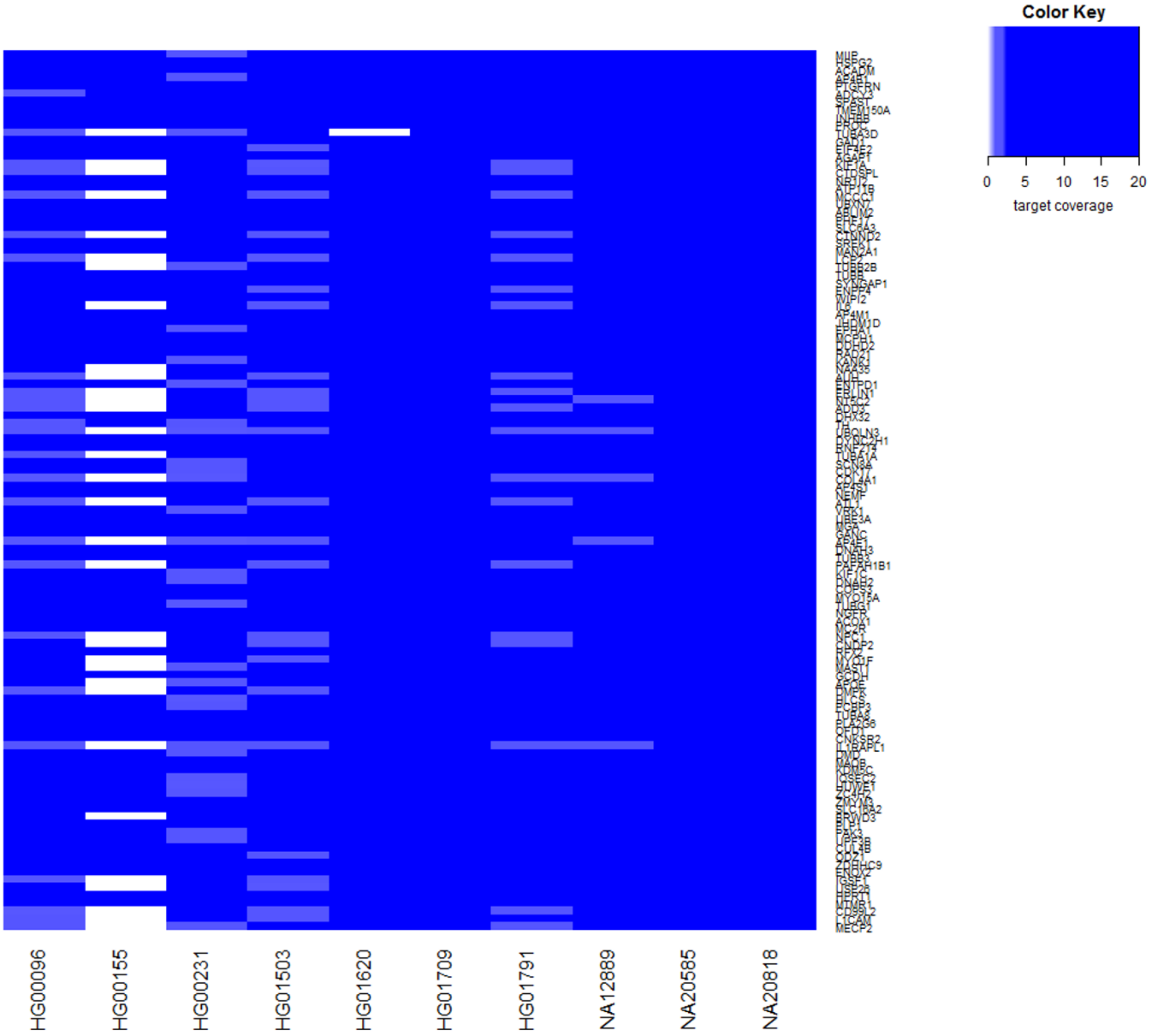

Supplementary Figure 5: Coverage of HaloPlex target regions in a random sample of 10 individuals from the EUR cohort of the 1000 Genomes Project. Coverage was calculated per base for all target bases in the HaloPlex design then aggregated to give median coverage per target gene.

Supplementary Table 9 Statistical analysis of differences in distance moved by 4dpf zebrafish larvae (Figure 1d)**.** Distance moved was measured during 1 minute in the dark as described in methods and significance was calculated using the Kruskal-Wallis test.

	**Control^{MO} + AGAP1^{mRNA}**	**AGAP1^{MO} + Control^{mRNA}**	**AGAP1^{MO} + AGAP1^{mRNA}**
**Control^{MO} + Control^{mRNA}**	p>0.9999	p=0.0002	p=0.0789
**Control^{MO} + AGAP1^{mRNA}**	-	p=0.0004	p=0.0952
**AGAP1^{MO} + Control^{mRNA}**	-	-	p<0.0001

Supplementary Table 10 Statistical analysis of differences in trajectory of 4dpf zebrafish following tapping stimulus (Figure 1e)**.** Trajectory was manually scored from recorded video as described in methods and significance of differences in categorical groupings calculated using Chi-square tests with 2 degrees of freedom. All p values are two-tailed.

	**Control^{MO} + AGAP1^{mRNA}**	**AGAP1^{MO} + Control^{mRNA}**	**AGAP1^{MO} + AGAP1^{mRNA}**
**Control^{MO} + Control^{mRNA}**	Chi-square=4.687 p=0.0960 (ns)	Chi-square=51.56 p<0.0001 (****)	Chi-square=10.75 p=0.0046 (**)
**Control^{MO} + AGAP1^{mRNA}**	-	Chi-square=92.1 p<0.0001 (****)	Chi-square=27.19 p<0.0001 (****)
**AGAP1^{MO} + Control^{mRNA}**	-	-	Chi-square=15.7 p=0.0004 (***)

Supplementary Movie 1: Touch-elicited startle response in 72hpf zebrafish larvae injected with 1mM Control morpholino compared to 0.5mM AGAP1 morpholino. Control morpholino injected larvae show a characteristic touch-elicited startle response, scooting rapidly away from the stimulus. In some cases *AGAP1* morphant larvae displayed rigidity and a failed escape response when touched.

Supplementary References:

1 McMichael, G. *et al.* Whole-exome sequencing points to considerable genetic heterogeneity of cerebral palsy. *Molecular psychiatry* **20**, 176-182 (2015).

2 McMichael, G. *et al.* Rare copy number variation in cerebral palsy. *European journal of human genetics* (2013).

3 Quinodoz, M. *et al.* DOMINO: Using Machine Learning to Predict Genes Associated with Dominant Disorders. *American journal of human genetics* **101**, 623-629 (2017).

4 Huang, N., Lee I Fau - Marcotte, E. M., Marcotte Em Fau - Hurles, M. E. & Hurles, M. E. Characterising and predicting haploinsufficiency in the human genome. *PLoS Genet* 6(10): e1001154. (2010).

5 Lek, M. *et al.* Analysis of protein-coding genetic variation in 60,706 humans. *Nature* **536**, 285-291 (2016).

6 Petrovski, S., Wang, Q., Heinzen, E. L., Allen, A. S. & Goldstein, D. B. Genic intolerance to functional variation and the interpretation of personal genomes. *PLoS genetics* **9**, e1003709 (2013).

7 van Eyk, C. L. *et al.* Analysis of 182 cerebral palsy transcriptomes points to dysregulation of trophic signalling pathways and overlap with autism. *Transl Psychiatry* **8**, 88 (2018).

8 Rao, A. R. & Nelson, S. F. Calculating the statistical significance of rare variants causal for Mendelian and complex disorders. *BMC Med Genomics* **11**, 53 (2018).
